# Characterization of a novel species of adenovirus from Japanese microbat and role of CXADR as its entry factor

**DOI:** 10.1038/s41598-018-37224-z

**Published:** 2019-01-24

**Authors:** Tomoya Kobayashi, Hiromichi Matsugo, Junki Maruyama, Haruhiko Kamiki, Ayato Takada, Ken Maeda, Akiko Takenaka-Uema, Yukinobu Tohya, Shin Murakami, Taisuke Horimoto

**Affiliations:** 1 Department of Veterinary Microbiology, Graduate School of Agricultural and Life Sciences, University of Tokyo, Tokyo, Japan; 2 Division of Global Epidemiology, Research Center for Zoonosis Control, Hokkaido University, Sapporo, Japan; 3 Laboratory of Veterinary Microbiology, Joint Faculty of Veterinary Medicine, Yamaguchi University, Yamaguchi, Japan; 4 Laboratory of Veterinary Microbiology, Department of Veterinary Medicine, College of Bioresource Sciences, Nihon University, Fujisawa, Japan

**Keywords:** Virology, Infection

## Abstract

Recently, bat adenoviruses (BtAdVs) of genus *Mastadenovirus* have been isolated from various bat species, some of them displaying a wide host range in cell culture. In this study, we isolated two BtAdVs from Japanese wild microbats. While one isolate was classified as *Bat mastadenovirus A*, the other was phylogenetically independent of other BtAdVs. It was rather related to, but serologically different from, canine adenoviruses. We propose that the latter, isolated from Asian parti-colored bat, should be assigned to a novel species of *Bat mastadenovirus*. Both isolates replicated in various mammalian cell lines, implying their wide cell tropism. To gain insight into cell tropism of these BtAdVs, we investigated the coxsackievirus and adenovirus receptor (CXADR) for virus entry to the cells. We prepared CXADR-knockout canine kidney cells and found that replication of BtAdVs was significantly hampered in these cells. For confirmation, their replication in canine CXADR-addback cells was rescued to the levels with the original cells. We also found that viral replication was corrected in human or bat CXADR-transduced cells to similar levels as in canine CXADR-addback cells. These results suggest that BtAdVs were able to use several mammalian-derived CXADRs as entry factors.

## Introduction

Adenovirus (AdV) is a non-enveloped, double-stranded DNA virus, and is divided into five genera: *Mastadenovirus*, *Atadenovirus*, *Ichtadenovirus*, *Siadenovirus*, and *Aviadenovirus*. Recently, several novel members of *Mastadenovirus* were identified, and more than 40 species were registered in the International Committee on Taxonomy of Viruses (ICTV). Interestingly, several reports indicated cross-species transmission of viruses in *Mastadenovirus*. For example, canine adenoviruses (CAdVs), the causative agents for canine hepatitis (CAdV1) or respiratory diseases (CAdV2), classified into species *Canine mastadenovirus A*, could be transmitted to various carnivore species including foxes^1,2^. Moreover, cross-species transmissions of viruses in *Mastadenovirus* even among primates including humans have also been reported^3–5^. Therefore, we need to revisit the possibility that host range of viruses in *Mastadenovirus* could be wider than previously thought.

Bats are known to be natural reservoirs of various zoonotic viruses such as severe acute respiratory syndrome (SARS) coronavirus, rabies virus, Nipah virus, and Marburg virus^6–9^. Therefore, many studies have been conducted to investigate bat-harbored viruses around the world, resulting in identification of numerous novel viruses. The bat adenoviruses (BtAdVs) have also been isolated from various species of microbats and macrobats inhabiting a variety of countries since they were first isolated from a common pipistrelle bat (*Pipistrellus pipistrellus*) Germany, in 2009^10–16^. Until recently, seven species of BtAdVs (namely *Bat mastadenovirus A to G*) have been registered by the ICTV, and are divided into three groups depending on the host family classification; group 1 viruses of *Bat mastadenovirus A, B, and G* isolated from *Vespertilionidae* bats, group 2 of *Bat mastadenovirus C* isolated from *Rhinolophidae* bat, and group 3 of *Bat mastadenovirus D, E, and F* isolated from *Miniopteridae* and *Pteropodidae* bats^14^.

Several reports suggest that BtAdVs have a broad host range in cell culture^10,12,14^. Their molecular mechanisms, however, remain unclear. The receptor on the cell surface is one of the major viral host range determinants^17–19^. Although several molecules have been reported as adenovirus receptors^20–24^, majority of adenoviruses, including CAdV2, use coxsackievirus and adenovirus receptors (CXADR or CAR) via their fiber proteins^25–27^. The CXADR is a 46-kDa type I transmembrane protein with an extracellular region composed of two immunoglobulin-like domains^26^, having a major role in forming cellular tight junctions^28^. CXADR homologs are conserved in vertebrates such as human, mice, rats, dogs, bats, and zebrafish^29^. Therefore, it is possible that BtAdVs may use CXADR as a functional receptor to infect various cell cultures.

In this study, we have isolated novel adenoviruses from fecal samples of Japanese wild microbats and characterized their biological properties. Moreover, we examined whether bat- and other-derived CXADRs could be involved in the entry for BtAdV infection.

## Results

### Isolation and identification of BtAdVs

**Figure 1:**
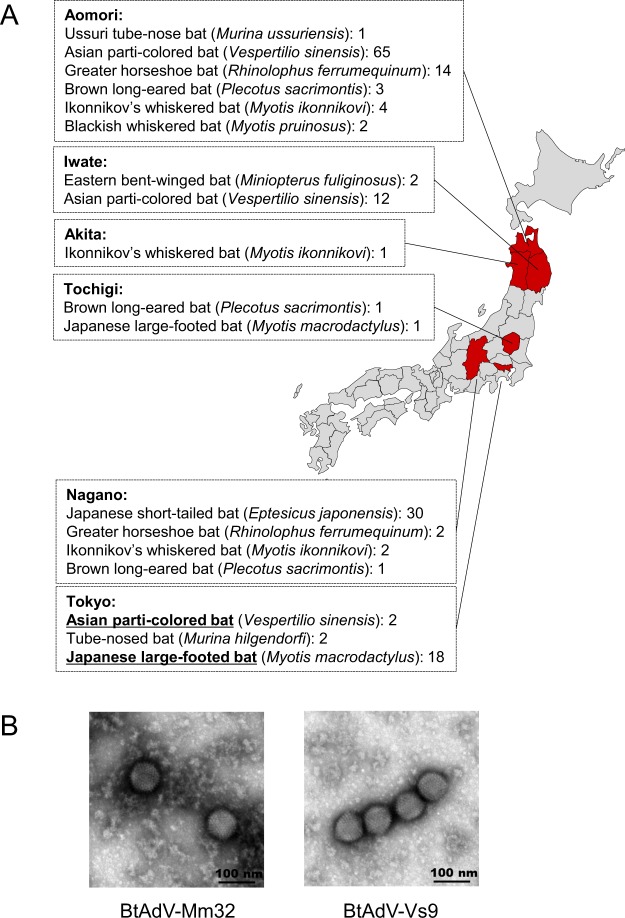
Isolation of BtAdVs from Japanese microbats. We collected 164 fecal samples from ten bat species captured in six prefectures in Japan. Bat common names and the numbers of samples are described for each prefecture. BtAdV-Mm32 and -Vs9 were isolated from the bats shown underlined, respectively (**A**). Both isolates were negatively-stained when observed by transmission electron microscope (**B**).

To examine whether AdVs exist in Japanese bats, we captured a total of 163 insectivorous bats in Aomori, Iwate, Akita, Tochigi, Tokyo, and Nagano prefectures of Japan (Fig. 1A). The captured bats were classified into 10 species by their morphological features and, in some cases, by *cytochrome b* gene sequencing. To isolate viruses, we inoculated antibiotics-treated fecal samples into several cell lines of different animal origins. Among them, we observed extensive CPE such as cell rounding, exfoliation, and death in Madin-Darby canine kidney (MDCK) cells, following inoculation with samples from *Myotis macrodactylus* and *Vesperitilio sinensis* microbats (Fig. S1). These two isolates were identified as having less than 100 nm particle size through a membrane filtration test, resistant to chloroform treatment, and susceptible to a pyrimidine analog 5-iodo-2′-deoxyuridine. Moreover, we observed adenovirus-like particles by transmission electron microscopic analysis (Fig. 1B). We strongly presume from these data that these two isolates were adenoviruses.

**Figure 2:**
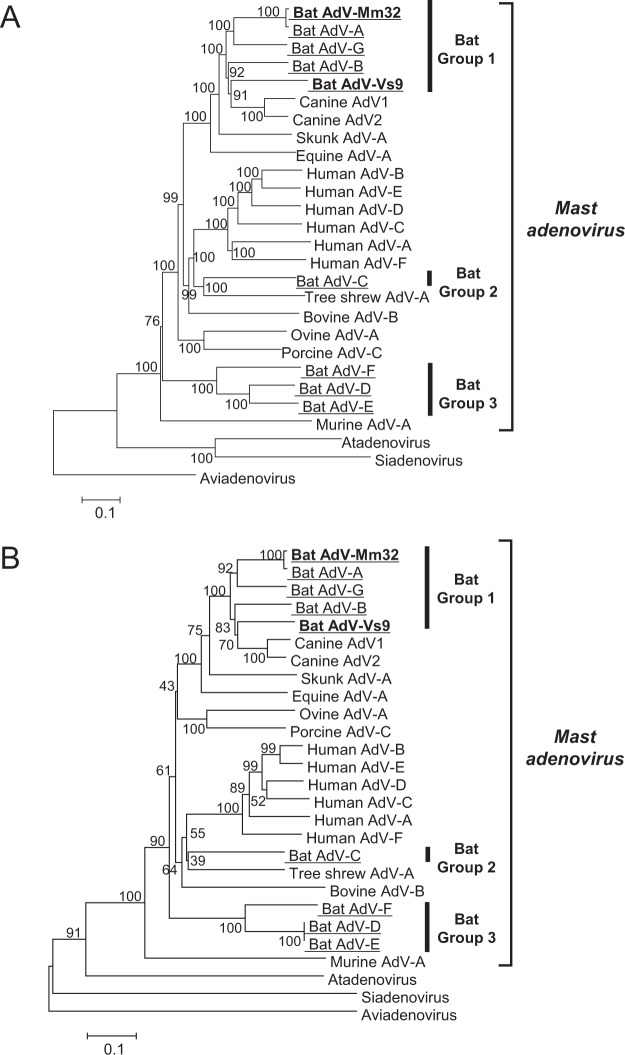
Phylogenetic classification of BtAdVs. A neighbor-joining tree was generated based on full-length genomic sequences (**A**) or DNA polymerase amino acid sequences (**B**). Bootstrap values are shown at the major nodes. Scale bar indicates the number of substitutions per site. All BtAdVs are underlined and novel isolates in this study are shown in bold.

To authenticate our presumption, we determined their complete genome sequences by a next-generation sequencing. BLASTn analysis of nucleotide sequence of *penton* gene indicated that the virus from *M. macrodactylus* (referred as BtAdV-Mm32) showed the highest identity (99%) to BtAdV in *Bat mastadenovirus A*, which had been isolated from *Myotis ricketti* bats in China^12^. On the other hand, the virus from *V. sinensis* (referred as BtAdV-Vs9) showed the highest identity (75%) to CAdV2 in *Canine mastadenovirus A*. For confirmation, we constructed a phylogenetic tree based on the complete genome sequences (Fig. 2A, Supplementary Data 1) or the DNA polymerase amino acid sequences (Fig. 2B, Supplementary Data 2). Both of our isolates belonged to BtAdV group 1 in the genus *Mastadenovirus*. Species demarcation in the genus *Mastadenovirus* is determined by a criterion that there is a 15% or more difference in the phylogenetic distance, based on distance matrix analysis of the DNA polymerase amino acid sequence, as described by the ICTV (https://talk.ictvonline.org/ictv-reports/ictv_9th_report/dsdna-viruses-2011/w/dsdna_viruses/93/adenoviridae). The phylogenetic distances between BtAdV-Vs9 and the viruses in group 1 species such as *Bat mastadenovirus A, B, G*, and CAdV1/2 in *Canine mastadenovirus A* were 22.0%, 23.9%, 19.8%, and 22.4/20.9%, respectively. Accordingly, BtAdV-Vs9 could be classified into a novel *Bat mastadenovirus* species (tentatively named *Bat mastadenovirus H*).

### Genomic organization of BtAdV isolates

We analyzed the whole genome sequences of BtAdV-Mm32 (31, 750 bp) and Vs9 (31, 218 bp). Both had 30 open reading frames (ORFs) homologous to other AdVs and inverted terminal repeats (ITRs) located at the 5′ and 3′ ends of the genomes (Table S1, Fig. S2). The amino acid sequence identities of our isolates with those of other bat or canine adenoviruses in each ORF have been shown (Tables S2 and S3). All deduced amino acid sequences of BtAdV-Mm32 showed the highest identity (95%–100%) to those of *Bat mastadenovirus A* in all ORFs (Table S2), confirming that the virus should be classified into this species. In contrast, BtAdV-Vs9 showed low sequence identities in most ORFs of any other BtAdVs (21–87% identity) and CAdVs (42–86% identity) (Table S3). These data support that BtAdV-Vs9 should form a novel *Bat mastadenovirus* species in genus *Mastadenovirus*.

### Pathogenicity of BtAdV isolates in mice

**Figure 3:**
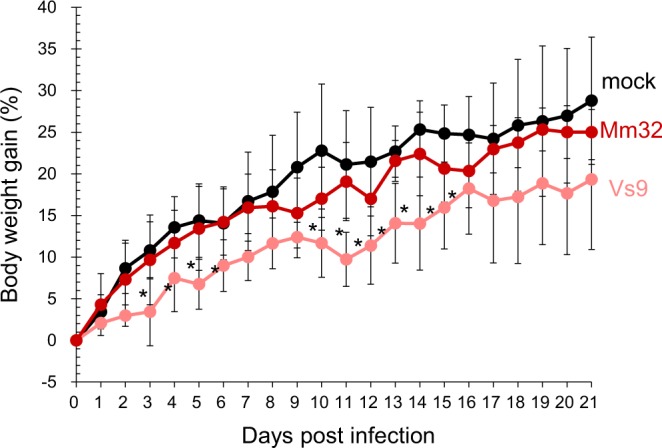
Pathogenicity of BtAdVs in mice. BALB/c mice (4-week old, n = 3) were intranasally inoculated with 10^5^ PFU of BtAdV-Mm32, -Vs9, or PBS (mock). Then, their body weights were measured daily for 21 days. Asterisks (*) reveal significant differences compared to mock-infected mice (p < 0.05 by *Dunnett*’s test).

A previous report indicated that some BtAdVs may cause mild pneumonia in bats^11^. However, there is no information about pathogenicity of BtAdVs in other animals. To approach this issue, we inoculated mice with these isolates intranasally or orally, and examined for disease signs or serology. Sera collected from orally-inoculated mice at 21 days post-inoculation were negative for neutralization antibodies. In contrast, sera collected from intranasally-inoculated mice became antibody-positive in the neutralization test; the antibody titers were 320, 160, and 320 in three mice infected with Mm32 and 20, 160, and 80 in three mice infected with Vs9, respectively. These data indicated that our BtAdV isolates were capable of infecting mice. None of the mice showed appreciable disease signs and died when intranasally-inoculated during the course of infection. However, the body weight gain of the BtAdV-Vs9-infected mice was significantly lower than that of mock-infected mice (Fig. 3). Additionally, we detected viral DNAs in the lung and intestine of 83% of sacrificed mice at 21 days post-infection with both viruses. These data indicated that although our isolates could infect mice, their pathogenicities were low.

### Cross-antigenicity of BtAdV isolates to CAdVs

**Figure 4:**
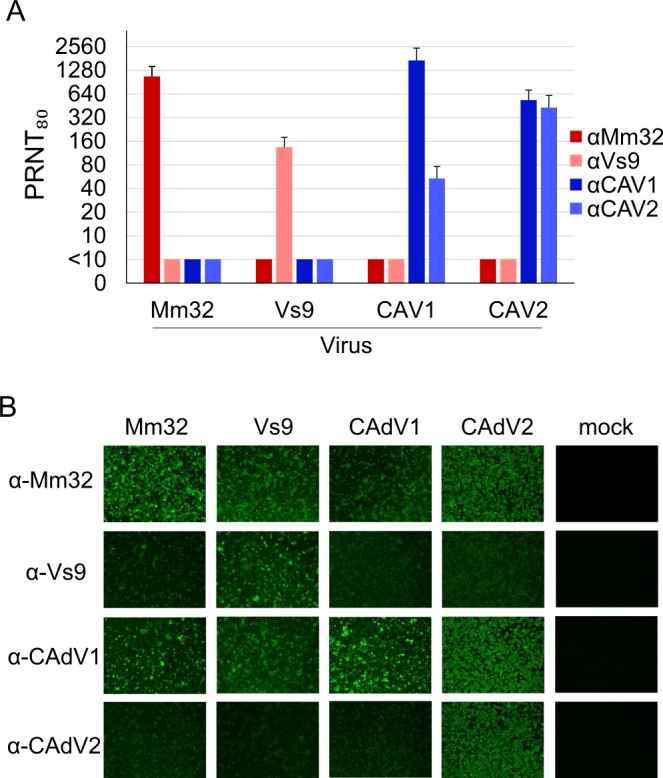
Antigenic cross-reactivity between BtAdVs and CAdVs. Viral neutralization titers were determined in homologous or heterologous combinations using BtAdV-Mm32, -Vs9, CAdV1, or CAdV2 and their respective antisera by plaque reduction assays in MDCK cells. An 80% reduction value in plaque numbers was used as neutralization titer (PRNT_80_). The data are reported as the mean titers with standard deviations for three independent experiments (**A**). BtAdV-Mm32, -Vs9, CAdV1, or CAdV2 were inoculated into cells at an MOI of 1. At 24 hpi, the cells were fixed and permeabilized. After blocking, each antiserum was used as the primary antibody in homologous or heterologous combinations. We used a secondary antibody conjugated with a fluorophore (Alexa 488) and observed the cells by fluorescent microscopy (**B**).

Since our isolates, especially BtAdV-Vs9, were closely related to CAdVs phylogenetically, we analyzed the antigenic cross-reactivity between BtAdVs and CAdVs, by using plaque reduction neutralization test (PRNT) and immunofluorescence assay (IFA). In the PRNT, we confirmed that each antiserum neutralized homologous virus with high titers and that CAdV1 and CAdV2 cross-neutralized each other as previously reported^30^ (Fig. 4A). However, no cross-neutralization was observed between the two BtAdVs and between BtAdVs and CAdVs. Contrastingly, in the IFA, anti-BtAdV-Mm32 and anti-CAdV1 sera showed strong reaction to heterologous as well as homologous viruses, whereas anti-BtAdV-Vs9 and anti-CAdV2 sera showed faint reaction to heterologous viruses compared to homologous viruses (Fig. 4B). These results indicated that undefined viral antigens, which did not contain neutralization epitopes, possessed antigenic cross-reactivities amongst BtAdVs, and between BtAdVs and CAdVs.

### Cellular tropism of BtAdV isolates

**Figure 5:**
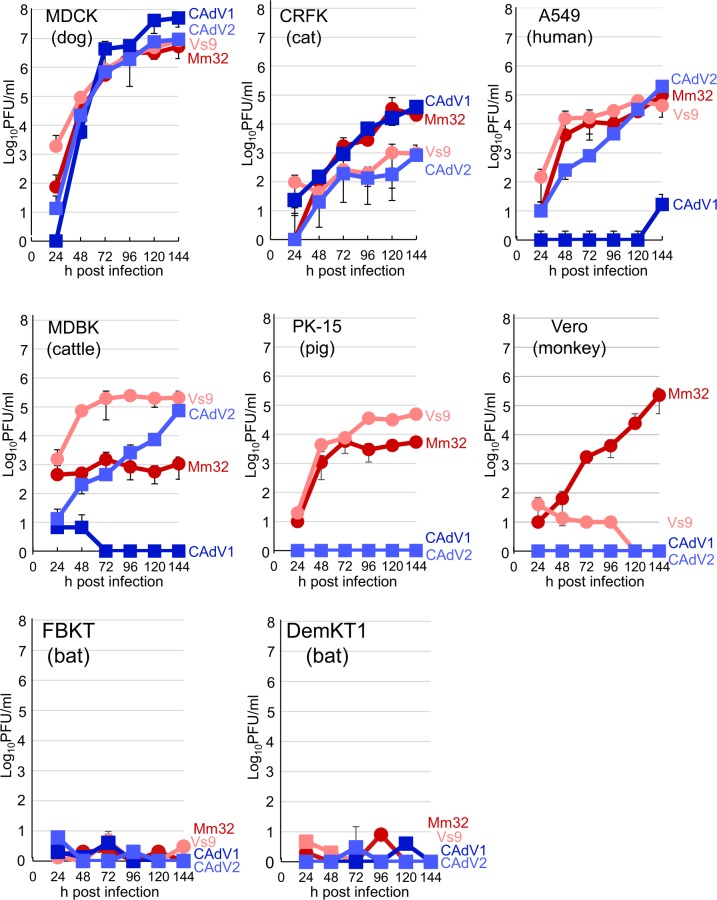
Viral growth kinetics in various mammalian cell lines. BtAdV-Mm32, -Vs9, CAdV1, or CAdV2 were inoculated into MDCK, Vero, A549, CRFK, MDBK, PK-15, FBKT, and DemKT1 cells at an MOI of 0.01. Following this, the supernatants were collected daily for 6 dpi. Viral titers were determined by plaque assay in MDCK cells. The data are reported as the mean titers with standard deviations for three independent experiments.

In previous reports, BtAdVs in *Bat mastadenovirus A and G*, and a virus isolated from *Eidolon helvum* had the ability to replicate in various mammalian cell lines^10,12,14^. To assess growth dynamics of our BtAdV isolates in cell culture, we inoculated eight cell lines of various animal origins with these viruses and CAdVs for comparison (Fig. 5). Interestingly, BtAdVs replicated in various mammalian cell lines other than FBKT and DemKT1 cells of macrobat origin. BtAdVs and CAdVs, both grew in MDCK cells with similar highest titers. Collectively, BtAdVs replicated efficiently in all non-bat-derived cell lines tested except Vero cells, where BtAdV-Vs9 showed poor growth. In contrast, CAdV1 or CAdV2 replicated in 2 or 4 non-bat-derived cell lines, respectively, indicating different cell tropism in cell culture between BtAdVs and CAdVs.

### Growth of BtAdV isolates in CXADR-knockout cells

**Figure 6:**
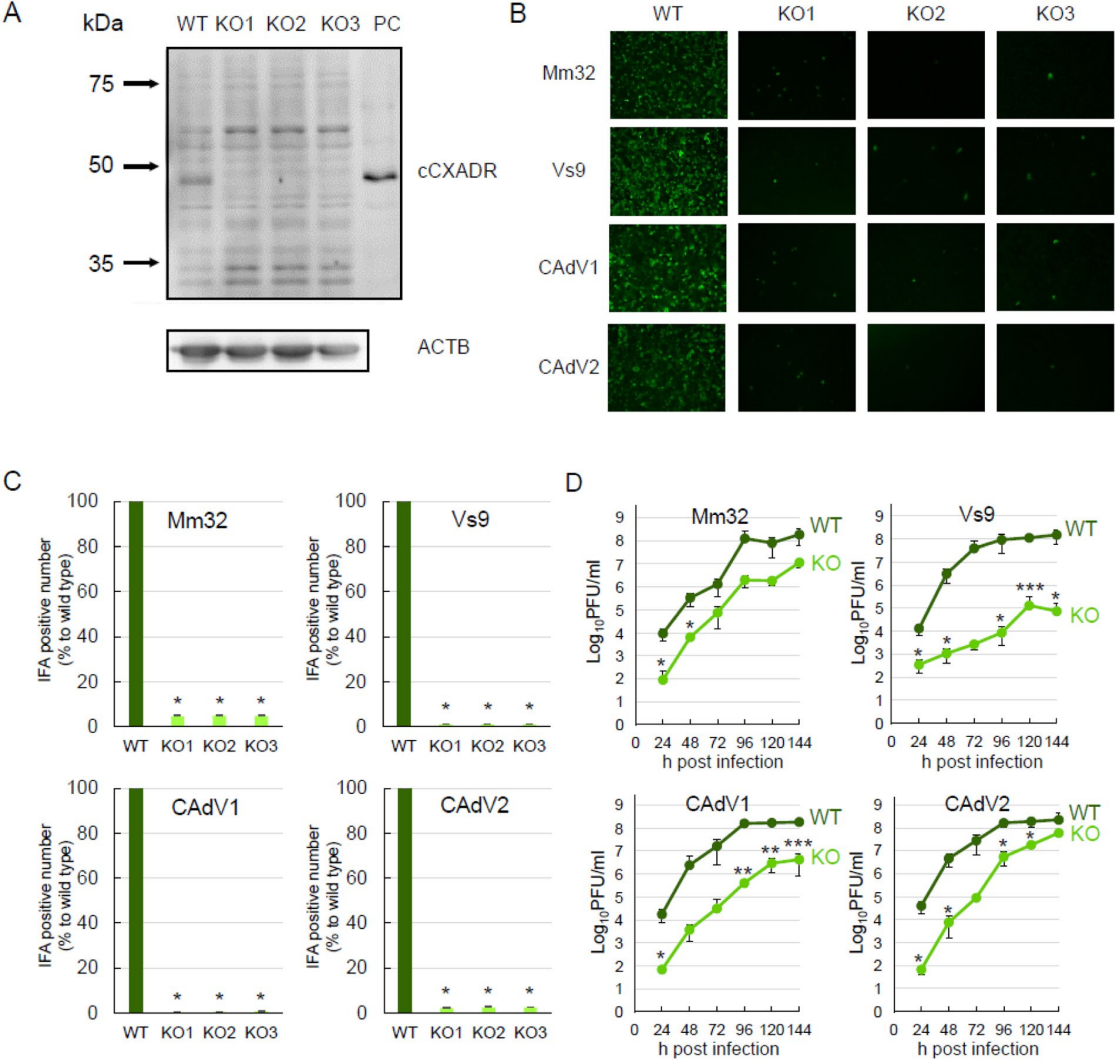
Viral growth in canine (c) CXADR-KO cells. cCXADR was not detected in three lines of CXADR-KO cells, unlike wild type (WT) cells, by a western blot analysis using a mouse anti-CXADR monoclonal antibody (CXADR E1). Plasmid-expressed cCXADR was used as a positive control (PC). ACTB was used as a loading control in each lane with the same amount of sample (**A**). WT or cCXADR-KO cells were inoculated with BtAdV-Mm32, -Vs9, CAdV1, or CAdV2 at an MOI of 1. At 24 hpi, virus antigens were detected by IFA using each antiserum under a fluorescence microscope (**B**). Numbers of IFA positive cells in panel B were quantified and reported as the mean values with standard deviations for three independent experiments. Asterisks (*) reveal significant differences compared to WT cells (p < 0.001 by *Dunnett*’s test) (**C**). WT or cCXADR-KO cells were inoculated with each virus at an MOI of 1 and the supernatants were collected and titrated daily for 6 days post-infection. The data are reported as the mean values with standard deviations for three independent experiments. Asterisks (*) reveal significant differences compared to WT cells (*p < 0.05; **p < 0.01 by Student’s *t* test) (**D**).

CXADRs have been identified as functional cellular receptors for CAdVs and some human AdVs^25–27^. To assess interaction between BtAdVs and CXADRs, we generated canine CXADR-knockout MDCK (cCXADR-KO) cells by using a CRISPR/Cas9 system^31^. We obtained three clones of cells (cCXADR-KO1, -KO2, and -KO3) and confirmed the lack of cCXADR expression by sequencing and western blot analysis (Fig. 6A). To analyze viral entry into these cells, BtAdVs or CAdVs were inoculated in either KO or wild-type (WT) cells, followed by detection of viral antigens by IFA at 24 h post-inoculation (hpi). The numbers of BtAdV as well as CAdV antigen-positive cells were much lower in KO than in WT cells (Fig. 6B,C), suggesting that attachment and entry of both viruses to the cells were hampered by knocking out the cCXADR. To totally evaluate viral growth in cCXADR-KO cells, we determined growth kinetics of BtAdVs or CAdVs (Fig. 6D). Viral titers of all the viruses were lower in KO than in WT cells at each time-point post-infection. These results indicate that cCXADR plays a crucial role in the viral entry and replication of BtAdVs as well as CAdVs in MDCK cells.

### Growth of BtAdV isolates in cCXADR-addback cells

**Figure 7:**
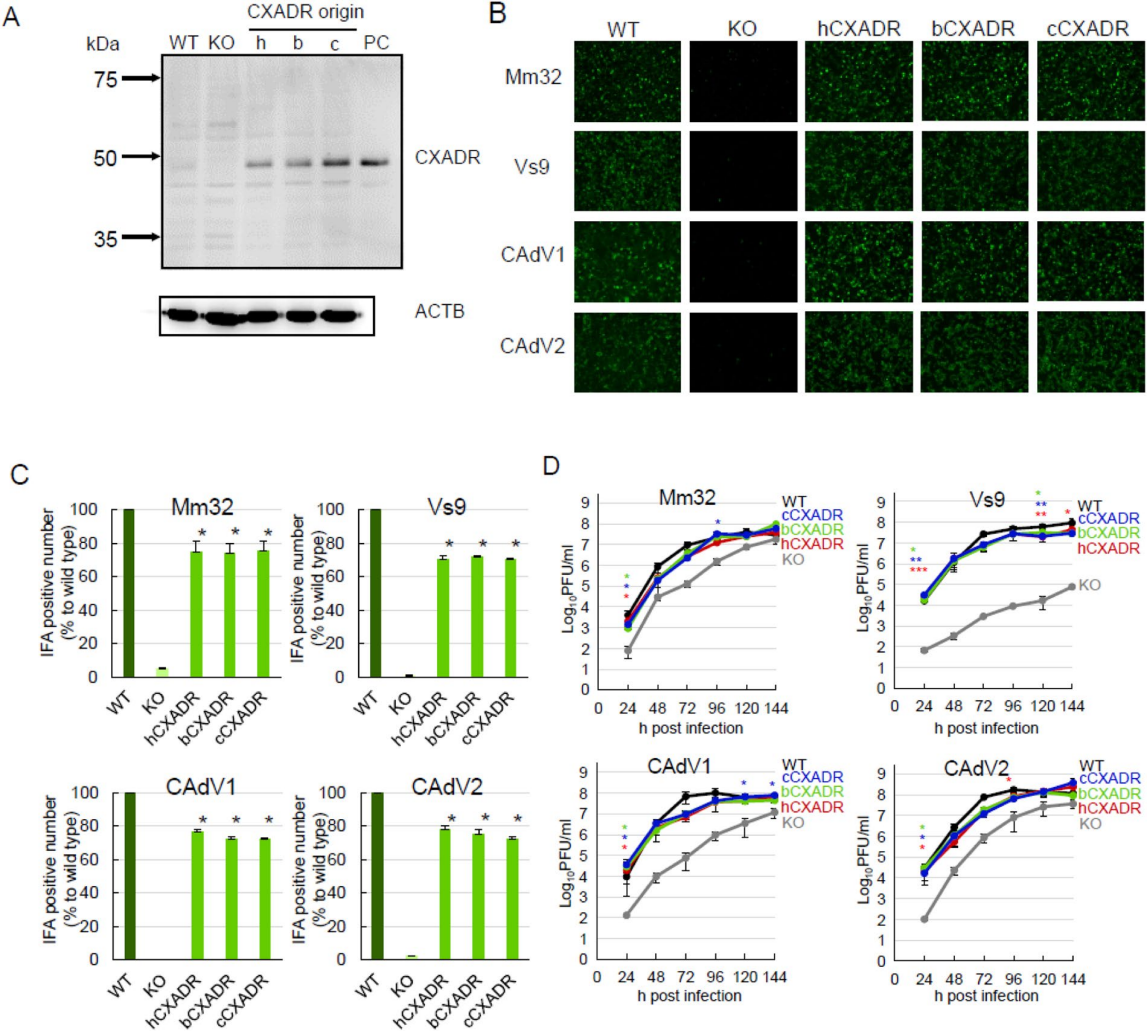
Viral growth in CXADR-addback or -transduced cells. Expressions of canine (c), human (h), or bat (b) CXADRs were rescued in these addback or transduced cells as revealed by western blot analysis using a mouse anti-CXADR monoclonal antibody (CXADR E1). Plasmid-expressed cCXADR was used as a positive control (PC). ACTB was used as a loading control in each lane with the same amount of sample (**A**). WT, cCXADR-KO, -addback, hCXADR or bCXADR-transduced cells were inoculated with each virus at an MOI of 1. At 24 hpi, viral antigens were detected by IFA using each antiserum under a fluorescence microscope (**B**). Numbers of IFA positive cells in panel B were quantified and reported as the mean values with standard deviations for three independent experiments. Asterisks (*) reveal significant differences compared to cCXADR-KO cells (p < 0.001 by *Dunnett*’s test) (**C**). WT, cCXADR-KO, -addback, hCXADR or bCXADR-transduced cells were inoculated with each virus at an MOI of 1 and the supernatants were collected and titrated daily for 6 days post-infection. The data are reported as the mean values with standard deviations for three independent experiments. Asterisks (*) reveal significant differences compared to cCXADR-KO cells (*p < 0.05; **p < 0.01 by Student’s *t* test) (**D**).

To validate that growth reduction of BtAdVs and CAdVs observed in cCXADR-KO cells was not due to off-target effects of CRISPR/Cas9-based knockout, we added *cCXADR* gene back to cCXADR-KO cells using a lentiviral vector. Restoration of expression of cCXADR in the cells was confirmed by western blot analysis (Fig. 7A). Then, we determined growth properties of BtAdVs or CAdVs in cCXADR-addback cells. Entry rates (Fig. 7B,C) and growth kinetics (Fig. 7D) of all viruses were rescued in cCXADR-addback cells to equivalent levels to WT cells. These data suggest that BtAdVs as well as CAdVs use cCXADR as an entry factor for their infection.

### Growth of BtAdV isolates in other animal-derived CXADR-transduced cells

To examine whether heterologous CXADRs could facilitate BtAdV growth in cells, we transduced human *CXADR* (*hCXADR*) and microbat *CXADR* (*bCXADR*) genes into cCXADR-KO cells and their expression was confirmed by western blot analysis (Fig. 7A). Entry rates of all viruses were recovered in hCXADR- or bCXADR-transduced cells with equivalent levels to those in cCXADR-addback cells (Fig. 7B,C). Growth kinetics of BtAdVs were fully restored in hCXADR- or bCXADR-transduced cells (Fig. 7D). These data suggest that BtAdVs could use CXADRs of several animal origins as entry factors for their infection.

### Attachment of BtAdV isolates to CXADRs

**Figure 8:**
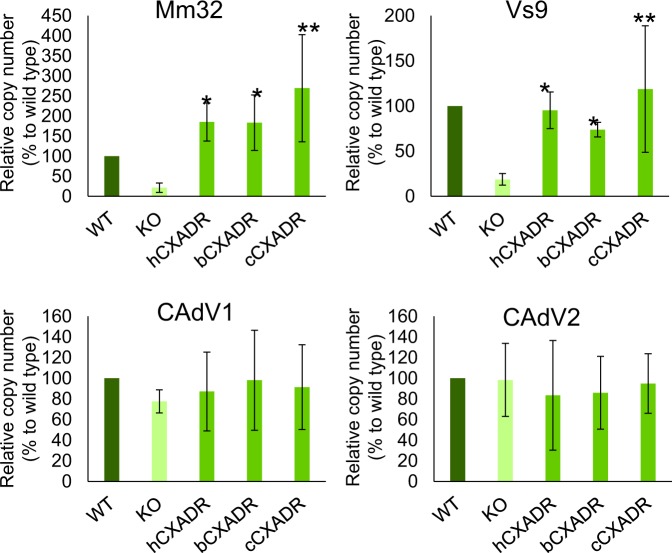
Viral attachment to WT, cCXADR-KO, -addback, hCXADR or bCXADR-transduced cells. Each of the viruses was inoculated to these cells followed by incubation for 1 h at 4 °C. Then, the cells were lysed and the DNA was extracted. The relative viral loads were measured by real-time PCR (n = 3). Asterisks (*) reveal significant differences compared to cCXADR-KO cells (*p < 0.05; **p < 0.01 by *Dunnett*’s test).

To directly assess interaction between viruses and CXADRs, we conducted a viral attachment assay, in which the cells were inoculated with viruses, incubated at 4 °C, and then washed thoroughly, followed by quantification of viruses attached to cell surface by real-time PCR. Significant reduction in attachment of BtAdVs was observed in cCXADR-KO cells. This reduction was fully rescued in cCXADR-addback and hCXADR- or bCXADR-transduced cells (Fig. 8), suggesting that cCXADR may act as a cellular receptor for BtAdV, and other animal-derived CXADRs could compensate for cCXADR functioning. In contrast, attachment of CAdVs to MDCK cells was not affected in cCXADR-KO cells, indicating that although CAdVs could bind to a non-specific molecule other than cCXADR in MDCK cells, this molecule is not functional for replication of CAdVs, as revealed by its entry and growth kinetic analysis in cCXADR-KO cells (Fig. 6B–D).

## Discussion

Since bats are known to harbor important zoonotic viral pathogens such as SARS coronavirus and Nipah virus^6–9^, vigorous surveillance conducted across the world has resulted in detection of numerous types of viruses, most of which are not well understood for their properties. Also, BtAdVs have been isolated from various species of microbats and macrobats across the world, and some showed a wide host range in cell culture, suggesting their potential for interspecies transmission^10–16^. Here, we report isolation of BtAdVs from Japanese microbats, with one isolate proposed to be assigned to a novel species of *Bat mastadenovirus*. In addition, we found that various mammalian-derived CXADRs could work as entry factors for propagation of BtAdVs in cell culture.

In this study, we isolated for the first time two BtAdVs from Japanese microbats. One isolate, BtAdV-Mm32, was phylogenetically closely related to a virus in species *Bat mastadenovirus A* (98% genome identity), which had been isolated from Chinese bats (Fig. 2). Both viruses were derived from *Myotis* bats, *M. macrodactylus* (Fig. S1) and *M. ricketti*, distribution areas of these two species did not overlap. Since *Myotis* bats belong to a highly species-rich genus, which inhabit across the world^32^, similar BtAdVs might be widely distributed on earth. Moreover, *M. macrodactylus* often forms mixed colonies with *Rhinolophus ferrumequinum*, *Murina hilgendorfi*, or *Miniopterus fuliginosus* in Japan. Although we did not detect viruses in these bat species, mode of their living may correlate to viral spread among bat colonies. Further studies using a large number of samples are expected to clarify bat-to-bat transmission of BtAdVs.

We isolated BtAdV-Vs9 from the Asian parti-colored bat, *V. sinensis* (Fig. S1), an insectivorous bat distributed across China, Korea, Mongolia, and Japan. Sometimes, *V. sinensis* makes a very long-distance flight (more than 300 km). Actually, during preparation of this manuscript, we successfully isolated a virus very closely-related to BtAd-Vs9 from the same bat species captured in Aomori prefecture, approximately 800 km far from the sampling site of BtAdV-Vs9 (our unpublished data). BtAdV-Vs9-related viruses are thus likely to be distributed in broad geographical areas of Japan and East Asia.

Although both BtAdV-Mm32 and -Vs9 were isolated from fecal samples, the viruses did not experimentally infect mice via oral route, unlike their infection causing ability via respiratory route (Fig. 3). With a previous report suggesting association of some BtAdVs with respiratory disease in bats^11^, it is thus likely that BtAdVs replicates more efficiently in respiratory rather than intestinal organs. Characterization of tissue tropism and pathogenicity of BtAdVs in bats may contribute to elucidating their interspecies transmission mode and zoonotic potential.

Interestingly, BtAdV-Vs9 was most closely related to CAdVs (Fig. 2), providing a possibility that BtAdV-Vs9-related viruses could infect canines, or be easily mutated to infect them. Our serological data indicated no cross-neutralization between these two viruses (Fig. 4). Therefore, CAdV vaccine widely used in companion dogs would not be effective against BtAdV infection, indicating that once BtAdV jumps over the species barrier, the infection may easily spread in dog population.

In this study, we suggested that CXADRs may act as functional receptors for propagation of BtAdVs in cell culture. However, considerable levels of viral replication were still observed in CXADR-KO cells (Fig. 6D). These data suggest the presence of multiple functional receptors in MDCK cells for BtAdVs and CAdVs. The studies with human adenoviruses have revealed the presence of alternative receptors, such as integrins or heparan sulfate proteoglycans, in addition to CXADRs^33,34^. Notably, group 2 and 3 BtAdVs possess integrin binding RGD (Arg-Gly-Asp) motifs in penton base proteins, which recognize integrins α_v_β_3_ and α_v_β_5_^33^. Contrastingly, while neither group 1 BtAdVs including BtAdV-Mm32 and -Vs9 nor CAdVs had the RGD motif in such proteins, our isolates possessed LDV (Leu-Asp-Val) motif in α_1_β_4_ integrin binding pIIIa minor capsid protein^35^. These observations imply that our isolates possibly use other molecules such as integrins on cell surface. These ideas indicate that host specificities of adenoviruses are regulated by multiple receptor molecules.

Since BtAdVs, including our isolates, infect a broad range of mammalian cell lines (Fig. 5), they have a potential for interspecies transmission. The receptors are one of the important determinants of viral host range (e.g. linkage formed between sialic acid and galactose on the terminus of sugar chain determines host specificity for influenza A virus)^17^. Our isolates possessed 4 out of 5 conserved amino acid residues in fiber proteins that are responsible for interaction with CXADR^36^ (Table 1) and may use several mammalian-derived CXADRs as their functional receptors. This notion suggests that BtAdVs may already have a potential to infect various mammals, including humans. Further characterization of BtAdVs is required to better understand their zoonotic potential and risk assessment on public health concern.

**Table 1:** Important amino acids of viral fiber protein for interaction with CXADR.

Virus	Amino acid positions of fiber protein					BtAdV Group
	370	372	381	384	441	
CAdV2	G*	**S**	**P**	**R**	**T**	—
CAdV1	**D**	N	**P**	**R**	**T**	
BtAdV-Mm32	S	**S**	**P**	**R**	**T**	**Group1**
BtAdV-Vs9	**D**	P	**P**	**R**	**T**	
BtAdV-A	S	**S**	**P**	**R**	**T**	
BtAdV-B	**D**	**S**	**P**	**K**	**T**	
BtAdV-G	S	**S**	**P**	**R**	**T**	
BtAdV-C	**D**	**S**	N	**R**	F	**Group2**
BtAdV-D	**D**	N	R	L	W	**Group3**
BtAdV-E	N	E	R	L	L	
BtAdV-F	I	K	I	I	W	

^*^Amino acids in bold were identified as important amino acids to interact with CXADR.

## Methods

### Bat samples

All bats were captured using a harp trap with the permission from the Ministry of the Environment, Japan, and each of the local governments from 2015 to 2016. Species of bats which were captured were mainly determined morphologically or in part genetically by sequencing the *cytochrome b* gene^37^ from intestinal cell debris contained in their fecal samples. To obtain fresh feces, captured bats were kept in a plastic pouch for 1 h, their feces were collected with a sterilized cotton bud and transferred to 1 ml of Dulbecco’s modified Eagle’s medium (DMEM) supplemented with 100 U/ml penicillin, 1 mg/ml streptomycin, 100 μg/ml gentamycin, and 2 μg/ml amphotericin followed by transportation in dry ice.

### Cells and viruses

Madin-Darby canine kidney (MDCK) cells, African green monkey kidney (Vero) cells, human adeno carcinoma (A549) cells, Madin-Darby bovine kidney (MDBK) cells, Crandell-Rees feline kidney (CRFK) cells, porcine kidney (PK-15) cells, cCXADR-KO cells, cCXADR-addback cells, and hCXADR- or bCXADR-transduced cells were maintained in DMEM supplemented with 5% fetal bovine serum (FBS). Macrobat-derived cells BKT, IndFSP1, YubFKT1, DemKT1, and FBKT were maintained in RPMI-1640 supplemented with 10% FBS^13^. Bat adenoviruses, BtAdV-Mm32 and -Vs9, isolated in this study, and canine adenoviruses, CAdV1 (D43 strain) and CAdV2 (Toronto strain), isolated from domestic dogs, were propagated in DMEM with 1% FBS in MDCK cells and used for experiments.

### Virus isolation

The fecal samples were suspended in DMEM containing antibiotics and then centrifuged at 10,000 × *g* for 15 min at 4 °C. The supernatants were diluted 10 folds with DMEM containing antibiotics and inoculated into cell lines described in the previous section. After absorption for 1 h at 37 °C, the inoculum was removed, the cells were washed with phosphate-buffered saline (PBS), and then added to fresh DMEM with 1% FBS. The sample-inoculated cells were blindly passaged three times with daily observation to check CPE. The supernatants were obtained from the cells showing CPE, biologically cloned by limiting dilutions, and proceeded for virus identification.

### Virus identification

To determine the approximate particle size of the viral isolates, we examined infectivity of the flowthrough using a 100-nm membrane filter. Chloroform-resistance test was performed as follows: 200 µl of chloroform or control DMEM was added to 1.8 ml of viral solution and then incubated at room temperature for 15 min. The mixture was centrifuged at 750 × g for 10 min. The supernatants were titrated in MDCK cells to determine the 50% tissue culture infectious dose (TCID_50_). One hundred microliters of 10-fold serial dilutions of each BtAdVs were inoculated into cells of 96-well plates and incubated for 5 days at 37 °C. Wells showing CPE were counted, and the titers were calculated by the Reed-Muench method. Chloroform susceptibility was defined when more than 10^4^ TCID_50_ reduction was observed in chloroform-treated with respect to control samples. Transmission electron microscopy was used for observation of the isolates. Briefly, each isolate was incubated in glutaraldehyde (final concentration of 0.25%) at 4 °C for 1 week and applied on mesh grids coated with a collodion film. The grid was then stained with phosphotungstic acid for observation.

For genome sequencing, viral DNA was extracted from 200 µl of viral solution by QIAamp DNA mini kit (QIAGEN) and applied for next-generation sequencing using Ion PGM^TM^ (Thermo Fisher Scientific) according to the manufacturer’s protocol. The sequence result with BtAdV-Mm32 was mapped to the data in *Bat mastadenovirus A* by CLC genomic workbench version 8 (CLC bio). The sequence data with BtAdV-Vs9 was assembled *de novo* by the same software. Sequences in the genomic regions which could not be assembled, or had low sequence reliability, were checked and determined by Sanger sequencing, with the use of specific primers in an automated sequencer (Life technologies, Applied biosystems 3170 xl).

### Phylogenetic analysis

A neighbor-joining tree based on the complete genome sequences was generated with the Kimura’s two-parameter model^38^. The phylogenetic tree was generated using ClustalW and MEGA version 7.0 software^39^. The accession numbers of sequences used in these trees are as follows: BtAdV-Mm32 (LC385828), BtAdV-Vs9 (LC385827), Bat AdV-A (GU226970), Bat AdV-B (JN252129), Bat AdV-C (KT698853), Bat AdV-D (KT698856), Bat AdV-E (KT698852), Bat AdV-F (KX961095), Bat AdV-G (KX871230), Bovine AdV-B (NC_001876), Canine AdV1 (NC_001734), Canine AdV2 (AC000020), Human AdV-A (NC_001460), Human AdV-B NC_011203), Human AdV-C (NC_001405), Human AdV-D (NC_010956), Human AdV-E (NC003266), Human AdV-F (NC_001454), Skunk AdV-A (NC_027708), Porcine AdV-C (NC_002702), Tree shrew AdV-A (AC000190), Murine AdV-A (NC_000942), Equine AdV-A (JN418926), Ovine AdV-A (NC_002513), Ovine AdV-D (NC_004037), Fowl AdV-A (NC_001720), and Frog AdV-A (NC_002501). The alignment data among genome sequences of adenoviruses will be provided upon request.

### Growth kinetics

BtAdVs or CAdVs were inoculated into respective cells at a multiplicity of infection (MOI) of 0.01. After incubation for 30 min at 37 °C, the inocula were completely removed. After washing, the cells were maintained in DMEM with 1% FBS and the supernatants were collected daily up to 6 days post-infection (dpi). To measure the viral titers, we performed a plaque assay with MDCK cells. After virus absorption to the cells in 12-well plates for 1 h at 37 °C, the inocula were removed. Then, Eagle’s minimal essential medium (MEM) supplemented with 0.3% BSA and 0.8% agarose was overlaid on the cells. At 5 dpi, the agarose gels were removed and the plaques were stained with 0.1% crystal violet to count PFU of the viruses.

### *In vivo* experiments

To assess viral pathogenicity in mice, we inoculated 10^5^ PFU of either BtAdV-Mm32 or -Vs9 into 4-week-old female BALB/c mice (n = 3) (Japan SLC) intranasally or orally. The disease signs and body weights were recorded daily for 21 dpi.

### Plaque-reduction neutralization test

Antisera to BtAdVs were obtained from the mice infected intranasally with either BtAdV-Mm32 or -Vs9 at 21 dpi. Antisera to CAdVs were obtained from the guinea pigs immunized intraperitoneally with either CAdV1 (D43 strain) or CAdV2 (Toronto strain). Fifty microliters of 2-fold serial dilutions of each antiserum were prepared in DMEM. An equal volume of the virus suspension containing 100 PFU of either BtAdV-Mm32, -Vs9, CAdV1, or CAdV2 was added to each serum dilution in homologous or heterologous combinations of virus and antiserum. After incubation for 30 min at 37 °C, 100 µl of the mixture was titrated by plaque assay. At 5 dpi, we counted PFU and calculated 80% plaque-reduction neutralizing titers.

### Indirect immunofluorescence assay

The MDCK cells were seeded in a 24-well plate and infected with each virus at an MOI of 1. After viral adsorption for 1 h at 37 °C, the inoculum was removed, and the infected cells were maintained in DMEM with 1% FBS for 24 h. The cells were then fixed with 4% paraformaldehyde for 15 min, permeabilized with 0.1% Triton X-100 for 15 min, and blocked with Block Ace (DS Pharma Biomedical) for 45 min. After washing with PBS containing 0.1% Tween-20 (PBS-T), antisera to viruses were used for probing as the primary antibodies for 45 min. Next, after washing with PBS-T, goat anti-mouse Alexa 488 or goat anti-guinea pig Alexa 488 antibodies (Abcam) were used as the secondary antibody followed by incubation for 45 min. Finally, after washing, the cells were observed by fluorescent microscopy (Axio Vert. A1; Carl Zeiss).

### Establishment of cCXADR-KO cells

The c*CXADR* gene was knocked out using CRISPR/Cas9 technology in MDCK cells. We designed a single guide RNA to target exon 2 of *CXADR* (5ʹ ACCCTTAGTCCAGAAGACCA 3ʹ), which exists in all cCXADR transcriptional variants deposited in NCBI database, and constructed the plentiCRISPR vector (a gift from Dr. Feng Zhang, Addgene plasmid #52961)^31^ expressing the guide RNA. The plasmids with the gene-of-interest were transfected into cells at 60% confluency in a 12-well plate. At 24 h post-transfection, the culture medium was replaced with fresh medium containing puromycin (final concentration 10 µg/ml). After monolayer formation, the cells were passaged into a 10 cm dish for colonization. We selected several cell colonies and assessed absolute *cCXADR* knockout from host genome of each colony by sequencing. Then, cCXADR-KO was confirmed by western blot analysis. Finally, we obtained three clones, CXADR-KO1, -KO2, and -KO3.

### Western blot analysis of CXADR

After the medium was completely removed and washed with PBS, the cells were lysed with lysis buffer (20 mM Tris-HCl, pH 7.5, 0.1% Triton X-100, 100 mM NaCl, 30 mM KCl, 1 mM EDTA, and 1 × protease inhibitor cocktail (Roche)) for 10 min at 4 °C. Lysates were centrifuged at 10,000 × *g* for 5 min. Equal volumes of 2 × SDS sample buffer were added to the supernatant and boiled for 5 min. The samples were then proceeded for SDS-PAGE and the proteins were transferred onto a polyvinylidene difluoride membrane. The membranes were blocked with Block Ace in PBS-T for 45 min, followed by incubation with mouse anti-CXADR (E1) monoclonal antibody (Santa Cruz Biotechnology) as the primary antibody for 45 min. After washing, the membrane was incubated with a sheep anti-mouse IgG, HRP-linked F(ab′)2 fragment (GE Healthcare) as the secondary antibody for 45 min, followed by incubation with Chemi-Lumi One (Nacalai Tesque). The signals were detected by an ImageQuant LAS 4000 (Fujifilm).

### Addback or transduction of *CXADR* genes into CXADR-KO cells

The cDNA of *cCXADR* (LC385829) was generated from total RNA extracted from MDCK cells. The cDNA of human *CXADR* (*hCXADR*: NM_001338) was synthesized from the mRNA extracted from A549 cells. The cDNA of bat *CXADR* (*bCXADR*: LC385830) was prepared from mRNA extracted from the kidney tissue of fresh carcass of *V. sinensis*. Each *CXADR* sequence was amplified with specific primers. The amplicon was cloned into a pHR-SIN-CSGW (pS) lentiviral vector between *Bam* HI and *Not* I digestion sites^40,41^. To generate recombinant lentivirus, we co-transfected pS-CXADR, p8.9QV^41^, and pCAGGS-expressing vesicular stomatitis virus G protein into HEK 293 T cells. The CXADR-expressing lentiviral vectors were inoculated into the CXADR-KO cells. CXADR expression was confirmed by western blot analysis.

### Viral attachment assay

The monolayered cells in a 12-well plate were infected with each virus at an MOI of 1. After viral absorption to the cells for 1 h at 4 °C, the inoculum was removed and the cells were washed three times with ice-cold PBS. Then, the DNA was extracted from the cells with the QIAamp DNA Mini Kit (QIAGEN) according to the manufacturer’s protocol. The relative numbers of viruses attached to the cells were quantized by a real-time PCR assay using KOD SYBR® qPCR Mix (TOYOBO). We created specific primer sets to detect BtAdV-Mm32, BtAdV-Vs9, CAdV1, and CAdV2 (Table S4). The viral loads were normalized by amplifying the canine *ACTB* gene using a specific primer set (Table S4).

### Statistics

The data with body weight gains in the animal experiments, viral entry assays by IFA, viral attachment assays by real-time PCR, and viral growth kinetics of CXADR-addback or transduced cells were analyzed using the *Dunnett*’s test to determine the statistical significance of differences. The data with viral growth kinetics in cCXADR-KO cells were analyzed by Student’s *t*-test with two tailed analysis to determine the statistical significance of differences.

### Ethics statement

Our animal study protocol was conducted in accordance with the Regulations for Animal Care at the University of Tokyo and was approved by the Animal Experiment Committee of the Graduate School of Agricultural and Life Sciences at the University of Tokyo (approval number P17-149).

## Supplementary information

Supplementary Information

Supplementary Data 1

Supplementary Data 2
