# Supplementary material for: Characterization of a novel species of adenovirus from Japanese microbat and role of CXADR as its entry factor
Source: Sci Rep. 2019 Jan 24;9:573. doi: 10.1038/s41598-018-37224-z (PMC6345744; doi:10.1038/s41598-018-37224-z)
Supplement: Supplementary file 1 — Supplementary Information [file 41598_2018_37224_MOESM1_ESM.pdf]

Supplementary Information

Characterization of a novel species of adenovirus from Japanese microbat and role of CXADR as its entry factor

Tomoya Kobayashi, Hiromichi Matsugo, Junki Maruyama, Haruhiko Kamiki, Ayato Takada, Ken Maeda, Akiko Takenaka-Uema, Yukinobu Tohya, Shin Murakami & Taisuke Horimoto

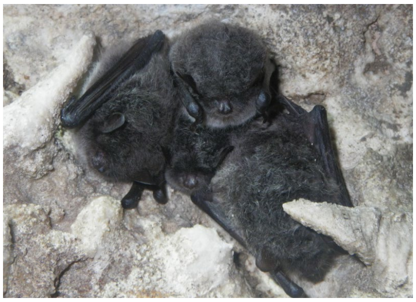

Myotis macrodactylus

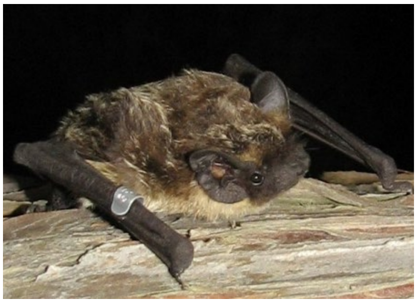

Vespertilio sinensis

Fig. S1 Bat species from which we isolated BtAdVs. BtAdV-Mm32 and Vs9 were isolated from *Myotis macrodactylus or Vespertilio sinensis*, respectively. We took all these photos.

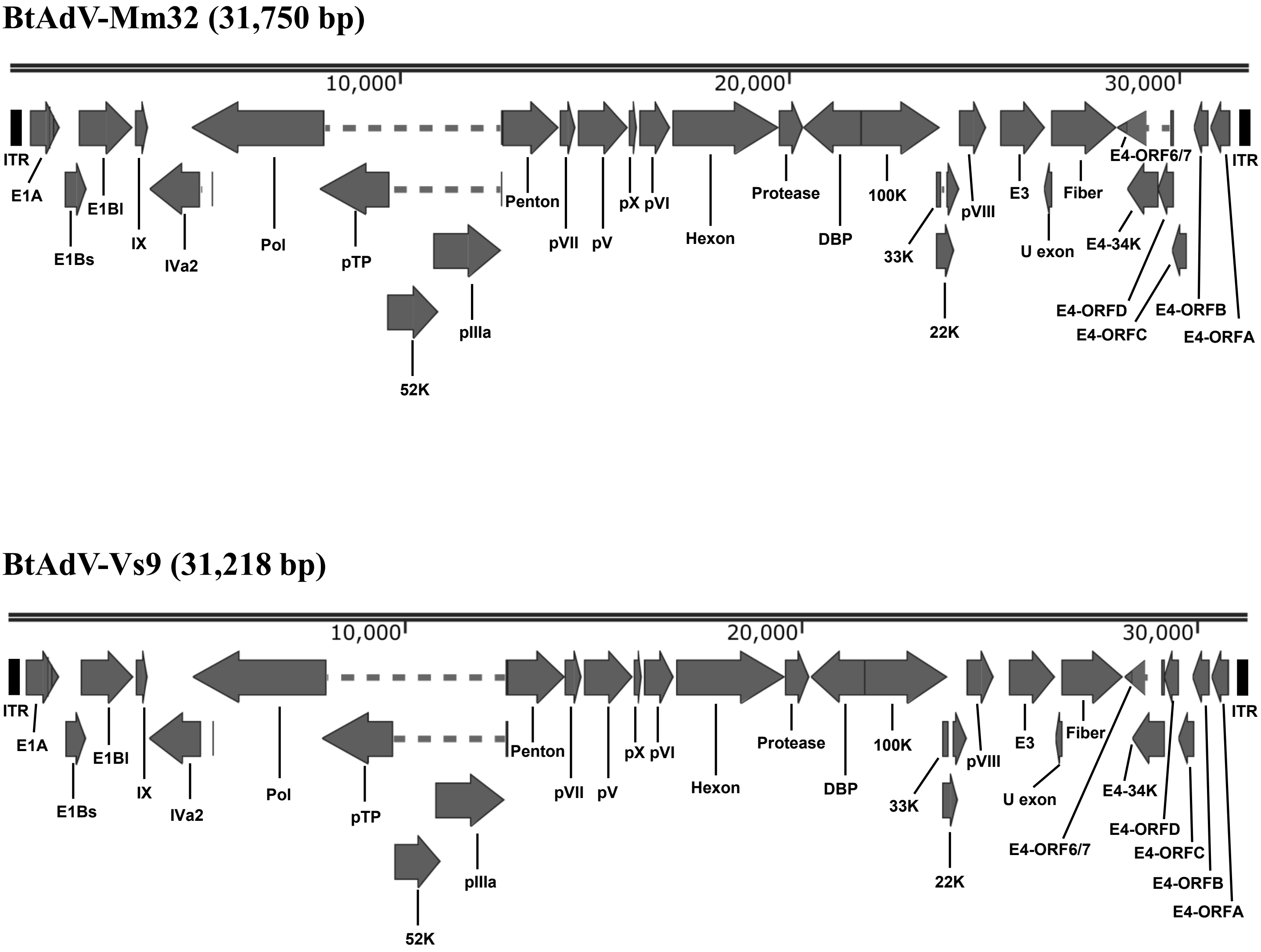

Fig. S2 Genome organization of BtAdV-Mm32 and -Vs9. The total genome length is shown next to the strain name. Each arrow indicates protein-coding ORF and its direction. Black square shows ITR.

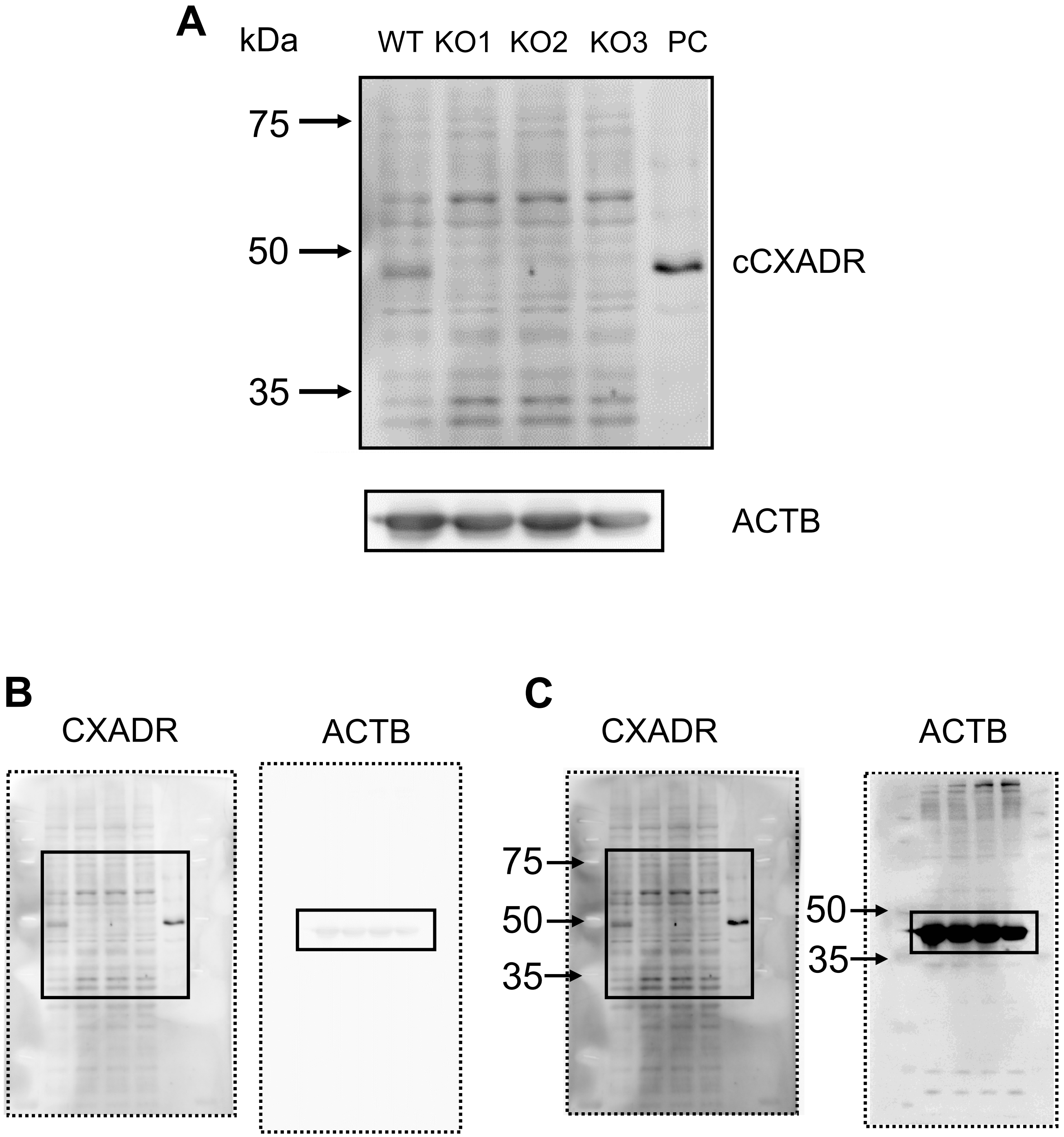

Fig. S3. Expression of canine (c) CXADR. cCXADR was not detected in three lines of CXADR-KO cells, unlike wild type (WT) cells, by a western blot analysis using a mouse anti-CXADR monoclonal antibody (CXADR E1). Plasmid-expressed cCXADR was used as a positive control (PC). ACTB was used as a loading control in each lane with the same amount of sample. This gel/blot was shown in Figure 6A (A). Multiple exposure images of the full-size gels/blots are also shown (B, C).

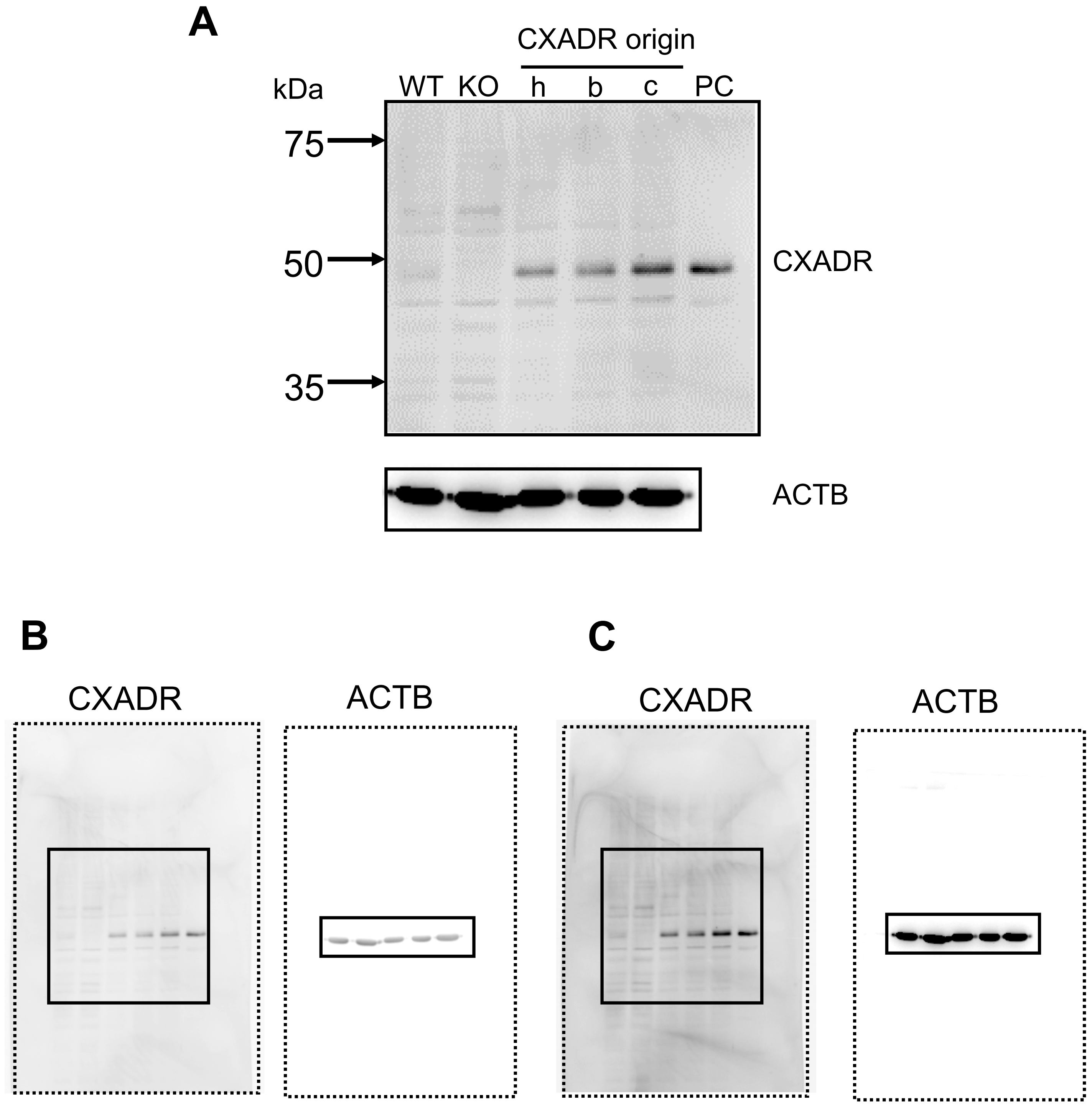

Fig. S4. Expressions of canine (c), human (h), or bat (b) CXADRs. They were rescued in these addback or transduced cells as revealed by western blot analysis using a mouse anti-CXADR monoclonal antibody (CXADR E1). Plasmid-expressed cCXADR was used as a positive control (PC) ACTB was used as a loading control in each lane with the same amount of sample. This gel/blot was shown in Figure 7A (A). Multiple exposure images of the full-size gels/blots are also shown (B, C).

Table S1. Genomic loci of BtAdVs genes and probable functions of their coding proteins.

**Gene**	**Genomic position (nt)**		**Function**
	**BtAdV-Mm32**	**BtAdV-Vs9**	
**E1A**	536-1034, 1118-1278	462-1014, 1094-1293	Transcriptional activator
**E1Bs**	1424-1972	1466-1984	Small T-antigen
**E1Bl**	1786-3165	1828-3165	Large T-antigen
**pIX**	3231-3551	3231-3521	Minor capsid protein
**IVa2**	3575-4890, 5190-5202	3555-4882, 5161-5173	DNA packaging
**Pol**	4675-8094, 12623-12631	4646-8053, 12581-12589	DNA polymerase
**pTP**	7590-9770, 12623-12631	7912-9732, 12581-12589	DNA terminal protein
**52K**	9697-11010	9757-10935	DNA packaging protein
**pIIIa**	10886-12610	10802-12553	Minor capsid protein
**Penton**	12657-14087	12613-14046	Major capsid protein
**pVII**	14119-14523	14074-14484	Minor core protein
**pV**	14589-15875	14551-15780	Minor core protein
**pX**	15901-16110	15805-16011	Minor core protein
**pVI**	16159-16959	16060-16806	Minor capsid protein
**Hexon**	17029-19755	16879-19605	Major capsid protein
**Protease**	19767-20387	19607-20224	Endopeptidase

**DBP**	20393-21847	20264-21622	DNA binding protein
**100K**	21860-23911	21634-23718	Hexon assembly protein
**33K**	23793-23937, 24050-24399	23588-23744, 23857-24212	DNA packaging protein
**22K**	23793-24260	23588-23986	DNA packaging protein
**pVIII**	24403-25071	24214-24894	Minor capsid protein
**E3**	25438-26586	25274-26425	Modulates immune response
**U exon**	26559-26762	26428-26595	Viral replication
**Fiber**	26761-28428	26594-28144	Major capsid protein
**E4-ORF6/7**	28442-28684, 29814-29861	28166-28387, 29121-29168	Transcriptional activator
**E4-34K**	28685-29467	28389-29168	Double strand break repair
**E4-ORF4**	29469-29861	29170-29550	Induced cell death
**E4-ORF3**	29840-30214	29553-29924	Shut-down of cellular protein
**E4-ORF2**	30394-30753	29905-30303	Unknown
**E4-ORF1**	30845-31318	30391-30783	Oncogenicity

Table S2. Amino acid identity of BtAdV-Mm32 compared to other BtAdVs and CAdVs.

**Viruses**	**Amino acid identity to BtAdV-Mm32 (%)**																							
	**E1A**	**E1Bs**	**E1Bl**	**pIX**	**IVa2**	**Pol**	**pTP**	**52K**	**pIIIa**	**Penton**	**pVII**	**pV**	**pX**	**pVI**	**Hexon**	**Protease**	**DBP**	**100K**	**33K**	**22K**	**pVIII**	**U exon**	**Fiber**	**E4-34K**
**BtAdV-Vs9**	41	42	50	53	72	74	80	74	76	83	77	59	83	72	87	78	70	73	59	53	75	65	42	50
**BtAdV-A**	95	97	98	95	98	98	99	98	99	100	98	96	100	95	100	98	99	98	98	98	99	100	98	98
**BtAdV-B**	46	47	53	48	75	74	84	75	78	82	71	60	84	67	86	80	70	78	63	56	80	74	48	49
**BtAdV-C**	21	25	25	42	64	60	64	59	52	65	34	30	58	50	72	65	43	56	43	43	56	32	25	26
**BtAdV-D**	39	17	25	41	63	57	61	59	50	63	39	22	55	45	70	58	53	54	60	41	54	43	26	27
**BtAdV-E**	35	23	25	31	60	56	58	55	52	65	46	21	56	42	71	59	48	55	52	38	56	32	24	24
**BtAdV-F**	20	23	27	42	58	59	52	55	51	63	46	25	57	44	69	56	45	53	53	34	56	39	23	26
**BtAdV-G**	48	58	63	60	79	78	88	82	85	89	84	72	85	80	90	88	79	81	78	67	86	74	50	63
**CAdV1**	44	45	50	52	72	74	85	77	78	84	74	60	83	71	86	78	66	74	57	60	76	80	45	49
**CAdV2**	40	47	51	54	76	75	86	78	80	83	74	62	82	73	86	82	66	75	59	58	80	80	50	50

Table S3. Amino acid identity of BtAdV-Vs9 compared to other BtAdVs and CAdVs.

Viruses	Amino acid identity to BtAdV-Vs9 (%)																							
	**E1 A**	**E1Bs**	**E1Bl**	**pIX**	**IVa2**	**Pol**	**pTP**	**52K**	**pIIIa**	**Penton**	**pVII**	**pV**	**pX**	**pVI**	**Hexon**	**Protease**	**DBP**	**100K**	**33K**	**22K**	**pVIII**	**U exon**	**Fiber**	**E4-34K**
**BtAdV-Mm32**	41	42	50	53	72	74	80	74	76	83	77	59	83	72	87	78	70	73	59	53	75	65	42	50
**BtAdV-A**	43	46	50	52	72	74	80	75	76	83	77	58	83	70	87	79	70	72	60	53	76	65	43	49
**BtAdV-B**	49	44	58	54	75	76	84	73	79	86	85	61	85	64	85	82	69	75	81	63	79	72	40	45
**BtAdV-C**	22	24	28	50	61	60	62	56	50	67	35	27	61	44	72	61	53	53	36	50	53	33	25	26
**BtAdV-D**	33	21	26	31	64	57	58	62	50	65	37	24	57	45	70	60	53	55	62	37	49	51	25	27
**BtAdV-E**	26	21	24	30	60	56	58	59	52	64	35	23	57	47	71	59	44	54	58	36	51	47	19	26
**BtAdV-F**	27	18	26	35	58	59	54	61	50	62	44	23	55	50	69	58	48	53	58	36	53	42	24	25
**BtAdV-G**	43	38	54	62	74	75	83	77	78	84	81	66	81	72	85	81	73	75	64	62	77	63	44	49
**CAdV1**	43	45	55	56	75	76	82	75	77	86	76	65	80	69	85	82	64	73	71	67	74	69	42	48
**CAdV2**	44	50	58	55	75	77	83	77	78	86	80	66	82	71	85	84	64	74	56	63	75	70	43	46

Table S4. Primer sets used for quantification of viral attachment by qPCR.

Primer name	5'-3'
rt Mm32 pol F	TCTCATTGGAAGGGGCCAAC
rt Mm32 pol R	TGTTTCAGCGGCCTACCATT
rt Vs9 pol F	CCTCCCCAAGTGTCCCATTC
rt Vs9 pol R	GTTGAGACCTACACGTGGCA
rt CAV1 pol F	GCATAAAAGGGCCTTCAGCG
rt CAV1 pol R	TTTGTCGTAGCGTACCCACC
rt CAV2 pol F	TGCGGGTAGGTCATAGAGGT
rt CAV2 pol R	CACTAAGCATTTCCGCCGTG
rt ACTB F	GGACCTGACCGACTACCTCA
rt ACTB R	GTCCAGGGCCACATAACACA
