# Supplementary material for: Characterization of a novel species of adenovirus from Japanese microbat and role of CXADR as its entry factor
Source: Sci Rep. 2019 Jan 24;9:573. doi: 10.1038/s41598-018-37224-z (PMC6345744; doi:10.1038/s41598-018-37224-z)
Supplement: Supplementary file 2 — Supplementary Data 1 [file 41598_2018_37224_MOESM2_ESM.docx]

>Bat AdV-C

------------------------------------------------------------

------------------------------------------------------------

------------------------------------------------------------

------------------------------------------------------------

------------------------------------------------------------

------------------------------------------------------------

------------------------------------------------------------

------------------------------------------------------------

------------------------------------------------------------

------------------------------------------------------------

------------------------------------------------------------

------------------------------------------------------------

------------------------------------------------------------

------------------------------------------------------------

------------------------------------------------------------

------------------------------------------------------------

------------------------------------------------------------

------------------------------------------------------------

------------------------------------------------------------

------------------CAATCAAATATAATACAGGACAGTTTTATGACC-CCCTTTTT

CCCGCCAAAAAATTCAAATTTCGCGCCATTTTTAACGGTCGGGGGCCGGAAGTCCCACCC

CCGAAAGGGCGAAGGTCGGGACAGCGTG--------GAGGGCGCCATTTTGGGGGCGGGC

AGATGATCTCA------CCCACCGCGGACTTTGAG---CGGGAGTTTTTGACCATAAAGT

GGTGAAT-TGGCCTCTTATCGGTGAACTT-TCGCCCCGAATAGTGTACACTTCAGC--AC

TACGTCAGCGGGTGG-ATTTTATTGACATTGGGTTAATTATTCCTCGAAAGGGGCCAGAG

TCC-GCGCCAGCGTGGAGAAAGGGGAGGAGAGAATGGGGTATATAGTCAGTTCTCT--CT

CCCCGAGAGGCC-----ACTCTTGGGTGA-GTTCGA-GAAGGGGTTTCTCTGCCGCTCCG

TGCCGCGTCGCTGACCCTCCCCCTACCTGTCAAGATGAAGAAGATCGAGC--TGGTGCTG

GATGACAGCATTCTCAACCTGGCTGACGTCCTTTTGGGGCAAGACCACCCG------GAG

GAGGAGCCTCAGGAGCTGTCAATCTTCAGTGGTGAGGACCCCC-CGACCCTCCACGACCT

TTTTGATGTGGTCCTGG---GCGGAGACCA-CCCCGATTACTCTCCCGATGTGAACCTGA

TTTTTCCCACCGCGGAGCTGGAACAGGCTGAGGCTGAAAATGAAGAAGAAGTTCGCTGCG

AGACACCTGTTGTGCAGGTAGATCTGGAATGCAATGAGAGTATGATT----TCTACTACT

CCTGAGGGGTCGGTGGGCTCTCCGGAAAGGGAGGAGCCAGAAGAGGGACCCTCTTCCCGT

TGGGGACCCCTGGAACCCGGAGAATCCCCCC------GTCCGTGCCGCGACTGTACTTAC

CACCGTGTGGAGAGCGGGGATCCATCTCT--GAAGTGTGGCTTGTGCTACATGAAGGATA

CTTACTACCAGGTTTACAGTAAGT---AGAGATTTTAAATGTCTTTATGTCTTCTTTGTG

GTTGAGA-GAAAGTTCTGTGTTTC-TAAGTGGTTTTTCTATGA--ATTTTCTCAGGTCCT

GTTTCTCCTGCCACTCCGCTGAC----CACAGAGGAGGATGTGGTTAATGATCAACCAGA

-----GGAGGTGGTGA-----CCTCTGCTCCTTCTAGCCCGGTTGCCGAGGAAACCGGGC

GCGGTGCCAGGCGTCGCCCTGCTGAGGCAGCTGAACCTTTGGACCTCAGTTTGCCTAAGA

GGCGTCGTCAGCAGTAATTGCCCTGCATGTCACCCCGTGACTCATACCATACCAGTGTTA

TAAAAAGACAGTCTTAGAGACTCAGAGCACAGAG--------TTCTTGGAAACTCTTGGA

AGAACATCAGCTTCTCTGGGACTTACAAAC----AATTCTCTGGGACTGCTCAGATGGAC

TACCTCAAGTTCTT-AACCAACTATGCTGTGCTGAAGAGGATTATTCGGGACTCTAG---

---TGAGAGGACTGGACCTTGGAGGCGCTA---CTTCTTTTGCGGGCC-TTTGGCTAATT

TTAT-----------TCACAAGATCAAGGAAGAAAATAAGGAGCTTTTTGCTCAGTGCTT

GGATTCAGAGGGACAATTCTTGGAGCTTTTGCGCAGCGGGGATCTTGCTCTGTACAAC-A

C--TAAGGTTGTTCCTGCTTTACAATTTGAGAATCCGGGGCGGGTGGCTAGTAGCTTGGC

TT-TTGTGGTGTGGTTGCTTG-ATCAGTGGGATCTTCAGAATCAATTTTC---TCCTGAG

TTTTCTTTGGAAGCTCTCTGTATTCCAGTATGGAAGATAATCAAACAGGAGGTAGCTCAG

AGGGAACTGGGGACAGCGGCTCGGGTTTTCCAACAGGAGCTGGAGCGGGAGGGTCTGATA

GATCCCCCGGGATCCATGGTGGGGGAGACCCCGGAGAGGGAGGACACGGAGGAAGGGGAG

TTGCATCTAGCGGAGCCAGCAGTGATTCAGGAGACGGAGAGCGAGGGGGAAGAGGAGGAG

ATGCCTCCCCTGGAGGAGAGCGAGAGCGAGGAGGAGAGCATGGGAGAGGAGGAATTGGAG

GAAGTGGAGACAGAGGAGGACAGCGAGTTGGAAGTAGTTTTTGCTGCCGAGAACGAGGTG

CTGAGTCCGACCCCGG---------ACCTGTTGAGGCAGGCTGCAGTTTCTGGCCTGAGC

AGCAGCAGCGAGGAGCAGTGTTTCAGCCCATTGAGCGGGGTGGCTCCAC--TGGCCT---

----------CTTCGGACA-GCTCTTGTTCAGAGCCAGAA-GAAGAAGAGAGGGTCGTGC

CTCCGAGCGGGGGGCGCGCCATCCGAGTCAGCGTGATCGTTCA---CGCTCCCGCTCAAG

GGAC--CGGAGGGGCTCAGGGACAGAGCTCGTCAGGTGGGAGGATGT--AATGCAGCAGT

ATGCTCAGGGTGATGCCATTTTTAGTGAACAATATAGTTTTGAGCAAATAATTACTTATC

CA---ATGAAACCTGAAGATGATTGGAATGCAATGTTTAAGAGACATGCTAAAGTGAGTC

TGAATGCTAATAAAGTGTATACTTTGAAAAATAAAATTGATTGTGAGGGTCCTGTGTATG

TGATTGGGAATGGGGCTAAGGTAATAGTGGAGGG---TGAGTTAGATTGTGTGATTCAGG

TTCACCCTAAG---AACCCGGGACCCTCAGTGACTAACATGTGGGGGGTGGT-CTTCACC

AATGTGCAGTT-------------TGAGAGGGGAACTAATTTTACAGGGACCCTCGCTAG

GTGTCACTCTTTCACTGTGTTTCATGGCTGTAGCTTTTCTGGCTTTGTAAGTAC-TGTTC

T--GCATTTGCTGGCGGGT------------GGAGAAGTCAAGGGCTGCTGTTTCATGGC

CAATTATAGATGTGTGCTGAAT---GAGAGTAGGAATGTAGTGCTGGTGAAGAGCTGCAC

TTT-TGATAAATGCATACTTGGAGTCATCGGCCGGGGCCCCTGCAATGTCATCTACACTG

CCTTCAGAGAAACCTACTGCG---CAGCCCTCTTCCAAGTTCATGGACGCTTTAGATTCA

ATACTGTGGTGGACCCCACCGCCCTGGCCGACCGGTCCAACTTGCTTATGGGCACCTGTG

CCGAAGGGAACATGACTGC------GCTGTGTGCGGTGCACATCGTGGGG---AACTTTG

CTTCTCGCTTTGTGGAGATGAGCCACTGCCAGTTTTTGAGAGCTGATGTGTTT-GTGGGT

TGCCGCAGCGGGGTCTGGACATGCC---CCCAGAGCAGCTTCAA-CTTCTCGCGGATCTA

TGTGACTGCTGAGAGCCAGAGCAGG---ATCAGT--ATGACCGGGATTTACACTTGTTCC

CTGAGGATCATGAAGATGTA---TCGCCC---CGACATTGAGTCTTACCGGGGC-CGCCT

GTGTGAGTGTGGAGCCATGCATGGCTACTATCCTATGATGCTGGTGGATGTGACCCAGGA

TCTGCAGCTGAACCCCTGCCTGCA--TTCGGTGGACAGCTTGGATTACAGCAGTGATGAG

GAGGATCAGGTGGGTCTGGGGTCCGTGTGGGCGTGTCTGGGGGTGTGGTCACGTGAGAGT

ATATAAGGGTCTGACGGGTG--GTCTTTTGGTATGTTTCAGTGATCCCCTCGCGCTGAGA

AGATGGCTACTTCTACCAACAAGGAGAAGGGCACCATCAACACTGCATTTCTGACCTGCA

AGCTACCTGCCTGGGCCGGAGTGAGACAAGACATCGTGGGCTCTGACATCC--ACGGGAA

CCCGGTGAGGCCCACCAACTTCACGACC---ACCTCGACCAGCAGATACGTGGCCCCCGC

GGCGACCACCGCTTCGGAGACCACC-CTGCCACCCCCCGTGGCTGCTTCTGCCTATTCC-

---------ACCCACCATGTGGACCTGGTGCAGAGCACTGCCGCCACCCTCCTGCAGCTC

ACCAGC-AAGATCGACACCCTGGCTGAAAAAGTGTCCCAGCTGGAGCAGAC--CCTGGCA

GTGGTGTATGCGTCTTTGGCAGAGCCTGAAGCGATGGATGAAGATGA-------------

-----------AGAAGTCTAAAGCCCCCACTGATTATGAATC-ATTCAA----TCACATT

CTGATTGATTAAATTTCAATAAAATTTATT--------TTCTTTTGGCATGATAATAACG

AGTCCAGCGTTGTCTGTCAGTAATGATTCTGTGGATTTTTTCCAAGATTTGGTAGAGGAG

GGTCTGGACATTGAGGTACATGGGCATGAGACCCTCTTGGGGGTGGA-GGTACATCCATT

GCATACTATCATTCTCGGGGCAGGTGTTGTAGATGATCCAGTCATAGTGGGAGTTTTGGG

AGTAGTAGTTAAAGATATCTTTCAAGAGGAGGGAGATGGCAAGGGGGAGACCCTTGGTGT

AAGTGTTAACAAAGCGGGTGAGCTGGGTGGGTTGCATGCGGGGGCTGATGAGGTGGAGTT

TGGCCTGGATCTTGAGGTTGGAGATGTTACCTCCCTGGTCCTTGCGGGGGTTCATATTAT

GCAAGACCACCATGACACAGTAGCCAGTACACTTGGGGAACTTGTCATGCAACTTTGAGG

GGAAGGCATGGAAGAATTTAGCCACCCCCTTGTGTCTGCCCAGCTCTTCCATGCACTCAT

CCATGATAATGGCAATGGGCCCCTTGGAGGCGGCTTGGGCAAAG-ACATTTTGGGGGTGG

GTGACATCATAATTATAGTCTTGCAAGAGCTCATCATAGGACATTTTTACAAATTTGGGC

ATGAGGGTTCCGCTCTGGGGGACGATGGTGTCATCGGGCCCGGGTTGGTAGTTTCCCTCA

CAGATCTGGGTCTCCCAAGACATGATCTCTTGAGGGGGGATCATGTCCACCTGGGGGGCG

ATAAAGAAGACGGTCTCGGGGGCTGGTGTGATGAGGTGGGCAGACATGAGGTTGCGAAGC

AACTGGGACTTGCCCGAGCCGGTGGGCCCGTAGATGACCCCGATGACGGGCTGGGTGTTA

TAATTGAGGGATTTGCAAGTACCCGCATCGGTGAGA------------------AAGGGG

GCTGCGCTGTTGAGGGTCTCACGAATCTTTTTATTTTCACTGACTAAATCATGCAAG---

-----------AGGTTGTCCCCACCCAGGGA-----------------------------

GAGGAGTTCTTGGAGGG-AGGAGAAGCATTTGAGGGGCTTGAGCC----CCTCGGAGAA-

----AGGCATCTTTTTG-----AGGGATTCATGCAAGACCTGGAGGCGTT-------CCC

AGAGTTCGGTTACATGT----CCCAGGGCAATTGGATCC----------AGCAGACCTCG

CGATTTCTTG--------GGTTGGGGGCGCTGTTGCTGTAGGGGA--CCAGGCGATGGTG

GTCGTC------------------GAGCAGGTTCATGGTCTGATCCTTCCAGGGGCGCAG

GGTCCTCTTCAGGGTGGTCTCCGTCACGGTGAAGGGTTGCACGTGACTTTGCGCGCTTGC

GAGGGTGCGCTTGAGACTCATCCGGCTGGTGCTGAAGCGGTC------------GCTTCC

CTGCTGAGCATCGGCATA---GTAGCAAGATTTGAGCAGG---TCGTAGGAGAGGGTGGT

GGTGGCGTGGCCCTTGGCGCGCAGCTTGCCCTTGCCCACC------TTCTTGCAAACGGT

ACAGCAGGTATCCTTGAGGGCATAGAGCTTGGGGGCGA-GGAAGACGGACTCGGAGGCGT

AGGCGTCGGCCCCACAGAAGGAGCATTGGGTCTCGCACTCGACGAGCCAGGTGAGGTCGG

GGTGGTCGGGGTCAAAGACCAGGCGACCGCCATTTTTTTTGATGCGGTGCTTACCTTGCG

TCTCCATGAGGTAGCGTCCGCGCTCGGTGACGAAGAGGCTGTCGGTGTCTCCGTAGACAG

ATTTGAGGGCGCGCTGGGCGAGTG---GCACGCCGCGGTCCTCCTCGTACAAGAATCCGG

CCCACTCTGAGACGAAGGCCCGCGTCCAGGCCAAGACAAAGGAGGCGATCTGCGAGGGGT

AGCGGTCATTGGTGATGAGGGGCGAGTTTTTCTCGAGGGTATGCAAGCAGTAGTCCCCGT

CCTCGACATCCAGGAAGGTGATTGGCTTGTA---GGTGTAGGTCACGTGACCC-GAGTCT

GGGGTCTCCCCGGCCGGGGGGGTATAAAAGGGGGCGCGGCCCCGGGGGTCCCCACAGTCC

GAGAGTTGGCTTTCGTCCGCGAGCTC-----------GCGTGGCTGAT------------

------------------------------------------------------TTGGCT

CGGGTGAGTAAATGATTTTGAA---TTCGGGTAGAATTTCCGCGCTAAAGTTGT------

------------------CTACTTC----------TATGTACGAGGAAGAT--------T

TGACCGTGTATTCACCGGTGGCGATGCCTTTGAGGTACTTGTCTTCCATCTGGTCGGCGA

AGACGGTGGTCTTGTTGTCCAGCTTGGTGGCGAAGGAGCCGTAGAGGGCATTGGAGAGGA

GCTTGGCAATGCTGCGGATGGTCTGGTTCTTGTCCCGGTCCGCCTGTTCCTTGGCGCGGA

TGTTGAGCTGGACATACTCGCGGGCCACGCAGCGCCACTCGGGGAACACGGTGGTGCGCT

CATCGGGGAGCAGGGTGACGCGCCAGCCGCGATTATGCAGGGTGATGACGTCGATGGAGG

TGACAATCTCTCCACGCAGGCGCTCGTTGGTCCAAGCCAGACGCCCACCTTTGCGAGAGC

AGAAAGGGGGGAGGACATCCAGGTAGTCTTCGGGGGGCGGGTCGGCATCGATGGTGAAGA

TGCCGGGTAGGAGG-GTGGTGTTAAAGTAGGAGATG---GAGGCGGGGTCTTGCAAGCGC

TGCTCC---CAGGCGCGCATGGCGAGGGCCCGATCGAAGGGGTGCAGGGGGCGCCCGGCG

GGGAAGGGGTGGGTGAGGGCGCTGGCATACATGCCACAGATGTCGTAGACGTAGATGGGT

TCCTCCAGGACCCCCAGGTAAGAGGGGTAACAGCGCCCCCCGCGGATGCTGGCGCGCACG

TAGTCGTAGAGCTCCATGGAGGGTGCGAGCAGG------ACGCTGCCGAGGTTGGCGCGG

TTGGGTCGCTCGGCCCGGT-----------------------------------------

----------------------------AGACCACCTGGCGGAAGATGGCGTGGGAGTTG

GAGGAGATGGTGGGGCGTTGGAAGATGTTGAAGCGAGCCTGGGGCAGGCCGACGGACTCG

GCGACGAAGGTGCGGTAGGAGTCTTGCAACTTCTGGACGAGTTGGGCGGTGACGACCACG

TCCAGGGCGCAGTACTCGAGGGTGTGTTGCACGATGTCGTACTCA---------------

---------------------GTCTCGCCTTTTTTCTTCCAGATCTCCTTGTTGAAGGAA

TACTCCTGCTCATCTTTCCAATAGTGCGGAGCCGGGAACCCGTC---TCCATCTTTC---

AGGTAAGAGCCAAACATGTAAAACTGGTTGACGGCCTGGTAGGGACAGCAGCCCTTCTCG

ACCTGCAGGGAGTAGGCCTGCGCGGCGTTGCGCAGGGAGGT---GTGCGTGAGGGCGAAG

GTGTCGCGCACCAGAAACTTGAC-------------G-TATTGGAACTTGAAGT--CGCT

GGTCTCGCAGGCGCCCC--TTTCCCAGAGCTCAAAATCCCCCGGCACGCGCTTGCGGTAG

TCG---GGGTTGGGTAGGGAGAAGGTGATGTCATTGAAGAGGATCTTGCCGTTGCGCGGC

ATGAAGTTGCGGGAGATGC---GGAAGCAAGAGGGGATGTCGGTGCGGTTGC-------T

GATGACCTGGGCGGCCAGCACGATCTCGTCGAAGCCGTTGATGTTGTGGCCCACGACGTA

GACCTCGATGA--AGCGGGGCTGACCGCGGAGAC--------GCAGAGCTT---CCATCT

GTTGGGGACTGAGCTCCTCGGGCGAGCG-GATGGCATTGAGCTGGCAG---AACTCGGCC

AGTTGGGGGTTCTGCTTGGTGACAAAGTTGAGGAGGAGACGCGCG---GTGCGTTTTTGC

AAAGAGTGGCGGAAGGTCTTAAACTTGCTGCCGATGCTGCTCTTCTGGGGGTTGA---GG

TAGTAGAAGGTGTGCTCGGCCCCGTCCCA---GACCTGCCACTCCTGACGGTGGGCGAGC

TCCTCGGCGAGGCGCACGAGCTCCTCGGTGCCCGAGAAATGCATGACCAGCATGAAGGGC

ACGAGCTGCTTGCCGTGCTGCCCGTGCCAGGTGTAGGTCTCGACATCGTAGGTGATGAAG

AGGCGTTCGGTGGCGGGGTTGGATCCGATGGGGAAGAAGGAGATGGGCTCCCACCAGGCG

GCGGAGCGGGTCTGGATCTGGTGAAAGTAGAAGTCGCGACGACGCACGTTGCAGGTGTGG

GCGGCCTTGTAGAAAGAGCCGCAGTGGTCGCACTTGGTGACGCGGTGGATCTCCTTGATG

-AGGCCGACGACGGGCTTAC-----CGTTGCTGTTTTTGACCAGGAA-------------

------------------------GCGGATCGGGAAGGGTA-GCGAGGATTCGGGGTAA-

--GAG-------------ACGGAGGTGACTTGAAAGAAGCCTCGAGCGTGAGTATAGATG

TCCCCCTGCGGCGGTTGGATGTTCTCCAAGGTGGCGAGGAGCCGGTCTCGTCGGAGCGCC

TCGGGA------CGCAGCAAGTCTTCGGGCGGATCGGAGCAAAGATGCACGAGGAAGAGG

TTGCGCAGCCCCTTGACGGGGTCGAGCGCGTAGCTGACTTCCAAGGCCTCGCCCTCGGC-

--GTTGGTGCCGTAGACTCGCTGCGTGTGTCGGCGGTTGACGATGGTCCC----------

------------------------------------------------------------

CTTCAG--------------TGGGGGGCGGCCCCGACGGGGGGCAGCGCCACCA---CCT

GGTTGAGTTCCGGCAGGGGCTGGCGTTGACGTTGGAGCTGCGTGGCCAGCTGGACGACCC

GGCGGTTGATGTTCTGGATCTCGCGCTTCTGGGAGAAGACCACCGGGCCGATGACGCGGA

ACCTGAAAGAGATTTCCACAGAATCAATCTGGGCATCGTTCA-TGGCGACCTGGCGCAGG

ATCTCCTCGACGTCGCCCGAGTTGTCGTGGAAGGCGATGTCTTGCATGAAGCGCTCGGCC

TCGTCCTC-----GTCGAGCTCG-CCTTGTCCGGCGCGCTCGACGGTGGCGGCGAGGTCG

CGGCTGATGCGGCGCATGAGCGCGGGGAA------GGCCTGGGTGCCGTTCTCGTTCCAG

ACGCGGTTGTAGATGTTGCGTCCTTCGGCGTCGCGGGCCCGCATGATGACCTGCGCGAAG

GGGATGTCGAGGTGTCGAGCGAAGGGCGCGTAGAGGCGCAGGTTGTGATGCAAGTAGTTG

AGGGTGGTGGCGATGTGCTCGGCGACGAAAAAGTACATGACCCAGCGGCGCAGGGTGAGT

TCGTTGATGTCCCCGAGGGTCTCGAGTCGAAGCATGGCCTGGTAAAAGTCGATGGCGAAG

TTGAAAAAGTGGTGGTCGCGCGCGGCGACGGTGAGTTCCTCTTCCAAGAGTCGGATGGCG

CCGGCCACGGCGGCGCGCACCTCCTCCTCGAAGGAGGGCGGTGGGGCGGGGGGTCCGGCT

GCTTCCTCTTCCTCCTCGAAGCCCTCAGGTGCGGCGGCGAACGGTTCTTCCTCCTCCTCT

TCGACGTCGGAGACGGGCGCCGGTGCGGCGACGCGGCGTCGGCGTCGACGGATGGGCAGG

CGGTCGACGAAGCGTTCGATGATCTCGCCGCGGCGTCGACGCATCTC---CTCGGTGACG

GCGCGTCCGTTC---TCGCGGGGACGCAGCTCGA----AGGCCCCTCCGCGCAAGAAGCT

CTCG---------GT-GGCCTGACTGGGTA-----AGCTGAGGGCGGAGACCACGC-ATT

GGAGTAGC-TGAC-GGTTGTGATTTGTTCCCTCTCCCCACAGGCATCGCCGTAGCGAGT-

---CTGAATCTACCGGATCGCTGAAGCGTT--CGACGAAGGCTTCGAGCCACAGAGCATC

ACAAGGTAAG---CTGAGTTTGGTCTGTTG------------------------------

------------------------------------------GTCTCGGGAGAGGAGGTA

GTTAAAGTAGGCGGTCTTGAGACGCCGGATGGCGGTGAGGACGACGAGGTCCTTGCGACC

GGCTCGCTGGATGCGGATGCGGT-CGGCCATCCCCCAGGC-GCGCTCCTG-ACAGCGTCC

CATGTCGCGG--TTCTGCTCTTGCAACATCTGCTCGACGGGCACGTT-------------

------------------------------------------------------------

--TTGGTCGGCATCCATGGAGGTGCGTCCGTAGCCTCGCAGGGGCCGGAGGAGGGCGAGG

TCGGCGACGACGCGCTCGGCCAGGATGGCCTGCTGCACCTGCGCGAGCGTCTCCTCGAAG

TT------------GTCGAGGTCGATGAAGCGGTGGTAGGTGCCGGT-GTTGATGGTGTA

AGAGCAGGTGGCCGTGGTACTCCAGAAGACGTA--GTGCTGGCTGGCGCGCACGGTATCG

TGG----TAGCGGAGGCGGCTGTAGGAGCGGGTGTCGAAG-ATGTAATCGTTGCAGGTGC

GGACGAGGTACTGGTA-TCCGACGAGCAGGTGAGGCGGGGGGTAGAGGTAGAGGGGCCAG

TTGAGCGTGGCGGGCGAGCGGGGCGAGAGGTTGGCGAGCATGAGGCGGTGGTAGCGG-TA

AATGTACTTGGACATCCAAGCAAGTCC-TCCGGCGGAGACGGAGGCAGGCACCCAGTCCC

GGACCCGGTTCCAGAGGTTGCGCAGGGGTTGAAAGACCTCGATGGTGTAGACGGTCTGGC

CGGTGAGGCGCGCGTAATCCATGGCGTTCTGCGTACG-----------------------

------------------------------------------------------------

------------------------------------------------------------

------------------------------------------------------------

------------------------------------------------------------

------------------------------------------------------------

------------------------------------------------------------

-------------------------ACAAGCAAAGAGACC--------------------

--------------------------------------------CCCTGTCTTACCCG--

-----------GCAG-ATGCATCCCGTGTTGCGACAGATGAAACCCG-------------

-----------------------------CGTCGGCTC----------------------

------CGAGCCCGGCGGCGACGGAGGCGGTGGGCCGCGG--GCTGGCCGCGGCGGC---

---GGCGGCGCTGCCCGAGCCGCCCGAGGAGGGGGAAGGCCTGG-CCCGGGT---GGTGA

GCGGGGGTCCCGAGC--GACACCCGCGCGTGCAGGTGAAGCGCGACGCGAGCGAGGCGTA

CGTGCCTCGGCAGAACGTGCTGCGCGA---------CCACCCGGGCACGGAGGGAGAGGA

GATGCGCGACCTGCGTTACCGCGCGGGGCGCCACATGCG------CGTGCCCAAAAACC-

GGGTGCTGCGCGACGTGGACTT--CGAGGCGGAACCGCTGACGGGCA------TCAGCCC

GGCGCGGGCCCACCTCGAGGCCGCCAACCTGGCCACGGCCTACGAGCAGACGGCGCGCGA

GGAAGCCAACTTTCAAAAGACCTTCAACAATAACGTGCGCACGCTGGTGGCGCGCGAGGA

GGTGACCATCGGTCTGATGCACCTGTGGGACTTTACGGAGGCGTACGTGAGCAACCCGAG

C-------AGCAAGACGCTGACGGCGCAGCTGTTCCTGATCGCCCAGCACTCGCG-CGAC

GAGGGGGT---GTTCAAGGAGA--GCCTGCTGACGTTGGCGGAGCCC----GAGAGTCAG

TGGCTGGTGGACCTGATTAACATCCTGCAGTCCATCATCGTGCAAGAGCGGGGGATGCGG

GTGCCCGAGAAGGTGGCGGCCATCAACTACAGCCTGATCAGCCTGGCGAAGCACTACGCG

CGCCAGCTGTTTAAGACGGCGTACGTGCCGCTGGACAAGGAGGTGAAGATCCAGACGTTT

TACATGCGCACGGTGATCAAGCTGCTGG------CGCTGAGCGACGACCTGGGCATCTAC

CGCAACGAGCG--CATCCAGCGGGTGGTGAGCGCGTCGCGCCGC-----CGCGAGCTGAG

CGACAGGGAGCTGATGTGGAACCTGCGGCGCAC----GTTGGCGTCGAGCGGGGCCGACG

CGGAGCAGGCGCGCGCCTTGGAGGCGGGCGACGACCTGCGTTGGGTGCATCCGCAGCAG-

CAGCAGCAGGGGCTGCGGGCGGTGCCCGAG-----------------------------G

AGATGGAAGAGGAGTACAGCGAGGATGACGAAGAGATGGACGACTATTGA--TGAGTGCT

CCCCCTGC-------------TCTTCCTCCCTCACCATCAGATGACTAGCCCTAATGTGA

ACAA-GATTAACCCGGTGGCCCTGGCCGCGGCCCAGTCCCAACCCACGGCGGAT------

---GCCGGTTGGAGCGCCGACATCCGGCGCATCTTTGATCTGACGATCAATCGCCC--CG

GT-TTTAAGAACCAGCCGCGGGCGAAT-CGGTTCGACGCCATCTTGGAAGCGGTGGTGCC

GAGCCGACAGGACCCCACCCACGAGAAGGTGCTGGCCGTGGTGAACGCGCTGGTGCAGGC

TCACGCGGTGCGCGCCGACGAGGCGGGGCAGTTGTACAATGCGCTGTTGCATCGCGTGTC

CCGCTATAACAGTATGAACGTGCAGACTAATTTGGAGCGGTTGGTGACGGACGTGA----

--AGGATGCGCTGGGGCAGCGGGAGCGCAACATCCGCAGTAATAATCTGGGGTCGCTGGT

GGCCCTGAATGGTTTTCTGGCGACCTTGCCTTCGGTGGTGGAGCGAGGGCAGGATAGTTA

CGTGAGCTTCGTGAGTGCCCTGAAGCTCATGGTGAGCGAGGTGCCTCA---GAGCGAGGT

GTATCAGAGCGGTCCGGATTTCTACTTCCAGACCACGCG-GA--GCGGGTCTCAGACGGT

GAACCTGACCAAGGCTTTTGAGAACCTGCGCCAGCTGTGGGGCGT-CAAGGCCCAGCAGG

CGTCGGGCAGTGCCATCTCTAGCTTG-----TTGACCCCCAATACGAGGTTGCTCTTGCT

GCTGATC-GCCCCGTTCA-CGGATAGCGTAGGCATCCCGCGGAGCACTTACATCGGTCAC

CTGCTGACCCTGTATCGCGAGACGCTGGGCCAGACGCAGGTG---GCCGAGAGCACGTAC

CAGGAGATCACGCAGGTGAGCCGCGCCTTTGGCGAGGAGAATGCCG---ACAACCTGCAG

GCCACTCTGAATTTCTTGCTGACCAACCGTCAGCAGAAGCTACCTACTCACTACGTGTTG

ACCGAAGAAGAGGAGAGGGTGTTGCGCTACGTGCAGCAGAGCGT-GGCCCTGTTCTTGAT

GCAAGATGGGGCGACCC--CGTCTACGGCCCTGGATAGGACGGCTGCCAATTTCGAGCCG

ACCATGTACGCCGCTAACCGAGCTTTCATCAACCGTCTGATGGACTACTTGCATCGGGCG

GCCGCCGCCAGCCCCAATTACTTTACCAACGTGATCCTGAACGCCAACTGGCAACCGCCT

CAGGGCTTTTTCACGGGCGACTTCGAGATGCCCGAACCCGAGGAGGAGGCTTACGCCTGG

GACGATATCAACAGTTCCCTGGCTAGCATGTCGGC-------------TCAGCGTCAGCT

GCGAGATAAGA---TCACCGCGAGCC--------TGCCCTCGCTGC----------CGTC

CTCGCGCCCCAGCACCCCCCTGTCCGCCAGCCGCCGAGGTAGC--------CTGAGCGAC

C----------TGGGTGCGGCCGCCCCCCGCGAG-TTAGATCCGGGCTCGGCCGGCCTCA

ACCTGGAGCGCGAGCTGGAGAGCATGTTTCAAGACAAGGAT------------------A

ACAACCTGGTGGATAGCCTGACGGATAAGATGGCG-CGCTGGAAAACCTACCGCAATACT

CAATCCGAGCTAGAGC---------------GGCTGAG--------TAACGTGAACCTGC

GTCACC-CCGCTTCCGGAGAGCGACGGGAAAACGCGGACGACGG----TCCCCGGAGCCT

GC--GCGACCGCGACCTGTCGGGTCACGGTCTGCGCCGCTTCGCCCCCAGCAACCCCTTC

GGCCACCTGATGCCCCAGGGTGGCCGGAGACGCTTGTAGTGA--TTTAGGGCTGCTAAAA

---------------AATAAAAAAATCCACAGGGATGCT-TACCAAAGCCATGTCG----

-TGTTTTTATTGCAAGACGAGCCGCGATGCAGAG---AAGCGTGCCGCTGTCCCCTCCTC

CTCCGTACGAGAGTCCGCCTCCCAGTTACGAGAGCGTGA---------------------

----------TGCAG-----------CCGATCCTG-GAGCCTTTCGTGCCTCCGCGCTAT

CACGCA-CCGTCTGAGGGGAGAAATAGCATAAAGTACAGTCAGCTGCCCCCGTTGTACGA

CACGACGCGCCTGTATCTGATCGACAACAAGTCGGCGGATATCTCCACGCTGAACTACCA

AAACAACCACAGTAATTACCTGACGAGCGTGGTGCAGAACAGCGACTATACGCCTCTGGA

GGCGAGCACGCAGAATATACTGTTGGACGACCGTTCTCGGTGGGGCGGGGACCTGAAGAC

TAT--TTTGCATACGAACCTGGTTAACATCACGGAGTTCATGTTCAGCAATAGCTTCAGC

GTGAGGGTGATGAC------TAAGCGGACAGATAA-------------------------

-----------GGGCCCCGAGTACGATTGGTTCACCTTGCAAATCCCCGAGGGGAACTAT

TCCGTGCTCACCACCATCGACATGATGAATAACGCCATCATCGAGAATTACTTGCAAGTG

GGGCGTCAGAACGGGGTGAGGGAGGAGGACATCGGGGTAAAGTTCGACACGCGCAACTTT

CGGTTGGGCTTTGACCCCGTGGCCAACCTGGTGATGCCGGGGAAGTATACCTACGAGGGC

TTTCACCCGGACATCGTGCTGCTGCCCGGTTGCGCGGTGGACTTTACTTATTCCCGCTTA

AATAACCTGCTGGGCATTCGTAAGCGGTGGCCCTATCAGGAAGGGTTCATCATCAGCTAC

GATGACCTGCTGGGCGGCAACATTCCCGGGCTCTTGGATGTGGAGGCCT-----------

----ATGAGAATAG------------------TCTGAACAACAGCAGCCCGGCCGATGA-

------------------GCCGCCCCGG--------------------GGGGATAC----

-----------TTATGCCACCCCCACACCCGAGCCT---GTGATTCAACCT---------

-------------------------------------------CTGAAGA----------

-------------AAGATGCGGGTAACCGCAGCTATCACGTGGGCGAA---GATCCCGAG

GCTCCAAATGCCAATTTCACCGCTTATCGCAGCTGGTACCTGTCTTACAACTACGGTAAC

CCCGACAAG---GGCGCGCGCGCCACCACCCTGCTCTGCGCCTCGGATGTAACTTGTGGG

GTGGAGCAGGTGTACTGGAGCATGCCGGACCTGGCCGTGGAGCCGGTGACTTTCAAGGCC

TCGC---AGGACCCCAACAACTACCCGGTGGTGGGCACGGAACTGTTACCCCTGATGTCC

CGAACTTTTTACAATGATCAGGCTGTGTATTCCCAGATGATTCGCGAGAGTAC-TAACCA

--GACGCACGTATTTAACCGATTCCCCAACAACCAGATTTTGGTGCGACCGCC-GGCACT

CACGATTACTTGCGTGAGCGAAAACGTGCCGACTCTGACAGACCACGGAGTGGTACCCCT

CAAGAACAGCCTGTCCGGTGTGCAGCGCGTGACGCTGACCGACGCTCGCCGACGCGTGTG

TCCCTACGTGTATAAGAGTCTGGGCGTGGTCACACCGCGAGTGCTGTCTAGCAAGACTTT

TTAGC-------------------------------------C-----------------

---------ATGTCTATCCTCATCTCACCTAGCAACA-ACTCCGGTTGGGGGTT-GGGCG

TG--AGTAAGA---------TGTACGGAGGTGCCAAGGTGCGCTCCGAT--AGCCACCCC

GTGTTGGTGCGTCAGCATTATCGCGCTCCCTGGGGGATCCACAAGGGCCG-----CCGCC

GCGGGGGGACCGATAGCATCAGCCGGT-CGATCGAGGCCGTGGTGCGACGGGCCCGCCGA

TACAAGGGCCGCCGCGGTCGCCGCTCTG-----CCGCCTCTGACCCCGTGAGCGCCACCA

TCGAAAGCGTGGTGCGTAGCGCGCGTCGTTACAAGGGACGCAAAGGGAGGCGCGTGGTCG

CTTCCCCAGATACTTATTCCGACGCCATCGAGAGCGTGGTGTCCGCCGCCCGGAACTACC

AGCCGGCGGCCACGCCTATGGATACGGTGTCCGACGCCATCGAAAGCGTGGTGTCGGCGG

CCCGGGGTTACAAGCGTAAGCGCGACCCGGTGGCCGCCTCCATCGAGCGGGTGGTCAAGA

GAGCGCGGCGTTACCGCGACCCGGTGAGTCGGACGATCGATCGGGTGGTCCGACGCGCCG

GCCGTTACCGCGACCCCGTGAGCCGCACCATCGATAACGTGGTGCGCAAGGCGCGGCTGT

ACCGCCCGCGGCGCGGAGCCCTGCCCGGAGTGAGGGTGCTGAGGCGGACCATCAAGCAAG

CGGCACGACGATCCCGTCTGAGGGCGAGGGCCGCGGCCCGCCTGGCGGCGCGAAGAGGCC

GACGTGGCAATGTGTATCTGGTGCGAGATGCAAGTGGAAACCGTGTACCCGTGCGCGTTA

GACCTCCCCGTCAATAAAGCTTCCCGTTTTTGAACACTGCACAGCGCGCGTCCGAATCCT

TCTTCGCCGCCGTCATCATGGTTTCTCGAAAGATAAAAGAAG-AGATGC--TGGCCGTGG

TGGAGCCCGAGTTTTACCCCGATCTTAAACGCAAGCGGG------CCGTAAAGCGGGAGT

TTAAGGAGGAGCCCGACGTGGTTTACATTAAGAGCGAGGGTGGTGACGTGAAACCCTTCG

TGAAACGCGAAATCAAAAAGGGGCGCGTGAAAAAGGCTAAAAAGGATGAGGATTGGGGAG

ACGATGTAAAATTCGAGGGTCAGGTGATGAGC---AAGCGGCGACCTTATCAGTGGAAGG

GGCGCAGGGTAAAAAAGATCCTCCGCCCCGGGGTGCCGGTGGTCTTCACCCCCGGCCAGC

GC--AGCGGGGTG-GCTCTGAAGCGCAGCAACGAGGAGTTGTTTGCCGATGAGGATATCC

TGGAGCAGGCCGAAAAGCTGGAGGGCGAATTCGCCTACGGCAAGCATCT---GCG-----

-GATGTCCTATGACGAAAAACCGGCTTACGTGCCG---CTTTCGGTGGGAAACCCCACTC

CCAG---------------CTTGAAGCCGGTGACGGAGCAGAAGGTGGTGAAGATAGCTA

AGAAACGCGGGCGCAATGAATTCGATGAGCTGCAGCCGACGG--TGCAGAT-CATGGAAG

GCAAGCGCCGCCGCCCCAACGAACCCGAAGTGGGTTTCAGCGAGGTGCAGGATGTCAAAG

TCAGGCCCATCAAACAAGTGGCCGATACGGTGGCGGTG------GCGACGGTCGATGTGG

AGATGCCTCAGGCCGCGGCCGCTCAGATGATCGAAGCCATGGAAGCACAACCCTCGACCA

GCCGGGCCGCTAGTGCGGCCGCGCCCCT---ACCTACCATCACTCCGATGGAAACTCAGA

GCGGCACGGTGCGCGCCATGGCCGTTCCCAAAC--GCAAACACAAGAACCGCTACCCCG-

TGAGCAAAAATTACGGGAAGGCGAACAGTA-----TCATGCCGCAGGTGGTCTACCATCC

CAGTATTCGCCAAGGCCCATCGG--------CCGCGACGGTCACATCCCGGCGGCGGCGT

GGTACCTCCACCCGT-CGCAAGAGTACTACCCGGCGTCGCCGCGGTGCTAGTGGACGC-A

CCCGCGCGGCGGTGCGCACGCAGGCCGGTAGAGAATACTATCCTTCTAGCTCAGGGCGAC

----GCTATACCCGACGGGGGCTGCGTCTGCCCATGGTG-CGCTACCACCCGAGCATTGC

CCACACCAGCCAGTCCCGGGGCGTGATG--CCTACCGTGC--------GCTATCATCCTT

CCATTACA----CTGT------------------AAGTAACCGCTTGTGTAATC-CCCGT

CGGGCC-----------CTTCGTCTACAGCTCTTTTCCGCGCGAAGACCCACC---AAGC

ATCAACAAAAGTGATGGCTAAACGAATGGTGTGCCGCGTCC-GCGTTCCCA---TCCGC-

------ACCGCCAGCGTGCGTCGCCGCCGCCGCGTGCGTAGGCGACGAG--CCGGAACGA

TAGCCTCTTGCTATAGCGCCTGTCAGAGGCGGTTCGGCGTGACAGGCAAGC----GCAGT

C--GCCGTGGGCG-ACGGCGTCGCG-GGCGAAG----AATCCGCGGAGGCTTTTTGCCGG

CCCTGGTGCCCATCATCGCGGCGGCCATCGGAGCCATCCCCGGTATCGCGGGTGTAGCCA

TCCAGGCTTCTCAGAGACATTAAAGTTGTTGTG------TGTGAGCCCAAATTGATGAA-

--------------------GTCGGTGCTTCCTTTCCTTTTCGCC---------------

----------GCCAGCAACTCCCGATCACCACCACCA-----------------------

CCATGGACGCAGTCACATTTACCTCCCTGGCTCCGCGGATCGGCTCCCGCCCGATGATGA

GCGGGTGGTCAGAGATCGGCACGAGCGCCATGAGCGGCGGCGCGTTTAGCTGGGGCAGTG

TGTGGAGCGGGCT---------------GAAGAATTTTGGCAGCAATGTCAAAAACTATG

GCTCCAAAATTTGGAACAGCAGCACCGGCAAACAGCTGAGGGATAAGCTGAAGGAAACTA

AGTTTCAGGAGAAGGTGGTAGACGGTCTGGCTACCGGGATCCACGGTGCCATCGACATCG

CGCGCCAGAAGCTCGATAAAGAGCTGGAGCAGCGCTTGGATC---GCCCGATCCAGGTGG

AG------GTAGAAG-------------ACATTCAAGGCCCACCAGATGGCGAGGCTGGT

TTGCTA---------------ACTGAGAAGGAAAAGGAAGAGCTGGTGGCGGCTTCTAAA

AAGCCGGTGATAGTGGAATCACCCATCAAGAAGCGCCCTCGAGATGAAGATCTGGTGATG

ACTTTGGATGAGCCGCCCTCCTACGATGAGATCTTTGGT-------------AACAAAGC

-C-GGCAACGCTACCAC----------------CTACCCCATGACGCGCCCGGTTCACCC

GTTG-----GCGCGCCCCGTGCT-------------GCCGGCCGTGGATCC---------

--ACCGGCTTACGAAGCG-CCCACTCCCGCCCCGGCTCCGGTCGTCCCCGCCGTGG--CG

CAGCCGTCTC----------GCCCTCGACCATCTCGAGGC---AACTGGCAGAACACCCT

GAGTAGCATCACGGGCTTGGGAGTTCGCGCGATTAAACGCAGACGTTGTTGGTATTA--A

GAGTCATCATGGTGTGTGCTCCT-CGTTTCTT------TCTGT-----TCTCGTAGCCG-

------------------------------------------------------AAGATG

GCCACCCCTTCGATGCTGCCGCAATGGTCCTACATGCACATCGCCGGTCAGGATGCGTCA

GAGTACCTGTCGCCCGGCTTGGTGCAGTTCGCCCGGGCCAC-GGAAACGTACTTTGCCCT

GGGCAACAAGTTTAGGAACCCCACGGTGGCTCCCACTCACGATGTGACCACAGAACGTTC

TCAGCGACTGCAGTTGCGCTTCGTGCCCATCAACCAGGAGGACACGCAGTACACTTACAA

GACCCGCTTTCAGCTGGCCGTGGGTGACAACCGGGTGCTGGACATGGCCAGCACGTACTT

TGACATCCGCGGGATCATTGACCGCGGCCCCAGCTTCAAACCCTACAGCGGCACGGCTTA

CAACTGCATGGCTCCCAAGAGTGCTCCCAATAATACA--------------------CTT

TATAAAGCTGGTGAT---------------------------------------------

----AATGGTGAT------------------AAGGTGGCAGTTTTGGCTCAAGCTTCATA

CCCAGGTCAAATTATAGTTAATAATGG-CAATGCTTCTGTTCAAACTGGTGATAATCAG-

-----ACAGCTGATCCA-AAGTATGAACCAGAACCACAGATTGGTGAAAATC---TTTGG

GCAGC-AGGTTTTGCCGGACTACTTG---A--ACCAAAAACCGGT---TCGGG--TAGAG

CTTTAAAAACTGCAGTAGC---TGATTTACCTTGTTATGGTTCATATGCTCCACCAACAA

ACGAAAAGGGAGGACAAGCAAA---------------AACTGGCACT-------------

-GAC-------------GTAGAAATTAAAACGTTC---------------GTACAAGGAG

CTGCAGCTGCTCAAC------CAAATTTGCTTTTATATACTGAAGATGTAAATTTAATTG

CTCCGGATACACATATTACATTTGAAACACTTCCTGAAAGTGAAGA------TCAGCTGT

TTAGCCTTAATCAAACTGCGGGTCCAAATAGACCTAACTACATTGGCTTCAGAGACAATT

TTATTGGGCTAATGTATTACAACAGCAATGGCAATCTTGGTGTTTTGGCTGGGCAAGCTT

CCCAATTGAATGCTGTTGTTGATTTGCAAGACAGAAACACAGAACTGTCATATCAATTAA

TGCTTGACAGTTTAACTGACAGATCCAGATACTTTAGTGTTTGGAACCAGGCTGTAGACA

GTTACGATATTGATGTTAGAGTTATTGATAACGTAGGTGTGGAAGACGAGATGCCAAATT

ACTGTTTTCCAATACCAGGTTTCAGTAAGCCAGCTAAACAAGTTACAGCTGTAAAATATG

A---------AAACACG---------------TGGCAGACAGATG---ATACTTATGTAC

AAAGTCCGCTAA-TTGATGTGGGCAATCTGGATGCGATGGAAATCAACCTCACGGCCAAC

CTTTGGAGGGGTTTCCTGTATTCCAACGTAGCCCTCTACCTGCCAGACAAATACAAGTTC

-------ACCCCACCCAACATCAAGCTG--CCTAACAATACCAA---CACCT---ATGAT

TACATGAATGGCCGCATTCCCTTTAACGGGCTGGTTGATACTTATGTAAACATTGGGGCG

AGGTGGTCCCTGGACGCCA-TGGACAACGTCAATCCCTTCAACCACCATCGCAATGCCGG

TTTGCGCTATCGGTCTCAGTTGCTGGGTAATGGTCGCTATGCAGAGTTTCACATTCAGGT

GCCGCAGAAATTCTTTGCTATCAAGAATCTTCTGCTGCTGCCTGGAACCTACACTTACGA

GTGGTCTTTCAGGAAGGATGTGAACATGATTCTGCAGAGCTCTCTGGGTAACGACCTGCG

TGTCGATGGGGCTAATATTGTGATCAAGAGTGTGAACTTGTATGCCAGCTTTTTCCCTAT

GTCCCACAACACCGCTTCCACCCTGGAAGCCATGCTGCGTAACGATACCAATGATCAGTC

TTTCACCGACTACCTCAGCGCTGCCAACATGTTGTTCCCCATCCCCGCCAATGCCACTCA

GGTTCCCATTTCAATCCCTGCACGGAACTGGGCCGCCTTCAGAGGCTGGAGCTTTACCCG

CCTGAAGCAGCATGAGACCCCAGCTTTGGGTTCGCCCTTTGATCCCTACTTTACCTATTC

CGGCTCAATTCCCTATTTGGATGGAACTTTCTACCTGAATCATACTTTCAGGAGAGTTTC

CATTCAGTTTGATTCATCTGTGAGCTGGCCGGGTAATGACAGGCTGTTGAGTCCCAATGA

GTTTGAAATCAAGCGCGCTGTA------GATGGGGAGGGTTATAGCATTGCTCAGAGTAA

CATGACCAAAGATTGGTTCCTGATCCAGATGCTGGCGCATTATAATATTGGCTACCAGGG

ATACCATCTGCCCGATGCTTACAAGGACAGGCTGTATTCCTTCCTACATAATTTCATCCC

CATGACCCGCCAGGTAATTGACGAGAC----CAATGTCAACACCGGCTACCAG--GCCGT

TTCCATCCTCAACCAG-----------------------CAT-------AACAACAGCGG

GTTTGTTGGCTACATGAA---TCCAGTGGCCACCCGGGAG----GGGCACCCCTACCCGG

CTAACTGGCCCTACCCGCTCATCGGACCCAATGCCTAC---------GCCACCGTCACCC

AGCGC--AAGTTCCTCTGCGATCGGGTGCTCTGGCGCATACCCTTCTCCTCCAACTTCAT

GTCCATGGGCACGCTCACCGACCTGGGCCAGAACCTGCTGTACGCTAATGCCGCACACG-

CGCTCGACATGATCTTCGAAGTCGACCCCATTGACGAGCCCACCCTGCTCTATGTTCTCT

TCGAAGTCTTTGACGTCTGCCGCGTGCACCAGCCTCACCGGGGCGTTATCGAAACCGTCT

ACCTCCGAACCCCCTTCAGCGCGGGTAACGCCACCACATAAGCAG---------------

------------------------------------------------------------

---------------------------------------------------GATGGG---

-------------------------------------------CTCCAGCGAGGTCGAGC

TTAAGCGCATCGTCGCCGACCTGGGCGTCGGAGGCATGTTCCTCGGCACCTTCGACAAGC

GCTTCCCCGGCTTCGTCT-CGCCCCACAAATCGGCCTGTGCCATCGTGAACACGGCCGCG

CGCGAGACCGGAGGCGTGCACTGGCTCGCCCTCGCCTGGCATCCCCCCTCCCGGACCTTT

TATCTCTTCGACCCCTTCGGCTTCTCCGACTCCAAACTCAAACAGATGTATCAGTTTGAG

TACGAGGGACTCCTCAAGCGCAGTGCCCTGCAGTCCACCCCCGATCGCTGCCTCAACCTC

GTCACCAGCACCCAGTCCGTGCAGGGACCCAACAGCGCCGCCTGCGGGCTCTTTTGTTGC

ATGTTTCTCCACGCCTTCGCCAAGTGGCCCAACACCCCCATGGATCACAACCCCACCATG

GACCTCATCCATGGCGTGGCTAACTCGCAGCTCATGAGTCCCGG-GGCGCAGC-GCACCT

TCTATCAGAACCAACAGGTCCTCTACCGCTTCCTTCAGACCCACTCCCCCTATTTTCGTG

CCCACCGCGCGCAGATCGAGCGCGATACCCACTTTGATAAGGCGCG--------------

-----AGAAATGTCATGAAA-----CCACACCGTGTTGCTGTCAAACAATAAAAGTGCTT

TTATTTAA--------------AAGCTT-----------AAAACAGACACTCGTCGGCGT

CGTCACTCGCG-----------TGCGTCATG----------------GGCAGCAGGGTGT

TTTGGTACTGGTA-----CTCGGGGGACCAGCGGAACTCGGGGA-TGCAGACCGGGGC--

---CTGCTTCTCCATACAGCTCT---GCCAC-----ATCTGCTTGGCCAGTTGCAGTGCC

GTCATCACGTCGGGGGCGCTCAGCTTGAAGTCGCAGTTCTTCTGCGCACTCGCCTTGC--

-----TGTTGCGGTACAC------GGGG--TTGCAGCACTGGAAAACCAGGATGTTAGGG

TGCTCCAGCGTCGC---CAGCAGCTTCGGGTCTTGCACCAGGGTACGATCCACGTTCGCC

GCC--GAGCT----CAGCGCGAACGGG---GTCAGCTTGC----------ACACCT---G

GCGACCCAGC---------------------AGGGGCATCACCGCCTGACCCCAGTTG--

CAATCG-----CACTTGATGGGCAT-CAGCAGCTT----GCGTCC--GGCACCGGGCATC

TTGGGATAGCACGCCTGCTGCAGA-------GCCATGATCTGCTTGAAGGCGTCTTCGGC

CTTGGGGCC----CTCGCTGTAGAACATACCGCAGGATTGGCTGGT-------GAACAGC

CCG-CTCGCCGCCG--------AGGCGTCTTGCAGGCAGCAGCGCGCCTCCTCGTTC---

-TTCAGCTGGA--CCACGTTGCGA--------CCCCAGCGGTTCGTGGTGAT----CTTG

GTCTTGGTGGGCTGCTCTT----TCAGAGCGCGCTGCCCG-----TTCTCGCTGT-----

----TCACATCCATCTCGATCACGTGATCCTTGTTCA--------------GCATGCCCA

CACCGTGCAGGCAGCGGAGCCCTTGGC---CGTC------------GGTGCAGCCGTGCT

----------CCCAGA----TCGCACAG-----CCGGTGATATTCACAT---------TA

GAGGTCA-GCCCGCTGCTGCGCATGATAAA--GTCCAGC---AGGAAGCGCGCCATCAGG

GTC---TGCAGAG-----------------------ACTTCTGAGTCGAGAAG--GTCAG

ATTGGT-----------GCAGATCTTCTTCTCCTGAATCCAAGCGTGGGCCGCCTTGCGC

AGGCACTCCAGGGTGCCCGCGTC------GGGCAGCAGGGT-------------------

-CAGCTCCTTCACGTCC----ACCTTCA----GGGGGACGC-----AGAGATTCA-CGGC

CAGATCCAT---AGCTTGCTGCCACATC----TGCTCCTGGGCG---------TCCAG--

------------------GGCTTGA--GGTGGGG-----------GCGGCACTGC-----

---CGCTTCCATGTTAATTCTCTTAGCT------TTGGGCTCGCCGCTTCGGCCCGGCGA

CGGTGTGCGCGGCGGCAGCGGGGG--------ACCCGTTTCCACG---GGGGACCC----

--GGTGCGCTTGCGGCCTATCT-----TGTGCTTGACGGTACCGTCGGGACGGTGGACGA

TCTTC--------ACCGGCGGATTGGAGAACCCGATGATGCCGA----CGCCGTTGTCCG

GTTCCCCGTCGCTGT------CGTCGGAGAACTCCCAGCGGACGTTCTCCG--AGTACTG

GGGT-------------CGCGGGGCGCTTCCGGCGTCCGGCAGGTGCAGCTTCA------

---GATCGGTGGGTCTTTTGGCTTTCGACGGACCCGCCGGTGGGGGGCTGCGCCCG----

--CGGGCCGCCCCCGTCTCCAC---------CTCGTCGACCACCA---------------

-----------GCTCCTCGTTTTCGCTTTCGC-------TGCTGCTCA-GCTCCCGCTTC

GGGGTCAGAGTTTGC-----TTGCGGCGCTGCTTCACCGGGGCCGGGGTG----------

-----CGTCCCC--------------GCTTTTTTGGTGGCA------GAGGGATCTCCAC

GTCGCTGTCGCTGCCGTCGCTGC-TGAGCTGC----------------GGCACGCCGGCG

TCGATGGACCTGGC----------------------------ATTGGGGTACTGGCTGCT

-----CATCTTTCTCTCCTAGATGG------CAGAGGAGTACAAC-----AAGCAAGAGT

CAGTGGTGGCGCTCG--AGCAGCAGCAGGACCAGGAGGAGGAGACG--------------

-------CAGCAGCA--------------------GCAGGTGCAACAGCTGGCGGAGGAG

GTGGCGGAGGCGGTGCTCGCCGATGATGACGTCATCAAGAC--------GACCACCCCGA

CCCCGCCCC---------CCAC---------GCCGGAGCCCCCCAGCGAGGCCGA-----

---------------GCGCGAGCTCGCCCTCG----------------------------

AGTTGCA----------------------------------------------------A

GTCGACCC-----GGACC------------------------------GAGCCTATCTCA

GCGAGGACGTCC------TGCTCAAGCACCTGCGGCGCCAGGCGCGCATCGTCTCCCTGG

CTTTAGAAGCCCGCGCCAG-CACCCCGCTT-TCCTCCGGCGACCTCACGCTGGCCTAC--

GAACGGCGCCTCTTCTCGC-------CGCCCACGCCGCCCCTG-----CGGCAGGAGAAC

GGCACTTGCGCGCCGGACCCCCACATCAACTTCTACCCGGCTTTCATGGTGCCCGAGGTG

CTCGCCACCTACCACCTCTTCTTTTTCAACCAC-AAAATCCCCGTCTCGTGCCGCGCCAA

CCGCACCCGCGCCGACTCCCTACTCGCCCTCCGCGAGGGGGCGCACTTACCGGAGTTTCC

GGCGATGTCAGAGGTCAGCAAGATCTTTGAGGGTCTGGGCGACGAAGAGATCGCGGCCGA

GGTCAAGTCCCTGCA------AGAGAGTCA---------------CAGCGCTTTAGTGGA

ACTGAGCGGAGACAACCCGCGCCTGGCGGTGGTCAAGCGCACCGTCT---CCCTTTCCCA

CTTCGCCTACCCGGCCATCACGCTCCCACCCAAAGTCATGGACTGTGTGATGGAGCAGCT

C---------ATCATGAAAAAGCAAAAGCCTCAGGA-------GGATGACGCCGCGCCCC

-----CCGAGGACGAAGACCTGGCCGTCGTCTCCAACGACGAGCTGGCTGGCTGGCTCAA

----ACTCGCCCCGGGG--CCGGAGGTGCC---CGCCCTGCTGGAGGAACGGCGCAAGAC

CCTCATGGCCGTCACCTTGGTAAGCGTGCACCTGGAATGCATGCGCCGCTTCTTCACTGA

ACCTCAGATCATCCGCAAGCTCGGCGAGAGCCTGCACTACCTTTTCCGCCACGGCTACAT

TAAACAAGCCTGCAAGATCTCCAACGTCGAGCTTACCAGCCTCGTCTCCTACCTGGGGAT

CTTGCACGAGAACCGCTTGGGTCAGAA-CGTGCTGCACAACACCCTGGAGGGAGAGGCGC

GCCGCGACTACGTGCGCGACACCATCTACCTTTTCCTGCTCTACACCTGGCAGACCGCCA

TGGGTATCTGGCAACAGTGTCTGGAGAATACCAATCTCAAAGAGTTGGAAAAGCTGCTGC

GTCGTGAGCGTCGGGCCCTGTGGACCGGCTTCGACGAGCGCACCATTACCCAG-GACCTG

GCTGACATCATC-TTCCC--CCAGCAATTGCTGGCCACTTTGCAAAACGGCCTGCCC-GA

CTTTGTCAGTCAGAGTATGATGCAAAACTTCCGCACCTTTGTGCTGGAGCGCTCGGGTCT

GTTGCCCGCCGTCTGCAACGCCCTGCCCTCGGACTTTGTCCCCATCGCCTACCGCGAGTG

CCCCCCACCCCTCTGGGGCTACACCTATCTGCTCCAACTGGCCAACTACCTCATGTACCA

CAACGACGTGGCC------GAGGACGTCAGCGGCGAGG------GCGCCCTGCTCGAGTG

CTA---CTGCCGCTGTAACCTGTGCACCCCCCACCGCAGCCTCGCCGTGAACACCGCGCT

GCTCAACGAGACGCAGAGTATCGGTACCTTCGAGTTGCAAGGTCCGCCCGGGGCCG--AC

GGCACTCCGGGTCGCTC----GCTGAAACTCTCGGCGGGTCTCTGGACCTCCGCCTACCT

GCGCAAATTTGTACCCGAAGACTATCACGCCCACAGCCTGCGCTTCTACGAGAACC---A

ATCCAAACCCCGTAGCCAGGAGCTCACGGCCTGCGTCATCACCCAGTCCAGCATCCTCGC

CCAATTGCAAGAGATCAAGAAAGCCAGGGAGGAGTTTCTCTTGAAAAAGGGGCACGGGGT

CTACCTCGACCCTCAGACCGGAGAGCAGCTCAACACGTCGGACGCCAGCGCCGACGCTAA

CGCGCTGACGACCGCCGGTAGCGTGCGCCGTTGCAAGACCAGCGATAATCTGCCCGCCCT

GACGCAGCAGCGCCATGGTCGCCCGCAAGGGAGCCAGCAGCTACAAGGCGAAGGCCGCAG

CGAAAGCCGCGGCAGCTTCCGC----CGAGGCGGCAAAGGGGGACGTCGACATGCAAACG

GAGCCCATCAGCAGCGAGGAGGAGGAGTACAGCGAGGAGGAGGGGAGCTATACGGACGAG

GAGGACTACAGCAGCAGCGAGGAGCTGGTAGACCTGGAGGACCTGCCCGAGGAGGTCCTG

GCAAGATTGCCCCCGGAGGCGCTGGAGGCGCTGAAGTCTGTCCGGCAGACTTCGGAGGCC

CCCAAAGCTGCCTCTCCGCCAGCAGCTGCCCCATCGGAGCCGCCTGCAGCGGCGGCAGCG

GAGGCACCTCTTGCAAGCTCGCCGGCGCCACCGGCTCCGAAGAAAGCCAAGCGTAGGTGG

GACCAGGTCCAGAAAGACGGTAA--------GACCACCCCCGACCACCACGA---CCACC

CCAGCGGCCGACGCGCTTATCGCTCCTGGCGCCAGCACAAGAACGCCATCCTGGGCTGCT

TGTTGGAGAGCGGCGGCAACGTCGCCTTCAGCCGACGATACATGCTCTTCCACGAAGGGG

TCTTGCTGCCCAAGAA-TGTAATCCGCTACTATAA--TTCTTACTACAGATAATCCAGGC

TCT---------------------------CCGGTTCTGAAAAAGCCCCAAACGCGAGCG

GCT-------ACCCGGCGCGCCAACAAGCA------------AGCAGCTCCGGCCGCGGC

CAGCCTCTTCGTCGCCGG--ACCCCGCGAGCCGGCCACCACCAAGGC-CCTCCGGGAAAA

AATCTTCCCCACTCTCTACGCGGTCTTCCAGCAGAGCCGCGGCCAAGAGCGAGAC-----

----TTGAAAGTCAAGAATAGATCCCTCCGATCCCTCACGCGCA-GTTGCTTGTACCACA

AGAGTGAGGCTCAG-CTGCAGAGGACGCTCGAAGATTCCGAGGCGCTCTTCTCCAAGTAC

TGCTCGCTAGCG---AAGCGCTGCAGTGAAGCTCCATCT----------TCCCCGGCCAG

TAT-------TTA-------------------------------------AACATGAGCA

AGTCCA-TACCCACCCCTTACGTGTGGGGCTATCAACCCCAATTAGGTAATGCCTCGGGC

GCCTCCCAAGATTA---CTCCACCCGCATGAATTGGCTGAGTGCCGGTCCCGGGATGATC

AGCCGTGTGAACCGGATCAGGGAGGAACAGAATGATATTCTGCTCAAACAGGCCCTAATT

ACCGAGACACCACGCCCAGTAATGAACCCGCGGACTTGGCCCTCTG-ATCTCATCATCGA

CCCGCCCAACGCC-CCGACC-ATAGTCCAACTCCCACGGAACGAAAGTGCTGAGCTAGCC

ATGACGGACTCTGGAC--TCCAGCTGGCGGGCGGGGGTGCGGTC--------ATGCGGTA

CCGCCCTATCTC-GGGCCCTATATATGGGAGCGGA----------CTCCGAGGTGGCAGT

ATCCAGCTGAGCTCTCGGGAGGACCTAGTCC-AATACCGACCAGCTCGCCTGCGATCCGA

CGGACAGTTCCAGC--TAGCCGGAGGCAGTCGGG--TGATCTTGCCGCAACAAGGCACCC

TTCT----ACTGCAAGGCTCTTCTTCTCAACCTCGCTCGGGTGGCATCGGAGCTCTCCAA

TTCACC-CAGGAGTTCGTGCCCACCGTCTACCAGA-ACCCCTTCTCGGGGCCGCCTGGAG

TCTACCCGGACG---AGTTTA------TTTACAACTATGACCTGGTCTCTGACTCAGTGA

ATGGATATTCCTGATCTGGAGGCCTTCATTCAACTTGAAAACCAGAACCTGACCAGAATC

TGCTTGGACCACCTGCTCGAGTGCCAGCGACCCCAC-TGCGTACTCAAGTGCGGCAGGAA

-ACCTGCTTTCTTCTACT-------TTCCCGAGCCCGGACCCGAGCAGCTCGAC-GTACC

CGACTCTCTGCAGCCCGGCCACGGCCTGAAGCTCTGCTTACCACTGGTGAGTACCTGCTC

AACGGTGTTCTGCAGAGCGGTGTCTGCGGAGTGCGTGGTGA--GCTCTCGCTCCTGGGAA

CAGGCTTGG---AAGGTG-CAGTGCTGC--TGCCAGACCCCCGCCTGCCATCCCCACCTG

C----TGGATTTGCTGTGTGAAAACT-ACAATAAAAATCACCACTGATTTTGCACCGTGA

ACT--CGGACTGATGCCAGTTTGTTCTCGCGAGGCTTGCCTGCTCGCTACGGTGGTCTTT

CTCCTGGTGGTCTTTCTC--------------ATCTACCTGTACGTGGAATTTGTAGTG-

-------CCCAAGCCGGTGGTCTACGAGAAGCCC---GCTATTGGCAGCTATCATACCA-

--GCTGGTGCCGCGATCTGAATG-AGGATTAAGGAGTACCTGAGCAAGA--TGAAGTTGG

AAGCCT----------GCTTTAGCTTTTTGGGACTCTTTACCCTGGGACTGATCTGTGGA

TACTTACAACTACCCATTCCCACCACTAACCCGC--------------TGAAGAAAATAG

CCATTAATG---------TCAATGGAGAGATCTGGGCTACAGAAGGCACCTCTGTCTTCT

T-AGCCCCACCTAAGCCCCTCTA-----CAATGGCACTGGTGGATATTATGCCTGGCTGA

AGAATGGTGTCTCTATTGGATA-TAGTTTTGATGGAAAGTGCT-------TTCTGCCTCG

CTGTGCAGTGAGGAATGATGGCAGTCTGTTTCTGATGAATGTTAAAAAAGAAGATGGGGG

GAGGTTTGAAGGGCAGTTTTTGGTGGATGACAAGTTTCAAACAAAGGTGGAATATTTGGT

GAAAATCATGAGCACTGAAGAGCTCATGTCTCATATGGGAGGGGGGCCTCATTGGGGTCC

TATGAGTCCTGAAGAA-GAGTTGGCTGTGCAGCTGAAGCCCCCCAGTGGGCCTAGGCAGA

GGAGGGCAGCTATTGACGCCACTGCTGACTATCAAGTGCTGGCT-ATTACATTTTTCTTT

GGAGCTATTCTGT--TTTTCTGTATGGT----GATCATAGTGTGCTGCTATAGATGGAGG

GTGGGTAGATTGCATCAGCTCTATGGCAG-TACTGCCTTACTGATTGGAGGGG-----CT

AAAGCTCAGACCCCCATGGCGGAGGAAACTTGGGAGGAGTGGGTGGCTGAACTTCCCTGG

TGGGACT--GGCTATCTATTGCTC-TTGT-GGCGGTGGCAGCAGTATGCACAATTATAGT

AGTGGCTTATGTGCTGTGGAAATACCCACCAAGATGCAGAAGAGGAGGCAAAAATCTCTG

--GATCACAACTCCCCTTCTGCTGCTTTGTGTGGCTGCTCCTGCCT-TCACGGCTAAGA-

-CTTATCAAATTCCCG---ACTACTATCAGGTTGAAGAAGGAGGAGAGGTGATGTTTGAT

TTAGACAATGGTAGCAGGT----TTCAAGAAATGAATTGGACAAAGCTTATAGGTAATCA

GGAATA--TGCAGT-GGCTTATGCCAATGAAACTTAT-------GTGAGGTTGGATTTTG

AGGACAGGAATGAGACAGGCATTAGC-ATGGCTGGCATTTACATCAATGGCAGCGTAAAA

ATTATG----AACATCAGTAAAGAGGCAGAAGGCACCTACCAGGCTAACCTCTATTTGAA

TGGAAATCATAGC---TACCA--------------------GATTTTCATGCTAGGAGTA

GTCTA-CCATTTAAAAATGCCAGTCATAACTGTGGAAAGGCTAAACACCTCACAATGCAA

ACTG-CTGTGTGGCTGCAGTGGAGAAAATGTCACTCTAGAAGCTATAGTAGGCAACACTA

CTACTAAGCTAAAATATAGCTGGCAGGTTTTAAATTCAGATGATGTGAGATTTCTGCGCT

GCACTGCTAACAGAAACAACAACAAAAGGACCACACTGGTAAGTGTTCATGAGCCCTGTC

GAGCTGAATACATAATCCACAAAGTGGTCTATACCAATGATGAACTGCTGTGGATGCTGC

TGTCCTTGGTGCTGATGATTTTGGC-----TGCTGGAATCTGGTATTGTGTGGATGGAAT

CAGAATGCATCTCAGA--GCAAGAGATGCAATGGCTATACCCCTTTTGACTGTCATGTTG

CTCACA-CCTGCCCAAGCGGCTTTCACT--ACCACAAATTTTAACCTCAGAGTTGGAGAG

AATGC-AACTCTGCAGCTTAATGAAACCAATG-CCACTTGGTTTCTGGAAACTGCTGCTC

CA--ACTATAAATTACAGACATATTCACAATTTTTCAAAT-----CTAAGTGATGTCTGT

ACTGAGGTGCAAATGAAAAACAACTCAATT--------CACCTGCTTGGAGTTAAACCCA

CTTTGAGAACACTGACAGC-CAAAGCTTC---AAATTTTACACACTATTTTAATTTTAAC

ATCTCTGATTTACAAAGGTCTTCTAATCAATCAT-CTAATTATTGGAGAGACTACTGGTG

GGTCTACCTGGTGGCCTGCCTAGTACTCTTTCTGTTGGTGCTTATTTGTGTGATTTGCAC

TCACAAAATCAGAATGAGAAACAGAAATGC--CTGGATGTTGGGGCCTCTGGTGTTGCTT

ATGATTTGTGATGCTTCTTTCTGCTATAAATTACCAGCGCTTGATGAAAGAAATACTATC

TGTAGATATGATAGTAT------------TGGAAGTTGGGTAAGGTATGAATCAGTCT--

---CTGTCGTTATGGGAGGCACTTTTACTTTAACTTTACAATGTGGTAAACCACATAAAA

TAGTCAGGTGGTA---------TGCCAATGATGGAGCTATAAATATTCAATTCCTAGAAT

TCAATAATG-----GAACATTTTCTGACATGCCATATGTTCTAAGTGTTTTGTGTCATAG

TTACGTATACTCAATGGATTCATTTTCTTGCGAGTTGAAGTCAGATCTAAATTCAATCAT

TGCCT----CTGCTGAAACTCACGACAACAAATCAGACTTAGAAACATTTTATTTTAACT

ATGGCATTTCCACACCTGAACAAGTAATTCTTGCTGATGTGGGGGACACGGTAATTCTCC

ACTGCTTTATTGGTCCATCATGGCA--AAGTTCTGATACAAATATGGTGATTTGGAAGTT

TAATG-ATCAGTGCCTTGGATATTATAGTAACGGTGGAGT----GAACTCTGAATCTTTT

TCTAATT--TTTATTCAATCGATAAAACTGAATTTTCATTAACTATTGAAGTTAATAAAA

GTTCAATTTTTGGA--AGGTATGAATGCAAAACACAAATCAAACCCTCTAAAGAAAAACA

TGCAGA----AATTAAACAATATTTCTATAATCTCCAACCCAAAACACTTTTTTCAACA-

-GCACCTGCATTTGATCATGTAGGCAATGCT---GACAAACA-TGAATCAACTGAAACAA

ACAATTTGCCTTGGTGGGGGGCTATGTTGATTGGGATTGTTTTAATTGCTGCGATTTTGC

TAATCGGGAGTGCTGTGATTAATAAAATGACA------AATCGAACACAAAA-AGTTTTT

CTGCTGTCTCCTTTA-ATTCTAACTGCTTGCATTCCAGCTGCCGATTGTGATAATTTAGT

TAAAAAGGCATCTTTTAGTATATCAATTACACAAAATGTAAGTGCAATGACTGGCGAAGA

T-TTACTTCTAAACCCATTTGCAGAGTTTGAAACAATAGTTTGGGGAAAATTAAAATCTG

CTGAAAGTAATAAGTTTAGTAAGATT---GCTGCTGTTACTGAAGAAGGAAGTTCAAAAT

ACACTATGAACTTTCAGGAGCTCTTTTTCAGTG-------------TAGTAGATATGTTT

ATCAATGGTAGTTTGAAAATTCACAAACTAACATCTGAAAACAGT---GGGATCTATGTT

CAAGATATAAAAAAAAACAACGGTTTTGGGGCGACAATTTATATTAATGTAACA--GTCA

AGCCAAAACCATATAGCTTTCCAAAAATGATTTTTATAAATATCACAGATCAAACAACGG

TTA--C------AGTAACAACAGCGAGAAACCACTGGTGGGTCATAACGGTAGCTGTACT

-----GACTTTGCTGCTATTTTTAG-----GTATAGGGATAAAGTTTTATGTTGATAATA

ATTTGG----GATTGCCTCCCCCTTACTACCCCCCGAAGAAACCAATCTACACTAACCA-

------TTTGTTGTTGACTGCATGTTTGTGC--CTGCTGTTGCCAACTACCATTGCTTCT

CCTAACTGTAAGGTATTTAATCAACATCTTTACGGTTTATCTAATCAGT-CTCTTTTGTT

AAA--TGCTTCAGTTGACGTTTCATCTTTATTTAAGTACCGCTGGAATAAAGTTATTTCT

AATAATGC-TAAGCAGCTAATTGTTCAAAA-TCAGACCTCAAT-TCAATATTTTCTAAAA

CATAATTTTCAGCTATTAAACAATGGC-TCTCTCTTAATAAAAAATTCTTCAAGAGCAGA

TTCTGGCACCT-ATCAACTGTCTTG--CATAGCAAGCACTGGAAAATCAACTG-AAGCTA

TAGTAGTTGTGGATATA-TTGGATAAGTGTAGACCGCCAAAAATGCAGTTGACAGTGGAG

CTTTTTGGAGACTTCTGTAGAACTAAAGTGTTTTGTAATTCTACAACTTC--TAA-CACT

ATTAAATTTCCTGAACCTAAT------CCAAAAGAGGTGGATT-TTAACATACAGTCAAA

GACTAACACCTACTGGATCTATCCTAACCAAACTT-CTCCCTACTTT-GT---GAGCTGC

AACTGCACTAACCTCTACAGCTCTGCTC-ACTCCATGGTGGACTTAAAAAGAGAGTG---

-------------TGATGAAGCCAGGCACGCATACCACCTGCTCATCCATCCACAAACCT

ACATCTCCATGCACCACCTT-TGGTGG--ATATATGGACTGATGTTGTTTCTCGCTGGC-

-CTTGCCGCAGCTGCCTGGTGGTGGTGGCACAAACACCACAAGAGACCCGACAAACCTAA

TGGGCTGAAG--GACTC----TGCAGAGGAAAAACCAGCCC--TAAAAGACTTGGCAG-A

AATCGTCTAAT-CACCATGAATCTCTCCAATGCCACCCTCGAGA----CCCTGCAGATCC

AGATTCAGCAGACTGAT-ACCTTCGCCAACTACCTCTTCGTCTCC---AACCTCCTACAA

TTCATCCTCTATCCCCTCGCTGCCATGCTTGCAATCTCCATCT-TCCTCTGTCAGAGATA

TAAACTCTGTCAAAAAATCAGAGACCTCAAGGCCTACAACCTCAGC--C---TCAAACTC

AGGCAA-----TCATTCAGGACT-G--CTATGCAGCATGCCTACTCTCCCTCCTCT-CCC

AAACCCCCCGACCCCGTACCCCTCAACACCATTGTGTAATTGAACCCCTCAATAAAGCTT

ACCTCAATTG-ATTGAAAAGTTCACTCGTGCTCTGGTCTTCCTTGGGGTAGGCCTCGATC

CAACTCCCGTTCTCCCAGCTGCAGAATCTCAGCTCATGCTGACGAGCAAACAGACAGAAC

TTTTTAAAACTCAGTCCCTCAAAGT-CTCCCACTTCCTCCCCACATTT---GATCTTCAT

CTCCTTTCTTCCCGCA--GCCAACACCACAATGAAGCGAGCTGCCAGCTTCAACCCAGTT

TATCCCTTCGAAGAACAGACTCAGCCCAACACCGCTCTGCCCCCCTTCTTCAGCTCGGAT

GGGTTGGAAGAGAAACCCGGGGGGA-CTCTGGGACT------GAAAATCACCA--ACCCC

CTGGGC------TTTGACTTGGATGGAAACCTCAAACTCAAACT---------GGGAGAG

GGGATGAGGATTGATGTCAAT--GGGGCCCTCGAAAATGCTCAAACAC---TTACAGCTA

GCAAGCCCTTGG-AAATTAACAGCAATGATGAGCTGT-------CTCTAAAAATGAACAC

TTCTTCATTTTTTGTAAATGGAAGTGGAGAACTAGATATTA----TGCACCAACAATCCC

TTAGTGCTGAAGCCCCCCTGGCAATTGAACAAGACAAGATTG------TTTTAAAAATTA

ACCCCACTCTGACAGTTAGAAAT-GGACAGTTACAAATTAGCTTTCATGCTTCCCCACCA

CTTAATATTGTTAATCAAAACCTACTTCAATT------------------AGATCTGGAA

CAAAAGGGCCTCAAAGTAGAAAACAGTATGCTGAAGCTTAAGTTGCAGTCACCTTTATAT

TTTACTCCTGACAATCTCGTGGGACTAGAAAGATTTACCATGTGGACTGGAAATGACACA

TCAAATAATGCCATC-TTAGACA-GAGGCAATAACATGAATGCCAGACTAGAATTAATTC

TAAACAAAGTTGGAAATTTAGTCATGGGGATGATCAGAGTCAATGCCAATGGTTTAGGCT

CAGTTGCAAATGTGGATTG-CATGATTTATTTTAACTCTAACGGAGTGTTTGATGCTACC

AGAAGTAATCTAAAAAGTTTTGGATTTAAAAAAAATT-ATTCAATCGATACAAACTAC--

ACTGAAAATTTGAAAATGATGCCAGATTCTACTTTTTATCCAAGACATAATGCTTC-TTA

TGATCAAAATTTAAGAGTTTATGATAGAGTCTTG-GCCCCATTAAATGTAGTTTCTATG-

GTTAATGATTTCTACACTTTAAAGAT--AAGCTACAACCAAATGCAAACAGGTGACAATG

GTTACAGTTTATATTTTAAATGGAACGCCAGGGCGAC--CACTTTCAACACCACTTGGAC

TATGTTTTCATACTTGGCTGCACAGTGAAAAATAAAAGGCCGGA-AACAAAAAAATTCAA

ATTCAAG-TCATTTTTTATTGTGGGGTAAAATCACAGCGGG-GGTG----GAGTGGACCC

GGACCGTAATCCCCCCTCCCCCCGACCACTGCACGCAATACAGCCGGCGTGTCGAGTGGC

AGGATTTATAAATATCTTCGGGGCTGATCTGCACATAAGGATCCAACCGCCTCCGGCGCC

GCTGCCG-ACGGCTCAACTGCAGGGACACGG--TGTTCTC-TTCCACCAGAAAATCATCA

GAGGTA-ATGGACACAAATCCTTCACTGCTCAGGCGCAAG---TGAGGCAGTCCATTTAA

CAGGGGAGAAGTACAAGAC-TGAGAAGAAAATCACACAGTTAAAACATAATCTTCATGAG

TAAAAGCTATGCGGTAGCGATGCAAGCGGGTAAACATTTTCTGTCTCAGGGGTTCCATAA

AAT---TTAGCTTTAAACTCTTGGGCCGGTGCACAGCTATGGCGAGGCACATCTTTTTCA

CAATCCTGCGCGTGCGTCTGGCACACACGCGGGCCTGTCTCTCATTCAGCCTTTTACAAG

AATTACATTTTAAAAC-TATCCAAGTATTCAGGGTGCAATCGATCACCTTATGGGGACCT

AGCCAATTAGGATTGATAAACTGCAGATCTGACTCACGGTTAAGATGTAAGTAGATCAAG

TGCTTCCTGTGGGCAAAGATGCTGCCCACATACCTCACAGCCCGAAGCTGGTGGTAATTG

GCC-ACGTAGCGATAGAAGGGGAACATGGCATTGTAAAATCCACCTTTCATCACCAA--G

CGGCAGGACCCT---CGCAAAAGCTTGGCACCGGCCACACA--TTGTAAACTCCCGGGGT

TTTCACAGTGACAGTGGACATCCCACATCTCCCTCCCGTGATAGCAG-GAAGACTTTCTT

TCATCGATACTGAGAAGAGCTGAACAATTCCTCATGAAGTGACTGCGATAGAAGCGCTCT

CGGCGCGTCAGCAGCAAGTCCCAAGGCACAGGTAATTCCAAGTAAGGC-ACAAAGCAGAC

AATGAAGCGGTTCCCCTGCACCCGGCCCACCTCGTGTTCATCATTGCCCAAGTCCAAGCA

GGATTGATCAACATCAACAAGAGGATGAGTCGGGACCTGGGGTCGAGGCCCGCGTGGCAC

GACGGAGTTCGC--CATCCAGCATCAAAT--CTATCAAGTTGGTGCGCACCCGCTCGTAT

TTCCCGTGCAGCCAGGTGGTCTTGCGCCACAGCAAAAGCTGGAACTCGCTGCTTCGCGCG

CGGGCACGGTCACGCAGGCACACCAGATCGAGCCTATGCAGAGCAGAAGTCAAGAGAGC-

--AATCAGGCAGGAGAGTTGGCGGTGAGAGGGAGACCAAGTAGAGTAAAACAGCAG----

-GGCCTGGCGGGCTTCG-AGCAGGGCCGTCTCCAGCCAGAAGCGGTAACCCTCGGCATCG

GCA--CCAAAGATCGGGGGAGGCAGCGGCAGCAGAGACATCCTACAACACAGAAGGTTTC

AGCTCATTCCTCTGGGGCACACACACGGAACGCTCATACTCGGGATATTTAGCAAACAAC

TGCACATTAA-GCCACTCTTCCAAGCGCTGGCAGAGGGCCCACAGGCGCTGACCACAGGC

CTCGTCGCCACCGCC-CAGGCTCTCGCTGGCCACAAAGACAAACAGTCCATATTGCAGCG

GGCCACACTTCTGGGGGTTCTCCACAGCGCAGGCAAAGATCTTCTCATAGCCGCCCTTGC

TCTCAGATTCAAAGA-AAGCCCGGGTCAGC-AGTTGGATGTCATGCTTGAGCTCCTCGCT

GAAGTCTTGATGCTCGTAGATGTGGCTCACCAGAGAGATGCATTTACCCAGCTTGAAGTT

GCTCATCTTGGGTGGGGGGCGAGTAGAGCTGGAAGAACACGCGGCGGCACCTTCTTCTTC

AACAGGATCTTGAGGCACCACGGTGA-CGGTCTCG-AAGCCCGGCTCGCAGTGCAATTGA

TGAGTCAGCCAGGTGGTCAACTTCTCAGCGTAGTCCGCGATGTTAAATTCAGCACCAGCT

TCCACCAGTAGAT-CGCAGCTAAAGAAGCCCGGAGAA-AGAACTGTATTTTTAAAATC-A

AGTTGTACACTGTAATCCATGCCAAAATCATCCTGATGAAAACCCTGAAGGCAGAGAGTG

GCATCCTTCACCATCTTAAGCATAAACT--CGCTGCTATACTTGGGCTTGCAGCGGCCAA

AGAGGGTCAGACGTGAGGGGGCCATCCTGGGAAAGATACAAGAACAGTCCAT-TAGGCA-

AAACGGGCACTCTCATCCAAGCAGTTCATGAGGGGCTGGCACCAGACACCGCGCACGCGG

GGGTAGCTCAATTGTAATTC-CTGCC-TGATCCAAGAAGCCAGGCGGCGGGC--GAAATT

ATTACACAAGAT----GTGAGAGTCCAGATCGGGGTTGTCAAACTCGGGACCACCAAAGG

TGAAAAAATAGCAAAACTGTTT--GTGATCGCGTC-CGTGAGAAGGCCAAGCGGCGGTGT

GATAGATGCCCAGGTTCAAGCGAGTAGCTTCCAGAGACAGGTAAGTCTGCAGCATGTCCA

GAAAATCCCGAAAAAACTGAGGGTTGGGGGTAAAATCACCATCAAAGACCCCATAGATTT

TAAAAACCTTGGCCAGGCGAAGAGGCGAGTGGGCAGCGCCTTCCATCCTGTCAATTAAAT

TCAAACTGATTAAACTGAGGCAAGCACCAGGCATACATCTGGTGACCAAAATGCAACCTC

AAAAAGGCCTTAAGATCTTCATCCAAACGGTTGGCCAAGGTAAAGGCCCGATTCACAAAC

TGGCGGTCGGGGGCATCAAAGTCCACTCCCCCCACCGTAAAAATAAACTTGAAACGGGTG

GGATCATTCAAGGGCAGGAAAAAACCCGTGACAAAAAAGGGATTCACGGAGCCCACGGTG

GGAAGGTAATGTTCCAGCCATTTTTGTATCCAACCGGGAAGCTGACGCTGTAGGGGTCCG

AGCTCTTCGGGATAAACATCCGCACAGGTCACGCTGAAGCATTTTCCAAAGCGCACTTGC

GGGGCCATGCTACTAGACAGCAAAAGCAGCAGAATCACGCACTCTGACAAGCATTCACCA

CCGGCACACATTCAACCTCGGGGCAAAAACCCTGTTCTACAAAAACAGCCTGGATATGCT

GAGTGATGCTCTCGGCCAGAGCCCGGCTCTCGGGGTCTTCCACGTGGTGAGCGCCGTAGT

GGCATTTACTGCCCACACACAGGTAAATCGAGCTGGTCTCGTTACCGCTGTAACGTCTAA

TAAAACCGCCCAAATAAACGGTGCGGCAACCCAAACAGCAACAAAAATCATCCAGAGACA

CAAAACTGTTAAACTCGTGTCTCAAAAAATCGCCCACCGTCTGCAAAATCGTCTCCAGGT

ACCGCCCCTCGTAATGATAATCGCCATAGGCGGTATCATGCACCAAAATCTCATAGGTCC

AGGCGTATTCTTGAGGAGTGGGCGACATGGTCAAAAACCCACCTGACGGAGGAGCGAGCG

AGTTCGATCGGCAGCGAGCGGAGAGCGAAAGCGGCGGCGGAGGAGCGCAAGCCGATGTCC

GCCACCACCGCCACCGCTCACTATATACCCGCGCGAAACGGCGCCGCCCGCTCAAAGTCC

GCGCGCCGTCAGCACTTCCGCTGCCCGCGCCCAAGATGGCCGCCGCGGTCTCACGGACTT

TGACCCCGTTCCCACGCGACGTCATCGGTTCCCACGCGTACGTCGGCCGTTGACGCACGT

CACGTATTTTAAATTTTGGCGGGAAAAAGGGGTCATAAAACTGTCCTGTATTATATTTGA

TTG---------------------------------------------------------

------------------------------------------------------------

------------------------------------------------------------

------------------------------------------------------------

------------------------------------------------------------

------------------------------------------------------------

------------------------------------------------------------

------------------------------------------------------------

------------------------------------------------------------

------------------------------------------------------------

------------------------------------------------------------

------------------------------------------------------------

------------------------------------------------------------

------------------------------------------------------------

------------------------------------------------------------

------------------------------------------------------------

------------------------------------------------------------

------------------------------------------------------------

------------------------------------------------------------

------------------------------------------------------------

------------------------------------------------------------

------------------------------------------------------------

------------------------------------------------------------

------------------------------------------------------------

------------------------------------------------------------

------------------------------------------------------------

------------------------------------------------------------

------------------------------------------------------------

------------------------------------------------------------

------------------------------------------------------------

------------------------------------------------------------

------------------------------------------------------------

------------------------------------------------------------

------------------------------------------------------------

------------------------------------------------------------

------------------------------------------------------------

------------------------------------------------------------

------------------------------------------------------------

------------------------------------------------------------

------------------------------------------------------------

------------------------------------------------------------

------------------------------------------------------------

------------------------------------------------------------

------------------------------------------------------------

------------------------------------------------------------

------------------------------------------------------------

------------------------------------------------------------

------------------------------------------------------------

------------------------------------------------------------

------------------------------------------------------------

------------------------------------------------------------

------------------------------------------------------------

------------------------------------------------------------

------------------------------------------------------------

------------------------------------------------------------

------------------------------------------------------------

------------------------------------------------------------

------------------------------------------------------------

------------------------------------------------------------

------------------------------------------------------------

------------------------------------------------------------

------------------------------------------------------------

------------------------------------------------------------

------------------------------------------------------------

------------------------------------------------------------

------------------------------------------------------------

------------------------------------------------------------

------------------------------------------------------------

------------------------------------------------------------

------------------------------------------------------------

------------------------------------------------------------

------------------------------------------------------------

------------------------------------------------------------

------------------------------------------------------------

------------------------------------------------------------

------------------------------------------------------------

------------------------------------------------------------

------------------------------------------------------------

-----------------------------------------------------

>Bat AdV-D

------------------------------------------------------------

------------------------------------------------------------

------------------------------------------------------------

------------------------------------------------------------

------------------------------------------------------------

------------------------------------------------------------

------------------------------------------------------------

------------------------------------------------------------

------------------------------------------------------------

------------------------------------------------------------

------------------------------------------------------------

------------------------------------------------------------

------------------------------------------------------------

------------------------------------------------------------

------------------------------------------------------------

------------------------------------------------------------

------------------------------------------------------------

------------------------------------------------------------

------------------------------------------------------------

------------------------------------------------------------

------------------------------------------------------------

------------------------------------------------------------

-----CATCATCAATATATAACCTGGCA--TTTTTGCGTCGGGGGGTGGAGTTGTAAGGG

GGTCGTGCGTGAGGGGTGGGGAAATGAGG----TGGAGGGGTGGGGAAAATAAAGTGGGA

GGGGTGGGAAAATTATATAATACATGAGGGGTGGAGTTAGAAATTC-TGACGTAACGTGG

GAACAGACTGCCCAAAGGGGTGGAGTTGGGTGGTGTTGTTTTACGATCATTC-TGA-TTG

GTTTTTCA--CT-----AATTGGTAACAGA---TGGTCAGTGACCCGTGAGGTTAAGAAT

GTAATCAGTATTTTAGATCTGTACTGTGGCAAAATGAATGATTTTATGGGGCTTTTGACA

TTCAGAATGTGTTTTTTTCATGCTCTCCAAGGGTGGAGTGTTTTTCGAGGA---------

AATGAAATCATAATTTTA--TGATT---TTTTTCACTTGTTGTGCAAGTGGGGTGGTGTT

TTTCCACTTGGGGTGAAGTGTATCAAGGTT-AGGTCATTTTGTCTTGTGATTCCGCCCTA

TTTTTTTGGAAAAATTGAGAACCGATGTGTCCTTTTCAGTC------TTTC-ACCA---T

GAAGCACTTCAGTCTCACTTCGTTAGATCCTGTGTATTCTTTTGTGG----ATCATCTTT

TTGACTTTCCGCCAGAGGGGGACGAAACTAATGGAGAAATGGGACATTGGC---AGTATC

CCACTTTGCCGGATTTAGTGGA--TTTGGAC-------CTCGCTCCGTTAA-ACGCGCTG

------GACGATTTTGTGGATTT--------GGGGTATGA-TTCTGATAGTGATTTTAAC

ATGGATTTG-CCT---ATTTCG------GCCTTTCTGTTTTGTGATGAGGA---------

---GGATCTTACCGCGTCCGGTTCGGAAACGATT-----GACC--TTCATTGCTATGAGG

ATCTCTCTGCCAGTTCTGATTTGT-----------CTATGGAGGTTCCTAT------GTT

------GGAATGTCCAGATATTCCTGGAGTTGACTGCTCAGTGT---GTAATTTTTATCG

AAGCCGTGGGGA------GTCGATGTGTAGTTTATGTTACATGAGGTCTACGTATGCTCT

AATTTACAGTGAGTATAACTTTA------------AGAGGGTGTATGACTTTAAATTTTT

GGGC-------GCTCTTTGGGGATTTTGAGAT------------ATGACTTGTTTTTCAG

GTCCTGTCACGCCCTTGCCTGAGGATG-------AGCAGATGGAAATCATTTCGGAG--G

CAGATGGGGTTA----------TGGAATGCGTGACGC-----AGGATGAACCACTTGACC

TTTCCTTAAAAAAGAATTGAA-AA------------------------------------

---------------ATTCAGTAATCAGCAGTAAGTACATTTTTGGCTTTGGAGATAAGA

TCGTTTTGTGCCAGACTTGCTGTCTTTTGCAACGG--TATATAACT-GCATGAAAG-ATG

AAGAAGACCT------------ATAAGGACGTTCTTCGTTCTTACAATACTGTCCGAGAC

AT-T--TTAGCCGGGGCCTCG-GATAAAACTTGGTTTTTTTATCGCAAG----CTTTTTT

GCCACAGATGGGTCTATTC----AGTGCATCGAATTAAAATAGATTATAAGAAAGATTTT

TTTAATATTTTGGAGCTGTGG----CCTCAGATTTTG---C---AATTTTTAGAGATTGG

AAATACTTATTATTTT--GAGAAAATTGTAGTGCCGGAGTTAATTTTTGATACTTTTGAA

AGAACAATTGTTTCTTTAGCTTTTGTGGCTTTTTTAATTGATCGGTGGAAGGAGCAA-GG

AATTTTTTCTTGGGATTATATTATCGAAACAGCCGCTTTGCAAGTATGGCAG--------

-GCCTTCATGAG------------------------------------------------

---------------------------------------------GATGTTGT-------

-------------------GGAGAGAGAATCTGTTATGA---------------------

-----------------------------------TTCCTCGGGGGCCTACACATAG---

----GGTGCCTTTGGAATCT----ATTCCT--TTTCAGCA------AATTTT--AGATGA

AACAGAGAGGCAACCCAT--------------TAATCTTAGGAGGTTATGTATGGAGTAT

A-----------------------------------GTTTTGAAGAAATTGCTACTTATG

TT---ATGCAGCCTCATGATAATTGGACAGAAATGATTGATACTCATGTTAAGATTGCAT

TGAATCCTGGGATTATATACAAAATTTCTGCTCCAGTCATTATTGGTAGGGAATGTTATA

TTATTGGAAATGGAGCTACAGTAGAAATTGATTG---TGCTCATTCAGCTGTTTTTATTG

TTAGAAAGGAT---CGCTTTAATCCTAACATTAGAAATTTGTCTGGGGTGGTGTTTCAAA

AT-GTGAAATTTTTG-------CATAATATGTATGAAAGTGGACAGGGGATTTTGTTTCA

AGTGACTAAAAAAACTACTTTTCACAACTGTTACTTCTTTGGTTTTAATCATACTTGTGT

AATTGCTTATGAGCAGGTTATTATGCGTTCTTGTGTTTTTGAAGCTTGCTTTCGGGGGGT

TTGT---TTATATCATAATGATACGCCTTACTTTGGAACTTCTAGGATTGTGCAGTGTTA

CTT-TAAACGTTGTGCAATTGGCATTATTGCAAAAGCTTTTATAAAAATTTGTAACAATG

TTAGTCAGGAAACTTACTGCA---TGGCCTTGCTGTTTAATGGAGCACGTTTTACTCATA

ATAAAATATTAACTCC---TTATACTGCTGAAACTTTTGCTCAGAGATATGTAACTTGTC

GAGATGGAATAGTTGTGCC------CTTGCAAACAGTGCATATCAGTGGA---AATCATA

GGGTGAAATGGCCTGTGTTAAAGGATAATTTTTTTCATAGGTGCAGTTTATTT-TTGGGA

AACAGAAGGGGAGCTACTATGTTTA---AAAATTGCAGTTTTTT-CAGAACACAAATTTT

TGTGGAACCGGAAGTGGGCA-ATAAG--GTGTCTT--TTAGCAGTATTTATGGCCTAGAT

TTGTCAATCTTTAAGAT-------GATTCGTATGGCCACTGATGCAATGGAGACAGAAAG

TTGTGAGTGTGGGGCAGTTCATCGTCATCATCGTTTTATGTTTGGCGATATTACCCATGA

AGTGTTGCCTAATCCCAGGCTTG--TTTCTTGCCAAACATTGGAATATTCCTCTGCTTCT

GATTCAGGTGGG--TTAAATATTAATT-----------TTGGGCGTGGCGTAAAAAGGTA

TATAACTACCT-------GTATTTTTATTGTTTTTTATTATTTTTCAGATTAACTG-CCA

CCATGTCTGT---------TTCCTACGCTGGAGCTATTCAAACTGCTTTCGTGACCCGAA

ATTTGCCACGCTGGGCTGGAGTTCCACAAGATGTTATCGGATCTGACCTTG--GAGGAAA

ACCTGTTCTGCCAACTAATGCCATGGCCGAGATGACTGCAGGTGGAG-------------

------AGCTGCTTAAAGAAGTAGAACTTTTACGACGTAGACTTATGTT-----------

--------------------------AATAGAGCAAAAACTTAAGCTT------------

----------TTGGAAGCTAGAGCTCGTTTTGCTGATGGGGCTTAATTACTTAAAACACA

GCTATTTAT-------CACAAAAATAAAAAAGAATGCA----------------------

--------TTAAATTTTCACA---------------------------------CACCAC

TATGTAATAAAATTTTGTTAAACTTTATTT---------CTTTTGTT-GTGGTAGTATTT

ATTCCAGCGTTGTCGATCATTTAACACTTTATGAATTTTTGTTAATATTTCATATAGTTT

ACATTGCACATTTAAATACATAGGCATTACTCCTTCAGTAGGGTGTA-AATACATCCATT

GTAAAGCATCATGTTCAGGTACAGTATTATAAATAATCCAATCATACCTGGAGTGGGTGC

TATGATAATTAAAAATATCTTTTAACAATAATGTTATAGGCAGTGGGTAACTTTTGGTAT

AGGTGTTAATAAATCTGTTTAGTTGCATAGGTTGCATTTGAGGACTTATAATATGCATTT

TTGCTTGTATTTTTAAATTGGCAATGTTACCAACATTGTCTTTTCTGGGATTCATGTTAT

GTAAAACAACTAAAACGGTATACCCAGTACACTTGGGAAATTTATCATGTAACTTTGATG

GAAATGCATGAAAAAATTTAGCAATACCTTTATTGCTTCCAAGGTTTTCCATGCATTCGT

CCATTATTATGGCTATTGGCCCATCTTTTGCAGCTTGAGCAAAA-ATGTTTTCTGGGTTA

GTTATATCATAATTTTTATCATCAATAAATTCATCATAGGATAATTTAATAAATTTTGGC

ATTAAAGTGGTTGTCTGAGGAACAATAGTACCTTCGGGACCAGCTAGGTAATTTCCTTCA

CAAATTTGACTTTCCCAAGCAGAAAATTCATGTGGGGGTATCATATCAATTTGTGGAACT

ATGAAAAAAATAGTTTCGGGGGGAGGATTAATAAGTTTAGAGGATAATAAATTTCTAAGT

AGTTGAGATTTTCCACTGCCTGTAGGTCCATATATAACTCCTATAAAAGGTTGAGTATTG

TAGTTTAAAGAAGTACATTTTTCTTTACAATCTAAG------------------AAAGGA

AGAACACTTTTAAAATTTTGTTTTACATTTTTAGCATCATTTAATAAATCTTGTAAA---

-----------AGTTTATCTCCCCCTAAACA-----------------------------

AAGTAAATCATCAAAAG-AAGAAAATATATTTAATGGTTTTAACC----CATCAGCCCA-

----AGACATACATTTT-----AAAGTAGAATGCAATGTTTCTAATTTTT-------CCC

AAAGTTCTTTTAGTTCT----CCCACGGCAATTGGGTCC----------AACATACTTCG

TTGTTTCTTG--------GGTTTGGGTTGCTGTTCGAGTAAGGGA--TCAAAATATTCTG

GTTT---------------------AGCTGATGAAGTGTCTTGTCCTTCCATGGGCGCAG

AGTTCTTGTTAAGGTTGATTGAGTTACTGTGAAAGGTTGTACATTGTTCACTGTTGTAGT

TAGGGTTCGTCGTAGACTCGTACGGGAAGTCTGAAAGACTTC------------GCTGCC

CTGTTGAACATCAGCATA---ATAACAAGCTAGCATAACA---TCATAAGATAGATCTTG

TTTAGCATGACCTTTTGCCCTTAATTTTCCAGGGCCTACA------TGTTTACAAAAATC

ACAAGTTGTATCTTTTAAAGCATATAATTTTGGCGCCA-TGAATAAGGATTCAGAACTAT

AAGCATCTTGTCCACATTTGCTACATTGGGTTTCACATTCTACTAACCAAGTTAAATCAG

GATTTTTTGGATCAAATGTTAAAGAGCCCCCATTTTTTTTAATTCTGTGCTTACCTTTTT

GTTCCATTAGGTCCTTGCCTAGTTTTGTCAGAAATAAACTGTCAGTATCTCCGTAGACAG

ATTTTAGTTTTCTGTGTTCTAAAG---GAGTTCCTCGGTCATCAGCATATAAAAAATCTG

CCCATTCTGAGATAAAAGCTCTTGTCCAAGCAAGTACAAAAGAAGCAATTTGAGAGGGGT

AACGATCATTGGTTATAAGAGAGGAATTTAATTCTAAAGTGTGTAAACAGGCATCATCTT

CATCACAATCTAAGAATGTGATTGGTTTATA---AACATAAGTCACGTGC----------

----TCCTG--------GGGAGTATTTAAGGAAGTGAGTTCCTTAGTGTTTTGGTTTTCG

GGTGGCTGCTGCTGCTCGCCGGCATC-----------TGGTTCTAA--------------

------------------------------------------------------------

-AGGTGAGTATAAAACTGAAAA---TTGAGGAATAATTTGAGCGCTAAAATTGT------

------------------CAGTTTC----------TATAAATGTTGAATTT--------T

TTATTTTATAACTTCCGTTAGCAATTGATTTTTTGCAATTTTCATCCATTTGGTCAGAAA

ATAGGGTTTTTTTGTTGTCTAATTTTGTAGCAAAGGAGCCATATAAAGCATTGGATAATA

GTTTGGCTATATTACGTAATGTTTGATTTTTGTCTTGATCTGCTTTTTCTTTTGCCATAA

TATTTAAGCGGACATATTCTGCAGCAACGCATTTCCATTCAGGAAATACAGTGGTTCGCT

CATCAGGTAAAATTGTCACAGTCCAACCTCGGTTGTGTAATGTAATTATGTCTATGGATG

TTACTACTTCTCCTTTTAAAGGTTCATTTGTCCAGCATAATCTACCTCCTTTTTTGGAGC

AAAATGGAGGTAATACATCTAAAAATTCTTCTGCTGGTGGAATGGCGTCAATAGTAAAAA

TGCCAGGTAGTAGA-CTTTTATTAAAATATGATAATTTTTGGTTTTTATGCATAAGAATT

TGAAATTGCCATTTGTTAACTTCTATGTTTCTTTCAAACGTGTTTAAAGGTTTTCCTGAG

GGAAATGGGTGAGTTAGAGCACTGGCATACATTCCACAAATGTCATAGACATATATTGGA

TCTTCTAAAACACCAATATAAGTTGGATAACATCGACCACCTCGAATGCTTGATCTGACA

TAGTCATACATTTCTTTTGAAGGAGCAAATAAA------ATATTGTCTAAGGTGGAAGAT

AGCAAATTTTCTGATTTAA-----------------------------------------

----------------------------ATAGTATTTGTTTAAAGATTGCATGGGAATTT

GAACTTATGGTAGGTCTTTGAAAAATATTAAAATTTGCTACAGGTAAGTTAACTGTTTCA

GCTATAAAATTTTTATAAGAATTTAATAATTTGTCAACTAATTCTGAAGTTACTATTACA

TCTAAAACACAATATTCTAATGTTTCATCTATAATATCATATTTA---------------

---------------------GATTTTTTAGATTGTTTCCACAGTTGTTTGTTTTCTTGG

TATTCGTCTTGATTTTTCCAATATTTGGAAGCTGGAAAGCCATC---TTCATCGATT---

TCATATTGACCTATCATATAGAAATCATTTACAGCTTGGTATGGACAGCAACCTTTACTA

ATTTTTAAATTGTAAGCTTTAGCAGCATTTTGCAAACTTGT---GTGGGTTAGAGCGTAA

GTATCTCTTACCATAACTTTTAC-------------A-AACTGATATTTTAAAT--CTTG

TGCCTGACAAATTCCTT--CTTGCCAAATTTTATATTCATTTTTTCCTGGTTTAATGAAT

TGG---GGATTGGGAAGAGCGTATGTAATGTCATTAAATAAAATTTTTCCACAGCGGGGC

ATAAAATTTCTCATTATTT---TAAATCCTTTTGGAAGATCAAATTTGTTAT-------T

TATTACTTGAGCAGCTAATACAATTTCATCAAAGCCATTGATGTTGTGACCTACAATATA

TATTTCTATAA--TTTTACAGTATCCTTTAAGTT--------TTAATTTCT---TTAATT

GTTCAAAGGTAAGTTCATCAGTTTCACT-GATATTTTTTTCCGTTTTAAATTTTTCAATT

TCATCATTGTTATTTGTGATAAAGTGATGCCATAAAATGTCAGTT---AATTGTTTTTGC

ACAAGTTCTCTAAATCTTTTAAATTTATATCCAATGCTTCTTTTTTGTGGATTTATATAA

AAAAAAATATTTTCATATTTTAAAGGTTGAAAACAATCCCATTGCTCTGCATGAGCAATT

TGTGAAGCAATTTTAACAAGGTTAGTATTACCAGTAAGTTTAAAAACCAACATAAAGGGA

ACCAATTGTTTTCCATATTGACCATGCCAAGTATATGTTTCTACATCATATGTAATGAAT

AATTGTTCAGTACCGTGAAAACTACCTATTGGAAAGAAAGAAATACTTTCCCACCAGTCA

CTTGTTTGAGCATTAACACAATGAAAATAAAAATCTCGCCTACGTAAGGAGCAGGTATGA

TTATTTTTATATATTCTACCACAGTGTTGACATTTTTGCATTCGACTTACTTCATTAATA

-AGGTATAATTTACTTTTAT-----AAACTAAAAATTCTAAGGGAAAA------------

------------------------TCATACTTTTGTTCTTTAACACCAACATCAGTTA--

----------------------------CATAAAATTTTCCTCTGTAAAAGTTATAAATT

AAACCTAAAGATGGTCCACATTGCATAACTTTTGAGTGAAC---------AGAAGCGCAA

CTAATA------TTATACATACAAGGTGGTAATTCTTG---TAAATGTACATGAAACAAA

TTTTGCAATGCTTTCATAGTATCATTATAGTATTTAACTTCTAACACATTTCCACCTGTA

T---CTATTCCTTGCACGGTGTTACAAGCTCTTTGAGCTACTCTTGTGCC----------

------------------------------------------------------------

TTTTAA-----------------TGGCTTTAATCTTAAGGTGGCAGTTGTACCG---CTT

GGTGGCGTTCTGGGAGAGGAAGTCGTTGGGAGCGTAGCTGGGATGCTAATGCTACCACTT

TCCTGTTGATGTTCTGAATTTCTGGGTGTTGACTGAGAACTACTGGTCCCGTGAACTTGA

ACCTAAAGGAAATTTCCATAGAATCAATTTCAGCATCATTCA-TTGCTACTTGTCTTAAA

ATTTCTTGAACATCTCCACTATTATCATGATAAGCAATATCTGTCATAAATTGATCTATT

TCTTCTTC-----TTGTAATTCT-CCCATGCCTGCTCTCTGAACAGAAGTTGCTAAGTCA

TTACTGATGCGAGTCATTACATTAATAAA------ACCATCTTGTCCATATTCTGTCCAT

AAACGATTATAAATTACATCTCCTTCAGAATCTCTAGCCCTTAAAATTATTTGAGTTAAA

TTTAAATCAACATATCTAGTAAAGGGAATTGCTAGTCTGAAAGCATGATGAAGATAATTT

AATGTTGTTGCAATGTGTTCTGTAACAAAAAAATACAAAACCCATCTTCTTAAAGTAAGT

TCAGTAATTTCTCCCATTATATCTAGTCTTTGCATTGCTTGATAAAAATCTACAGTAAAG

TTAAAAAATCTTTGATTTCTAACTTGAGCTGATAATTCTTCTTCTAAAAGGCGGATTACT

TCAGCAATAGCTGCTCTAATTTCCTCCTCAAAA------------GTAACTGGT---TCC

TCTTCTCCTT------------CAGGCTCTTCCGCTTCCGGTGAGGAAGGA---------

-----GAAGC-GGGCGCTGGACGAGGAAT------TCTTCTACGAGTTGGAAAAGGTAAA

CTTTCTATAAATCTTTGAACAACTTCACCTCTACGCATTCTCATTTC---TTGGGTCACT

GCTCTGCCATTT---TCTCTAGGCCTTAGTTCAA----AGGCTCCACCTTGCATTT----

----------------GAAATGGACAAATG-------TTTGGTAAACTTAAAGCACTAAT

AACACTTT-TTAAAATATATTGCAGAGGCAAGTCG--------GATTGTAACATATTGT-

---TGATATTGATAGGTTCATTGAACCTTT--CAACAAACGCGTGAAGCCAGTCACAATC

ACAAGGTAAG---CTGAGTTGCATGTCGGC------------------------------

------------------------------------------ATTAACACTTAATAAATA

ATTAAAATAAGCGCTTTTTAATTGCCTAATAGTTTGCAAAATAATAACATCACTTTGGCC

AGCTTGTTCTATTCTAATTTTTT-CTGCCATTCCCCATGCATTTGTTTCTCCCAT-----

---ATTTCTT--CTTTGCTGTTGCAAATAATTATGTACAGGAACATTTTG----------

------------------------------------------------------------

-----ATCAGGCATTATATTAGTGATTCCATATCCATGTAAAGGTTGTATTAAACCTAAA

TCAGCCACTACACGGTCAGCTAAAATGGATTGTTGAATTTGTGAGAGAGTTTCTTGAAAG

TT------------TTCCAAGTCTAAAAAACGATGATATGTTCCTGT-ATTTAATGTATA

GCTACAATTAGATACCATAGACCAATTTAGTAG--TTGCTGCATTGGTCCATGTATTTCA

TGA----TACTTAATTTTACTGTAGGCGCGTGTATCAAAA-ATATAATCATTACACACAC

GTATTATATATTGATA-ACCAATTAAAAAATGGGGAGGAGGATAACTAAACAAGGGCCAT

CTAAGAGTTGATGGTTGTCTAGGACTTAAATTCATTAACATTATTTGGGGATAATTA-TA

AATAAAACGAGACATCCAAGTAAGGCC-CGGAGCAGTAGTAGAAGCGCGTGTCCAATCTT

GTACACGAGCCCAAAAATTTCTGATGGGTCTAAAAACTTCAATGGTGTACACTGATTGGC

CGGTAAGGCGAGCGTAGTCCATGGCGTTCTGAA---------------------------

------------------------------------------------------------

------------------------------------------------------------

------------------------------------------------------------

------------------------------------------------------------

------------------------------------------------------------

------------------------------------------------------------

----------------------AGAAGAAATATGGCCTTT--------------------

------------------------------------------------------------

------------CAG-ATGCATCCAGTACTGCGCCAGATGAAATCCTTCAGA--------

----------------------------TCTACTGATG----------------------

-----------------------ACACCGATTTTCCTCAA--------------------

-------------------------GTGGAGGGTGAAGGTTTAG-CTAGATT------AC

ATTTACCACCTGAGC--AACACCCTCGAGTGCAACTTAAAAAGGATAGTAGTGAAGCAGC

AATACCTAAAGTTAATTTATTTAGGGA---------TCAACCAGGAGAAGAAGCAGAGGA

AATGAGGGATTTAAAGTTTAAAGCTGGAAGGTTAAT------AGATATAGAAAAAAATA-

AAGTTTTACAAGATACAGATTT--TGAAAAAGATGAAAGTACTGGAG------TGAGTTC

TGCTCAAGCTCATCTAGCTGCAGCTAACTTAGTATCTGCATATGAACAAACAGTTAAAGA

AGAAGTAAACTTTCAAAAATCTTTTAATAATAATGTTAGGACACTTTTGGCTAGAGAGGA

AGTAACTATTGGGTTAATGCATTTATGGGACTTTGTGGAAGCATATATACATAATCCAAA

T-------AGTAAATGTTTAACGACTCAATTATTCTTAATTGTTCAACATATTAA-GAAT

CAGGGTAT---ATTTAAAGATA--CTTTGTTAAATATTGCAGATATA----GAATCAAAA

TGGTTATATGATTTAATAAATATTTTGCAATCTATAGTAGTACAAGAAACAACTTTAACT

ATTAGTGAAAAAGTGGCTGCTATTAATTTTTCAATAATTTCTTTAGGAAAATATTATGCC

AAAAAAATATTTAATTCCCCTTTTGTGCCATTAGATAAAGAAGTAAAAATACAAACTTTT

TATATGAGAATGGTTTTAAAAATTTTAG------TTTTAAGTGATGATTTAGGTGTTTAT

AGAAATGATAA--AATTGAAAAAATTGTTAGTAATTCTCGAAAA-----AGGGAATTTAG

TGATGATGAATTATTATTTAGCTTGCGTAAAGC----TTTCT------------------

------------------------------------------------------------

------TGCATCCTTCTC------------------------------------------

------AAACATGTGAAGATGA------TTCAATTG--ACACCTCA-----------TTT

TTTAGTGAA--------------------------CAACATGCAACCCACC---------

--CGGAAT-----CTAATGCAGATCCATACCACGGAAA--------------AA------

---CAAGAATGGCCACAGGCTTTTAAACGTGTTTTAGGCCTAACTAAAAA---TTCTGAT

---TTTGCAAGACAACCTAAAGGAAAT-AGATTTAGTACAATTTTGGAAACTTTTGTTCC

ATCCAGAAAAAATCCTACTTATGAAAAAGTTTTAAATATAGTTAATGCTTTAATTAAATG

TAAAGCAATTAGAGCTGATGAAGGTGGAGAGATGTTTAATGCTTTATTGCAAAGAATTAG

TAAATATAATAGTATAAATTTGCAAACTAATTTGGATCATTTAGTTACAGATGTTA----

--AGGAAGCATTAGCTCAAAAGGAACGAAAAGGTTTAAGGCCATATATGGGTTCTTTGAT

TGCTTTAAATACTTTTTTGAGCACTTTGCCAGCTACAGTACCTAAAGGTCAGGATATTTA

TTTAGCTTTCATAAGTGCTTTAAAATTATTAGTAACTGAAGTGCCACA---AACTGATGT

GTATCAAGCTGGGCCAAACTTTTATCTTCAAACTTCTCG-AA--ATGGTTCACATACTGT

TAATTTAACTAAAGCCTTTGAAAATTTAAAATCTCTTTGGGGGGTTAAAGCTCCTAATAC

TTCTA-CTTCCAGTGTTTCATCTTTA-----TTAACTCCCAACACCCGTTTGTTATTACT

ACTAATA-GCCCCATTTA-CTAATTCTACAGATATTCCTAGGGACACTTATTTAGGTCAT

TTATTAACATTATATAGAGAAACTATAGGACAAGCAAACTTA---CAAGAAACTACTTTT

AGAGAAATAACACAAATAAGTGAAGCTTTAGGAGATGAAGATGTTT---CTAACTTGCAA

GCTACTTTAAACTTTTTATTAACCAATAGACAAGCTAATGTTCCAGTAGATTATTCATTA

ACTGTTGAAGAAGAAAGAATTTTACGTTATGTTCAACAGTCTAT-CTCTATGTTTTTGCA

ACAA---GGATTTGATG--CTACAGATGCTTTAGACCAAACTGTGGCAAATCTTGAACCT

TCCTTTTATTCTAAAAATCGCAGCTTTATTAACAAACTGATGGATTACTTTCATAGAGCT

GCAAGCATGTATCCTACTTATTTTACTAATGCAGTCTTAAACCCTAAATGGTTACCACCT

GAAGGTTTTTATACCGGTGATTTTGATTTTCCTGAAGTTTCAGATGGA---CTGCTTTGG

AACGATTTTGACAGCAGTTATTT--------------------------CGGTCAGGAAC

AAAATCAAAAACTCCTTTCAGAAGATGTTAGACAGGGTGACTCCAGTAGTATCCT-TGAT

AATGGATCTGCTTCCAGACATTCTCTCC---TGTCAACGCCCTCCC----TCCTCGGCGC

CACAGCGGCCAGCCCAGTAAATAATAATAATTTCTATTTGCCCAATAATACTAATAGTGT

AGATTAT-TATTCTTTAATGAAAGATCCCTCTGATAAAAAT---------------CTTG

AAAAAGAAATGGAAACTATAACAGAAAAGTT-TGCTCGATGGAAAACTTA-TGCTCAAGA

GCATAAAGA---------------------------------------------------

---------------------------------------------------AATGCAA--

---------GATAAATTTAATGGAAGTGGTAGA-----------------TTTCATTGTT

CAATTGTTGGCAATAATGAATGTTGGTAATTAAAATTG---------------AATAAA-

----------------CTTACCAAAGCCATTATAACTT---GTGTCTTCCTGCTTAATAT

GCCAGGAAAAA-------TGTACCGAATGGCTGT--------------------------

----------TACACCTCCTCCTTCTTATGACAGCGTCTT--------------------

-----------------TAATGATAACGCTCTACAAGATCCTTTTGTTCCTCCAAGATAC

AACGCT-CCGTCTGAGGGAAGAAATAGCATTCTTTATTCTCAACTTCCACCTGTTTATGA

CACTACTAATCTTTATTTAATAGATAACAAATCTGCAGATATTGACAGTTTAAATTATCA

AAATGACAGAAGTAATTTTCTTACAAGTGTTATTCACAATAGTGATTATACACCTATGGA

AGCTACTACTCAAACTATAAAATTAGATGAAAGGTCTAGATGGGGTGGTAGATTTAGAAC

TAT--TTTACATATGGCAATTCCAAACATTACTGAATATATGTTTAGTAATTCTTTTAAA

GTTAAAGTTATGGT------AGATAAAACAGATATAG-----------------------

----------CTAATCCAGTTTATGAGTGGGTGGATTTGCAAATTCCTGAAGGTAATTTT

TCCTTAATTATGGTTTTGGAACTAATGAATAATGCAGTAGTAGAACATTATATGTCTGTG

GGTAGGCAAAAAGGAGTAGAAGAATCAGATATAGGTGTTAAATTTGATACTAGAAATTTC

AAATTGGGTTGGGATCCAGTTACTAAATTGGTTAGCCCTGGAGTATATACTTATGAGGCT

TTTCATCCAGATGTTATTTTATTGCCTGGCTGTGCTGTTGATTTTACAAATAGTAGGTTA

AGTAATGTACTAGGAATTAGAAAAAGGTTTCCTTTTCAAAAAGGATTTATTATAACTTAT

GAAGATTTAAAGGGAGGAAACATACCAGCTTTAAGAGATTTAACAGGTAAT---------

--GTTAGAGGTGAT----------------------TTGCAAAGTGTCCAACCA------

---------------TGGAAACCTTTGA------------------AAAAAGA-------

------------------------------------------------------------

------------------------------------------------------------

-----------------TCCTGATGGTAGGACTTATCATGTAGGAGAG---GATAAAGAT

GCA---GGACCTACTGATACAGCTTATAGAAGTTGGTATTTAGCTTATAATTATGGAGAT

CCAGAAAAA---GGAGTTAAAAGTTGGACTTTATTGGTAACACCAGATATAACTTGTGGA

GCAGAACAAGTTTATTGGAGTTTGCCTCTATTAGCTACTCCACCTGTAAGTTTTAGAGAT

TCTC---ATAATACCAATAATTATCCAGTGGTAGGAACAGAATTATTGCCAATGCATGCG

AGAAGTTTTTATAATCCTATGGCAGTATATTCACAATTAATTCGTGAGAGAAC-AGCACA

--AACTCACGTATTTAATAGATTTCCAAATAATCAGATTCTTATTCGACCACC-AGCACC

TACTATAACCACCATTACTGAAAACGTGCCAGCGATAACAGATCATGGAACCATACCATT

AAAGAACAGCATTTCTGGTGTTCAACGTGTTACATTAACCGATGCTAGAAGAAGACTTTG

CCCATACGTAACAAAAAGTGTTGGCATAATAACACCTAAAGTATTATCCAGTCGTACTAT

ATGAAAT-----------------------------------------------------

---------ATGGCAATTTTAATATCTCCATCAGATA-ATACCGGTTGGGGAATTGGAGC

TCGAAAAATGTATGGAGGTTTATTGGGGGGTGCGCGAGAATATAGTGAA--GAAACTCCA

GTTCTTGTAAGACAATATTATAGAGCTCGTTGGGGAAGTAAAACTAGGCA----------

-----------------------------TGGTAGGCCTGTTATAC-------------A

TAGA---AGAGTAGAAGTAGAACCAGAG---------CCTGATGAAAATGAACCTAGA--

------------CGATC-------------------------------------------

------------------------------------------------------------

-------TCTTCGT-----------------------------CTTAGACGAA-------

---AAGGTCGAAGACCTATAATT------------------ACTAGAGCAGTT--GTAGC

TAGGAAAAAAAGACGTAGGCGTTAATAATA---------------------ACTTTTTTA

TTTT--------------GTAACTGTG--CAG-----------------ACTAAT-----

-----------TTAAATAAACTCC-------------------------CATTTTA----

----------------CT-GCACAGAG----------TGGACT-----------------

GGATAATTTACT-ATGGC-----------------------------TTCTCGTCCAA--

-------TAAAAGAAGAAATGTCTCAA---------GTTCTCCTTT--------------

CTGATGAAGTTGAAT----------TTCCTCAGCCAAAAAAAAA----------------

TAAAAGGAAAA----------------GATCATAT-------------------------

--AAAGATGATCTTGAA------------CCAGAATATAAAGATATTAAAAGG---GACA

TTAAACCTATTATTAAAGAAATTGAAAAAGCT---------ATCATCGCTCCA-------

-----------------------------------CCAAGGAGAAAATATCGATGGAGAG

GAAGAAAAGTACAAAAAATTTTAAGGCCTGGTTCTGCTATAATGCTCTCTTCAGTTCCAA

AAAC---AAGA------CAAAAACGCACAGCTGAAGAAATGCATACTGATATAGATATTT

TAGATCAGGCTAGTAAAAAGGAAGGTGAATTTGCATATGGTAAAATGC------------

------------CTCATTTGGAAATTCAAACAA--ACAAAAGAAAAAGAGAAAGAGACAT

TTC------------------TCCACA------TGA-TGATTTTATTACTACTGAACTTA

AAAAACAAAAAATAGCTTTAGAGAT---GCCTGTATTAAAAAGAAAAAGACCAAGTAGTG

AAATTGAAAGTTTTAGCAAAAAAAGAAAAAGTG----ATGATTCTTTTGTAGCTTTAGAT

AATTATAATCCAACTCCAAATATGCAACCTA-----------------TAACAGAACAGC

AAGTACTACCAATTAATAGAAAAAGAGGTGGTACTTGTTTACAGCCAACATGTCAATTGC

TGGCTTCTAAAAAAAGAAAAATTGCTCAGAGGGCAGATAGAAGTGAAGCAAATATGGAAG

TAGAAATGCCTGTTAGTACA------------------GAAAATGGTTCTGAAATGGTA-

AGAGCAGATGTAAAGGTGCGACCTATTAAAA---AAGTAGCGCCTGGAATTGGTATTAGA

ACAGTTGATGTTGATATA-------------CCTTTACAAA-----ATAGTGTACAAAAT

ATTATATCT--------------TCTACTGTTTC-----------TACTTCTGAAAGT--

-----------CTACCATCTACTTTTGTTGCTCCTTTTCAGGAAACAAAATCAAA-----

-----------------------AAAGATGAT--TCCTGATGTTA-------AATATCAT

CCTTCTATATCAA----------CATCTAAAATTCCTAGAA----GGAAAAAATATCCTT

CTGCAA----------------------------ATAGCATCATTCCAGAAGCTGTCTAT

CATCCA------TCAATTGAC-CATCCACGAGCTA--AAAAAAAGATTATTCC---TGAA

GTTAGGTATCATCCTAGCATTGCAAAAGCTCGTCGTTATACTGTATAATTTTATTTTT--

------ACTTTTACTACCCGTAATTCTTAGGAC-AAAAAATGGCAAAATCATCAATGGTC

ACCTACCGGTTGAGAATACCTGTGTCTACTCGAAGACGAAAAAAAGGGCA----------

---------GCGTCGCAATGGCCATATTAAA----AATATTAAGGGAGGGTTTTTACCAC

TGCTTGCTCCAATTTTGGCAGCAGCCATTGGTGCCATTCCAGGCATTGCTTCAGTTGCTT

TAAATGCTTCTCGCAATTAAA--TTA----------------------------------

----------------------CGGG-------TAATTCTTTTTTG--------------

----------TCAGAAAAC-----------------------------------------

--ATGGAAGAAATCAATTTTGCAGCTCTTGCCCCGCGACAAGGCACTAAACCTTTGATTG

GCGTTTGGTCAAATATTGGAACCAGCGAAATGAATGGCGGCGCCATTAATTGGGGAAGTT

TGTTAAATGGTCT---------------AAAAATGTTTGGTAGTACTGTTAAAGATTTAG

GTAGACGAGCTTTAAGTAGTAGTACAGCGCAAGCATTAAAACAAAAGCTTAAAGATACAA

ACTTGCAAGAAAAGATTGTAGAAGGAATTAGTACAGGAATTCATGGAGCTGTAGATATAG

CAAAACAAGAGGTAGATAGGGCT------------ATAGCAA--------AACGTCTTGA

AC-----------------------------------------------AAATTCCTACA

-----------------------CATGCTAAAGATTCTTTACAAGAAGCA----------

GAAGAAGCTG-------ATTTGA---AAGTTTCTTTACCACCTGTATCAACTAAGCGAAA

GATTGAAGAG--------ATACAGGATTCACCCC--CAT-------------CCTATAGT

GA---------------------------------ATTGTTTGAAAAAGATGTTAAAGCT

C---------------CCTAT------------TCCACCCCCAGTTCAGTT---------

------------------------------------------------------AGGACA

TTTCTTCCCT-------------ATTC--ACAGC-------GCAATTGGCAGTCAACTTT

AAGTAATATTGTGGGCGTGGGAGTTAATTTTAGCAAAAAAAGACGGTGTTATTAAGA---

---TT-------------------TTATTGAAA----ACTTTTTTCACCGAAATCAGC--

-----------------TGAAG-----------------------------------ATG

GCTACCCCTTCGATGACGCCCCAATGGGCCTACATGCACATCGCCGGTCAGGACGCTTCT

GAATACCTTTCACCTGGCCTAGTCCAATTTGCTCAAGCAAC-AGAAAGTTATTTTAATCT

TGGCAGCAAATTTAGAAATCCTATGGTTGCTCCTACCCATGATGTGACTACAGAAAGATC

CCAAAGGCTCCAACTAAGATTTGTACCAGTCGACAAAGATGAAACCCCATATGCTTATAA

AACTCGCTTTCAACTTGCTGTGGGTGACAATAGAGTTTTAGACATGGCTAGCTCTTATTT

TGATATTAGAGGAGTTGTAGACAGAGGACCAAGTTTTAAACCTTACAGTGGCACAGCTTA

TAATAGTTTAGCTCCAAAAAGTGCCACAAACAATGTAC-------------------AGT

TTGAAGGTAAAAATA---------------------------------------------

--ATGCCAGTCAA---------------ACTGTTACAAAAACTTTTGCCCAAGCTTGTTT

TCCTACTACTTTTGATAATAATAAACTTTTGGTGGCTAGCACCACA--------------

-----TCTACTGGCCAAACTGTGGCAGCTGATATAAATTATCAACCAGAACCTCAGTTTG

GTATGGAAACTTGGGCCAGTCCAACT---GTTGCAACTAATCTAGTTTTTGGTGGCCGTG

TATTAAGTAAAAACACCCC---ACATGCACCTTGTTATGGATCATATGCTAAACCTACAA

ATATTCAAGGAGGTCAAAGTA------------------CTGACAA--------------

----------------CCCTGCTTATGCTTATTTT---------------GCAGCTGGTG

GAGCAGCTAAAGCTC------CAGATGCTGCTTTAATAGTAGAAACGGTAGATTTGGAGG

CTCCTGATACCCATTTAGTATATAAAATTGATAATGGAACTTTAAA------AACAATGA

CAGGTTTAGGACAACAAAATGCCCCTAATCGCCCTAATTATATAGGTTTTAGAGATAATT

TTATTGGGCTAATGTATTATAATAGTAATGGTAATTTGGGAGTTTTGGCTGGTCAAGCAT

CTCAATTAAATGCAGTAGTAGATTTACAAGATAGAAATACAGAACTTTCTTATCAGTTGT

TATTGGCCAGCACTACTGATAGATCTAGGTATTTTGCCTTATGGAATCAAGCAGTAGATG

ATTATGATCCAAATGTTAGAGTAATAACAAATAATGGGGTAGAAGATGAAATGCCTAATT

ATTGCTTTCCTTTAGCTGGTATAGATATTGAACAAGCTTATGCTGTTGATAAAAATGGAA

C---------AGCAGAT-----------------AAAGATGGACAA-CAAAATATTGA--

--ATTAGTTAATATTGGAAGAGGAAATGTGCATGCTATGGAAATTAATTTAGCTGCTAAT

TTGTGGAGAAGTTTTTTGTATTCTAATGTAGCTTTATATGTTCCAGATAAGTATAAAGTA

-------ACCCCACCAAATATAATATTG--CCAGAAAATAAGAA---TACTT---ATGCT

TATATGAATGGTAGATTACCTTTAGCCAATTTAATAGATAATTATGTTAATATAGGTTCT

AAATGGTCTCCTGATATTA-TGGATAATGTTAATCCTTTTAACCATCATAGAAATACAGG

GTTAAAATATAGGTCACAATTGTTAGGAAATGGTAGAGTTTGTGAATTTCATATTCAAGT

ACCTCAAAAGTTTTTTGCTATTAAAAATTTAATGTTACTTCCTGGTACTTACACTTATGA

ATGGTCCTTTAGAAAAGATGTAAATATGATTCTTCAAAGTTCTTTAGGTAATGATCTCAG

AGTAGATGGAGCAGAAGTTAGAATTCACAGTATAAATTTATATGCAAGTTTTTTCCCAAT

GGCCCATAATACAGCTTCAACTTTGGAAGCTATGTTAAGAAATGAAACTAATAATCAAAA

TTTCAACGACTATTTATCTTCTGCTAATATGTTGTATCCTATTCCTCCTGATACTACTCA

AATACCTATCTCAATTCCGTCAAGAAATTGGGCTGGTTTCAGAGGATGGAGTTTTACAAG

ATTGAAACAAAAAGAAACTCCTGCCTTAGGATCTGCTTTTGATCCCTATTTTACATATTC

TGGAACTATACCATATCTTGATGGAACATTTTATTTAAATCATACCTTTAGAAGAGTTTC

AATTCAATTTGATTCTTCTGTAAGTTGGCCAGGAAATGATAGGTTGTTGACACCTAATGA

ATTTGAAATTAAAAGAATGTAT------GATGGAGAAGGCTATACAACATATCAAAGTAC

TATGACAAAAGATTGGTTTTTAATTCAAATGTTGGCAAATTATAATATAGGTTATCAAGG

GTTTTATCTTCCTGATGAATTTAAAGAAAAAAGTTATTCATTTTTGAAAAATTTTGTTCC

TATGTGTAGGCAAACAGTAGATCAAGA----TAGAT--TTGC-AGCTTATAAA--GCAGT

TCAAATTGAACATCAG-----------------------CAT-------AATAATTCTGG

TTTTGCAGGAGCAGGAGT---TCCTGTAGTTGCTAGGGAA----GGGCATCCATATCCTG

CTAATTGGCCTTATCCATTGATTGGAAATGCAGCTGTG---------CCAATGAAAACTG

AAAAG--AAATTTCTATGTGATAAAACTTTATGGAGAATTCCATTTTCTTCTAATTTTAT

GAATATGGGAACTTTAACAGACTTAGGTCAGAATCTTTTGTATTCAAATGCAGCACATG-

CTTTAGATATGACATTTGAAATTGATCCTATGGAAGAAACTACTTATTTATATGTTTTAT

TTGAAGTGTTTGACGTGTGTCGAGTCAATCAACCCCACAGAGGTGTAATTGAAGCAGTTT

ACCTGAGAACTCCGTTTTCTGCAGGAAATGCTACAACTTAAGTTTTATGATTAAAGCAAA

GCAAGCATAATTTCTTTGCGAGGGAATTGTGTGCTTTATTCATACATTTTTATATATCTG

AAAATATATAGAG-TATTAAGAGTTTAATGCTAGCTTTTGATA-CTTTTTAGAAAGACTC

CT-CTGTAGTTATTTGT------TCAGGTTTTAGAAGAATGGGATCTTCAGAATCTGAGT

TAAAGCATATAATTACAGATTTGGGAGTAGGGAAGTATTTCCTTGGAACGTTTGATAAAA

GGTTTCCTGGTTTCATAC-AAAAAGATAAACCGTGTTGTGCAATAGTCAACACAGCTTAT

CGAGAAACTGGGGGAATGCATTGGATAGCATTTGCTTGGTATCCGCCAAGTTTTACTTTT

TATATGTTTGATCCATTTGGTTTTTCTGATGAAAAACTAAAACAAATTTATGATTTTGAA

TATCAAAACTTATTGAAAAGGAGTGCATTAACATCTACAGAAAGTAAGTGTTTAACTTTT

ATTAAAAGCACTGAAAGTGTGCAAGGAGGCCACAGTGCAGCTTGTGGATTGTTTTGTTGT

GCTTTTTTATATTCTTTTGTTAATTATCCTTTGAATCCCATGAA---AAACCCTTTTATG

AAAATTTTTAAAAGTGTCCCTAATGACAAAATTTTAACACCAAAATGT-CAAT-TTATTT

TTAAAAAAAACCAGGATAATTTATACCATTTTTTATCTCAAAAATCTCCTTACTTTAAAC

TTAATGAACAAAAAATAAAATGCCAAACAAATTTTAATAAA-CT----------------

-TCTGTAAATTTT---------------------TATTAATAAAATTTATTAC------A

AAATTTT--------------------------------------AAAACAAAGTTTCGT

CATTGTCTTCT-----------TCTCCCATG----------------GGTAAAATAGTAC

TTTGGAATTGATA-----TTGGGGCATCCATTTAAAGTTTTGAATTGTTATAGGAGCT--

----GGGCTTTTAAAAACGTTTA---GCCAA-----ATTTGTTTAGCTATTTGCAAAGCA

CAAACAACATCTGGGGCGCTGATTTTAAAATCACAATTTTTTTGGGGGTTAGCACGAG--

-----TATTTCGGTATAC------AGGA--TTACAACATTGAAATACTAGCAAAACAGGA

TGATTAACAGTAGC---TAACAATTTGGGGTCATCAACCAATGATCGATCCATGTTGGCA

ACAG--CAGA----TACAGCAAAGGGA---GTAATTTTAC----------AAGTTT---G

TCTTCCCAAA---------------------AGAGGCAAATTATTT---CCCCAATTA--

CAATCA-----CATTTTATAGGCAT-CAGAAGCAACTTATCT------GCATTATCCATT

TTTGGGTAAGCAGCTTTTTGAAAA-------GCCATAATTTGCAAAAATCCCTCTCTAGC

TTTTTTACC----TTCAGTAAAAAACATTCCACATGATTTTGTGGT-------ATGATTT

CCAGCAGGAACATT---------AAGGTCATTAGGACAGCAAGCTGCATCTTCATTT---

-TTGATTTCAA--CTACATTTCTT--------CCCCATTTATTTGTAGTAAC----TCGA

GCATTTGTTTGATTGTCTT----TAAGGGCTTTTTGAGCA-----TTTTCACTAC-----

----TTATATCTAGCTGCATTACTTGTTCCTTTGTTAG--------------CATAGCAA

GGCCATGCAAACATTTAAG---ATCATCTTC---------------ATTACAACCATGTT

----------CCCAAA----TTACACAT-----CCAGTAGGGTCCCAAT-----------

-TTAGAGTATCCAATTCACAAGACCTTATAATAAAGCTAAACAAAAATCTAGCAATCATA

CTT----AACATA----------------------CTTTTTTGAGTTGTATAT--GACAA

TTGAAG-----------ATGTTGCTTTTTTTCATTTAAGTATGATTGTACTCCCTTTTTA

AAACATTCCAAAGTACCCATGTC------TGGCAGCAAAGT-------------------

---------TAAATTTCTAATATCTACTT----TAAGTGGA--ACTAAAAATTCCACAGC

TTTATCCAT---TGCTTTTTGCCACAAAACAGCTTCATCAACCA----------------

----------------------------CATCATCATTGAGTACTTTGGAACGTTTA---

-----------------------------------------------------------C

TTTTAGTTTTAGCAAGTTCTTCATC------AACCATTTCAGACATAGTGGGCCATCTGG

GACGTTTGGAACTCTTAGGGTCGGATTGTGGAGGGGAAGTTCCATCTCCC-TCCGAATCA

CTGTCA---TTTA---AAATTGCCGGACGACGACGTCCAG--------------GAATTC

TTTTT-------------------------------------------------------

-------------------------------------------GTTTGCT----------

CCGAGCGAGTAACAGGTTTGCTAATTTGAAG-----------------------------

------------------------------------------------------------

---------------------TTCTTCTTCAC--------------------TACTGTCT

GAAATCT------------------CAGCCCGGTTATTTGCGCT----------------

-----CATTTTT------------------CCTAGATGGCAGAAGAGCAAATTCTTCAGT

CAATTGAGG--------------------------------------------------G

ACAAGAAATGAAGATGAACA----------------ATAGTGAACAGCCAAATGATTCAC

A--------TACATCTG-------------------------------------ATAGTG

ATGAAACGTG--------------------------------------------------

------------------------------------------------------------

--------------------TGAATTTCACACTCCCAATACT------------------

------------------------------------------GATGTTTCATTTGAC---

-------------CTATCTAAA--------------------------------------

------------------------------------------------------------

GATGATATT-----------------------------------TCCTCAACATATATTA

CTGAAGATTGTT------TGATAAAACATATTCAAAGACAAAGTTTAATTTTATCTAAAG

CT------ATTA---CTGA-TATTCCTATC-TCAGTAGCAGAATTAAGTTATTTTTAT--

GAACTTAATTTATTTTCA----------AAAAATATTCCTCGT-----AAATTAGAAAAT

GGAACATGTGAACCTAATCCAAATATTAATTTTTATCCTTGTTTTATAGTTCCAGAAGTG

TTAGCTACTTATCACATTTTTTTTCAGAATTAT-AAAATTCCTCTTTCATGTAAGGCTAA

TAGAAGTAAGGCAGATGATATTTTAATGTTAAAAAAAGATGATACTTTACCTACCTATTT

GACTTTGGAAGAAGTTCCCAAAATTTTTGAAGGTCTTGGTCGAGAGGAGGTAATAGCTCC

AAAAG---CTCTAGA------AGAACAAAAC---------------AGTGCATTAATAGA

ATTGAAAGGGGATAATCCTCGTTTAGCTGTTGTAAAAAGAACTATTA---CTGTTACTCA

TTTTGCTTATCCAGCTTTAAATTTACCACCTAAAGTAATGAATACAGTTATGGAAACTTT

A---------CTGATGAAAACAGTTCATCCTCAACA---------AAAA---GATGGAGA

G---------GATATAGAAGTAATGGCAGTTAGTAATGAACAACTAGCAAAGTGGTTATG

TTTAAGTACAACAGA---TACTCAAAAAA------------TAGAAGAAAAAAGAAAAAC

TATGATGTCTTCTATTTTAGTTTCTGTAGCATTAGAATGTATGCATCAGTTTTTTACTGA

TGTGCAAGTATTTAGAAAAATAGAAGAAAATTTACATTATATGTTTCGCCATGGTTATGT

TAAACAAGCTTGTGAAATTTCTCAAGTTGAATTAACAAATATAATTTCATACATGGGAAT

TTTACATGAAAATCGGCTAGGACAAAG-TGTTTTACACAACACACTAAAAGGAGAAGCTA

GACGAGATTATATAAGAGATACAATTTATTTATATTTAGTTTATACTTGGCAAACAGCTA

TGGGGGTTTGGCAACAATGTCTAGAAACAGAAAATTTAAAATTTTTAGAGAAAATGTTAG

AAAGAGAAAAAAAGAACTTATGGACTCATTTTAATGAGTCAACAACTACTGTT-TGTTTG

GCAAAAATGATT-TTTCC--TGAAAAACTGTGCAGCACTTTAAAAAATGGTTTGCCA-GA

TTTTACTAGTCAATCAATGATACAAAACTTTAGAAATTTTATTTTAGAAAGATCAGGAAT

TTTGCCTTGCATTTCTAACAGTTTTCCCACAGACTTCATTCCCCTTTTTTATAAAGAATG

CCCTCCTCCTTTATGGGGACATACATATTTATTAAGATTAGCTAATTATTTTATGTTTCA

TACTGACATAGCT------TATGATCTTACAGGAGATG------GA---TTAATGTCCTG

TTA---TTGCAGATGCAATCTTTGTTCCCCACATAGATCACTAATTTTTAACAATGCTCT

ACTAAATGAAACACAAACCATTGGTACCTTTGAACTGCAAGGTCCTCCAAAAGAAG--AT

GGCACTGTCCCGTCTTC----CCTAAAGTTAACACCGGCAGTGTGGACCTCTGCTTATCT

TAGAAAATTTATAGAAGAAGATTACCACCCCTCAAAAATTTGCTTTTTTGAAGACC---A

ATCAAAAAAGCCTCAAAAAGATTTAACTGCTTGTGTCATCACTCAATCAAATATAATTAC

CCAATTACAAGAGATTAAAAAAGCTCGCGATGAATTTTTAGTTAAAAAAGGTCATGGTAT

TTACTTAGACCCTATTACTGGAGAAGAACTTAACGGAGTTCAAAGCTCA-----------

-------------------------------------------------ACCCACCACAA

TGCCTTCAAAACC-----------------------------------TACAAAAAATCT

GACAAATTGCAAAATTGTTCAA--------ATGCCAGAACAAACAACCATG---------

------------------------------------------GAGGCGCCAGATTCAGAT

GTGGAGAGCAG-------------TCAGGCCAGTTTGGAAGAGGGGGAGATTCCACAATC

ACCTCCTATTCCTCCGCAGCCAGCT-----------------------------------

-----------------------------------------------------GCCCCCA

AAAAGAAG-------------------------------------------AGTAGATGG

GACATAAAATACAGCAAT---AG----CGGTAAG--------------------------

-----------AAGGGCTATAAATCCTGGAGGGTTCATAAAGCTAAAATTTTGGATTGTT

TAATTCATAGTAAAGGAAACTTGTCTTTTACACGTCGTTACATGTTAATGCATAATGGAG

TATTATTGCCAAAAAA-TGTAATTCATTACTATGC--CAATTCTTACTACA---------

-----------------------------------------GAAGCC-------------

--------AGGAAAACAAGTCTTAAAGCT-------------------------------

--GCTAATG-----------------CTTCTACCGCTGCTGCAGAAAAGCTAAGAAACTC

TATTTTTCCCACTATCTATGCCATTTTTCAGCAAAGCCGGGGACAAGATAAGCAA-----

----TTAAAAATTAAAAATCGCACTCTTAGATCCCTAACCCGCA-GTTGCTTTTACCATA

AAAATGAAGCTCAG-CTCCTGAGGACCCGCCGCGACGCAGAGTTGCTGCTGCAAAAGTAC

TGTA----------CCGTTAACAAAACTGCTT-----------------------ATAAA

TAG------------------------------------------------TTATGAGCA

AGGAAA-TACCAACCCCCTACGTATGGACCTTTCAACCCCAGATGGGAGTGGCCGCTGGA

GCCTCACAAGATTA---TTCCACCCAAATGAATTGGCTTAGTGCCGGAAATAACATGGCT

CAGAAAGTATTTCAAATACGTAATGCCAGAAACAATGTTCTTGAAAAACAAGCTGTTTTG

ACAGAAACACCGCGCCTAATTCAAAATCCTCCTACTTGGAAAGCT--AAAAATATTCAGC

GACCTTGGCAAATTCCAACT-GTTCTTAAATTGCCTCGTAATCAACTTCTAGAAAATTAT

ATGTCAAATTCTGGCA--TGCAATTAGCGGG-----------------------------

-----------------------------------------------------TGGTTCT

GTTCAACTGAGCGCAAACAAT--TATACTGG-TTTACCCATC----------CGTTCTGA

TGGAATTATGCAAC--TAGCGGGAGGAGGAAGAGA---AATAACTCCTCTTAATG---CT

TATTTAGTGTTGGAAAACAGCTCTTCCCGGCCTAGAAGTGGTGGAATAGGAGCTAATCAA

TTTATA-CAAGAATTTGTTCCCACAGTTTATTATA-ATCCTTTTTCTAGTTCTCCTAAAA

ACTTTCCTGATG---AATTTA------TTTCTAACTATGATATAATTTCAGACTCTGTAG

CTGACTATAGTTGAACACATGGCAAGAGTGGATTTTGATGCTATATTTTCTGATGGCACA

TTTTCTGAACAACAGACATTATTTAGAAGAGCT-----GCAGATTTTAATGCTAAGTATA

AAGAAGAATTAAAAAATC-------TTATTCTTTTACATAACTGTAAAAAAGGTGTTTTT

TGCCCTGTTAAAATGTGT----AAATTAAATTTTG-AGAAAACTAATAATGATCATGTTC

TACAATTTGAACTTTCTCCACAAATTACAAATTTGACACAAAGTTGTCAGATGGGAAACT

CTATTACTGT-AAATGTTATAGGTCAAGGCTACAAAGGATATATTAATTGCTCTTGCACA

T---------TTTCAGAATGTATGCCTACATTAATTAAAACTTTATGTAATCTTCATTTA

ATA--AAACATTATTAATAAAAAAATTTTTCTTACCTTAAAATTTTTTTGAATGTGTTTT

TTTTAAGGCGACCATAAGCTTCAATAT----GCTCTCCTTCTTCCCAGCTTTGATAATGA

-----------ATTTTATATTTATAAGCCACTTTTCTAAACTTTTTAAAATTGATTTTT-

--TCTTTTAGTGCTTTACCATTATAAAACAAACTCATGATGAAAAGGCGAGAACC-----

-------------------CCCTGTCGTCAATCCCGTTTATCCT---TATGAAGTGAAAC

GTATTAACTTGATGCCTCCTTTTTATGATGCT------------------AATGGCTTCA

CAGAAGAAA------------CTGAAGGGGCGCTCTCCCTTAAAATTTCAGATCCTTTTT

CGTTTTCTGAAAAAGGAAATTTACAATTAAAATTAGGAAATGGATTAAGAATTGATAATG

ATGGAGATTTGGAAACTGATGCAAAATCAGCTTCATTAGCTAC--------TCAATTACC

TTTACATGTAAATGTACATGAAGTATTAGAATTGTTATATTCTGAAAGTGATGGTTTATA

TTTAAATGAAAATAGT---TTAGCTGTTAATCTTAATCCTCCTTTA-ATATTTACAGATA

AAAAAATATCTCTAAATATGGATAA---TAGTCTTATGGTTGAAAATAAAACTTTAAAAA

TAAAATTTATTCCCCC-TTTCACATTAACAGATTATGGGTTAGGAATATCTT--TAGGAA

CAGGTTTAACAATTAAAGAGTCTTCTTTAGAAGTTGTTTTTCCTACTGGACAAAGTCCTA

TAGTTGCAGAAAG-------TGGAAAAATAAAACTAAATCTAGGTCAAGGATTAATTACA

ACTAATAACTTATTACAATTAAATGTAAATAGTCCT--TTTAGTTTACTAAA-------T

AATAAACTGCAATTAAATGTTAGTAATGGATTACAGATTGTAAATGATAAATTGACTTTA

AATTATGGAGATGGCTTAACCTTTGTAAA-----TGATAAACTTCAAATTTTAACTGAC-

---GATACCTCAG-GTTTACAACTTTTAAATAAGGCTCTACAAA---TTAACGTTGGTGA

TGGCATGCAGATTCAAGATAACAAATTAACTATAAAACTTGGGAAAGGTTTAATTTTAGA

TGCAGACAAAAATATC---TCTA---TAGCTTCCTTTAATATTACTTTTACTCCCCCATT

ATTTTTTCAGAATAATACAGTGTCTTTAC-GTTATGATACTT-TATTTACAATAAATGAA

AATGGTTTATTAAATTTAAAAATATCAAAAGGATTAAA-TAAAAGTGTAACAGATGGA--

ACTTTAAATGTTTTGTTAGGAGATGGAATGCAGTTTTCAGATAATGCTATAGTACCTTCT

TTAGGTAAAGGAATTCAACTAAACAATAAAAAAATAGAAATATTTTG---TGGAAAAGGG

TTAGAGTTATTAAGTACAGGA-------------------CAATTGCAAGTAAAGTT--A

GGAAAAGGATTAGTATTTGGAAGTAGTAATGAAATTTTCCCTAGCATAGCACTAAAATTA

GGAAATGGTTTAG--AGCAAATAAATGGAGGCGCTATTAGTGCAAAAGTTGGAGATGGTT

---TACAATTTTCTGCTCATAAAGAAATTATACCTAACTTAGGA----------GATGGT

CTTGAAAT-----------TGCAAACAAAAAGATTAAAG-----------TTAAAGTAGG

TGAAGGGTTAGTTCTTA-ATAACACAGGCATTAAA-----------ATGTTGCAA-ATGC

C----TAATGTTGTTTGGACTG---------GGGCAGACATGAATAATAAT--GTTTTTG

AAAACCAGGGAAGAAT--ATTTTTATATTTACATTCTTATCATGCTT--------TAGTG

ACAGGG-TTAATACAGATTTATTTTCCT--AT---------AGCTAAAGTAAATGAGTCT

CATATAGAGTTTGCTTTATATTTTGG----------CGCCGATGGT--------------

-----AGTTTATTGAGTTCCAGTGATTTAACTGTTGATCA--------TTGGATCCCTAA

AACT---AATTTC--------------------CCTTCAAATTCCATGTTACAATTTATG

CCCAATAAAAATATATATA-TGAGAGATAGA--------------------GAACAATTA

AACTACCAGGGGATGAATTATATGACAATTTTAA---CTTATTTTAATTCTTCTCAAAAA

AA-CAATGTTTTGGGAA-CAGTTAAA-ATTAATTTTAATGTAGAGCAATCAAGTGTTTAT

TCTTTAGTTTTTAGGTGGGGCCCCGTGG-C--TGGAACGGAATTTACTGATGAATTTATT

TGGACTTCTATGTGTCA--------ATTTTCTTATATCGCCGAACA---ATAAAA-----

---------ACTCATAAAACA---------------------ATTTCTTTCTTTTAGTGG

TTTT-TATTATTAGCACCGAGCAT-ATGTAAAATTATAAAATTTAATG------------

----------------------TTATATTT------CATTAGTCTTCT-----AAACCCC

TTGTCTCTG-----TTTTTTTCTCTTGTATTGTGCAATATAAGAGCTCCACTTCTTCTAA

GAATTTTTATTGCCCT-----------------------------TCT---TAACAATTT

TCGGGT---------GCGTCGCCTACAACATGCAGAAGCAATAGAAGTAAGATTTTTACA

ATAGGTGCAACGTAAAACTAAC--ATGTTTTGCTTAGTTTTAATA-AAGCAAGCATAACC

AAAAGACATA-----TATCTTAGCACAA-ATCTTAATTCAGCTAGATGTTTAAATCTTAC

ATAGA------------------TTAAGTGATATTCCCTAAACATTACACTTCCAACATA

---CACAATATTACCAGGTAAGCGATGGTTAACATAGGATCTGTAGTC-----TTTGAAC

ATAACATTATAATTGCAACCAAAAATAAATTCTCCATGCAGCATACTCATTACTTGTCTG

CCAGCT--AGACATTGTAAAGAA---------GTCTGCCCT-TGACAATGGCAATGTACA

TCCCAATCTTCATGTCCAAAAA-TATATCTGTAAC-ATTTAGTTAATTTTAAAG-AACAA

AAACAAAAAATTCCTAATCT--TTTTAAAAGGTTATAAATTTGTCCAAAAAAAAGTAAAT

CCCATGGAATTACTAACTCACG-CAAAGGTACA-----AAAACACTGCAGCCTGGATTGC

TTCTAATGCGACCCACGCGGTGAGAAGATCTCAAAGTACATTTTGACATTGGAAGAGCAG

CCATCTAAA------------AACAAAAATATGTTTTCTAAATTTAATAATATTTAGCTC

TATGTTTTCCAAGCACAAAATCT-GCGTGAATTTCCATGCGTTGAATTTTCCAAAAAATA

TGCATAACTTCTAATTCTATCAT-------------ATACCATAGCTTTAAAGCTTTATA

AGTAGTTTCACTTGTTTCGCATACATTAGGAT-----------------GATTATTATGA

ATATTATTCCAAAGAGTAAAGTAAATAGCCTTAACACACCTAGCTACACGTCTTTCATGA

CGTATTCTAAAATAATATTTTTTAAACAAAGCA--TATATAGGATCTAGACAGCG-AGCT

ACTTCTTTGATAGTATCTGCAGTATACAACATTTTACAGAA-------------------

----------------CATGATAAAA-----CCAATTATCAAGAGGAGTGACAGTGA---

-------GATCAGAAAAATC---------CAAACGAACGTTTACTCTATAAGCTATATCC

AAAAAATG-TGAACGAAGGCTTGTTTCAAATTCAGCAGCAAAAGCATGCACATCGTGCAA

ACAAGTTCCATGGTAAT-CAGGACCTGCTAC--AGACACAAGAAAAGTACGA-TAATCAT

GAATAGGAAAGGCCGTTACATAGATAGAAT------GTTCAGGTTCATGATTTAATTCCC

AAAA-AGTTTTTAGTTCATCGCAGCAGGCTTTTAGCAGCGCTATAGTAAAACTGGGTTGC

AAGAAATTTAATGCCTGTT--TAA--------AATCTTCAGTTACTGTCACTCCCAGCAA

ATAA-GAGGGT-GTAACGCGAGGCGGCATATTTACA---CTGTGTTAGGTGAATCCTTAG

GATCTTCTTCCAAGGCAGGGCAAAACAAATGACCACAAAGAACTTGAGCCACC----GGA

TCTCCAATACAGACAGTA--TACTTTTCAGCACTAAAATTGTAAACAACA-ACACA----

------------CACGTTATACATATTTTCAACATTTAATTGACTGCCTTCTACACCCAA

AATGCTACCCGCAGAGGCTGGCAAGTAAGGTAAAATACGCGCA-GCGGTGCCTGGTGGCA

GCCGCAAAGC-AAGGTCCGTATA-AACAAGCATCTTGCCATTTGGAG-GAATGACATAAT

GATAAGCGCTGCAAATAACCATTCCCAATCCTCCGTTAGGGTCGCGACGAGGCGGTAGAG

CC--------CGGCGAGAAAGACGAGTATAACGTAAAGCTCCATTATAGTTGCAGTTAGA

ACTCATTTCTAAAAAAATAAAAGTGAGC-ATTAACTGCTTTACTAAGAAAAGTCCCAAAA

ACTTTCTCCAAAACCCACTTAAATGTAACACTTACCAATAGCACCAAGAGAGC-ACAATT

TCCAAAGATTTAATGAAGCCACTGCGTCTCCCACAGCTTTTTTCTCTTAGTTGGACAGCA

GTTCGGGAAAACTTGCTGTAAAGCTGCAAGGGAAAAGGCAGACC-TTTTTATAATATCCA

AAAACATCCTCGGAATTGACAAA--AAAACAAGCTCATAAAACAAAAAAAATAGCCTCAA

-CCCAAGCGGAAAACAAAGCCAA------AAAAT---ATGGA-----------AAATTTT

TCCCCTTAAAAAA--ATCTCAAGATCTCATATCCTGCCCCTGACCCTGAACTTCTTCCTG

ATA-AAAACCGTTATTTCTCCTCCCAACAGCTGTATCTCATTAGAAGTCTGGAACTGGGC

ATACCGCCAGTGATGTTTTAA-TGTTCCA-GGCGTTCTGCCAAGATTTTAAGCGAACAC-

-AAAAACAGGCTCCCACCCATTCCCGCTCTCTAAAAACTTTCCATAACCATTTTCTCAA-

-AAATTCTCACTTTCTCCCACTCAAAACCACAAAAACCATACTCACAGCAGTCTAAACTC

CT-CGAAAACAGTAATCT---TGGAGAGCAGAAGTTCCTCATTTTCACT---TTTGACTC

ACAAAATCCACGAA--CTGTGAAAC-CGCAACGGTCATAGTCAGCCATCTTGACCG--TA

AAATGCGCTTAGGCGTGACCAGCTGTCAACAGTTA--ATT-TGTAGTCAGCTAAAAATAA

ACCCTCCACCCCTTCCGGAGTTAAACCCACCTCCAT-------TTCCTGTATAGTTTCAC

TTCCTTTCCCAGTCAAAAACCGGAAGGCCTCTTTAACCCCTTCCTTTCC--TGCTTACCC

ACCCCTCACGCACGACCCCCTTACAACTCCACCCCCCGACGCAAAAAT-------GCCAG

GTTATATATTGATGATG-------------------------------------------

------------------------------------------------------------

------------------------------------------------------------

------------------------------------------------------------

------------------------------------------------------------

------------------------------------------------------------

------------------------------------------------------------

------------------------------------------------------------

------------------------------------------------------------

------------------------------------------------------------

------------------------------------------------------------

------------------------------------------------------------

------------------------------------------------------------

------------------------------------------------------------

------------------------------------------------------------

------------------------------------------------------------

------------------------------------------------------------

------------------------------------------------------------

------------------------------------------------------------

------------------------------------------------------------

------------------------------------------------------------

------------------------------------------------------------

------------------------------------------------------------

------------------------------------------------------------

------------------------------------------------------------

------------------------------------------------------------

------------------------------------------------------------

------------------------------------------------------------

------------------------------------------------------------

------------------------------------------------------------

------------------------------------------------------------

------------------------------------------------------------

------------------------------------------------------------

------------------------------------------------------------

------------------------------------------------------------

------------------------------------------------------------

------------------------------------------------------------

------------------------------------------------------------

------------------------------------------------------------

------------------------------------------------------------

------------------------------------------------------------

------------------------------------------------------------

------------------------------------------------------------

------------------------------------------------------------

------------------------------------------------------------

------------------------------------------------------------

------------------------------------------------------------

------------------------------------------------------------

------------------------------------------------------------

------------------------------------------------------------

------------------------------------------------------------

------------------------------------------------------------

------------------------------------------------------------

------------------------------------------------------------

------------------------------------------------------------

------------------------------------------------------------

------------------------------------------------------------

------------------------------------------------------------

------------------------------------------------------------

------------------------------------------------------------

------------------------------------------------------------

------------------------------------------------------------

------------------------------------------------------------

------------------------------------------------------------

------------------------------------------------------------

------------------------------------------------------------

------------------------------------------------------------

------------------------------------------------------------

------------------------------------------------------------

------------------------------------------------------------

------------------------------------------------------------

------------------------------------------------------------

------------------------------------------------------------

------------------------------------------------------------

------------------------------------------------------------

------------------------------------------------------------

------------------------------------------------------------

------------------------------------------------------------

------------------------------------------------------------

------------------------------------------------------------

------------------------------------------------------------

------------------------------------------------------------

------------------------------------------------------------

------------------------------------------------------------

------------------------------------------------------------

------------------------------------------------------------

------------------------------------------------------------

------------------------------------------------------------

------------------------------------------------------------

------------------------------------------------------------

------------------------------------------------------------

------------------------------------------------------------

------------------------------------------------------------

------------------------------------------------------------

------------------------------------------------------------

------------------------------------------------------------

------------------------------------------------------------

------------------------------------------------------------

------------------------------------------------------------

------------------------------------------------------------

------------------------------------------------------------

------------------------------------------------------------

------------------------------------------------------------

------------------------------------------------------------

------------------------------------------------------------

------------------------------------------------------------

------------------------------------------------------------

------------------------------------------------------------

------------------------------------------------------------

------------------------------------------------------------

------------------------------------------------------------

------------------------------------------------------------

------------------------------------------------------------

------------------------------------------------------------

------------------------------------------------------------

------------------------------------------------------------

------------------------------------------------------------

------------------------------------------------------------

------------------------------------------------------------

------------------------------------------------------------

------------------------------------------------------------

------------------------------------------------------------

------------------------------------------------------------

------------------------------------------------------------

------------------------------------------------------------

------------------------------------------------------------

------------------------------------------------------------

------------------------------------------------------------

------------------------------------------------------------

------------------------------------------------------------

------------------------------------------------------------

------------------------------------------------------------

------------------------------------------------------------

------------------------------------------------------------

------------------------------------------------------------

------------------------------------------------------------

------------------------------------------------------------

------------------------------------------------------------

------------------------------------------------------------

------------------------------------------------------------

------------------------------------------------------------

------------------------------------------------------------

------------------------------------------------------------

------------------------------------------------------------

------------------------------------------------------------

------------------------------------------------------------

------------------------------------------------------------

------------------------------------------------------------

------------------------------------------------------------

------------------------------------------------------------

------------------------------------------------------------

------------------------------------------------------------

------------------------------------------------------------

------------------------------------------------------------

------------------------------------------------------------

------------------------------------------------------------

------------------------------------------------------------

------------------------------------------------------------

-----------------------------------------------------

>Bat AdV-E

------------------------------------------------------------

------------------------------------------------------------

------------------------------------------------------------

------------------------------------------------------------

------------------------------------------------------------

------------------------------------------------------------

------------------------------------------------------------

------------------------------------------------------------

------------------------------------------------------------

------------------------------------------------------------

------------------------------------------------------------

------------------------------------------------------------

------------------------------------------------------------

------------------------------------------------------------

------------------------------------------------------------

------------------------------------------------------------

------------------------------------------------------------

------------------------------------------------------------

------------------------------------------------------------

------------------------------------------------------------

------------------------------------------------------------

------------------------------------------------------------

-----CATCATCAATAAATAACCGGACA--TTTTTGCGTCGGGGG-TGGAGT----AGGG

GGCGGAGGACGTATTGTGGG----------------AGGGATTTATAAAATTTGAATAGG

GGAGTGGAATATAGATAAAAAATTTGAATATGGGAGTGGTGTATTTGTGACGTATTGTGG

GAAAAATTTTTTCAGATTCTCTAGA--GGGTGGAGTTGTTTTTCCACTTTTAGCGG-TGA

ATTTTATGACCT-----AATTAGGCTCGCA---TGAGTAGT-ACTACAGAGGAAAGGAAT

TTTATCAGTGTTTTTGATCAACAGTGTGGCAAATTTGTTTTTTTAGTGTATTTTTTCGTG

ATGAGAATAT--------TATACAGTCTAAA-------------TTGGGAG---------

AAAAAATTT----TTCCA--CTATT---TTTTTTAGCTGTTGTGTAA-CGTTTTGGGATT

TTTCCATT--------ATCTCATTTAGGAG-AATGTACTGGG---TGTGGGAACATCCTG

TTTTCT-GACTATTTTTTGAGTAAATGTGTATTTTTTTTCA------TTTTGACCA---T

GAGACATTTCACTTTAACTGGGTTGGAGCCGGTCTATTCTATTGTGG----ATGACTTTT

------TCCAGACAGAAGCCTTAGGAGAAGGTATGGATATTTCTGATG------ATTTTC

CTTCTTTACCTGACCTTGTGGA--TTTGGAT-------TTAGCCCCATTAA-ATAATATT

------GACGATTTTTTGGATTT--------GGGCTATGA-TTCCGATTCTGATTTTAAT

ATGGATGGG-CTTCCGGTATCG------GCTTTTTTATTTTGTGATGAAGA---------

---TCAAAACGGCATTAATTGTTCTGATGAGACTCC--TGATC--TTCGCTGTTATGAGG

TTATGTCGCCAAGTGAAAGTGAG--------------AGCGAGGCACCTTC------TCT

------TTCTTTTCCTGAAGAGCCTGGTGTTAATTGTCCTACTT---GTGAATTTCATCG

TAGTCGTGGCGA------CTCTATGTGCAGTCTTTGTTATATGAGGTCTACATATGCATT

AATTTACAGTAAGTAT--TTTTT------------TTTGGGTTTTTAATTTTTATTTTTT

AAATTTAGA--GTTTTTTAGTGTATTTAATTT------------TTTATTT--TTTTTAG

GTCCTGTGACTCCCCCTCCTGAAG----------AGACTGCGGAAAACATT--------A

CAGATAGCTTTA----------TGGAATGCATGACGC-----AAGATGAACCACTTGACC

TTTCTGTGAAACGAAGGAGAACAA------------------------------------

---------------GTTTTTTATAGATTTTTGGGTAAGTTTTTTATCTCTAA--TCATT

TCACATTCCACACATTTTGTTTTTTTACACAGAAG--AGGATAAATAGTCTGAGAGCATG

GAAGCAAACT------------ACATTCAGTATTTGAAGTCCTACTCTGATTTTCGGGAC

TT-A--CTGAATGGAGCTTCG-GCCCGGACTGGTTTTTTATATCGTTGG----CTTTTTT

GTGATAAACTAACTTATTG----GGTTTATCGAGTGAAGATTGAAAATCGAGTTATTTTT

GAAGAAATAGTAAAAGATTTT----CCTGAGACTTTT---TTTGAATCTTTAGAAGCTGG

TTATACTTGTGATTTT--GAAGACTCAATTGTTCCTCAATTGGAATTTTTGTCTTTGGGA

CATACTTTATCTTCTTTGGCTTTTGTGGTTTTTCTTATTGATTATTGGAAAGAAAAT-TC

TGTTTTTTCCTGGGATTTTATTATTGAAAGTTTTGGCTTAATAACATGGCGA--------

-GCAATAATAAT------------------------------------------------

---------------------------------------------GGCCTTCA-------

-------------------CCAGGGAGAAGCTGTTGTAA---------------------

-----------------------------------TTCATGGGGCGGTAAGCCAAAG---

----AGTACCTATGGAATCT----GTTCCT--TTTCAAAG------AGTAAT--AGAAGA

AACTGAAGCTCATCCTGT--------------TAGCTTAAGACATTTATGCATAGAATAT

A-----------------------------------GTTTTGAAGAGTTAATGACTTATA

CT---ATGCAAGCAGAAGATAATTGGAGTGAAATGATTAATACTCATGTTAAAATAGCTT

TAGATCCTAAAATTGTTTATAGAATTCGAGATAGTGTTACAATTGATAGAAATTGTTACA

TTATGGGAAATGGAGCAAATGTACAAATAGAAAT---GAGTGAAGGATCTGCATTTATTG

TTAGAAAAGAT---AGATTTAATCCTATCATAAGAAATATGTCTGGAGTTACTTTTCAAA

AT-GTAAAATTTTTT-------GCTGGAGATTGTACACGTACTGAAACTGTTGTATTTCA

AGTAACTAAAAAAACATATTTTAATAATTGTTTGTTTTTTGGTTTTACTTATACATGTAT

TTTAAGTTATGATCAAATTGTTTGTAGGTCTTGCATTTTTGAAAGTTGTTTTAGATGTGT

TTGC---TTATATCATAATGATTCTCCTTATTTTTCTCCTTCTAAGGTATCTCATTGTGT

TTT-TAAACGTTGTGTTTTAGGGGTTAATTCTAGAGCTTTTATAAGGGTTAAAAACAATG

TAGCTTATGAAACTTATTGTT---TAGCTTTGTTATATGCTGGTGGATTTTTTGGTTATA

ACAAAATTTTAACTCC---TTGGAATGTTGAAACTGTTAATGTAAAGTATTGTACTTGCA

GAGATGGGATTATATTACC------TTTACATACTGTTCATTTTGGAGGT---AATACTA

AAGCTAGATGGCCTGTTATAAAAAATAATGTTTTTACTAAATGTAGTATTTTT-GTAGGA

TTTAGAAGAGGAACTACTATGTTTA---GAAATTGTTGTTTTTA-TCAAACATCTATGTA

TGTTGAACCTGAGGTTGGGA-ACAAA--ATTTCAT--TCAGTGGTGTTTATGGTCTTGAT

TTGGCAGTGTATAAGAT-------GCTTAGATACACTTCAGACTTTGTGGAAACTCAGAA

TTGTGAGTGTGGTGCTGTGCATCGCCATACTCGGGTTATGTTTGGAGAAATTACTTCGGA

AGTGCTGCCAAATCCTAGACTTA--TGTCTTGCCAGAGTGCGGAATATTCCT------CA

GATTCAGGTTTG--TTAATCATTAATT-----------GAGGGCGTGGTTAAAAAAAAAA

TATAAAAATTAAAAAATAGTAAAAATATTTTCATTTTTTATTTTT-AGATTGACAGACTA

CGATGGATAC---------TACTTTTGCTGGCGTTATTAATACTGCCTATTTAACTCATA

ACATGCCACGTTGGGCTGGAGCTGCACAAAATTTAATCGGCACTGATCTTC--AAGGACG

TCCTGTTCTGCCTACCAATGCCACGCCTGATACTAC-ACCGCTGGA--------------

-------CCTGCTTAAACAAGTTAACAGATTGCAAAAACGCTTAAGATC-----------

--------------------------AATAGAAAATAAAATAGCCCTT------------

----------TTAGAAGCAAAAA------TTACTG------CTTAAAGTTTTAATAAATA

--TGTTTA--------------AATAAAAAACAAT-------------------------

---------TACTCTTTTACA---------------------------------GT----

----TAATAAAAATT--TTAAATCTTTATT---------TTTTTGTT-GTGGTAGTATTT

ATTCCAGCGACTGCGATCATGCAAAATTTTGTGAATTTTTACAAGTATATTATATAACTT

ACATTGCACATTTAAATACATAGGCATAACTCCTTCTGTGGGATTTA-AATACATCCATT

GTAAAGCATCATGTTCAGGAACAGTGTTATATATTATCCAATCATATTTGGAATGTAAAT

TATGATAATTAAAAATATCTTTTAAAAGAAGTGTAATTGCCAAAGGTAAATTTTTAGTAT

ATGTATTTATAAATCTATTTAGTTGCATAGGTTGCATTTTTGGACTTAATATATGCATTT

TTGCTTGAATTTTTAAAGTAGCAATATTTCCACAATTATCTTTTCTAGGATTCATATTAT

GTAAAACTACTAAAACAGTATAACCAGTACATTTTGGAAATTTATCATGTAATTTTGAGG

GAAAAGCATGAAAAAATTTAGCTATTCCTTTAGTTGAGCCTAAATTTTCCATACATTCAT

CCATAATTATAGCTATTGGACCTTCTTTTGCTGCTTGAGCAAAC-ACATTATTAGGGTCA

CTAATATCATAATTATGATCTTGAATAAATTCATCATAAGACATTTTAATAAATTTTGGC

ATTAAAGTAGAACTTTGAGGTATAACTATTCCATCAGGTCCAGCTAAATAATTTCCTTCA

CATATTTGACTTTCCCATGCTGAAAATTCATGAGGGGGTATCATGTCAATTTGGGGCACA

ATAAAAAATATTGTTTCTGGTTGTGGATGAATTAATTGAGAAGATAGTAAATTTCGAAGT

AACTGGGATTTTCCACTACCAGTAGGACCATAAATAACCCCTATAAATGGTTGCATATTA

TAATTTAAAGATTTACATGTTTGATTTTTATGTAAA------------------TATTCT

TGTACACTAGTAAATTCTTTTTTAATAATTTTATTTTCTTCTAATAATTCATTTAGT---

-----------AGTTTTTCACCCCCAAAACT-----------------------------

TATTAAATCATCAAATG-AATTAAAAATATTTAAAGGTTTTAAAC----CTTCAGCCCA-

----AGACATAGATTGT-----AAAGTATTATTTAAAGTCTGTAATTTTT-------CCC

AAAGTTCTTTTAATTCT----CCCATGGAAGTTCCGTCC----------AACAAACTTCT

GTGTTTCTTG--------GATTTGGGTTGCTCGTAGAGTATGGTA--TCAGTAGATGTTG

ATTC---------------------ACTTGATGAAGAGTTTTGTCTTTCCATGGTCTTAA

AGTTCTTGTCAATGTTGATTGAGTTACCGTGAATGGTTGAACATTGTTCATTGATGTTAC

TAATGTTCTGCGTAGACTCAATCGGGATGTCTGAAAATGATC------------ATTTCC

TTGTTGAATATCAGCAAG---ATAACAAGCTAACATTGTT---TCATATGACAATTCAGT

TTTAGGATGACCTTTGGCTCTTAATTTACCTGGGCCTACA------TATTTACAAAATTT

ACAAGTAATATCTTTTAAAGCATATAATTTTGGCGCCA-TAAAAATTGATTCTGAACTAT

AAGCATCTTTACCACATTTTATACATTTAGTTTCACATTCTACAAGCCAAGTTAAATCTG

GATTTTTGGGATCAAAAACTAATTTACCCCCATTTTTTTTAATTCTATGCCTACCTTTTT

GCTCCATAATGCTTCTTCCAAGTTCCGTTAGAAATAAACTGTCTGTGTCTCCATATAAAG

ATTTTAATGGTCTTTTATCAATTG---GAATCCCTCGATCATTTTCATATAAAAAATCTG

ACCATTCGGATATAAAGGCTCTTGTCCAAGCAAGAACAAAAGAAGCAATATGTGAGGCGT

ATCTGTTATTAACAATAAGAGGGGAATTTAATTCCAAAGTATGTAAACATGCCTCATCTT

CATCACAATCTAGAAAGGTGATTGGTTTATA---GTGATAGGTCACGTGA----------

----TTTAT--------GACACTATAAATAGAGGTCTCGGTGTCAAGATTTTGATCATTG

AGTAGCTGTGGCTGCTCAGAATCAGC-----------TCTTTTTAA--------------

------------------------------------------------------------

-AGGTGAGTATAAAACTGTAAA---TTGGGGTATAATTTCTGCGCTGTAATTAT------

------------------CTGTTTC----------TATAAATGTAGAATTT--------T

TGATTTTATATGTTCCTTTGGCTATGTCTTTTTTACAAATGTCATCCATTTGATCAGAAA

AAACTGTTTTTTTATTATCTAATTTGGTAGCAAATGAGCCATATAAGGCATTTGATAATA

ATTTTGATATATTTCTTAAAGTTTGATTTTTTTCTTTATCTGCTTTTTCTTTGGCTGCTA

TATTTAGTTTTACATATTCAGCTGCTATACATTTCCATTCTGGAAAAATTGTACTTCTAT

CATCAGGTAAAATTTCTACCTTCCATCCTCTATTATGTAATGTAATTATATCAATAGTAG

TAGCAATTTCACCTTTTAAAGGTTCATTTGTCCAGCATAATCGACCTCCTTTTTTAGAAC

AAAAAGGAGGAAGTAAATCTAAGAAATGTTCAGGTGGGGGCGTTGCATCAATTGTAAGTA

TTCCAGGAAGTAAA-TTTTTATCAAAATATGAAATTGTTTTATATTTTTG--TAAATAGT

AAACAT----ATTTATTAATTTCAATATTTCTTTCAAATGTATTTAGGGGTTTTCCAGCT

GGGAATGGATGTGTTAGTGCACTGGCATACATTCCACATATATCATAAACATATACTGGT

TCTTCTAATATTCCTATATAATTTAAATAACATCGTCCTCCTCTTATACTACTTCTAACA

TATTCATACATTTCTTTTGAAGGAGCATATAGT------ATTTCATCTAAATTTGGACCT

AAAAGATTTTCAGATTTAT-----------------------------------------

----------------------------ATAAACATTGTTTAAAAATTGCATGTGAATTA

GAGCTAATAGTTGGTCTTTGAAAAATATTAAAATTTGAATTTGGTAAGTTTACTGCTTCT

GCTATAAAATTTTTATAAGATAATAGTAATTTGTTTACAAGTTCTGAAGTTACATTTACA

TCTAAAATACAATAATCTATTGTTTCTTGAACAATGTCATAACTA---------------

---------------------GGTTTTTTGGATTGTTTCCAAATTTGTTTATTTTCTTTA

TATTCTTCACTATTTTTCCAGTATTTACAAGCAGGAAAACCATC---TTCATCAGTT---

TGGTAGGTTCCTAACATATAAAATTCATTTACTGCGGTATATGGACAGCAGCCTTTTTTA

ATATTTAAATTATAAGCTTTAGCTGCATTTTCTAAACTGGT---ATGGGTTAAAGCGTAA

GTATCTCTAACCATAACTTTTAC-------------A-AATTGGTATTTTAAAT--CTTG

TGGGTTACAAATTCCTT--CCTGCCATATTTTATAAGTTTCTTTTGTAGGTTTAATATAT

TCT---GGATTTGGAAGAGCAAAGGTAACATCATTAAATAAAATTTTACCACATCTAGGC

ATGAAATTTCTTAATATTT---TAAATCCTTTAGGTACGGAATATTTATTAT-------T

AATAACTTGTGCTGCAAGAACAATTTCATCAAAACCTGTAATGTTATGTCCTACTATATA

AAATTCTATTA--AACGAGTTTTACCAGAAAGTG--------TTAGTTTTT---TAATTT

GATTAAAACTTAATTCTTCATAATCAGT-AATATTATTTAATTGCATAAAAGTATCTAAA

GAGTTTTTATTACTTTCAATAAATTCATTCCATAATAAATTAGTT---AATTTATGCTGC

ACAAGTTCTCTAAAGTTTTTAAATTTGTAACCTATGCTTCTTTTTTGAGGATTTATATAA

AAAAATAAGTTTTC------TGAAGAATTATATTGATCCCATTTTTGTTCCAAGGCTATT

TCAGTGGCAATTTTAACAAGGTTTGTATTACCTGATAATTTAAATACAATTAAAAAAGGT

ACTAACTGTTTTCCAAATTGTCCATGCCATGTATAAGTTTCAACATCATAAGTTACAAAT

AATTGTTCTGTTAAAGTACAACTTCCTATAGGGAAAAAAGATATACTTTCCCACCAGTCT

GCTGTTTGCACATTAATATTATGAAAATAATATTCTCGTCTTCGTAAATTACAAGTGTGA

AAATTTTTATAAATTCTACCACAATAATCACATTTTTGCATTGCATTAATTTCATTTATT

-AAAAATACTTTATTTTTGT-----AAATTAAAAATTGTAAAGGAAAA------------

------------------------TTTACAATAGGTTCATTAAAATTTATATTAGTTG--

----------------------------TTTTAAATTTTCCTTTAAAGTAAGAATATATT

TTTGCATTAGTAGGTTGACATTCTTCTATTTTGGTAAAAAT---------ATTACTAACA

TTTAAA------TGTGTTAAACAAAATGGTAATTCTTG---TAAATGAACATGAAATATA

TTTTGTAAAGCTTTTAAAGTATCATTAAAGTATTTTATTTCAATAATTTCTCCATCACTG

T---CTATTCCTTGTATGTAATTACAAGCTCTTCTTACTACTCTAGTACC----------

------------------------------------------------------------

CTTTAA-----------------TTTACTTATTTTTAAGGTGGAAGTTGTACAG---CCT

GATGTGCTTGGGGTAGTGGTAACCTTTGAGCTCGTAGTTGAGTTGCTAATAACACTACTT

TCCTGTTGATATTCTGAATTTCTCGATGTTGACTGAGAATCACTGGTCCCGTTAGTTTGA

ACCTAAAGGAAATTTCCATAGAATCAATATCAGCATCATTCA-TGGCTACTTGTTTTAAA

ATTTCTTCTATATCTCCACTATTATCATGATAAGCAATATCTGTCATTAGTTGATCAATT

TCTTCTTC-----TGGCACTTCT-CCCATTCCTGCTCGTTCTACAGACATTGCTAAATCA

TTACTTATTCTATTCATTACATTAATAAA------AGCATCCTGTCCATAATCTGTCCAA

AGTCTACTATAAATAACTTCTCCTACTGTATTTCTAGCTCTTAAAATAATTTGAGCTAAA

TTTAAATCCACAAATCTTGTAAATGGATTGGCCATTCTAAAAGCCTGATGAAGATAATTT

AAACTTGAGGCCACATGTTCAGTAACAAAAAAATATAACACCCATCTACGTAAAACAAGT

TCAGTAATTTCACCCAAAGCTTCTAAACGTTGCATGACTTGATAAAAATTTACAGTAAAA

TTAAAAAAATTTTGATTTCTAACTTGAACAGATAATTCTTCTTCTAATAAACGTATAACT

TCTGCAATAGCATTTCTAACTTCTTCTTCAAAT------------GTAATT------TCC

TGTTCCATTT------------CAGATTCATCCCCTTCTGGAGAAGGTGGA---------

-----GGAGG-AGTGACTGGGCGAGGCCT------TCTTCGACGAGTTGGAAGAGGTAAA

CTGTCAATAAATCTTTGAATCATTTCCCCTCTTCGCATTCTCATTTC---TTGAGTAACA

GCTCTTCCATTT---TCTCTAGGTCTTAATTCAA----AAGCTCCTCCTCTTAATT----

----------------GAAAGGGTGTATTA-------TTTGGTAAACTTAATGAACTAAT

TACACTTT-TTAATATATATTGTAGGGGCAATCCT--------GACTGTAATAAAGTTT-

---GAACATTAATTGGTTCATTAAATTGTT--CAATAAAAGCGTGAATCCAGTCACAATC

ACAAGGTAAG---CTAAGTTGCATGTCACT------------------------------

------------------------------------------ATTAACATTTAATAAATA

ATTAAAATAAGCACATTTTAACTGCCTAATTGTTTGTAATATTATAACATCACTATGGCC

TGCTTGCTGAATTCTTATTTTTT-CTGCCATTCCCCAAGCATTTGTTTCTCCCAT-----

---ATTTCTT--GTTTGTTGTTGTAAAAAAATATGCACAGGAACATTTTC----------

------------------------------------------------------------

-----TTCAGGAGAAATATGTGTAATACCATATCCTTGCATAGGTTGAATTAAACCTAAA

TCAGCAACTACTCTATCAGCTAAAATTGATTGTTGAATTTGAGATAAAGTTTCTTGAAAA

TT------------ATCTAAATCTAAAAATCTTTGATATGTACCAGT-ATTTAATGTATA

GGTACAATTACTAACCATGGACCAATTTAAAAG--TTGTTGCATTGGACCATGTATTTCA

TGA----TATTTAATTTTACTATAAGCTCGAGTGTCAAAG-ATGTAGTCATTACACACTC

TTATAACATATTGGTA-TCCAACTAAAAAATGGGGTGGTGGATATAAATATAATGGCCAT

CTTATTGTAGCTGGTTGACGTGGACTTAAATTCATTAACATTAATTGGGGATAATTA-TA

AATATAACGTGACATCCAAGTAAGGCC-TGCAGCTGTAGTTGAGGCTCGTGTCCATTCTT

GCACACGTCCCCAAAAATTTCTGATCGGTCTAAAAAGTTCCATGGTATAGATTGATTGAC

CAGTAAGGCGAGCGTAGTCCATGGCGTTCTGAA---------------------------

------------------------------------------------------------

------------------------------------------------------------

------------------------------------------------------------

------------------------------------------------------------

------------------------------------------------------------

------------------------------------------------------------

----------------------AGAAGAAATATGGCCTTT--------------------

------------------------------------------------------------

------------CAG-ATGCATCCAGTACTTCGCCAGATGAAAAACTTCAAA--------

----------------------------ACCACTGATG----------------------

-----------------------ACAGCGAAAATACCCAA--------------------

-------------------------ATGGAGGGTGAAGGTTTAG-CTAGATT------AC

ATATTCCACCTGATC--AACACCCTCGAGTACAACTAAAAAAAGATAGTAGTGAAGCTAC

TATTCCTAAAGTAAATTTATTTAGAGA---------TCGTCCTGGAGAAGAACCTGAGGA

AATGAGAGATTTAAAATATAAAGCTGGCAAATGTAT------TGAGTTAGATAAAGATA-

AATTGCTAACTGAAAAAGATTT--TGAAATTAATGAAGAAACTGGAG------TTAGTCC

TGCTAAAGCACATTTAGCTGCTGCTAATTTAGTTACTGCTTATGAACAAACTGTTAAAGA

AGAATTAAATTTTCAAAAATCTTTTAATAATAATTTAAGAACTTTACTAGCTAGAGAGGA

AGTTACTATTGGCTTAATGCATTTATGGGATTTTGTAGAAGCTTATATACATAATATAAA

T-------AGTAAGTGCTTAACAACCCAACTTTTCTTAATAGTTCAACATATAAA-AAAT

CAAGGTAT---TTTTAAAGATA--CCTTTTTAAATATTGCAGATAGT----GAATCTAAA

TGGCTTTATGATTTAATTAATATTTTACAGTCTATTATTGTGCAAGAAACTACTTTATCT

ATTTCTGAAAAAGTAGCAGCAATAAATTATTCTATTATTTCTTTGGGTAAATTTTATGCT

AGAAAAATTTTTCAATCACCATTTGTACCTTTAGATAAAGAAGTAAAAATTGACACTTTT

TATATGAGAATGGTTTTAAAAATTTTAG------TTTTAAGTGATGATTTAGGAATTTAT

AGAAATGAAAA--AATTGAAAAAATTGTTAGTAATTGTAGAAAT-----AAGGAATTAAA

TGATGATGATTTATTATTTAGTTTACGTAAAGC----TTTTT------------------

------------------------------------------------------------

------TAAATTCCAATC------------------------------------------

------AAAATTTTGAAGATCA------TTCAAATG--ATAACTCA-----------TTT

TTTAGTGAA--------------------------CATCATGCAACCTACC---------

--CGGAAG-----TTAATGCAGATGCAGGCTACGGAAA--------------AA------

---CAAGAATGGCCAAAGGCTTTTAAAAAAGTTTTGGGATTAACTTTTAA---TTCTGAT

---TTTACAAATCAACCAAAAGGAAAT-AGATTTAGTACTATTTTGGAAACATTTGTACC

TTCTCGTAAAAATCCTACTTATGAAAAAATTTTAAGTTTAGTGGATGCTTTAATTAAAGC

TAAAGCTATTAGAGCAGATGAAGGAGGGGAAATGTTTAATGCTTTACTAGAAAGAGTTAG

TAAATATAATAGTATAAACTTACAATCTAATTTAGATTATTTAGTTACTGATGTAA----

--AAGAAGCATTAGCTAAAAAAGAAAGAATAAAATCAGGTCAATTTTTAGGTTCTTTAAT

TGCTTTAAATAGTTTTCTTTCAACTTTGCCAGCAACTGTTTCTAAAGGACAAGATAGTTA

TTTAGCTTTTATTAGTAGTTTAAAAGTTTTGGTAAGTGAGGTACCTCA---AACTGATGT

TTATCAAGCAGGGCCTAATTTTTATTTGCAAACATCTAG-AA--ATGGTTCTCATACAGT

TAATTTAACTAAAGCATTTGAAAATTTAAAATCTTTATGGGGGGTTAAAGCTCCTACCAC

CTCAT-CAACTAGTATTACATCCTTA-----TTAACTCCAAATACACGGTTATTATTGTT

GTTGGTG-GCTCCATTTA-CAAATTCTACAACTATTCCAAGAGATACATATATTGGACAT

TTACTAACTTTGTATAGAGACACAATAGGACAAGCTAACATG---AATGAAACAACTTTT

AATGAAATTACTCAAATTAGTGAAGCAATTGGAAATGAAAATATTT---CCAATTTGCAG

TCTACATTAAACTTTTTATTGACTAATAAAAAGTCTAATTTACCTAGTGATTTTTCATTA

ACTGCTGAAGAAGAAAGAATTTTAAGATTTGTTCAACAGTCAGT-TTCTTTATATTTACA

ACAA---GGATTTGATG--CTACAGATGCTTTAGATCAAACTGTAGCTAATTTTGAACCT

TCATTTTATTCTAAAAATCGCAGCTTTATTAACAAATTAATGGACTACTTACATAGGGCT

GCAAGCATGTACCCTAATTATTTTACTAATGCAGTCTTAAATCCAAAATGGTTACCACCT

GAAGGATTTTATACCGGTGATTTTGATTTTCCTGAAGTTTCAGATGGA---CTGCTTTGG

AACGATTTTGACAGCAGTTATTT--------------------------CGGTCAGGAAC

AAAATCAAAAACTTCTTTCAGAAGACAGTAGACAGGGTGAGGCCTCTAGTAGCCG-TGAT

CATGCAACTGTTTCCAGATCTTCTCCCTACTCCTCAACGTTCTCCC----TCCCAGGAGC

CACAGCAGACCCCCAAATAAATA---------------------------------GTGT

AGATTAT-TATTCTTTACTAAATAATTCTAATGATAAAAAT---------------ATTG

AAAAAGAAATGGAATCTATAACTGAAAAATT-TGCTCGATGGAAAACTTA-TGCCCAAGA

ACGTAAAGA---------------------------------------------------

---------------------------------------------------AATGCAA--

---------GAAAAATTTAATGGAAGTGGTGGT-----------------AGATTTTTTT

CTGTAATTGGAAATAATTCTTGTTGGTAAT--ACATT----------------AATAAA-

----------------CTTACCAAAGCCATTATAATTT---GTGTCCTCCTACTTAATAT

GCCAGGACAA--------TGTTTCGATTGACT----------------------------

-----------ACACCTCCTCCTTCTTATGACTCCGTCTT--------------------

-----------------AAGTGATAATGCACTTCAAGATCCTTTTGTTCCTCCAAGATAC

AACGCT-CCGTCTGAGGGAAGAAATAGTATTCTTTATTCACAACTTCCTCCAGTTTATGA

TACCACTAATTTATATTTAATTGATAACAAATCTGCTGATATTGAATCTCTTAATTATCA

AAATGATAGAAGTAACTATCTTACTAGTGTTATTCAAAATAGTGATTTTAGTCCTATGGA

AGCAAGTACACAAACCATTAAATTAGATGAAAGATCACGATGGGGAGGACAATTTAAAAC

TAT--TTTACATATGGCTATTCCAAATATTAATGAATTTATGTATACTAATAATTTTAGA

GTAAAAGTTATGGC------TGATAAAACCAATATTA-----------------------

----------ATAGACCTATTTATAAATGGTTTGATTTAACTATACCAGAGGGTAATTTT

TCCTTAATAATGGTTTTAGAACTTATGAATAATGCAATAGTTGAAAATTATATGAAAGTA

GGTAGACAATATGGAGTTAAAGAGGAGGATATAGGTATTAAATTTGACACTAGAAATTTT

AAACTTGGATGGGATCCTGAAAAAAGATTAGTTGCTCCAGGTGTATATACTTATGAAGCT

TTTCATCCAGATATTGTTTTACTACCAAATTGTGCTGTAGATTTTACTAACAGTCGTTTA

AGTAACTTATTAGGGATTAGAAAAAGATTTCCATTTCAAAAAGGTTTTATAATTACTTAT

GAAGATTTAAGAGGAGGTAATATTCCAGCTTTAAGAGATGTAGAAAATGAT---------

--GGGCGAGGAGAT----------------------ATGCAAGGC---------------

---------------TGGACTGCTTTAA------------------AAAAAGA-------

------------------------------------------------------------

------------------------------------------------------------

-----------------TAAAAAAGGCAGAAGTTATCATGTAGGTGAA---GATCCAGAA

GCA---GATATAAATGATACAGCTTATAGGAGTTGGTATTTAGCTTATAATTATGGAGAT

CCAG---AG---GGAGTTAAAAGTTGGACTTTATTGGTTACACCTGATATTACTTGTGGT

GCTGAACAAATTTATTGGAGTCTTCCTAATCTAGCAACTGCACCAGTAAGTTTTAGAGAT

TCTC---ATAGTTCATCAAATTATCCTGTAATTGGAACTGAATTAATGCCACTTCATGCA

AGAAGTTATTATAATCCTCAAGCTGTCTATTCTCAATTAGTAAGAGAAAGAAC-AGCTCA

--AACTCATGTATTTAATAGATTTCCAAATAATCAGATTCTTATTCGACCACC-AGCACC

TACTATAACCACCATTACTGAAAACGTGCCAGCGATAACAGATCATGGAACCATACCATT

AAAGAACAGCATTTCTGGTGTTCAACGAGTAACTTTAACCGATGCTAGAAGAAGACTTTG

CCCATACGTAACCAAAAGTGTTGGCATAATTACACCTAAAGTTTTATCAAGTCGAACCAT

TT-AATC-----------------------------------------------------

---------ATGGCTATTCTTATATCTCCTGCTAATA-ATACAGGATGGGGGATGGGTTC

TCGTAAAATGTATGGAGGCTTAATTGGAGGAGCAAGAGAGTATAGTGAA--GAAACTCCA

GTGTTAGTAAGACAGTATTATCGAGCTCGTTGGGGTAGTAAAACAAGACA----------

-----------------------------TGGTAGACCTGTAGTTC-------------A

CAGGCCTAGAATAGAAGTTGAAGCAGAG---------GTAGAGGGA---GAACCAAGA--

------------CGATC-------------------------------------------

------------------------------------------------------------

-------TGCACGG-----------------------------TTAAGAAAAA-------

---AA------AGACCATTGAGT------------------ACTAAAA---TT--ATAGT

AAGTAAGGGCAAACGAAGAAAAAGAAGATA---------------------AATTATAT-

--TT--------------GTATCTGTG--CAGGTA--------------ATTGTT-----

-----------TTAAATAAATCTC-------------------------CATTTGA----

----------------CTTGCACAGAC----------TGGAGT-----------------

GGATAATTTACT-ATGAC-----------------------------TTCTCGCCAGC--

-------TTAAAGAAGAACCGTATCAA---------CCTTCAGTTA--------------

CTGAAGTTCATGAGT----------TGCCC---------AGGAA----------------

TAAAAGAAAAA----------------GGGCTTAT-------------------------

--AAAGATGA---------------------AAAGGATGAACCTATTATAAAA---GAAA

TTAAA---------AAAGAATTTAAAATAAAT---------ATTCCTCCTCAA-------

-----------------------------------CCTAAACGACAATATCGATGGAGAG

GAAGAAAAGTTCAAAAAATTCTAAGACCTGGAACTGCTTTAGTTTATTCTTCAGTTCCAA

AACT---AAAT------AGAAAAAGAAATGCAGAAGAAATATTTACAGATACTGATATAT

TAGAACAAGCTAAAAATAGGGAAGGGGAATTTGCTTATGGAAAAATGC------------

------------CTTTATTAGAAATGGTAACAC--CTAATTTAAAAAGAAAAAGAGGTTT

TAG------------------TGAAGAGATTACTGA-TGATTTTATAACTACTCAATTAA

AAAAACAAAAAATTGAACAATTACA---GAAAAATTTAAAAAGAAAAAAAG-----AATC

AAAT-GAATATTTAAATAAGAAAAGAAAAATAG----AAGATTCTTTTGTAGCTTTGGAT

AATTATAACCCCACTCCAAACTTAGAGCCAG-----------------TAACTGAGCAAC

AAATTATACCTATTAATAGAAAGAGAGGTAGAGAACATTTGCAGCCAACTGGTCAATTAT

TAACTTGTAAAAAAAGAAAAACTTCCCAGAGAGCTGATAGATGTGAAGCAAATATGGAAG

TTGCTCTACCAATAGAAACT------------------TCTACAGGTTCTGAATTAGTA-

AAAGCTGACATAAAAGTTCGTCCTGTAAAAC---CAGTAGCTCCAGGGCTTGGAATTAGA

ACTGTAGATGTAGATATT-------------CCAGTTAATA-----ATA-----------

-------CT--------------TCTGATAATGT-----------TATTTCTGAATCT--

-----------TTGCAATCTACTTTGGTTGCTCCCATTCAGGATAAAAACAGAAA-----

-----------------------GAAGATGAT--TCCTGAAGTAA-------AATATCAT

CCTTCTATA---A----------CAGTTACAA---CAAAAA----AGAAAAAGTATCCTT

CTGCAA----------------------------ATTCTATTATTCCTGAAGCTGTCTAT

CATCCT------TCCATTAAA-CATCCTCGAGCTA--AAAAAAGAATTATTCC---TGAA

GTTAGATATCATCCTAGTATTACAATTCAACGTCGTAATACTATTTAATAGTGTTTTTGT

ATGTTAATTTTTATCACCTGTAATTCATAGGCTTAAAAAATGACAAAATCTTCAATGGTC

ACCTATCGATTGCGAATACCTGTTTCTACACGAAAAAGAAAAAAAGGACG----------

---------TCATCGTTCTACTCATATAAAA----AATCTTAAGGGAGGGTTTTTACCTC

TCCTTGCCCCAATTTTGGCAGCTGCCATTGGAGCTATTCCAGGCATTGCCTCTGTCGCTC

TAAATGCTTCTCGGAATTAAAATTTG----------------------------------

----------------------TGGG-------TAATTTTTTTTTA--------------

----------CCAGAAA-T-----------------------------------------

--ATGGAAGAAATCAATTTTGCTGCTCTTGCCCCGCGACAAGGCACTAAACCTTTGATTG

GCGCTTGGTCAAATATTGGAACCAGTGAAATGAATGGCGGCGCCTTTAACTGGGGAAGTT

TGTTAAGTGGTTT---------------AAAAGTATTTGGCAATGCAGTCAAAGATTTAG

GAAGAAAAGCTTTAAACAGTAGTACAGGGCAGGCCTTGAAACAAAAATTAAAAGATACAA

ATTTACAAGAAAAAATAGTAGAAGGAATAAGTTCAGGTATTCATGGAGCTGTTGATATAG

CTAATCAAGAAATTCAAAAAACA------------TTAGCAA--------AACGTCTTGA

TC-----------------------------------------------CAGTACATACA

ACACC-----------------ATCTCCCTTGGATGTTTTAGAAGAAACA----------

GAAGAAAAGG-------ATTTCAGTCATGTTTCACTCCCACCTATTACTAAAAAAAGAAA

GGTGGAGGAA--------AGTTATGAT---CCTC--CAT-------------CTTATAAT

GA---------------------------------AATTTATGGCAAAGAGGTTGTTTTG

C---------------CTTCT------------ACCAATGTTAACACTCCA---------

------------------------------------------------------AAAACA

TATTTTCCTC-------------CTTC--ACAGC-------GCAATTGGCAAACTACTTT

AAATAATATTGTGGGCGTGGGAGTTAATTTTAGTAAAAAAAGACGGTGTTATTAAGA---

---AT-------------------TTTTTGAAA----A-TTTTTTTACAAAAATCAGC--

-----------------TGAAG-----------------------------------ATG

GCCACTCCTTCGATGATGCCCCAGTGGGCCTACATGCACATCGCCGGTCAGGACGCTTCT

GAATACCTTTCACCTGGCCTAGTACAGTTTGCACAAGCAAC-AGAAAGTTATTTTAATCT

TGGCAGCAAATTTAGAAATCCCATGGTGGCTCCTACCCATGATGTAACCACAGAAAGATC

TCAGAGACTCCAACTCAGATTTGTACCAGTGGACAAGGAAGATACTCAATATGCTTATAA

AACTCGATTTCAATTGGCTGTAGGTGATAATCGAGTTTTAGACATGGCAAGTTCTTATTT

TGACATTAGAGGTGTATTAGACAGAGGGCCTACATTTAAACCTTATAGTGGAACGGCCTA

TAATACTCTAGCCCCTAAAAGTGCCACTAACAATATTC-------------------AAT

ATGATGGCAAAAATA---------------------------------------------

--ATGAAAATAA------------------TGTTATTAAAACTTTTGCTCAAGCTCCATT

TCCTGGAAAATTGGATAATAATAAAAATTTTGTAGTAGGAACAGCA--------------

-----GCAAATAATGCAGATATAATTGCAGAAACAAATTATCAACCAGAGCCTCAATATG

GGCAAGAAAGTTGGAC---TCAAACA---ACTGGTAATAACATTGTTTTTGGGGGTAGAG

TTTTAAGTAAAGATACACC---TCATTTGCCATGTTATGGTTCATATGCTCCTCCTACTA

ATATTAATGGAGGACAAAATA------------------CAAATGG--------------

----------------AACTTCCTATGTTTATTTT---------------AAAAATGGTG

CAAATGCTGCTGCCC------CAGATAGTGCTCTAGTATTGGAAACTGTAAATATGCAAA

CACCAGATACACATTTAATTTACAAAATTAATGAAGATGAGGAAAC------TCACCTCC

CCAGTCTAGGGCAACAAAGTGCTCCAAATAGGCCAAATTATATTGCTTTTAGAGATAATT

TTATAGGTTTGATGTATTATAATAGTAATGGTAACTTGGGAGTTTTAGCTGGTCAATCTT

CTCAACTAAATGCTGTAGTTGATTTGCAAGATAGAAATACAGAACTTTCATATCAATTAA

TGTTGGCAAATACTACAGATAGATCTAGATATTTTAGTCTATGGAATCAAGCAGTAGATG

ATTATGATCCAGATGTTAGAGTTATTTCTAATCATGGAGTGGAAGATGAAATGCCTAACT

ATTGTTTTCCACTAGCTGGTATGGATTTCAATGTAGCTACTGCAGTAAAAAATGATGGTA

G---------TGCTGA------------------AAAAACAAATAATCAAGATATTGG--

--AACTGTAAATGTAGGGTTGGGAAATGTAAGTTGTATGGAAATTAATTTGGCAGCAAAT

TTGTGGAGAGGATTTTTGTATTCTAATATAGCTTTGTATGCACCAGATGAATATAAACAA

-------ACTCCACCTAATATTGAATTG--CCACCTAATAAGGA---TTTGT---ATGCT

TATATGAATGGAAGAATTCCTATCACAAATCTTATTGATAGCTATGTTAATATTGGTTCA

AAATGGTCTCCAGATATTA-TGGATAATGTAAATCCATTTAATCATCACAGAAATCCTGG

TTTAAGATACAGATCCCAACTTTTAGGAAATGGAAGAATTTGTGAATTTCATATTCAAGT

TCCTCAAAAATTTTTTGCTATAAAAAATTTACTTCTTTTGCCTGGTACTTATACTTATGA

ATGGGCCTTTCGAAAAGATGTTAATATGATTTTGCAAAGTACATTAGGAAATGATTTAAG

AGCAGATGGGGCAACTATAACCATCAATAGTGTAAATTTGTATGCAAGTTTTTTCCCAAT

GTCCCATAATACTGCTTCTACTTTAGAAGCAATGTTAAGAAATGAAACAAATGATCAAAC

ATTTAATGATTATTTGTCTTCTGCTAATATGCTTTATCCCATTCCTCCTAAGGCTACTCA

ACTTCCTATTTCAATTCCATCTAGAAATTGGGCTGCTTTTAGAGGATGGAGTTTTACTAG

AATAAAACAAAAAGAAACTCCAGCCTTAGGTTCTCCATTTGATCCTTATTTCACTTATTC

TGGAAGTATTCCCTATTTAGATGGAACATTTTATTTGAACCATACCTTTAGAAGAGTTGC

AATTCAATTTGATTCTTCTGTAAGTTGGCCAGGAAATGATAGACTTTTGACACCTAATGA

ATTTGAAATTAAAAGAACAGTT------GATGGAGAAGGTTATACCACTGCTCAAAGCAC

TATTACTAAAGACTGGTTTTTAGTGCAAATGTTGGCTAATTATAATATTGGTTATCAAGG

TTATTATTTACCTGATGAATACAAAGAAAAAAGTTATTCCTTTTTGAAAAATTTTACTCC

TATGTGTAGACAAGTAGTAGAT---AC----CAATT--ATAG-AAATTATAAA--GGGGT

AATTTTAAGTCACCAA-----------------------CAT-------AATAATTCTGG

TTTTGTTGGTGCAGTAGC---TGCTACTGCTCCTAGAGAG----GGACATCCATATCCTG

CAAATTGGCCTTATCCATTGATAGGCCCAAATGCAGTT---------CCAATGAAAACAG

AGAAA--AAATTTCTTTGTGATAAAACTTTGTGGAGAATACCATTTTCTTCCAATTTTAT

GAATATGGGAACACTAACAGATTTAGGTCAAAATTTACTTTATTCTAATGCAGCACATG-

CTTTAGATATGACTTTTGAACTTGATCCTATGGACGAAACTACTTATTTATATATTTTGT

TTGAAGTTTTTGATGTATGTCGCATCCATCAACCCCATAGAGGTGTAATTGAAGCAGTGT

ACCTGAGAACTCCGTTTTCTGCAGGAAACGCTACAACTTAAGTAATATT-TTAAAG----

--------------------------TTTTATATTTT----GAAGATTTATAT---TTTG

AAGATTTATAGAGATATTAATTGTTTA-TGCTAGCTTTTAATATCTTTTTAGAAAGGCTC

CTATTGTTTTAATTTGTAAAAACTTATTTTCTGAAAACATGGGTTCTTCAGAAACTGAGT

TAAAACATATTATAACTGATTTAGGAGTGGGAAAATATTTCCTTGGAACGTTTGACAAAA

GGTTTCCCGGTTTTATTA-ATAAAGATAAGCCTTGTTGTGCAATTGTAAACACGGCTTTT

CGAGAAACTGGGGGAATGCATTGGATAGCATTTGCTTGGTATCCACCAAGTTTTACATTT

TATATGTTTGATCCTTTTGGTTTTTCTGATGAAAAATTAAAACAAATTTATAATTTTGAA

TATAAAAGTCTTTTAAAAAGAAGTGCAATAACATCAGGAATAAATAAATGTGTTACATTT

ATTAAAAGTACAGAAAGTGTTCAAGGAGGTCATAGTGCGGCCTGTGGTTTATTTTGTTGT

GCTTTTTTATATTCTTTTGTTAATTATCCTTTAAATCCTATGAA---AAATAAATTTATG

GATATTTTTAAAAGTGTACCAAATAATAAAATTTTAGAACCCCAGTGT-CAGT-ATATTT

TTAAAAAAAATCAAGAAAATTTATATACCTTTTTATTATTAAATTCCCCTTATTTTAAAT

CCCATGAAAATCAAATAAAATGCCAAACAAAATTTGATAAA-TTGAA-------------

-TATGTAAAATTT---------------------TATTAATAAAATTGATTTT------A

CAATT----------------------------------------AAAACAAACTTTCGT

CCTCGTCTTCT-----------TCACCCATG----------------GGTAAAATGGTGG

TTTGGTATTGATA-----TTGGGGAAGCCATTTAAAATTTTGAATTCTAATGGGAGCA--

----TGACGTTTATACACATTAA---TCCAA-----ATTTGTTTAGCAATTTGCAAAGCA

GTAACAACATCAGGAGCACTTATTTTAAAATCACAATTTTTTTGAGGGTTTGCCCGAG--

-----TACTTTTAAACAC------AGGG--TTGCAACATTGAAAAACAAGCAATACAGGA

TGATCCACTGTAGC---CATCAATTTAGGATCATCAATCAAAGATTTATCCATAGTAGCC

ACAG--CAGA----AACAGTAAAAGGG---GTAATTTTAC----------AAGTTT---G

TCTGCCTAAA---------------------AGAGGCAAATTAGTG---GCCCAATTA--

CAATCA-----CATTTAATTGGCAT-AAGCATCTTTTTATTG------GCATCTTTCATT

TTTGGATAAGCTGCTTGCTGAAAA-------GCCATAATTTGCTTAAATCCTTCTCTGGC

TTTTTTACC----TTCAGTAAAAAACATTCCACAAGATTTTGAACA-------ATGATTA

CCAGCTGGTACATT---------GATATCATTAGCGCAGCAAGCAGCATCTTCATTT---

-TTTATTTCCA--CTACATTTCTA--------CCCCATTTGCTGGTAGTAAC----TTTA

GCATTTGCTTGATTTTCTT----TTAATGCTTTTTGAGCA-----TTTTCACTAC-----

----TTATATCCAATTGAACAATTTGTTCTTTTGTAAT--------------CATGGATA

ATCCATGAAGACATTTTAA---ATCAGAATC---------------ATCACATCCATGTT

----------CCCAAA----TTACACAT-----CCAGTAGGGTCCCATC-----------

-CAGGCCCGTCCAAATCACAAGAACTTAATATAAATTTAAACAAAAATCTTCCAATCATA

GTT----AACATT----------------------GTTTTTTGAGTAGTGTAA--GACAA

TTGCAA-----------ATGTTGTTTTTTTTCATTAAGATAAGCTTGCACCCCTTTTCTA

AAACATTCTAATGTACCCGCGTC------AGCCAAAAGGGT-------------------

---------AAGATTTCTTATATCCACTT----TAAAAGGA--ACTAAAAATTCTACAGC

TCTATCCAT---AGCATGCTGCCACA---------------TCA----------------

----------------------------TTTCATCACTAAAAGCCTCAGCCGAATCA---

-----------------------------------------------------------G

GCCTAACATTTTCAAGTCT------------GATTCGTTTAGGCATTTTAGATGATTTG-

--TTTTTAGGATTTGCTTTTTCTG-TTGTAAGTGGGCCTTCATTTTCCCCATCAGATTCA

CTGTCA---GATATTTGAATAGCAGGACGACGACGTCCAG--------------GGCCTC

TTTTT-------------------------------------------------------

-------------------------------------------ATTT-------------

CCGAGCGAGTTTGTAGTTTGATAATTTGAAG-----------------------------

------------------------------------------------------------

---------------------TTCTTCTTCAC--------------------TACTTTCT

GAAATCT------------------CAGCCCGGTTATTTGCGCT----------------

-----CATTTTT------------------CCTAGATGACAGAAGAGCAAATCCTTCAGT

CAGAAGGGG--------------------------------------------------T

TCAAGAAATGAAGA-------------------------ATGA--AACTAAATCA-----

----------ACATTTC-------------------------------------AGGATG

ATGAA-------------------------------------------------------

------------------------------------------------------------

------------------------------------------------------------

-------------------------------------------------CAT--------

------------------------------------------------------------

------------------------------------------------------------

GATGAAA-----------------------------------------CAGATTATTTAA

CTGAGGATTGTT------TACTAAAACATATTAAAAGACAAAGTTTAATTTTGTCTAAAA

GTTTAAATATAA---ATAA-CTGTCCTGAT-TCTATCAAAGATTTAAGTAATTTATAT--

GAACTAACTTTATTTTCT----------AAAGATATACCTAAA-----AAATTAGAAAAT

GGAACTTGTGCTCCTAATCCAAATATAAATTTTTATCCTTGCTTTATTGTTCCAGAAGTA

TTAGCTACATATCATATTTTTTTTCAAAATGCA-AAAATACCTTTGTCATGTAAAGCAAA

TAGAAGTAAAGCAGATCAAATTTTACTACTTAAAAAAAATGATACTTTACCTACTTATTT

GACTTTGGAAGATGTTCCAAAAATCTTTGAAGGTCTAGGACAGGAGGAGGTGATTGCTCC

AAAAG---CTCTGCA------AGAACAAAAT---------------AGTGTTTTAGTTGA

ATTAAAAGGTGATAACCCTCGTTTAGCTGTGGTAAAAAGGAATATAG---AAGTAACTCA

TTTTGCTTTTCCTGCTTTAAATTTACCCCCTAAAGTCATGAATGTTGTAATGGAAACATT

G---------ATATTAAAAACTACACAACCACAAAC---------AGATTTTAATGAACA

A---------AATGAAGAAACATTGGCTGTTACTAATGAAGAGTTGGCAAAATGGCTTAA

TATTTCTTTACAAGA---TAATATAAAAT------------TGGAAGAAAAAAGAAAAAC

TATGATGTCTAGTGTGTTAGTAACTGTAGCATTAGAATGTATGCAACAATTTTTTACAGA

TAAAAATGTATTTAAAAAAATAGAAGAAAATCTACATTACATGTTTAGACATGGATATGT

TAAACAAGCATGTGAAATTTCAAAAATTGAATTACCAAATATTATTTCATATATGGGAAT

TTTACATGAAAACAGATTAGGACAAAG-TGTTTTACATAATAGTTTAAAAAATGAAGCTA

GAAGAGATTATATAAGAGATACTATATATTTATACTTAGTTTATACATGGCAAACTGCTA

TGGGAGTTTGGCAACAATGTTTGGAAAAGGATAATTTAAAATTTTTAGAAAAAATTTTAT

TAAAACAAAAAAAATACCTTTGGACCCATTTTAATGAAGCAACTACCAGCATT-TGTTTA

TCTAAAATAATT-TTTCC--AGAAAAATTAGCTTCTACATTAAAAAATGGTTTACCA-GA

TTTTACAAGTCAAAGTATGATGCAAAATTTTAGAAGTTTTATTTTAGAAAGATCTGGTAT

TTTACCAGCAGTTTGTAATGCTTTTCCTACAGATTTTATTCCAATTGAATATAAAGAATG

TCCACCTCCTTTGTGGGGTCATACATATTTACTAAAATTAGCAAACTATTTTATGTTTCA

TACAGATATTGCT------TATGATTTAACAGGAGATG------GT---TTAATGGCCTG

TTA---TTGTAGATGTAATCTTTGCTCCCCACATAGATCTTTAATTTTTAATAATGCTTT

GTTAAATGAAACACAAACTATTGGAACTTTTGAAATTCAAGGTCCTCCAAAAGAAG--AT

GGCAGTCAATCACTGTC----TCTTAAATTAACTCCCGGTTTATGGACTTCAGCTTATTT

AAGAAAATTTGTAGAAGAGGATTATTATCCCACTAAAATTTCATTTTTTGAAGACC---A

ATCAAAAAAGCCACAAAAAGAATTATCTGCTTGTGTCATCACTCAATCAAACATAATTAC

CCAATTACAAGAAATTAAAAAAGCCCGCGAGGAATTTCTCCTTAAAAAAGGACATGGGCT

TTATTTAGACCCTATTACAGGCGAGGAACTTAACGGTGTAGGCTCATCT-----------

-------------------------------------------------ACACATCATAA

TGCCTTGCAAAAC-----------------------------------AGGCAAAAATCT

GACAAATTATCAAACTATTCAA--------CAACTATCAGAAACAA---TG---------

------------------------------------------GAGACACCAGAATCAGAT

CCAGAAACCAG-------------TCAGGCAAGTTTGGAAGAGGGGGAGATTCCAGAATC

ACCTCCAATTCCAGCACCGCCGGCT-----------------------------------

-----------------------------------------------------GTGAGCA

AAAAGAAG-------------------------------------------AGTAGATGG

GACATAAAACACACCGTTCCTAG----CGGTAAG--------------------------

-----------AAAGCTTATAAATCCTGGAGGGTACATAAAGCTAAAATTTTGGATTGCT

TACATAATAGTAAAGGCAATTTACGTTTTACCCGTCGTTATATGTTAATGCATAACGGGG

TATTAGTACCAGCCAA-TGTTATTCATTACTATGC--AACTTCTTACTACA---------

-----------------------------------------GAAGCC-------------

--------AAGAAAGCAACACCCAAAGCC-------------------------------

--GCTAACCTAT--------------CTTCTACCGCTGCCGCTGATAAACTCAGAGATTC

TATCTTTCCAACTCTCTATGCAATTTTTCAACAAAGTAGAGGACAAGAGAAAGGT-----

----TTGAAAATAAAGAACCGTTCGCTTAGATCCCTAACACGCA-GTTGTTTTTACCATA

AAAATGAAGCTCAG-CTCCTGAGGACCCGCCGCGACGCAGAGTTGCTTCTCAGAAAGTAC

TGTG----------CTGCTAACAAAACAGCTT-----------------------ATAAA

TAG------------------------------------------------GCATGAGCA

AAGAAA-TACCAACCCCCTACGTATGGACTTTTCAACCACAGATGGGAGTGGCAGCTGGA

GCATCTCAGGATTA---TTCTACTCAGATGAATTGGCTAAGTGCCGGAAATAACATGTCT

CAAAAAGTATTTGCTTTACGTAATCAAAGGAATAACATTTTGGAAAAACAAGCTATAATT

ACCGAGACTCCCAGGCTCCTACCAAATCCCCCGAGATGGCCTGCC--AGGAAAATAGATC

ACTCCCTAAAAATTCCCACT-CTTGTAAATCTCCCACGAAATCAGTTTTTAGAGACTTAC

ATGAGTAACTCTGGAA--TGCAGTTGGCGGGAGGAGGA----------------------

------------------ATA---------------------------ACTGGCGGTTCC

ATACAATTAAAC---AACGAA--TATGTGGG-AATGCCTATC----------CGTTCAGA

TGGGGTCATGCAAT--TAGCTGGAGGGGGTAGTCATTCATTAACTCCCCTAAATG---CT

TATCTACTTTTACAATCTGCTTCTTCTATGCCTCGCAATGGAGGTATTGGTTCTAATCAG

TTTGTA-AAAGAATTTGTTCCTACTGTTTATTATA-ATCCCTATTCAAGCTCTCCTGCTT

CTTTTCCTGATC---AGTTTA------TTTCTAATTATGATGTTATTTCAGACTCTGTGG

CAGAATATAGTTAAAGA-ATGGCTGAATTGGAAATTGATG---------------GAAAT

TTTACTGAACAACAACTTCTTTATAGAAGAA-----------ACAACGAAGCAAAATACA

ATGAAGAATTAAAAAGTC-------TTATTTTAATTCACAATTGTAAAAAAGGCCTATTT

TGTCCTGTTAAAGTTTGT----AAATTATTATTTG-AAAAAAAATATCAAGAACATAATC

TAAAATTTGAATTAGCTCCTGATATTACTAATCTTTGTCAAAGTTTTCATTTAGGTAGCT

CTATTAAAAT-AAATATAGTTAATCATGGGTATAAGGGTTATATAAGTTGCTCCTGTAAT

T---------CTCAAGATTGTATGCCTACTTTGTTAAAAACTTTATGTAACATTAATT--

-------ATATTA--AATAAAAAA----CACTTACCTTAATAAATTTATTAGTGTATTCT

TTTTTAAAGCTCCAAATGCTTCTATAT----AATCCCCTTCTTCCCAACTTTGATACTGA

-----------ATTTTATATTTAAAAGCAACTTTTCTAAATTTTTTAAAATTAATTTTT-

--TCTTTTAGTTTAATTCCATTATAAATCAAAGTCATCATGAAAAGAAAAGAACCTAGTT

CTCCTACAGAACCTGAAAGCCCTGTCTTCGATGCCGTTTATCCT---TATGAAGTGAAAC

GTATTAATTTAATGCCTCCTTTTTATGACTTC------------------AATGGCTTTA

CTGAAGAAA------------ATAATGGAACTTTATCTCTTAAAGTATCCAATCCTTTTT

CTTTTGATAATAAAGGAAATTTAGAATTAAATTTGGGGAACGGATTACGTGTAGATGAGG

AAGGTTTATTAGAATCTGATGCAAAGTTTTCTGCTTTAGCTAC--------ACAAGCTCC

TTTACATTATTCTTCAAAAAATATTTTACAACTTTTATATTCAGAAAATGAAGGTTTAAA

AATTAATAATAATAATGAACTAATGATAAAAATTTCACCTCCATTA-ACTTTTATAGATG

GTTCTATAAATTTATTATTAAATTCCTCTATACTAAGTATTTCTGATAATAAGTTAACTT

TTAAAATTAATGACCC-TTTTACAATTTCTGACACAGGCCTAGGTATAAATT--TAGGAA

CTGGTTTACTTTTACAAAATAATTCTCTTAATGTTCAATTACCTGATGGAGAGCCGCCAA

TTGTATCTGATTC-------AAAAAAAATACAATTAAATTATAGTAATGGATTAATAGTT

AAAGAAGGAGTTCTTCAACTAAATATTTCTGATCCA--TTTAGTATTAGTAA-------G

AATGTTTTAAATTTAAATATTGGTTCTGGTTTACAAATTAATAACAAAGAATTGCAATTA

AAAATTAGTAATGGCCTTCAATTTATTAA-----TGATAACTTACAACTTAATTTTAAT-

---GAAAATTCAG-CTTTGGAATTTATTAATAATGGCATACAAA---TAAAAGTAGGTGA

TGGGTTGCAAATAATAAATAATAAATTAACTCTTAAAATTGGAAATGGATTAGTTATTGA

TAATGATAATCAGGCT---TTAACCTTAGCTGCTTTTAATTTTATTGTTAATTCCCCATT

AAAATATGAAAATAAGACTTTATCTTTACTATTGGGACATGT-TTTTTCTATTTCTTCTA

CAGGAAAT-TTAGAATTAAAAATTGGGAAGGGATTAA----------TAATAAATAAT--

GTTACAAATGCTTT----------------------------------------------

-----------------------------AGAACTAAAATTA---------GGAAAAGGG

TTACAACTTTTACCAACTGGG-------------------GAATTACAGCTTAAATT--A

GGCCCTGGTTTAGGTTTTACAGCAGCTGGAGAAATTACACCCAGAATTGCTTTAAAATTA

GCTCAAGGGCTAG--AACAACTTCCTAGTAGTGCTTTACAAGTAAAAGTAGGATCTGGTT

---TGCATTTTTCACCTGAGGGAAATATAATTCCAAATTTAGGT----------AATGGG

TTGGAGTTATC--------TTCTGATCATAAAATTCAAA-----------TTAAAGTAAA

TGAAGGTCTTACTAATG-ATGA-ACATGGATTAAA-----------AATTACTAATACAC

C----TTGTATTTGGTGGACTG---------GAACCAATATGGAAAATAAT--ACTTTTG

AAAAACAAGGTAGAGT--ATTTTTATGTTTAAGTTCTTTTAACACTA--------TAGTT

ACTGGT-TTAGTTCAAATTTACTTTCCA--AT---------TAAAATAGTAAATGAAACT

T---TACTTTTTCGATTATTTTTTGA----------TAAAAATGGA--------------

-----CAACTACTTGAAATTAGTGATCTTACTGAAAATGA--------TTTAATAAATAA

AAATGTAAGTTTA--------------------CCTTTAAAT---AGAATGAAATTTATG

CCTAATAAAATTTTATATC-AAAAAGATGTAACTGAAGATGCTGAG-----GAAAATTTA

AATTTACAAGGAATGAAATATATGAATATACCTA---CTTATTTTAATTCTAAAGAAAAA

AA-TGATGTACTGGGA--CATTTACGTGTTTATTTTAATCAGGAAATGTCTAGTGTTTAC

TCTCTTGTTTTTAGTTGGGGCCCCATTG-C--AGCAACAGAATTTGCTGATGAATTTATT

TTTACCTCAATGTGTCA--------ATTTACATATATTTCTGAAGA---ATAATTT-TAT

TTTCCCCCCAATTATAAAACT------------------CACACTTCAAATTTCTGTTAA

TTATATTTTATTAATAATTGCTGT-ATCGAAAATTACGAAATTTTTTG------------

----------------------TTATAATT------CATTAAATCAGT-----AAAGTGT

TTTATGCGA-----AATTTTTCTCTTTTACTTGG--GTACAAAGGC-CAACGTTGTGAAC

GGATTGCTTTTACACA-----------------------------TTTCAGTAATAATTT

TCTAAT---------GCGTCTCCTACAGCAAGCGGCAGCTATATCAGTTAAATTTTTACA

AGAAATACATTTGATTACAAGC--ATATTATGATTTGTTTTTATA-TAACAAGCATCACC

AAAGGAAAC------TACTTTTATACAGTATCTTAATTCTCTTAAAAATTTAAATCTAAT

GTAGA------------------TTAAATGAAACTTTCTAAACATCACACTACCGATGTA

---AATAAAATTGCCAGGCATTTTGTAATTAACATATGGTCTGTAATC-----TTTAAAC

ATTATATTATAGTTACAACCGTACATAACTTCTACATGTAAGTAATACATAATTTTTTTA

CCAGCT--AAACATTGTAAAGA----------GTAAGCATTATAACAATGACAATGAACA

TCCCATTCTTCATAACCAAAAA-TGTAATAGCAGC-TACCCGGCAATGTAATTG-AGGAA

AAACAAAATAATCCTAATTG--TTTTAAAATCCTATAAGCCCCTCCCTGAAATAATAAGT

CCCATGGAATAACTAACTCACG-TAAAGGAATA-----AAGAAACTGCAGCCTGGGTTAC

TTCTTACACGACCCACGTGATGGGAATCAGTCATTAAGCAATGTGTAACTTTCGTGGTAG

GCATCTAAA------------AATTAAAATAATATTTTTATATTAAAGCTGTTCTACGAC

GACGACGTCCAAAAACAAATTCA-GCCCTAATTTCTAAAAATTCTAACTTCCAAAATAAA

TGCATTACTTCTTTTTCAATCTT-------------AAACCATTTTTTTAAATATTTATG

AATTTTTTTGTTACATTTAAGTGGCTGACGCC-----------------AATTTTCATTT

ATATATTCCCATAAAGTAAAATAAATTTTTATAGTATATTTTACTAACTTTCTTTCTGAC

CCAATTCTAAAATAATATTTTTTAAACAAACTA--TACACAGGATCCAATAGTCG-AGCC

ACTTCTTTTATTGTATCTTCAGTAAAATGTTTATT-------------------------

----------------CATGACTCAT-----CCAATTTTCCAAAGGGGAAACAGTTA---

-------ATTGAGAACAGTC---------AATGTCCAGACCTTTTTCTCTTGCCAATTCG

CGGTAATG-AAAGCGAAGATTTTTTTCAAAAATGCTTGCAAAGTCCTTTAATTCAGTCCA

ACAATGTCCGTTATGTT-CTTTCCCTGCTAC--AAATA-AAGTAAATTGCAAATAATCAG

CAGAAGGTAAAGCTCCAACATACATATGAT------ATCCATAACAATATTCCAAATCCC

ATAT-TACATAAAATTCTTCCATACATATTTTTAAAAAATGAACAGTAAAACTTGGCTCT

ACAAAATTAAAAGCATTTT--TAA--------ATTCTTCAGTGATTTTTACTTGTAATAA

AAAT-CCTAAA-TATACTTGTGGACGCATTTTTACA---CAGTACCAGCAAGGTGAACGT

TATCTTCTTCCACACGGGGGCAAAACAGATGACCACAAAGCACCTGAATCACA----GGA

TCTCCAATACACAAAGTG--TATTTTTCAGAGCTAAAATTATAAACAACA-ATAGA----

------------CACATTATACATGTGATTGATATCCAATTGACTTGAATCAGCCCCCAA

AACACAACCCCCAGTAGCAGGCAAATAAGGTACAATTCTTGCA-GCTGTTCCTGCCGGCA

ACCGAAGAGC-AAGATCGGTATA-CACCGTAATCTTCCCATTTGGAG-GAATAATATAAT

GATAGGCGCTGCATAAAATTAATCCCAGGCCTCCATTAGGATCTTGTCGAGGTGGAAGCG

CT--------CGACGAGATAAACGAGTGTAATACAATATTCCAGCATGGTTGCAGTTAGA

TTTCACCTCTAAAGAAATAAAACTAAA--ATTAAC-ATTCTAATCTTAAAA----TAAAG

-------TTAAAA---ATTAAAATATAGCTTTTAAAATTATTTTGTAGTTACTTACCAGT

TCCATA----CAAAAATTCTTTCACGTCCATTGAAGTCATATTC----AGCT--------

-TTTGAAAAAGGCTGCTGCTTCTCTGCAAAAGCTGGATAAAATC-TATTTT-----TCCA

AAACGGCCTTTTATATTCTTTTTCTAAAACAAATT--TAAAAAAAATCACATTACATCAA

-CTCTAGGCGGATCCAAAAGTAATTTATCAAAATTTCATGGA-----------AAAATCC

CCAAGTAAAAAAACAGTGGAAAAAGCCTCTAAAATGACCTTTA----AAAATCTCTAAAA

ATATAAAACCGTTATTCTGCTTA-----AAATGCATGACTTCAGTTAGCTA----TAAAC

ATTTTCCAGGCGTTTCGCCAAATGTTCTA-GGCGTGCGGCCAA---TTTAGAC-ATCCT-

-GTTCCCAACCATCCTGTTTTCCAGGCTAAAAGCCAAATTCCCTAAACAGCCCTCCCAA-

-GTTCCCAAAAACCTAAAATCTCAAAAAGCCCGTAATTTTAGCCATAACACCTAAAATAA

ATACCACAACTCTTATCTACACATTGTGCGGAATCGATCCATATATATT--ATTTTATCC

AAAACTACGGTAAAACCGGTAAAATTCCCAAAACTTACCGG-AGCGAAACCGGGCG--TG

CCGTTCCTGGGAACATTAGTATGCA-CAACAGCTGTGATC-CATAACGACCTGTCAACAA

AGCATTTATACGAGCCAAAAATAGATACACACATGA-------TCTCTGCA-AAAGTCAT

GTGATCACTAATTCTCTTTCCGTTTCCGTAATTTAACCCATACCTTACAAATAACTTCCC

ACACTACGTCCTCCGCCCCCT----ACTCCACCCCC-GACGCAAAAAT-------GTCCG

GTTATTTATTGATGATG-------------------------------------------

------------------------------------------------------------

------------------------------------------------------------

------------------------------------------------------------

------------------------------------------------------------

------------------------------------------------------------

------------------------------------------------------------

------------------------------------------------------------

------------------------------------------------------------

------------------------------------------------------------

------------------------------------------------------------

------------------------------------------------------------

------------------------------------------------------------

------------------------------------------------------------

------------------------------------------------------------

------------------------------------------------------------

------------------------------------------------------------

------------------------------------------------------------

------------------------------------------------------------

------------------------------------------------------------

------------------------------------------------------------

------------------------------------------------------------

------------------------------------------------------------

------------------------------------------------------------

------------------------------------------------------------

------------------------------------------------------------

------------------------------------------------------------

------------------------------------------------------------

------------------------------------------------------------

------------------------------------------------------------

------------------------------------------------------------

------------------------------------------------------------

------------------------------------------------------------

------------------------------------------------------------

------------------------------------------------------------

------------------------------------------------------------

------------------------------------------------------------

------------------------------------------------------------

------------------------------------------------------------

------------------------------------------------------------

------------------------------------------------------------

------------------------------------------------------------

------------------------------------------------------------

------------------------------------------------------------

------------------------------------------------------------

------------------------------------------------------------

------------------------------------------------------------

------------------------------------------------------------

------------------------------------------------------------

------------------------------------------------------------

------------------------------------------------------------

------------------------------------------------------------

------------------------------------------------------------

------------------------------------------------------------

------------------------------------------------------------

------------------------------------------------------------

------------------------------------------------------------

------------------------------------------------------------

------------------------------------------------------------

------------------------------------------------------------

------------------------------------------------------------

------------------------------------------------------------

------------------------------------------------------------

------------------------------------------------------------

------------------------------------------------------------

------------------------------------------------------------

------------------------------------------------------------

------------------------------------------------------------

------------------------------------------------------------

------------------------------------------------------------

------------------------------------------------------------

------------------------------------------------------------

------------------------------------------------------------

------------------------------------------------------------

------------------------------------------------------------

------------------------------------------------------------

------------------------------------------------------------

------------------------------------------------------------

------------------------------------------------------------

------------------------------------------------------------

------------------------------------------------------------

------------------------------------------------------------

------------------------------------------------------------

------------------------------------------------------------

------------------------------------------------------------

------------------------------------------------------------

------------------------------------------------------------

------------------------------------------------------------

------------------------------------------------------------

------------------------------------------------------------

------------------------------------------------------------

------------------------------------------------------------

------------------------------------------------------------

------------------------------------------------------------

------------------------------------------------------------

------------------------------------------------------------

------------------------------------------------------------

------------------------------------------------------------

------------------------------------------------------------

------------------------------------------------------------

------------------------------------------------------------

------------------------------------------------------------

------------------------------------------------------------

------------------------------------------------------------

------------------------------------------------------------

------------------------------------------------------------

------------------------------------------------------------

------------------------------------------------------------

------------------------------------------------------------

------------------------------------------------------------

------------------------------------------------------------

------------------------------------------------------------

------------------------------------------------------------

------------------------------------------------------------

------------------------------------------------------------

------------------------------------------------------------

------------------------------------------------------------

------------------------------------------------------------

------------------------------------------------------------

------------------------------------------------------------

------------------------------------------------------------

------------------------------------------------------------

------------------------------------------------------------

------------------------------------------------------------

------------------------------------------------------------

------------------------------------------------------------

------------------------------------------------------------

------------------------------------------------------------

------------------------------------------------------------

------------------------------------------------------------

------------------------------------------------------------

------------------------------------------------------------

------------------------------------------------------------

------------------------------------------------------------

------------------------------------------------------------

------------------------------------------------------------

------------------------------------------------------------

------------------------------------------------------------

------------------------------------------------------------

------------------------------------------------------------

------------------------------------------------------------

------------------------------------------------------------

------------------------------------------------------------

------------------------------------------------------------

------------------------------------------------------------

------------------------------------------------------------

------------------------------------------------------------

------------------------------------------------------------

------------------------------------------------------------

------------------------------------------------------------

------------------------------------------------------------

------------------------------------------------------------

------------------------------------------------------------

------------------------------------------------------------

------------------------------------------------------------

------------------------------------------------------------

------------------------------------------------------------

------------------------------------------------------------

-----------------------------------------------------

>Bat AdV-F

------------------------------------------------------------

------------------------------------------------------------

------------------------------------------------------------

------------------------------------------------------------

------------------------------------------------------------

------------------------------------------------------------

------------------------------------------------------------

------------------------------------------------------------

------------------------------------------------------------

------------------------------------------------------------

------------------------------------------------------------

------------------------------------------------------------

------------------------------------------------------------

------------------------------------------------------------

------------------------------------------------------------

------------------------------------------------------------

------------------------------------------------------------

------------------------------------------------------------

------------------------------------------------------------

------------------------------------------------------------

------------------------------------------------------------

------------------------------------------------------------

-----CATCATCAATATACCACCGGACAAATTTTGGCGCCAAAAG-AGGCGTGTTTTTTG

AGTGGGAGGGGTGGGGTGGGG---------------TGGAGTGTGGGAAGGGAAGTGACG

TTTTTTGATTTCCGCTGTCGGCC----ATTTTGACCCTGGGAATTTTCCACGGAACTTGG

CAAGCCCTTTGATTTTGCGTCATATCCGTGTGCTGTGCCAAGACGATCCGCCATAT-TTT

CCTCTTTGGCCT-----GGTTTGACCAGGAACTTGGCTGACCTTTTGTGTATTTGTGTGT

GAAATTAATGTGAATCATGTGCATTTTTGTC--CTTATCAGCGGAATGAACTGTTCCTCA

TT-AAACCACAATATTTCCACATTCCATAAC-------CATTTTTTAGCCC---------

TGCCCGCTT----TCCCAGTTAATC---CCTCCTTCCTTAGCCTCAAATTCCCTTGTGTT

TTTC-ACTCTCACGCAGCCTCTTGGACTTTCGGTTCGCTTTTCTCTGAGAAAAGGGTCTT

TTTGCCCTAAAAATGCGTCACTCCCTCTACTCTTTCAGTCAGGGGTTTTTCGATTTAGCC

GAGGAGCTTC-TTCAGGCCCCCGTGGATTTATCGGTTCCTATTCAGC----TCCACTATA

TGGAGGACGACGAAGACGTCTTTGTGAGTCCTTTGAGCTTTGAGACCCCGCCGCCTTCCC

CTGCTTTACCCACCCTGAACGAGCTTTGGGT-------TTCGGACGAGGAAGATGGACTG

TATGCAGATGGTCCTCTGCCTTCTCCTATACATTCTGTGGCTTCCAATACTGAGGATTCT

ATGGATGACTCTTGCGACGCCGA-----GGTTGCGGAGATTATGGCTAACAGTCTTTACT

GTTTTGAGACTTTACCTCCGTCTCCTTTGGGTTCGCC-TGCGT--CTCCTGGTTCTGAGT

CTGCAGCTGCTGCTGGTGGAGATA-----------CTCCTGAGATGCCTCCCCATCTTTT

------GGCGTGTCCGACGGTTCCTGGTGTAGATTGCCCGGCTT---GTGCTTTTCATCA

GGCTTATGGAAA------AAAATACTGCGCTTTGTGTTTTATGCGGCTGACTCATGACAT

GATCTTCAGTAAGTACAGGGTTA------------ATCATATTTTACTGTTGGGAGGGTG

GAAAT------GTACTGGAATTTGTGGGTAAA------------GTGGGAGGGGTTGGAG

GTTATATAAAGTGCTTACATAACTTGA-------TTTTTTATGAGGCTATTACTTTTTTA

TAGGTCCTGTGAC---------TCCTGTGTCTGACATTGATGAAGATTAAGTGCTGCAAT

TAACCTTTGCCCCATGCCCTCCCT------------------------------------

---------------TTCTTCTACATTCCTTTAA-TAATAAACTGGTTTTTGGCGGAATT

TTTTGTTTGTGGTTCTTGGCACCTATAAATAGGGGGGAGGAGAAAAATTTTTTTAATAT-

AAAGCAGGAT------------GGAGAGGGCTGCAGACATCCCTTTTTCTTTTCAGGGAT

TA-AAGCATATTTTGACTTTG-ACTTATAAAGCTTCTAAGTCTTCTTGG----TTTTGGA

GAAAATTTTGTACTCCTTC----TTTAATTCTTGCTATTCTTGAGGCTAAGGAATCTTAT

GCTGAGGACTTTGATTCTTTTTTAACTACTGATTTTTGCTTACAAAGTTTTTTAGATACA

TTAGATTGGCAGTCTTTAGAAAAATCTTTAATTCCCAATTTGGATTTTTCTTCTTCGGGG

AGAGCTATTGCTAGTTTGGCTTTTTTATCTAGTATTATAGACCGCTTGGAATTTTCT-AC

TATTTTTACATCTTCATATTTAATTGAAAATTTTGCTTTTGCTGTGTGGAGATGGGCGAT

AACATTGACGAA------------------------------------------------

---------------------------------------------GGGATCGA-------

-------------------AGACATCGGAGCAGTTGAAAATGTC----------------

----------------GAAAATGAAGACCCCCGAGCTAACGGAGAAGCAGGAGGAGGCGC

TGACGGAGCTAATGAAGAGTTGGGATTAATGATTGCAGAA------AGTTTTTTAGTAAG

AGTGGCATTTGGGTCCATG-------------TATTACTATGAGCTTATTGAAAGAGCAA

ATCAGGACCCTCAGTCTATTTTAAGGGTTTCTGTAAATTTTGAAGTGATTAAATACTATC

AT---TTGTCTCCAGAGGATGATTGGGGGATGATGATTAACAGATATGCAAAAATTAATT

TGGATCCAAAATATGAGTATACTATTGATATGCATACAATAATTCATTCTGATGTCTATA

TAAATGGAAATGGTGCTGTGGTTATGTTTGAACC---TGGTGGATA------TTTTGAGG

TTAAAAAAAAG---GGTTTGGTTCCAAATATTTTTGGCATGACAAGAGCTGTTTTTTATA

ATTGTAAGTTTGTTT-------CTCATAGACTTTATGCA-GCTATACCTTTTTTGATTCA

A------AGGGAAGCTCTTTTTATGAATTGCACTTTTTCTAATTTTCCAGATAATACCAT

TGTGTCTCATTCCACTGTTCATATTCGAGGTTGTTTTTTTAGTGGGTGTGAAACAGGGGT

AAGAGGATTAAATGTTAATGATTATGTTTCAGTGTCATCTTCCACTTTTGCAAATTGTCA

GGT-GGGG----GCGACATGTAAAGGAAAATTAAAGGTTGT-TAATTGTT----ATTTTG

ACACCTGTAAAAAT-GCTGC----CATTTTTATTAGTT-CTGGAAGATTTAGTGGAAATA

TTGTGACTCATCCTAAA--TTGGAACATGAGGAAGGTCAAGTTACAAT----TACCTGTG

CAGAAGGAAAAATTTTGCC------TTTGCAATCTGTTCATATTGCTTCC---AATCCAA

AATGTTTGTGGCCTATTTTTGAAAATAATACATTTTTTAGATGCAGGTTGTAT-TATGGT

ATAAGAAGGGGCTTAATGTTTGCTC---CCATGTGTCACTTCAG-AAACACTATTTTATT

TGTGGAAAGAGAGGTGGTTTCACGCA--TTGTGTT--TCATCAGTCTTT-TAATCAGGAT

TTAAAAGTTTTTAAAGT-------ACTGCGTTTGGAAACTACGCGTACCTTTCAAAGAAA

ATGTGAATGTGGATTAACTCACAACGTGTATCCATTCTTATATGGAAATATAAC----AA

AAT-----CAAAATTGGGACCTGATTATCTCAACAGCATGGACAACCTTGATTTTACATC

AGATGAAGAAGG--TATGATATCA-TA-----------GTGGGAGGGGCT-AAAAACATA

TATAAATTCCG-----TTATTAAATTTTTTTTATTTCATTGCTTCCAGACTGAGGATGCA

AGATGTCCCG---------CTTTTCGACTGGAGCTGTGAATACTGCTTTTTTAACCACCC

GTTTGCCAACCTGGGCCGGAACTCCACAGAATGTTTCTGGAAGTGATTTGG--AAGGACA

ATCTATACCTGCATCTAATGCGCTTCCCAATGCTGAGACATCGTCA--------------

-------TTTGCTGACATATACAAACTAATAGA---------TAATATG-----------

--------------------------GATAAAGAAATAAAT-GCTTTA------------

----------AAACAAACTTTAG--------ACCG------CCTAATAAGTCTAACCCCT

GCTGCTTA---------------ATAAAAA------------------------------

---------------TTCAAA---------------------------------TG----

----TAATAAAATTTTTTTGAAAAACTTTA---------TTTATGTTTGTGGTAGTATTT

ATTCCATCTTTCGCGATCAGAAATTACTCTATGAATTTTTAACAAAATTTCATACAACAT

ACATTGTACATTTAAATACATGGGCATTAAACCTTCTTTGGGGTGTA-AATACATCCATT

GTAAGGCCTCATTTTCAGGACAGGTGTTATATATGATCCAATCAAAATTAGAATGTAAGT

TATGATAATTAAAAATATCTTTTAATAATAAACTTATAGCTAAGGGCAGTCCTTTAGTAT

AGGTATTAATAAATCTATTTAATTGAGAAGGTTGCATTTTTGGACTTAATATATGTAATT

TAGATTGAATTTTTAAAGTGGCTATATTTCCACTGTGATCTTTTCTAGGATTCATATTGT

GTAATACAACAAGTACAGTATATCCAGTACATTTTGGAAATCTATTGTGTAATTTAGAAG

GAAATGCATGAAAAAATTGGGATATACCTTTGTGAGAACCTAAATTTTCCATACATTCAT

CCATTATAATAGCAATAGGACCATTTGCTGCGGCCTTAGCAAAA-ACATTATTTGGATCC

CCAATGTCATAATTTTGTTGAGCTGTAAATTCATCATAAGACATTTTTATAAATTGTGGT

AAAAGAGATCCACTTCTAGGAATTATAGTGTTGTCCTCACCTGGCATGTAATTTCCTTCA

CAAATTTGTATTTCCCAAGCAGCTAATTCTTGGGGAGGTATCATTTCAATTTGCGGTACT

ATAAAAAATATAGTTTCAGGGGGTGGATTAATTAAATGACAAGACAATAAATTTCTTAAT

AATTGAGATTTTCCACTACCTGTAGGACCATATATAACACCTATTACAGGCTGCATATTA

AAATTAATAGATTTACAAGTTTTATTGGGATTTAAA------------------TAAGGT

AACACAGAATTCATAGCTTCTGAAATTTGGTTATTTTCATTAACCAAGTCATTTATA---

-----------AGTTTATCCCCACCTAAACT-----------------------------

TAACAGTTCTTGTAAAG-AGGAAAAATTTTTTAAAGGTTTTAATC----CTTCAGACCA-

----TGGCATAGATTTT-----AATGCATTTTGTAAAACATTTAATTTTT-------CCC

ATAATTCTGTTACATGT----CCCAAGGTAATTCCGTCC----------AATAAACTTCT

TTGTTTCTTG--------GATTTGGATTGCTTTTGCTGTAAGGTA--CTAATTTGTGTTG

ATCC---------------------ACTAGTACCATTGTTTTGTCCTTCCATGGTCTTAA

TTTTCTTGTTAGGGTTGATTCCATCACTGTGAACGGTTGCACATTGGTTTGACATGATGC

TAAAGTTCTTTTCAAAGTCATTCTGCTCGTCTGGTAACGTTC------------GCTTCC

TTCCTGAACATCAGTATA---GTAACAAGCACTTAATAAG---TCAAAAGATAAAGTTTT

GGTAGCATGGCCTTTTGCTCTAAGTTTACCAGGGCCAATA------TGTCCACAGTTATC

ACAACAAGTATTTTTTAAAGCATATAATTTTGGAGCCA-AAAAAACACTTTCTGAACTAT

AAGCATCACATCCACATTTTGAACACTGTGTTTCACATTCAACCAGCCAAGTTAAACTAG

GATTTTGAGGATCAAAAATTAATTTACCCCCGTTTTTTTTTAATCTGTGCTTACCTTTAG

ATTCCATAAGCTCTCTTCCTTTTTCTGTTACAAACATACTATCTGTATCTCCATACACAG

ATTTTAAAGGTCTTTCATGTAGAG---GAATTCCTTTATCATCATCATATAAAAATCCGG

CCCATTCGGAAACAAATGCTCTTGTCCAGGCCAACACAAAAGAAGCAATTTGCGATGGGT

ATCTGTTATTTGTAACCAGCGGAGTATTTAACTCCAATGTGTGAAGACATGCGTCATCAT

CTTCAACATCAAGAAATATGATTGGCTTATA---GGTATAAGTCACGTGA----------

----TTTTG--------TGGGGTATTTAAAGTGACGTCTTCATCGTCGGAATCAGTGTCC

ACTGGAGAGTGGGGACC-TTTCTGGC-----------TGGTGTCA---------------

------------------------------------------------------------

--GGTGAGTACAAAACTTTAAA---TTCGGGTAAAAGTTCAGAACTAAAAGTGT------

------------------CAGTTTC----------TATGAATGATGATGCT--------T

TTACATAATATTGACCACTTGCAATTTTGTTTTGTGTTTCTTTATCTATTTGATCAGAAA

ATACTGTTTTTTTATTGTCAATTTTGGTAGCAAATGAGCCATATAATGCATTAGATAATA

ATTTTGCTATGCTTCTTAAAGTTTGATTTTTTTCTTTATCTGCTTTTTCTTTGGCTAAAA

TATTTAATTGAACATATTCAGCTGCCACACATTTCCACTCTGGAAAAATTGTTGTTTTTT

TGTCAGGTAAAATTTTAACTTCCCAGCCTCTATTATGTAATGTTATAACATCAATAGATG

TAGCAACTTCATTACGTAATGGTTCATTAGTCCAACACAGTCTTCCCCCTTTTTTTGAAC

AATAAGGGGGTAAAACATCTAAAAATTGTTCATCTGGTGGATCTGCATCTATTGTAAATA

TACCGGGTAAAAGG-TCTGGGTTGAAATATGATATATTTTCATTTCTACT--TAAATATT

GTTTCC----AAATTTGAATTTGAACTCCACAATCAAATGCACTTAAAGGTAAGCCAACA

GGAAAAGGGTGAGTTAAGGCACTAGCATACATTCCACAAATATCATAAACATAAATTTTT

TCACTTAATACTCCTAAATAATTTGGATAACAGCGACCTCCTCTTATGCTGGCTCTTACA

TAATCATACATTTCTTTTGATGGCGCTACTAAT------GTTTTACCTATATGTTTTGAT

AAAGGTTTTTCAGCTCTAT-----------------------------------------

----------------------------AAACAATCTGCTTAAAAATTGCATGTGAGTTT

GATGAAATTGTTGGTCTTTGAAAAATATTAAAATTACATTCAGGTAAATTTACAGATTCA

CATATAAATTTTTGATAAGATTTTTGCAATTCTTGAACCAATTTTGTTGTAACTAAAACA

TCAAGAATACAGTATTTTAATGTTTCCTGTATAATATCATATTTT---------------

---------------------G---TGTTTGATTTTTTCCACAGCTCTTTATTAGTGTTA

TATTCTTCTGGACTTTGCCAATATTTAATAGAAGGAAATCCATC---GGGATCTGAC---

AGGTAAGATCCAAACATATAAAATTCATTTACTGCAGAATAAGGACATGATCCTTTTTCA

ATTGGCAAATTATAAGCTTTAGCTGCATTTTTTAAGCTTGT---ATTAGTTAATGCAAAT

GTATCTCTAACCATAATTTTTAC-------------C-ATTTGATATTTTAAAT--GTTT

GCTTTCAAAAATACCTT--TTTCCCATTGTTCAAAGTCATTTTTACCTGGGGATTCATAT

TCA---GGATTTGGTAATGCATAAGTTATATCATTAAATAAAATTTTTCCACATCTTGGT

AAAAAATTTCTAATAACTT---TAAATGGCTGTGGAATTTCTAACTTATTAT-------T

AATTACTTGAGCAGCTAAAACAATTTCATCAAACCCATTAATATTGTGTCCAACTATATA

AATTTCAAAAA--AAGTGGGTTCTCCTTTTAATT--------TTAATTTAA---AAAATT

CTTCCTGTGGTATATCATAACATGAAATTAATCCAAGTTTATTTTTTAAATCATTAATGC

ATGGATTTTGAACTTGTAAC----TTATCCCATAATTTTATTATT---AAATATTGTTGA

ATTTTTTCTCTTAATACTTTAAATTTTTGTCCTACTTCTCTTTTATGAGGATTAATATAA

AAGTAAGTATTTTC------TTCAG---TACATTTATTCCAATTTTCTCTAATAGCTATT

GATGAACATATTTTAACAAGTTCATCATCTCCAGAAAAATTTAATACCAGCATAAATGGA

ACTAGTTGTTTTCCAAATTTTCCATGCCATGTATAAGTTTCAATATCATATGTAATAAAT

ATTTTTTTAATTTTAGAACATGATCCAATAGGTCTAAAAGCAATATTTTCCCACCAGTCA

CAAGTTTGAGTGTTAATATGGTGATAGTAATAATCTCTTCTTCTTAAAGAACAAGCATGA

TTGACTTTATAAAATCTTCCACAGTGCTCACATTTATTTAAACTTACAACTGAATGTATT

-AAGTATACTTTATAATTCT-----TTATTAAAAAATTTAATGGAATT------------

------------------------GATAGTTTGGGATTAGGAATTGTAACTTTAAATT--

----------------------------GTTTAAATTTACTTTTAAAAGCTGTATAAACA

ATTGAAGATGTTGGACAAGCTTGTTCAATTTTTTCTATTAA---------ATTTTTATAA

GTAATA------GATTGCAAACAAAAAGGAAATGTAAT---TAAATTTACCTTAAATAAA

TTATTAAGTGATTTTTCTAAATTGTTAAAATATTTAATTTCTAATGGTGTGCCTTCTTCA

T---CAATACCTTGGGCTAAACCACATGCTCTTTGAGCCACGTAAGTGCC----------

------------------------------------------------------------

TTTTAA-----------------GTTACTCATTGT---AATGGAACAT-TAGCA---TTT

AA-----TTCTGGAAATGGAGTTCTGTTTTCTCTAAAAAGAGTGGCCATATTAATAACCT

GAGCATTGATTCTTCTGATTTCTGGATGTTGAGTCAAAATAACTGGACCAGTAATCTTGA

ACCTGAAGGAAAGTTCCACAGAGTCAATTTCTGCATCATTAA-AAGATATTTGTTTAATT

AATTCTTCTATATTACCACTATCTTCATGATATGAAATTTCAGCCATAAAATGTTCTAAT

TCTTCTTC-----TGGAACATCT-GCTACTCCTGCTTGTTGAACTCCAGCAGCTAAATCT

ACACTTATTCTTCTCATAACATTAATAAA------AGCATCTACCCCATATTCATTCCAA

ATTCTAGAGTACACAAATGTTCCATCTTGATCTCTTGCCCTTAAAACCACTTGAGCTAAA

TTTATATCCATATGACGCATAAATGCTCTGTTATTTCTAAAAGCATTATGTAAGTAATTT

AAAGTAGAAGCAATGTGCTCAGTTATAAAAAAATTCATAATCCATCTTCGAACAACTGTT

TCTGTTATGTTTTCACTTGCTTCTAACTCTTGCATTATTCTATAAAAATCTACAGTAAAT

TGAAAAAATCCTTGTTCTCTAACTATAGGTGAAAGTTCATCTTGAAGAAGTTGTATTACA

TGAGCTATTGTGTTTCTAACTTCTTCAGCAAAA------------GGAATTTCTATAATT

TCTTCTTCTT------------CCTCTAATTCTAAATCTGGCGAGGGTGAA---------

-----GGTGATACTCTTTGTTCTCTTTCTA-CTCGTCTTCGACGTCTAGGTAAAGGAAGA

CTTTCAATAAATCTTTGTACCATTTCACCTCTTCTAATTCTCATTTC---TTGTGTTACT

GCTCTTCCGCCC---TCCCTAGGTCTTAGTTCAA----AAGCTCCTCCTTGTAATT----

----------------CAAATGGAGATGGA-------TTAGGGAGACTTAAAGAACTAAT

AATTGCTT-TTATTACTTTTTGCATGCAGATTCCT--------GGGTTTGCAAGAAGTT-

---CCTCATTAACAGGATCACTAAACTTTT--CAAGAAAAGCATTTAACCAATCACAGTT

ACAAGGTAAG---CTAAGTTTTAATTCATC------------------------------

------------------------------------------ATTTTCACTTAATAAATA

ATTAAAAAAAGCACATTTAAGTTTTCTTATTGTGCTTAATATAACCATATCGCGTCGTTT

GCCTTGCTGAAATCTAATTTGCT-CTGCCATGCCCCAAGC--TTGCTCCTGACATTCATT

AAAATCACGT--CTTTGATTTTGCAAATAAAATGGCAAAGAAACTTCTTC----------

------------------------------------------------------------

-----TCCTGCTATTCTATT--TAAT-CCATAACCCCGTAATGGTTCTATTAACCCTATA

TCAGCAATAATTCTTTCAGCTAAAATAGCTTGTTGAATTTGAGTTAATGTTTCTTGAAAG

TT------------ATCAAAATCTATAAATCTATGATAGGCTCCAAC-ATTAAGAGTATA

AGAACAGTTAGACATCATAGACCAATTCAGCAT--TTGTTGCATATGGGCATTAATTTGC

AAG----TATTTAATTCTACTGTAGGCTCTAATATCAAAT-ATATGTTCATTACATGTGC

GCACAATATATTGATA-ACCAACTAAAAAATGTGGAGGTGGCCAATTGTATAAGGGCCAT

CTATGGGTTACAGGTTCCCTTGGAGATAGGTTCATTAACATAATTCGAGGATAATGA-TA

AATGTAGCGCGACATCCATGATAATCC-TTTGGCAGTAATTGAGGCTCTGGTCCATTCTC

GAGCTCTAGACCAAAAATTTCTAATTGGTCTAAAAACTTCAATGGTGTATTGTGTCTGGC

CTGTAAGTCTTGCATAGTCTGCGGCGTTCTGAA---------------------------

------------------------------------------------------------

------------------------------------------------------------

------------------------------------------------------------

------------------------------------------------------------

------------------------------------------------------------

------------------------------------------------------------

----------------------AGAAGAAATATGGCCTTT--------------------

------------------------------------------------------------

------------CAG-ATGCATCCAGTTCTTCGCCAGATGAAAAGCAGCAAA--------

----------------------------ATGCCAGAAG----------------------

-----------------------AGTTCATCCGCAATGAA--------------------

-------------------------ATGGAGGGTGAAGGATTAG-CTAGGTT------AC

AATCATCCCCAGAGC--AACATCCACGTGTTGCAATAAAAAAAGATGCCGCTGAGGCATA

TGTACCTGAACAAAATTTGTTTAGGGA---------TAATGAGGGTCAAGAACTTGAAGA

AATTAGAGATTTAAAATTTAATTCTGGAAAAGAAATGTGTTGTGGTATTAACAGAAAAA-

AACTTTTAAAAGAGGAAGATTT--TGAATTAGATGAAAAAACAGGCA------TAAGTCC

AGCTAGGGCCCATATTAATGCTGCAGATTTGGTTACAGCTTATGAACAAACTGTTAAAGA

AGAAATGAATTTTCAAAAAGCTTTTAATAATCAAGTAAGGGTTCTAATTTCTAGAGAAGA

AGTAATGATTGGATTGTTACAATTGTGGGACTTTATTGAAGCTTATAAAATAAATCCAAA

A-------AGTAGGTGTTTAACTTCTCAACTTTTACTTATAGCTCAACATATTAA-GGGA

CCAGGAAT---ATTTAGGGAAA--CTTTTTTAAATATTAATGAACCA----GAATCAAGG

TGGTTGTATGATCTAATAAATCTTTTACAGTCAATTGTGGTTCAAGAAAGGTCTTTAAAA

ACTCCTGAAAAGGTGGCTGCTATAAATTATTCTGTAATAACTTTAAGTAAATTTTATGCA

AGAAAAATTTTTAATTCTCCATATGTGCCTTTAGATAAAGAAATTAAAATTAATACTTTT

TATATGAGAATGGTGTTAAAAATTTTAG------CTTTAAGTGATGATTTGGGAGTGTAT

AGAAATCAAAA--AATTGAAAGAGTTGTAAGTTGCACAAGAAGA-----AAGGAAATGAA

TGATGATGAGCTACTATTTAATTTAAAAAGATC----TTTTGCATCTTCAATGT------

---------------------------------------------------------ATG

ATGAGGTAGAGTCTGATG------------------------------------------

------AAGAAGTAGATGATGAGTTTTATTTAAACAGAAAACCCTA-----------TTT

TGAAGCTCAA----------------------CCTCACCATCCGCGTCACCATGTCAAGT

TCCAGAATGAACCCCAACGCATTAGCGATTTATCAGAGTCAACCAGCATCTCAA------

---GAAGATTGGGCGGCAACTTTTAAAAAAGTCTTGGCTTTAACTAAAAACAATCCTGAT

AATTTTGCAAGTCAACCTAAGGCTAAT-AGATTTAATGCCATCTTAGAAACTATAATACC

TTCTAGATCTAATCCAACTCATGAAAAAGTTTTGTGTATTGTGAATGCTTTAATTGATTC

TAAAGCAATTCGAAAAGATGAAGCTGGTCAAATGTATAATGCATTGTTAGAAAGGGTTTC

AAAATATAATAGTACAAATATTCAAACCAATTTAGATAAATTGGTAGGAGATGTTA----

--GAGAGGTTACAGCGCTTAAAGA------AAAATCTTCTCAAAATGTAGGTTCAATGAT

TGCTTTAAATGCTTTTTTAAGCAAATTGCCTGCCACTGTTGAAAGGGGTCAAAATGAATA

TATAGCTTTTATTAGTGCCTTAAGAATTTTTGTAGCTGAAACTCCACA---AACTGAAGT

ATATCAAGCTGGTCCAAATTTTTATTTGCAAACAACAAG-AA--ATGGGTCTCAAACAGT

TAATTTAACAAGAGCTTTTGATAATTTAAAATCTATATGGGGTATAAACAGCAATCAGGC

TTCAA-ATACAAGCATCTCATCTCTT-----TTAAACCCCAATACAAGACTTTTATTATT

ATTAATG-GCTCCATTTA-CTTCTAATACTTCAATTCCAAGAGGAAGTTATGTGGGCTAT

TTATTATCTTTGTATAGAGAGGCATTAGGAAATACTCATATA---GAAGAAAAAACATAT

CAAGAAATTCAAGAAGTAAGTAGAGCTTTAGGACAAAAAAATTCTG---AAAACTTACAA

GCAACATTAAACTACTTACTTACTAACCGCCCTAAAAAACTACCTGAGGAATATTCATTA

ACACCTCATGAAGAAAAAATTTTAAGATATATTCAACAAGCAAT-AGCTTTATATATGAT

GCAAACTGGAGCTACTG--CTACTACTGCTTTGGACCGCACTGCTTCTTCATTTGAACCC

AGTTTTTATTCTCAAAACAAAGGGTTCATTAATAAACTAATGGGGTATTTTTACAAGGCC

GCTGAAACATCTTCAGATTATTTTTTGAATGCAGTGTTAAATCGCCATTGGCTACCTCCC

CAGGGATTTTTTACAGGCAATTTTGATTTTCCTGAAGTTGAAGATGAC---TACATCTGG

GAAGATTTTGACAGCGCTTTGTT--------------------------TGACAGGGAAC

AAGATAAAAAAAGCTTTTCGGGAAGCATTCGCCAACATGAAAACCTTAGTGTCCC-AGGC

TCTGAG-------CCAAACATTTCACGAG--GTTCTGTGTCCTCAG----CTCTTGGAGC

AGTTGGACCAGCTCCGTCATATA---------------------------------GTCC

GCTCCCA-GAAACGCGGGTCAAAAA--CAAACCATTAA-------------------TGA

ATAATGAAATTGAAACATTAGCCGATAAGTT-TGCAAGATGGAAAACATA-TGCCCAAGA

ATATCAAAA---------------------------------------------------

---------------------------------------------------TAATTTA--

---------GATACATTGTCTGGTAAAGG-ATT-----------------TAATGCTTTT

AAACATTTAATGCCAAATGGGGGAAAATATTAATAAAA---------------AATAACA

T---------------CTTACCAAAGCCATTTTGATCT---ATGTG-TCCTTTTTGA---

-CCAGTAACA--------------AAATGGA-----------------------------

-----------------CCCTTTTTCAACGATGTTTTCTC--------------------

-----------------AA-----------CGTCAAGATATTTATGTGCCGCCAAGATAC

AGCGCT-CCGTCTGAGGGAAGAAATAGCATCACTTACTCTCAATTACCCCCACTTTATGA

CACAACAAAAATTTATTTAATTGATAATAAGTCTGCAGATATTTCATCTTTAAATTATCA

AAATGATCACAGTAATTTTTTAACAAGTGTAGTTCAAAATAGTGATTTTAGTCCTATAGA

AGCAGCTACTCAAACAATTAAATTTGATGATAGATCTAGATGGGGAGCTAATTTAAAAAC

TAT--ATTATATATGAACATGCCTAATGTAACTGATTTTATGTTTTCAAATACTTTTAAA

GTTAAATTAATGAT------ATCCAAAAAAGATA--------------------------

----------ATGTGCCAGTATATGATTGGGTTGATTTAAAAATACCTGAAGGAAATTTT

TCTGTAATGAAAATTATAGATTTAATGAATAATGCAATAGTAGATCATTATATGTCTGGT

CCTAGGGCTAATGGAGTTAAAGAAGAAGACATAGGAGTTAAAATTGATACTAGAAATTTT

ATGTTAGGTTATGATCCCACTACTGAAGTTGTTACTCCTGGTGTTTATACTTATGAGGCA

TTTCATCCAGATATAGTTTTGTTACCTGGTTGTGCTATTGATTTTACATTGTCTAGACTA

AATAATGTGCTTGGAATTAGAAAAAAATTGCCATTTCAACATGGGTTTATAATTTCATAT

GAAGATTTAGGAGGAGGCAATATACCTGGATTAATGAATTTAGTATCTTAT---------

--AAAAATGGACA-----------------------AGGTGAAAT---------------

---------------TGAGGTGGT--AA------------------CAAAAGA-------

------------------------------------------------------------

------------------------------------------------------------

-----------------TGAAAAAGATAGAAGTTATCATATAGGAGAA---GATAATTCT

GTG---CCTAAAACTTTTACATCTTACAGAAGTTGGTATTTGTCTTATAATTATGGAGAT

GAAGAACAG---AGTATTAGAAATTCAACTTTGTTAACTAATCCAGATATTACCTGTGGT

GTTGAACAAGTTTATTGGAGTTTACCTGATCTAGCTTGTGAACCTGTAACATTTAAAGCT

TCAT---ACAATGTAAACAATTATCCAGTTGTTGGAACAGAGGTGCTGCCAATGCAATCA

AGATCTTTTTTTAATGCTCAAGCTGTATACTCACAATTGGTGAGCGAAAGAAC-TAATCA

--AACGCACGTTTTTAACCGATTTCCCGAAAACCAGATCTTGGTGAGGCCACC-TGCGCC

TACTATTTCAAGCATCTCTGAAAACGTCCCGGCAGTAACAGATCACGGAGTCGTCCCTAT

TAAAAACACACTTTCTGGTGTTCAACGAGTGACAGTGACAGACGCCAGAAGAAGAATTTG

TCCATACGTCTATAAAGCAGTTGGGGTTTTAAGCCCCAAAGTGCTATCAAGTAGAACATT

GT-AAAA-----------------------------------------------------

---------ATGGCTATTTTAGTTTCACCCAGTAATA-ATACTGGGTGGG------GTCT

TACTAAGACCTATA------TGTATGGAGGTGCAAGAGAATTTAGTGAT--GATACTCCA

GTGCATGTAAGGGAATATTATAGAGCCCCTTGGGGCAGCAAAAAAGGAAGA------GTA

GTTAGAAGACGAAGGAGAAGAAGAGCAGCTGCTAGGGCTGTGGAAG-------------A

TATTGCTGAAGCCGCAGCTGAGGCTGTT---------GCTGAGGCAACAACACGTAGAAA

AAGAAAAAGGCGCGGTCGTAGGCCTTGGGGGCGTAAAAAAAGAAGAGTGGTTGTTGTAAG

AAATGCTAATGG-TCAGCAAGTTGCTGTGCAAGCTTCAACTTCCTCTTAATAAAAAATTC

ATTTTGCTGCACAGCCT-----TACGACTGATTAATCATGTCTTCAAGAAAAATAAAAGA

AGAAATGCTTCAGGTCATCGCACCGGAAATATACAA-----ACCAAAAAAGTTTAAAATC

AAGAAAAAAGATGTAAAACCAAAAAAAATATCTTCTTCTGA-GGTAGAAGAAGTTATGTA

CATACCTCCTCAAAGAAAGTATCAGTGGACAGGAAGAAAAGTTGCAAAGGTTGCTCGACC

TGGTGTAGTAGTTACCTACACCCCTGGACTT-----CGCTCTGGAATTTCATCTAAGCGA

CCTTATGATGAA---GTTTATGCAGATAATGATATATTGGAACAAGCATCTTTAAAAACA

GGAGAATTTGCTTATGGCAAACAACCAAAAATAGAATCAATTGTTAATTCTTATACACCT

TCATCTTTTAAAGAAAAAGTATCTGTAATCACACCTTCTTCACTTAAAGAAAAAGTAT--

CTGTAGTTAAAAAGTCAAATTACAGTCCTTATGTTAAACCAAAAGTTAAAATAGAGACTG

TAAAAGAAGAATACAAACCAAATTTTAGAGCTTTTGTTGACGCAAGGCCAAAATTAGTAT

TAAAAGAAAAATCTAAAATTTTAAAACAGTCAGAATATAAACCATATATAAAACCAGAAC

CTGGAGTAAATATAAAAGAATTTAAAAAAGATTTAAAAATTATTAAACCTAAATCTAAAA

AAGAAGAAATTTCTGAAGTTGAAGTTATGGAAATACCTAAAAAAAAGTATCAATGGAAAG

GAAGAAAAGTTCAAAAAGTTGCTAGGCCTGGTGTTGTTATTTCATATACTCCAGGGGAAA

GATC---AGGTCCTGCAATAAAAAGATCATCAGATGAAATATTTACTGACACTGATATTT

TAGATCAGGCTAAAAATAAAATAAATGAATTTGCTTATGGAAAAAGACAAAAAAT-----

-------TGTAACTGATACTGAAGTTCCAATGCTTATAACTCCAAAAAAAGAATTAACAA

TAGA---------------TATAAAAAGACCACTAGCTGAAATTAAAGTTGAAGATACTG

TTCAACCTAAAGTAGAGCCAATGGAAGTGGTTAAGTCTACCACTAAAAGATCTTTTGAAG

GTGACACAGTTATTGGTAAAAAAAGAAAAATTG----A---TACTTATGTTCCATTATCT

ACTCATAATCCTACACCACATTTAGAACCAA-----------------TAACAGAACAAA

AAATAATTCCTATGTTTCCAAAATCAAGTACTG------TTCAAGCAACTGGTCAGGTGA

TGACTTCCAGTAGAGTAATTAAAAAAGCAAGAACTAAAAAAGGCGAGGCTAAGTTTAAAG

TAGATGTACCAATGTCAACT------------------TCTATGGGTCCTGCTGTTGTT-

AAGACTGATGTAAATGTAATGCCTGTAGAAC---CTGTTGCTCCTGGTGTAGGTGTAAGA

ACAATTGATATTACTATT-------------CCTGAAAAAATGGAAGTGGTTTCTCAACC

AACATCTTTA-------------CCTACTATTGTAAAAGAAGTGGCAGTAACAAAATCAG

CTCCGTCTGGGTTAAAATCTGCAGTTGTAAAATCTTTTCAACCTGTTAAATTAATTTCTG

C---TCCTCCTTTGTCTTCTTATACAGTTGTTCATCCTAAAGTAATTT--CTAAAACCAT

TACTGAATATAAAAAACCTGGTGTAGTTGAAAAATCTGTAAC-TAAAAAAAAGTACACTT

CAGCAA----------------------------ATTCTATTATTCCAGTTGTAAGGTAT

CATCCA------AGTATAAAAATGCCAACAAAGTACAAAAAGAAAATAATTCC---AAAA

GTGCGTTATCATCCATCTATTAAAATGC--CTTAGCTAATTTTTATTATTCTGTAATT--

------AATTATAGAACTCGCTATCCAGTTTATTAATTCACCATGGTGAAGCGAGCTGTA

ACCTACCGCATCAGAGTACCTGTTGCTCGAAGAATGATTCACAAAAATAA----------

---------AAGAAGATATAGGCATAGTAGACA-TAGTCTTAATGGAGGTTTTTTTCCTG

CTTTAGTGCCACTAATTGCTGCGGCTATAGGAGCCATTCCTGGTATTGCTTCTGTAGCCT

TGGAGGCTTCTCGTAAAAAATAGTGA----------------------------------

----------------------TTAAATCACATCACCTTTTCTTCA--------------

----------ATAGAGACTTTTTGTTGAACTGCTGCA-----------------------

--ATGGAAGAAATCAATTTCACCGCTTTGGCCCCACGGAGAGGCAACAAACCCATGCTAA

CAGCATGGTCTAGCATTGGCAATAGTCAAATGAGTGGTGGAGCTTTCAATTGGGGTTCTG

TTTGGAGTGGGCT---------------TAAAAGTGTTGGAAATACTTTAAAAAATTGGG

GTAATAAGGCTTGGACCAGTAGTACTGGAGAGGCCTTAAGACAAAAGCTTAGAGATACAA

ATTTTCAAGAAAAAGTGGTGGATGGAATTAGCACAGGAATTCATGGTGCTGTTGATATTG

CAAGACAGCAACTTGATAAAGCAATAGAAAGTAGATTAGAAA--------GAGATCCTAA

TG-----------------------------------------------AAGAAGAAGAA

ATTTA-----------------TTCTGAAAAAGGCTTTATAGAAAAAGAAATGCCTTCAA

GAAGAAAAAAG-CGCCCACTGGAGGAAGAATTTGTTATTACTTCTGAAGAACCACCAGCT

TATGATGAAGTTTTTCTTAATTATGATGCCCCAAAGCAA-------------ATAACAAA

AG---------------------------------AGGTTGTGCTAAAAGAGCCTGTGCG

TAA---------AACTCCTAT------------AAAAGAAATTACATATCA---------

------------------------------------------------------AGAACC

TGTATTTACA-------------GCTCCAGTGGCCA-----GAAACTGGCAAAATACATT

AAACAGCATTGTGGGTTTGGGGTTGCCATACAATAAAAGACGACGGTGTTTTTAAAA---

---ATAATAAAAAGCTTAAGTGTTTTGTTAAATT---AATAATTATCCCAATACCAGGAG

G----------------TGAAGTACATCAAAATCCAA----------------GACGATG

GCTACTCCGTCGATGATGCCCCAATGGGCCTACATGCACATCGCAGGACAAGATGCCTCA

GAGTATCTATCCTCTGGTTTGGTTCAATTTGCCCAAGCTAC-TGAGTCATATTTTGACTT

GGGTAATAAATTTAGAAATCCAGTGGTGGCTCCAACACATGATGTAACTACAGATCGCTC

TCAACGTCTACAGTTAAAAATTGTACCAGTGGATAAAGAAGATTCAGCTTATTTTTATAA

AGCCAGATTTAATCTTGCAGTAGGAGACAACAGAGTGTTGGACATGGCCAGCTGTTATTT

TGACATAAGAGGAACCATTGACAGAGGTTCATCTTTTAAACCATACAGCGGCACTGCTTT

TAATTGTTTGGCACCAAAAAGTGCTACTAATAATACTG-------------------AAT

ATATTGG------TG---------------------------------------------

--GTGCAGACAA------------------TGTAACAAAAACAATTGCTCAAGCATCATA

TCCAGCTATTGAATTTGAAAATGGAGACTTAGTGGTTGCTAAGGAG--------------

-----GGCACTAATAAA---ACAAAAGCTGATTCTTCATTTGAACCAGCTCCTCAAATAG

GACCTGCATCCTGGGC---TGATAAT---GCTACACTTACAAAAG---TTGCAGGACGAG

CTTTAAAATCCACAACCCC---TCGAAAACCTTGTTATGGATCTTATGCTGCCCCTACAA

ATAAGCGAGGAGGACAAGGAA------------------CATCTGA--------------

----------------AATAGTAAAAACTTATTTT---------------GTAAAGGGAA

CCTCTGCTGCAGCAG------CTGACACTGTTCTTTACACAGAGGATGTTGCTCTTGACA

CACCTGATACTCATTTAATTTATAAAGTTAAGGATGCAGAAGCTGC------AGGAATAA

AAGGTCTTAGTCAACAAGCTAGTCCTAATAGACCAAATTATATAGGTTTTAGAGATAATT

TTATAGGTTTAATGTATTATAATAGTAGTGGAAACTTAGGTCTTTTGGCTGGTCAGTCTT

CTCAACTAAATGCTATTGTTGATTTACAAGATAGAAATACTGAATTATCATATCAACTAA

TGCTAGCTAATACTGTGGACAGACATAGATACTTTACTATGTGGAATCAAGCAGTTGATA

ATTATGATCCTGATGTAAGAGTAATAGAAAATAATGGTGTTGAAGATGAAATGCCAAATT

ATTGTTTTCCTTTGTCTGGAATGACCACCACTACTGCTTTTGCAGTTGATAGTAGTGGAA

A---------TGAACA------------------TAAATTAAATACAAAATCAGTAGG--

--AGAAGTAAGTATTGGTTATGGCAATATAGGATGTTATGAAATTAATTTAAATGCTAAT

TTGTGGCGATCGTTTTTATATTCTAATGTAGCCTTGTATTTTCCTGATGAACTCAAACTT

-------ACTCCTGAAAATGTAGAACTG--CCTGAAGATAAAAA---TACTT---ATGCA

TATATAAATGGTAGACTGCCTCCTGCTGGAGCTTTAGATGCTTATATAAATATAGGATCT

AGATGGTCAATTGAAGTTA-TGGACAATGTTAACCCATTTAATCATCATAGAAATCCAGG

ACTAAGATATAGATCTCAAATTTTAGGCAATGGTAGAATTTGTGAATTTCATATTCAAGT

TCCTCAAAAATTTTTTGCTATTAAAAACCTTCTTTTGCTTCCAGGAACTTATACTTATGA

ATGGTCTTTTAGAAAAGATGTTAATATGGTTTTACAAAGTACATTAGGAAATGACTTAAG

AAAAGATGGTGCAAGTATTACATTTGAATCAGTTAATTTATATGCTAGTTTTTTCCCAAT

GCATCATAATACAGCTTCTACATTAGAAGCTATGTTAAGAAATGACACTAATGACCAATC

ATTTAATGATTATTTGTCTGCAGCAGGAATGATTTATCCTATTCCTGCTCAAGCAACTCA

CATACCCATTTCTATTCCATCTAGAAATTGGGCTGGATTTAGAGGATGGAGTTTTACTAG

ACTTAAAAGTAAAGAAACTCCAGCTATAGGATCTCCTTTTGATCCTTATTTTAGATATTC

AGGGTCTATTCCACTTCTTGATGGTACCTTTTATTTAAATCATACTTTTAGAAGACTTCA

AATTATGTTTGATTCTTCAGTTTCTTGGCCAGGCAATGACAGATTATTAAATCCAAATGA

GTTTGAAATAAAAAGATATATA------GATGGAGAAGGGTATACAATATCACAATCAAA

TATGACAAAAGATTGGTTTATGGTTCAAATGTTAGCTCATTATAATATTGGGTATCAAGG

ATATCACTTACCAGAAGAATATAAAGAAAGGTCGTTTTCCTTTCTAAAGAACTTCACTCC

TATGTGTCGACAGGTTGTTGATTCTAG----TAAAA--ATGATAAATTATATA--ATA-T

TGCTTTAGGTGATAGA-----------------------CAT-------AATAATTCTGG

TTTTAGTGGTTTTGGTGT---GGGAGCAGCTGCCAGATGT----GGTCACCCCTATCCTG

CAAATTGGCCTTATCCTTTAATTGGTGAAAATGCAGTA---------GTAATGAAAACTG

AGAAA--AAGTTTTTGTGTGATAAAACTTTGTGGCGGGTGCCATTTTCTTCAAATTTTAT

GTACATGGGAACTCTTACTGACTTGGGACAAAATTTGTTGTATGCCAACGCAGCTCATT-

CTTTGGATTGTACATTTGAAGTAGATGTTATGGATGAGCCCACTTTGCTCTATGTTTTAT

TTGAGGTGTTTGATGTGTGTCGTGTACACCAACCCCATCGCGGTGTAATTGAAACAGTGT

ATTTAAGAACACCATTTGCTGCTGGAAACGCAACTACATAAACT--------A-------

------------------------------------------------------------

------------------------------------------------------------

---------------------------------------TGGGATCTTCAGAATCTGAAT

TGCAAAACATTATTTTTGACTTAGGTGTAGGGCCATATTTTTTGGGCACTTTTGATAAAT

ATTTTCCTGGGTTTATTC-ACAAAGAAAAATTATGCTGTGCTATTGTTAATACAGGTAGC

AGGGCTAGTGGAGGAATGCACTGGATAGCTTTTGGATGGAATCCTAAAAATTACTCATTT

TATATGTTTGAGCCTTATGGATTTTCTGATGAAAAGTTAAAACAAATATATAATTTTCAA

TATCAAAAGTTACTTAAAAATAGTGCTATTAGTTCATCTCCTAACAGGTGTGTAACTTTT

ATCAAAAGTTCAGAAACTATTCAAGGTCCTAAAAGTGCCGCATGTGGGTTATTTTGTGTG

TTTTTTTTAAAATGTTTTATAACCTACCCATCTTCGCCAATGAA---AAATCCATTAATT

GATCATTTTACTGCAGTGCCTAATAATAAATTAATGG-ACCCAAGTGTACAAC-CAATAT

TATATAATAATCAAATGTACCTTTATAAATACTTAGAATCAAATTCTTCCTTTTTTGCTA

AACATGCAACCCAAATAAAAAGTAACACAGCTTTTGATAAAATTTAA-------------

-AATTTACAATTTATTGAAA-TAAAGATTATAAATTTAAATGAAATTTCTTGTGTAAGTA

CAATTTTTT-----------------------------GTGATTTAAAACAAACATTCGT

CATCATCATCA-----------TCACCAATG----------------GGAAGAACAGTAG

TTTGAAATTCATA-----ATTTTTACACCATTTGAATTCGGGGATTACCACAGGTGCT--

----GGCACATTAAAATAACTCA---CCCAA-----ATTTGCTTTGCTAATTGCAAAGCT

GTCATAACATCAGGTGCACTTATTTTAAAATCACAATTTTTATTGGCCCTTGC---AT--

-----TACGTCCA---GC------TGGA--TTGCAACATTGAAAAACTAGCAATGCTGGA

TTAGTCACAGTTGC---TTTAATTCTGTTATCAGTAACAGAAGATATATCAATGTTTGAT

CCCA--TAGT----AATTCCAAAAGGA---GTAATTTTAC----------AAATTT---G

TCTTCCAACC---------------------ATTGGTGCATTAGAT---GCAATATTA--

CAATCA-----CATTTAATGGGAAG-CAAGATTTTTTTACTT------GCATCAGGCATT

TTGGGATAACATGCAACTTGAAAA-------GCCATCATTTGTTTAAAAGCAGTTTCTGC

TTTTGTCCC----CTCAGTGTAAAAGACTCCACATGATTGGCCACA-------ATGCATG

CCACACATAGTGTT---------TGAATCATAAAAGCAACTTCTGGCATCAGAATTT---

-TTAATTTGGA--CAATTTGTTTT--------CCCCATTTATTGATA---AT----CTTA

GCATTT---TTATTATCAT----TAACTGCTCGTTGGCCA-----TTTTCACTTG-----

----CTACATCCATTTCTATCACCTGCTCTTTTGCAAC--------------CATAGGCA

GACCATGCAAACATTTTAATCCATCACCTTC---------------AGTACATCCATGTT

----------TCCACC----CAACACAT-----CCAGTAGGATTCCATT-----------

-TTGGAGTGCTTAAATTACAGGATTTTATAACAAATCCAAATAAAAATCTGCCAATCATT

GAG----GCAAAT----------------------GTTTTGTTAGTAGTGTAA--CTTAA

AGTTAC-----------ATGCTTTTTTTTATCATTAATCCAAGCTTGGGCCAATTTTTTA

AAACATTCATAAGTACCAGCATC------AGCTAGCATGGT-------------------

---------AAGATTTTTAATATCCACTT----TTAGTGGA--GTCATCATTTGAACAGC

TTTTTCCAT---AGCTTGTTGCCAAT----------------------------------

----------------------------TTACTTCACCCAACTCCTCAGCAGA-------

------------------------------------------------------------

GGGCAGTATTGGCA-----------------------TTTGAGGATCA-GGAACATTAA-

--TTTTAGGCCTTTTAGGTGGCTG--TATAAGCAGTTGGGAATCTTCAATATCGGAGTCT

TC---------------AATTATAAGTCTGCGACG---AG--------------GAATTG

TCTTT-------------------------------------------------------

-------------------------------------------GGTT-------------

-----TTACTCCATG--TTCATAATCT---------------------------------

------------------------------------------------------------

----------------------TCGTCGCCGC--------------------TGCTTGCA

TACATTT------------------CAGCAAGGTTTTGTGCACT----------------

-----CATTTTTTTC-------------CTCCTAGATGGCAGAGGATCAAAGCTCACAGT

CAATTGATG--------------------------------------------------T

GAAAGATGCAGCGG--------------------------CGACAGAAGAACCTATT---

----------TCTACTG-------------------------------------ATACTT

TTGAA-------------------------------------------------------

------------------------------------------------------------

------------------------------------------------------------

------------------------------------------------------------

------------------------------------------------------------

------------------------------------------------------------

---GAAC-----------------------------------------ATGCCTATTTAA

AAGATGATATGC------TGATTTCTCATATAAAAAGACAAAGTACTATTTTGTCTGAAT

CTTTAAAAAATAGTTCTTA-TTTGCCTATT-TCTATTGCTGAACTTAGTTTACTATAT--

GAAAAAAATTTGTTTTCTC-------CTGACACTGTTCCAAAA-----AAACAAGAAAAT

GGAACTTGTGAAAATAACCCAAGACTTAATTTTTACCCAACTTTTGCTGTGCCAGAAGTT

TTAGCTTCTTATTATATATTTTTTCAAAATTTT-AAAATTCCAATATCATGCAAAGCAAA

TAGAGCAAAAGCAGATAAAATGCTTCAATTAAAACATGGAGATTGCTTACCTGGATGTCC

TTCACTGGAATCAATTCCTCGAATTTTTGAGGGACTTGGACAGGATGAAATTCCTGCTTC

AAAGT---CTCTGGA------AAAAAGTGAT---------------AGTGTATTAATAGA

ACTTGAAAATGACAACCCAAGAATTTCAATGATAAAAAGATCTACAT---CTTTAACTCA

TTTTGCTTATCCTGCTGTTTCATTGCCTCCTAAAATTATGGAAAGTGTTATGAATAGTTT

A---------TTAATAAAAAGACAGCGCCCTCAAAC---------GGAC---AATGAAGA

A---------AATACCCCTTTTCATGCAGTGTCAAATGAAGAATTAGCTCAATGGTTAAA

TTTAAATGATCTAGA---TCTTAGTAAAA------------TTGAAGATAAAAGAAAAAC

TATGATGGCAGTAGTGTTAGTAACACTTAACTTAAATTGTATACACAAATTTTTTGCAAA

TCCAACAATTATAAAAAAACTTGAAGAAAACCTACACTATATGTTTAGACATGGATATGT

TAAGCAAGCTTGTAAAGTTTCAAAGGTAGAACTTCCTAATATTATTTCCCACACTGGAAT

TTTACATGAAAATAGACTTTCTCAAAG-TGTTTTACACAATACTTTAAAAGGAGAAGTTA

GAAGAGATTACATTCGCGACACAATTTTTTTATTTTTGATTTATACTTGGCAAACTGCTA

TGGGAGTGTGGCAACAATTTTTGGAAGAAGCTAATCTTAAACAACTTGAAAGAATTCTAC

AAAAACAAAAAAAATCTTTATGGTGTGGGTTTGATGAAGTAACAATTTCAGAA-GAATTA

ACAAATATAGTT-TTTCC--TGACAAACTTTTAAAAACTTTACAAGAAGGGCTTCCA-GA

TTTTGTAAGCCAATCCATGATGCATAATTATAGATCTTTTATTTTGGAAAGATCTGGCAT

AATTCCTGCTTTGTGCAATGCTTTGGTCACTGATTTTATTCCTTTAAGTTATAAAGAATG

TCCTCCACCGTTATGGGTATATACTTATTTTTTAAATTTAGCTAATTATCTAATGTATCA

TTCAGATGTTGCA------TATGATGTTACTGGTGATG------GT---CTTTTAGATTG

TCA---TTGCAGATGTAATTTATGCACTCCCCATAGATCTATGATGTTTAACTTGCCTCT

TTTAAATGAAATGCAAACTATTGGTGCATTTGAATTTCAAGGTCCTTCAGTTAATG----

GACAACCGGGAAAAAGC----T-TAAAGCTAACTCCTGGATTATGGACCTCAGCTTACTT

AAGAAAATTTGAAGAAAAAGATTATTATCCCTTTAAAATTAATTTTTTTGAAGAAC---A

AAAAAATAAACCTAAGGCTGAACTTACAGCTTGTGTCATCACGCAAGCCACTCTAATTAA

CCAATTACAAGAAATAAAAAAATCTCGTGAAGATTTCTTACTAAAAAACGGTAGTGGGGT

TTATTTGGACCCTCATACAGGAGAGCGGCTTTCCGGAG----------------------

-------------------------------------------------ACACAAC----

--CACTGTGAACA-----------------------------------ATGAAAGCTCAA

GACAAGGCCCCAAAACCTCAAG--------AAAACATGGAGG-------TA---------

------------------------------------------GAAACCATAAGTTCTGCT

GATGAGAACAG-------------CCAAAGCAG--CGAAGCCAGCCTAGATGCT--AATT

ATTCTCAAT-------CAGAAGAGT-----------------------------------

-----------------------------------------------------CTCAACC

AAAACCT--------------------------------------------AATAGATGG

GATATG---------GCTCCTGG----CGGTAAG--------------------------

-----------AAAAAATATAAAAGCTGGAGGGAACTTAAAAGTGAAATTTTGACCTGCT

TGCATGAAACCAATTGTGATGTGGGATTTACCCGTCGCTATATTCTTATGCATCATGGAG

TGGCTTTGCCCAAGAA-TATAATTTACTACTATGC-----CTCTTGCTACA---------

-----------------------------------------GAAAA--------------

--------ATTCAAAAAACATCAGAATCA-------------------------------

--G--AATGAAG--------------ACTCTATGATAACTC---AACAACTAAGAGAAAG

TATTTTTCCCACCCTTTATGCTATTTTTCAACAAAGTAGAGGAAAAACTACCTGC-----

----CTTAAAGTCAAAAATCGAAGTTTAAGATCTCTTACCCGCA-GCTGCCTTTATCACA

AAAAGGAGCAGCAG-CTCAGAAGAACCCAACAGGACGCAGAACTTCTTCTTCAGAAGTAC

TGCA----------CTAGAACCAGAGCCGCGC-----------------------TTAAA

TA---------------------------------------------------ATGAGCA

AGGATA-TACCCACCCCCTACGTGTGGTCCTACCAACCACAAATTGGAACTGCAGCTGGA

GCTTCTCAAGATTA---TTCAACTAAAATGAATTGGCTAAGTGCCGGAAATTCCATGATA

AGATCAGTTAACAGAATTCGCAATCAAAGAAATGAAATATTGCTTAAACAAGCAATGATA

ACGGAAACACCAAGAAATGTTGCCAACCCGCCAATATGGCCTGCT--GAGGCACTTCATC

AACCATTCAAATTACCAACG-GAATTTCCTTTGCCGCGCAATGGCCCACTTGAGCACATG

ATGTCAAATAATGGAA--TGCAATTGGCTGGGGGAAAAGC--------------------

----------------TTATA---------------------------ATCTAGTGGGAT

GTGGAGTTCAACTACCAGAATCTTATGCACC-TTTGTCTGTG----------CGGTCAGA

CGGCATTTTTCAAC--TTGGAGGCGGATCAAGAGGACATATAAATCCTCAACATC---AG

TGGATTTTGCTTCAATCTGGATCTAACAAACCACGCTCTGGCGGTCTTGGATCTGCTCAA

TTTGTG-GAAGAATTTGTTCCCACGGTATATTATA-ATCCTTATTCTGGACCCCCAACTA

ATTATCCTGAAC---AATTTA------TATATAATTATGATGTTGTTGAAGATCGAGTAG

CCAACTATGACTGATATTATTATGGAAATTGCATCTGCTGAAACCGCGCGCATGGATGAT

GTGGCGGAACAACAAAGATTAT-TAGAAGAAGCTAA-CAAAAATATTCATTTTAAAAAAA

TGGACGAATTACAAAAAC-------TGCAAAATAATCATCGATGTGAAAAAGGAAGCTTT

TGTGGAGTTAA-GCAAGC----GGTTTTTGACTGGTATCATGTGGAAAATGATCATTGCT

TAAAATTTCATGTGGGTTCT-ACAAAAAGATCTACAATTATAGATA--ATGTAAAATGTC

TAATTAAATA-CAAAATAATGGATTATGGATATGCTGGAAAATTTATTTGTGGATGTACC

A---------ATCAAGATTGCTGTCCTGATTTTATTCAAAATATTTGTAAAATAGATTCT

-------TTAAGATAAATAAAAAG-TTATACTTACCTTAACTGTGTCTGAATTTCTTTTA

TGCGTTGATTCCCAAAAATTTCCACCC----AATTTCCTTCTTCAAAACTTTGATAATTT

-----------AAATTATACTTAATAGCAAATTTTCTAAAATCTTTAAATGTAATTTTA-

--TGTTTCAGCATATGCCCATTATATAACAAAATCATGGCGAAGAGAAAAGCAGA-----

----------------------AGATATCAACCTCGTCTATCCT---TTTTCAGCAAAAA

GAATTACTATAATTCCCCCTTTTTATAGTATA------------------AATGGATTTA

CAGATGAG---------------GGAGGAGAATTGATGCTTAAAACAGCTCAACCATTGG

ATTTTACAAAAGATGGAAAGCTTCAAATAAAAATGGGCGTAGGACTTACTTTAGGAGCTG

ATGGTTCGCTTCAAGCTCCAGGAGACAGCAATGTAACTGTTGC--------CC---CACC

TTTACAAAATATAAAGGGAAATGTGTCAG-----TTAAATTGGGGGAAGGAACTATTA--

--TGAAAGATGATGCT---TTAAGTGTTGATGTCCAATCTCCCTTACAAATTTATAATTC

AAAAGTTGGAT-TAAAAACAGATGA---TAGTTTAGAAGTAAAAGAAAATAAACTTTGTG

TAAGTTTAGACAGTCC-CTTACAACTAACAGAAAATGGTATTTCTATTAAAA--CAGGAA

AGGGATTAACTATATCTGATAATAGTTTACAAGCAGACCCTTATGTTGGTGTTAGGCCGC

TATATACAAGTGG-------AAATCAAATTTACTTAAATTATGGCCAAGGATTATTTTTA

CAAGATGAAAAAATTGAGGTTAAAACAGCTGACCCTATTTCATTGGATCCAAC------T

AAGGGTATTACTTTAAAAATAGGAAATGGGTTACAAATTAATAATCAGGGTGCAATAGAA

GCATCCGCAAGTAATTTAAATATTAAAGAACCTCTTATAGTGGAAAATGGGAAAGTAACA

CTGGATACTGCAT-ATCCTTTATATATAAAAGATGGAAAACTAAGTTTTAAACCAGGAAG

AGGGGTTAAAATTGTAGGGGACTCACTTGAATTAAAAATAGGATTTGGTTTAGGTTTTGA

TTATTCTGGTCTGGTG---TTAAAAATGGCAGCGCCATTATATGCAGATGCTTATG-GCC

TTCATGTAAGAACAGGGCCTGGAGTAGAAATTAATAGTAATTACATCCAAGCTGCTTGTG

AATCCCCTATTACCTTAAATGCTAAAGGAAAAATTGCT-TTAAGTTATGGAAGTGGGTTA

GATATTAATGCTAC---------------------------TAATCAACTTATTATTAAA

AAAAGCTCAGGACTTGCTTTTGAAAATAATGCTTTAACATTGGATTT---AGGATTAGGT

ATGGAATTTGAAGGAAAT--A-------------------CTATT-CAACCTAAGTT--A

GGTTTTGGTTTACTTTTTGATAAAGAAAATAAATTATCTACTAAAG--ACTGTGGATTTT

GGACAGGTCCCA---------TTCCCAAACCAAATGTATGGG-GAAATCAGGGTATTTTT

AA-TATAAGTCTCACTAGATTAGGACCTAGTGTGTTTGGAAATT----------TACAAA

TTACTGGAAT---------TGAAAGCAAAAAAATGATAA-----------CTGGAGAAGA

TGAAGTTTTAAGTATTGCATTATATTTTGACCAAA-----------ATGGAGAACTATGT

GAAAATAGTGATCTTAAAACTGCTG---ATTGGGGGTGTAAGGAAGGCAATCAAGTAACC

TCTAACAAATCCAACC--CCACTCATAATAAATTATTTATGATGCC---------CAATT

CCAAAG-CTTATCC--GCCCACGTCCAA--ACC--------TGCTTTAACTAATAGAATT

TTTTTACCCACTAGTGTATATGATGA----------AAAAGATGAATTT-----------

-AAAACAATTATGTGTAAAATAGGGTTAAATCAGGTACCT--------GCAAATGCTAAT

AAAGAAAAATATGG-------------------TCTTTTTATT--ACATGGGGACCTTTT

TTTAAAACACCTGTTGAAT-TTAGAACTGGAGTTGTAACCTTTAGTTATTTGGCCGAGGA

AATTGAATCTGAATAAATCACACCA--ATTTTAA---TAAAATTTATTTTTTATTAATCA

AGCTTGCGTATTACAAT-TCTTCCGCCATCAGAATTTTTTATTACA---CTAATACTGCC

ATTATCATTAATAACAGGTTTTTCATCAAC--AAAAATGTAAATGTCAGACCCAATCATA

-GTACCTTTAAATGTAAT-------GTTTAAATACTTATGTAAATATCCACAATTAGCGA

TGTCTATAAATTTATCAGGAA------------------TATCCTTTTGACACACTGTGA

AAGCTTCATAATCCTGCAAATTATTGAGGAAAATCATCAAATGTAAAA------------

----------------------TAGCAATTTTTAAACATTAATTTTTC-----TAACAGT

CT-ACAACG-----TTTAATTTCATGATATTTATCATCTCTAAAGTAAGACCTATGAAGC

CATTTCATGTGACTTAA-------------------------AAGCTTAAGCATAATTTT

TTTAGT---------GTTTCGCATACAACTACAGGCAGCAATTTCTGTTAAAGATACACA

TGAGGTGCACAGTAATATAACTTTACATGTTGTGCTGTTAAAATAATGATTAGTGTCTCT

AAAAAAAACTTCA--TATATGTCCTTAG-ATCTTTGGTCTTGCGAAAGC---AGCTGAAT

GTAGA------------------TTAAATGCTTGCCTCTAAAAAATATACTTCCTTCATA

TCTAATGATTTCAGTAGGCACAGCTGTGTTAACATAGCGTCTATAGAA-----AGGAAAT

TTTAAATTGTAATTAGATCCAATGATTATATCCTCAAGTAAATGCTTTTTGATAGCAGCG

CCAGCA--AGACATTGTAATGA----------TCCCTTTTTATCGCAGTGGCAGTGAACA

TGCCAAGATTCAAAACCAGATACTCTCCATTTAACATAGCAATCAGTTCCACAGCATTTC

CAGCAGAGGTCTAGCAGGTG--ACTATATAGATGAAGCTCAAATTTTTCTAAGATTAATT

TCCAAGGCACAGGCAAACCATAATTAACCCAAA-----AACAAGCAGCAGA-TGGTAAGC

TTGTTACCTCGGCCACAATGTGGCTATCTCC---AACACAGGTATCCATTCCTAGGGAAG

---TGCAAG------------AGCAAAATTAACACGTTGAACTGTTGGTTCTGGTTCTAT

GGCCCCTTCTGGAGATTCTTTTA-AATTTGATTGATTTGGAT---GATTTTCAGCAC---

--CGCGGCTTCTTTT-------T-------------AAGCCAGAGCGCGAAAACATGGCT

TGAGTTTTTAGTTGTTGAACATAAGATCGGGT-----------------G-TCAGCATTA

ATTTTACTCCACTT--TCGAACAGCCTCCTTTA-------TTTCTTCCACTTTCTCGGGA

GTAAATTCAGCCAACTTCAAATTGAAGAATTCAATTACTTTGACTTTAGTTTGGT-AAAC

AGCAGCTCGCTGTAGGTTGAAAGAAAATACTCTGCATTGAA-------------------

----------------CCTCATCCATGAGTTCAAGCAGTTCATCGCATTGATCGTGA---

-------ATCTCAAGAAGG----------ATTTTGAAAGTGATTTTATCACTGAAATACA

ACATGACT-TACTTCAGGGCAAAGAGATTGTTCAGTGGAGTAATCACCAGGTCTTGAGCA

ATGGTGTCAGAGTTGTAACCAAATCCACAAG--AACCAGCTGTTAATCTAAA--AAAGGA

CAAAAGAGATCTCTTAGCCAAAGATGCAA-------ACGCAGCCGGATGAGCAGATTCCT

CAGC-ATCATTAAATCGATGATCACAGGCGGCCATGGACAAAATGT--AGACCAGATGCT

TTTTATTGGAGCGCACTAAAGTAA--------GGCCCACATAACCATTTGCTCTTCTGAA

ATTTTCCTCCA-AATAGCGGTAAGTATGTAAGTACAGTTCAGTTTCAACTGATTTTAGGC

CAATAGCATAAGTATCATG-CTGCATAAACTTCAGGATTTCTCCCTGAATTTC----CAC

TTTCCAAAGCAAACAGCAATTCATTTTTGTAGGCCTAGTGATTAAGAGCACAGAAT----

------------TAAAAAATAAATCTAAAAAATAAAAACTTTTTCCCCACCAAGTATAAA

GAAAAAAGACTTACCAAAAAACTAGCAAAGTCCAG----CACA-G-AATGCAAAGCGTCA

AGT-AATTCC-AAGATAGGTGTAGCAGCCTGATTCTCTCCGCAGAAA-GAATAC--TGAG

AGCAAAATCTGACTTGCTTTTATACAGGGCATCTGAAAAA---------AAACAGCAGTT

AG--------TGGAAAAGCACTGGAATGTAAAA-AATATTGCATAA--ACAGCAGCTGAA

AGTCA--TTAAAAAAAGCCCGGGAAAAT-ATTATCTTTTTTGACCAAGTGC----CAAGA

A-----TGTGAAAAGCCATAAAAAGTGCTGATTGCAAAATGTTCTCAAACACT-GCAAG-

------------ATGGAGCCCGGAAG---GGTAAAGTCTCTGTG----------------

-TTTAAAGGAAAACGGCACCTTTTT---AGAATTAAATGAG----TTTTTA---------

-------TTTTTATATGGCCCTGAAAAAAGAGACGTCTGTGACTGCGAGTACTGCAGCGA

GCTGTATGTGCACCCAAAAATTATT-------------TTCA-----------AAAACTA

CCACGCAGTCAAA-------AAAATCACCCG-------CCTAG------------TGGAA

GGAGAAGAAGGAGGATTTGGACAC------CAGAACACCTTCTACGCGCCAAAACTGGAC

----CCCATGTGCATCCCAGA--ATTCAG-AGTGTATGGGCAAAAGCTGAGCGAAACGC-

-CCGCAGTGAAAAGGGCAGCTCTAATCAGAAAAATGCCTTACCTCAAAAAGCGGCGAGA-

-AATCC-----AGCTGGCGGAACAGAAGGC--GGAAATTGACCGAGGAAAATCGGGGTA-

---CTGCTGCTGTGAGAAATGCGAAAGCCAAAAAGGGGAAAACTGAA--------AAAGC

GGGGAAAGCACAAAAAGGGGAAAAAGCGCAAAAAGTTCAGA---TCAGGTCAAAAA--TC

CGGTCCCACACAAAAACGGCGCGAA--AACAGAGGAAATGACATAAAGGACATGACGCAA

AATCAAAGGGCTTGCCA-----AGTTCCGTGGAAAA-------TTCCC----AGGGTCAA

AATGGCCGACAGCGGAAATCAAAAAACGTCACTT--CCCTTCCCACACT----CCACCCC

ACCCCACCCCTCCCACTCAAAAAACACGCCTCTTTT-GGCGCCAAAATTT-----GTCCG

GTGGTATATTGATGATG-------------------------------------------

------------------------------------------------------------

------------------------------------------------------------

------------------------------------------------------------

------------------------------------------------------------

------------------------------------------------------------

------------------------------------------------------------

------------------------------------------------------------

------------------------------------------------------------

------------------------------------------------------------

------------------------------------------------------------

------------------------------------------------------------

------------------------------------------------------------

------------------------------------------------------------

------------------------------------------------------------

------------------------------------------------------------

------------------------------------------------------------

------------------------------------------------------------

------------------------------------------------------------

------------------------------------------------------------

------------------------------------------------------------

------------------------------------------------------------

------------------------------------------------------------

------------------------------------------------------------

------------------------------------------------------------

------------------------------------------------------------

------------------------------------------------------------

------------------------------------------------------------

------------------------------------------------------------

------------------------------------------------------------

------------------------------------------------------------

------------------------------------------------------------

------------------------------------------------------------

------------------------------------------------------------

------------------------------------------------------------

------------------------------------------------------------

------------------------------------------------------------

------------------------------------------------------------

------------------------------------------------------------

------------------------------------------------------------

------------------------------------------------------------

------------------------------------------------------------

------------------------------------------------------------

------------------------------------------------------------

------------------------------------------------------------

------------------------------------------------------------

------------------------------------------------------------

------------------------------------------------------------

------------------------------------------------------------

------------------------------------------------------------

------------------------------------------------------------

------------------------------------------------------------

------------------------------------------------------------

------------------------------------------------------------

------------------------------------------------------------

------------------------------------------------------------

------------------------------------------------------------

------------------------------------------------------------

------------------------------------------------------------

------------------------------------------------------------

------------------------------------------------------------

------------------------------------------------------------

------------------------------------------------------------

------------------------------------------------------------

------------------------------------------------------------

------------------------------------------------------------

------------------------------------------------------------

------------------------------------------------------------

------------------------------------------------------------

------------------------------------------------------------

------------------------------------------------------------

------------------------------------------------------------

------------------------------------------------------------

------------------------------------------------------------

------------------------------------------------------------

------------------------------------------------------------

------------------------------------------------------------

------------------------------------------------------------

------------------------------------------------------------

------------------------------------------------------------

------------------------------------------------------------

------------------------------------------------------------

------------------------------------------------------------

------------------------------------------------------------

------------------------------------------------------------

------------------------------------------------------------

------------------------------------------------------------

------------------------------------------------------------

------------------------------------------------------------

------------------------------------------------------------

------------------------------------------------------------

------------------------------------------------------------

------------------------------------------------------------

------------------------------------------------------------

------------------------------------------------------------

------------------------------------------------------------

------------------------------------------------------------

------------------------------------------------------------

------------------------------------------------------------

------------------------------------------------------------

------------------------------------------------------------

------------------------------------------------------------

------------------------------------------------------------

------------------------------------------------------------

------------------------------------------------------------

------------------------------------------------------------

------------------------------------------------------------

------------------------------------------------------------

------------------------------------------------------------

------------------------------------------------------------

------------------------------------------------------------

------------------------------------------------------------

------------------------------------------------------------

------------------------------------------------------------

------------------------------------------------------------

------------------------------------------------------------

------------------------------------------------------------

------------------------------------------------------------

------------------------------------------------------------

------------------------------------------------------------

------------------------------------------------------------

------------------------------------------------------------

------------------------------------------------------------

------------------------------------------------------------

------------------------------------------------------------

------------------------------------------------------------

------------------------------------------------------------

------------------------------------------------------------

------------------------------------------------------------

------------------------------------------------------------

------------------------------------------------------------

------------------------------------------------------------

------------------------------------------------------------

------------------------------------------------------------

------------------------------------------------------------

------------------------------------------------------------

------------------------------------------------------------

------------------------------------------------------------

------------------------------------------------------------

------------------------------------------------------------

------------------------------------------------------------

------------------------------------------------------------

------------------------------------------------------------

------------------------------------------------------------

------------------------------------------------------------

------------------------------------------------------------

------------------------------------------------------------

------------------------------------------------------------

------------------------------------------------------------

------------------------------------------------------------

------------------------------------------------------------

------------------------------------------------------------

------------------------------------------------------------

------------------------------------------------------------

------------------------------------------------------------

------------------------------------------------------------

------------------------------------------------------------

------------------------------------------------------------

-----------------------------------------------------

>Bat AdV-G

------------------------------------------------------------

------------------------------------------------------------

------------------------------------------------------------

------------------------------------------------------------

------------------------------------------------------------

------------------------------------------------------------

------------------------------------------------------------

------------------------------------------------------------

------------------------------------------------------------

------------------------------------------------------------

------------------------------------------------------------

------------------------------------------------------------

------------------------------------------------------------

------------------------------------------------------------

------------------------------------------------------------

------------------------------------------------------------

------------------------------------------------------------

---------------------------------------------------CATCATCAA

TAATATACAGGACAAAGAGGTGTGGCCTAAATTTGGCGG-GCAGCCTAGAAATT------

GGGGGTCCC-----TGGTGGTCTCTGTGAGCCCCTTCCTCATTTTTGGGTGTGCGTGCGA

CGAGAGCGCGC--GGGGAATT-TTACGACG-GGGGCGTTCCGCGGAAAGGCGGGTGATTT

TCCGCCGTAAAGTTTTATGAGGGGCGGAG-------ACCGAAACCGGGGCAGGTG-GCCA

GCGGGCGAGGCGCC-CACCTTGTCGAAAGTCCGCCAATTACAGTGAAAATTAATTGCCCT

TTCGTGT--CTGGGAGTACTGCCAAATGT-CTGGGCAAAAGTCCCAGAAACTCCGCCTGT

GTTTACAGTTACTC-----ACGGCTGCCAGGTAATTGTGG--TTTTCTGTTGGCA-CAGG

GCTGGACCGCAGTTAATCATTATTTTTTCCGGGAGTGAGGTTTAAATATACCTGCGCAGT

CGAAAGAGGACT-----ACTCTTGAGTGCGGAGCGA-GTAGAGTTTTCTCCTTTCG---C

TCTAATTTT-TCTCTGAAATGAAGCTGTCCGTGCTCGCTTCGACTGTCGATCTGG---A-

--GGACT---TTGTCGCTGACCTGCTCGAGGAGTGGAGACCCGA----------------

TTTGCCGCCGTCTCGCAGCCC--------------TTCTCCAC-CGACTCTCCACGATCT

CTACGATCTCTCTCCGTC----------------------------------------TC

CGC------CTTCACCCGATGTGGACGT------------GGTGGTTAT-----------

TGACAGTGAGTCCGATACTGACACTGCTCCTGAGGCCTGCTCTCG-------TCTGTCTA

CTCCTGCAGTCTCTCCTCTGCCTCCAATTTCTACTTCTCCCGC---GGCTTTAACTGAGG

ATATGTTGCTATGCCTGGAAGAAATGGCGAC-------TTTTGATGGAGGGGATGAGGTT

CGCAGCGAGACAAGCTCTTTTGAGCGTTG--GGGCGCCTCTTTTGAGCCCGATCAGGGCC

TTCACTTTGGCTGCCTGAGAT-------GTGCATTTTTCCAAGAGCGTGGCGAGTCCAGC

------ATTTGTGGCCTGTGTTACCTGAAAGCTCTTTCTGA----GGGTAAGTTTG----

-TGCCGTCTTATTTTTACTTTAT------------TGAGTGT------TTTTGTATTTCT

-------GGGTGC-----------TTACATACTCTTTAT---TT---TTAGTTCCTTTTC

AGA----------------TGCCCTCGCGCCCCGGACCTCCTGCTGCCAGT------GGT

GAACTGAA--------GCGCCCT-----------TGTTCTACTTCCTCATC-TGAGAGCT

CTTTGTTTT-----CTGAGAATAAGCGTGCGT---------------GCCTGGATCCGGG

TGTTT---CTGCTCCACCTGCTG---------------CTGAGCAAACAGAACCTTTGGA

CTTGT----------------CAACTAAGCCTCGCCCACGCTAATCT-----TGTTCT--

--CAAATAAAGTGTTTATCTTCC-------------------------------------

---------------CTGTGATCAT-----GTGGTTATGTC-A----TCATTGCTGGATT

AGTA--TTTAAGTG------------CTCTGTGCCAAGCACTTG---TTACAGCTCTTTA

G--CTACAGC-----AATCTCAGCAGGTAG-CATGGATCTTTTC-TCTATGTGCGCCGAT

TA-TAGCTCTTTTAGATATAT-TTTAAGGGGTAGTCTCTGTGGCAGTAGTTGGGTTAGAA

AGTGGTGTTTT---CCTGC----TTTAGCAGATGCTATTACTGGCATTGCAGATGATTAC

GAGGATGGATACTGGAGAGCTTTTCCGCCAGATGATATTAACTGGAACCTCCTGAGAAGA

GGTTACACTTTTGCAGCTTTTACAGATTTGTTTAGCAGTGTGGACCTAGGCAATGCTGGG

AGGTTTATGTCTTTTCTGGGGTTTTTAAACTTCATTTTGCGTAACTGGCCTTCTGAT-TC

TGTAGTGCTTTCTGCAGATAGACTTGACTTAATCTGTGTTCCAGCATGGACGAAGATCAT

GCTATGGAGCCA------AAC---------------TATGAGGCAGATGGAAGAGCTGCA

GC-TGTTGGAAAT--------------------GGCGGCCGCGCAGGCAGCGG-------

----------CGGCGG--CCAGAGGGGCTCGGGCAGAAGAGACCAGCGAGACCACCC---

---------CGCT-GCTGCAGTCAACC--AGCACCCAAACGGAGCAGCTGGAAACGGACT

TGTAGAAAATGTTTTCTTTGTGTTGGACCCCTTGCATGTT------CTTCCAATGATTAA

TCCTGATCATTATGTAGTT----AG---TGGGGATCAAGTAGAGTTTGCAGCTATTAGGG

AAGATGAGCGCCTGAATGGACCTTCAGACAGGTGGAATTATTCCTCTGTAAAAACTGTCC

GC---GTAAATCCTGGAGATGATTTGGAGGCAGCCATCAGGTTGCATGCTAAGATTTCTC

TGCAGCCTGGAGGGGTGTATGAACTTAATAGGAATATCAGGGTAGTGGGAGCTTGCTACA

TTATTGGCAACTGTGCAGTGCTTAAAGTTAATTTGCCGCAGGGGGGCCCTCTGTTCACTG

TGTCACACAGC---GAGCCTATTCCAGCTATTGGTTTTATGGAAAGAGTATG-CTTTTCT

AATCTGGTGTT-------------TGAGAGTGTGGGATCAAGCAAAGCTATCTGCTGCGT

CTCTAACCGCAACATTTTATTTCACGGGTGTATTTTTTCTGGTGCTCATATGCTTTGTTT

A--GACATGCGGGCTGGGG------------CAGAAGTTCGGGGGTGTCAATTTTTTGGA

GCAGTCTGTGCTGTCCGGTCAC---GGGGGCTCTA-TAGCATGCGTGTTAAGAACTGTTC

ATT-TGAAAGATGTGTCTTTGGAGTAGTTGCTGAAACCAAGGTGAATATTAACCGGTGTT

TCTTTGGGGATTGCACCTGCA---GCATTAAACTTTCCACTGTAGGATCTGTGACAAATT

CCCAAGTGATTGTAACAAATAGACACCAGTCCCCTATGAATGTGCAGCTATGTACTTGTG

AGGA---CGGGGGTCATGTTCAAGCTCTGGGTAATGTGCATATTGGGTCC---CACTGTG

AGGCTCCGTGGCCCAAGTTTGAAGGCAACACTCTGAACAGAGTGAGAATGTAC-ATGGGA

AGGCGGCGCGGGGTCTTTCACCCGA---GGCATTGCTTGTTTGG-CCTCTCGGTCATAGC

AGCTCCGACCGGAGTGGCGCAGCAG---GTGTACC--TATACAGTGTGTATGACACTACT

TGCGGGATTATGCAGCT-------GTCTACCTATGAG---GGCGATGCCGGGGAAAGACT

GTGTACCTGTGGCGAGCGACACAGCACTCCATGTATGCGGGCTTCTTATGTCACCGAC--

-AGCAGAGTGAACCGAGACCTGAAC--TCACATGACACTGCTGAGTTTTCAAGTTCTGAT

GAAGACGAAGAA--TTTTAGGTAAGGG------------GCGGGGCTTCCCGAGA-----

---------ATATAAAAGGGAGGGAAATTGTGGCTA--GTTG--------CTACACGTCA

CCATGGACCC---------CCAACAGGAGGGAATTGTTAACACCTGTTTCGTGACCACTC

GTTTACCTTCTTGGGCAGGATCGAGACAAAATGTTACTGGATCAGATTTGG--AGGGAAG

ACCGGTGCCTTCCGAAA-----CTGGCCCAGCCGGACGCCCT----G-------------

-----CCCTCGCCTCGCGCCTGCTTACGCCTCTGGAAGACC-ACGCAG------------

------------------------CTG----------CTGTAAATGCC------ATC---

----------ATAGAAGAACTGAAAACCCAGGTAGCTGAGATGCAGACTTC--GGTGGAA

ACCATT---------------CAGCAAGAAGTAGAAG-------------CTTTGA----

-AGCAAGCCCACGCTACAGCTCC-----------------------------ATAA----

----TCATG----AATCAATAAACTTTATT--------CTTTTTT-GAATGATAATACCG

TGTCCAGCGTTGTCTGTCTGCTAACACTCTGTGGATTTTTTCTAAGGCCTGGTACAGTTT

AGCTTGAATATTCAAATACATGGGCATCAGGCCTTCTTTGGGGTTAA-GATACAACCATT

GCATGCAGTCATCAACAGGTTCAGTGTTATACAAGATCCAGTCATAGTTAGTATTGAGAC

GGTGATAATTAAAGATATCTTTCAACAGAAGAGAGATAGCAGTGGGTAGACCCTTGGTGT

AAATATTAATAAAGCGGTTGAGCTGGGAGGGGTGAACCTTTGGACTGATAATGTGCATCT

TAGCTTGAGTTTTTAAGTTAGAAATGTTTCCTCCTTGATCTCTGCGGGGGTTCATGTTGT

GCAAGACCACGATAACAGAGTAGCCTGTGCACTTGGGATACCTGTCCAGAAGTTTACTGG

GGAAAGCGTGGAAAAATTTGGCAACCCCTTTGTGCCCACCGAGGTCTTCCATGCACTCGT

CCATGATAATAGCTATGGGACCCCTAGAGGCCGCCTTAGCAAAA-ACATTGTTGGGGTTG

GTGACATCATAGTTGACATCGCTGGTGAGGTCAGCATATGACATCTGGACGAAATTTGGA

AGAATGCTACCGCTCTGGGGGACAACAGTGTTTTCTGGCCCAGCTAGATAGTTGCCCTCA

CAAATTTGGGTTTCCCAGGCTGCAATCTCTTGAGGGGGGATCATGTCCACCTGGGGAGTA

ATAAAAAAAACTGTTTCGGGGGCTGGAACAATAAGCTGAGCAGACATGAGGTTTCTCAGT

AGTTGTGACTTTCCACACCCTGTGGGGCCATACACTACAGCAATAAAGGGTTGTTTGTAA

AAGTTAAGGGAGGTACAGCTTCCATCTTTATTGAGA------------------AAAACA

TTAGCTTTTTCACAGCAGGTTTTAATACTATCATTAATAGACAGCAGTTCTTGCAAA---

-----------AGAGAGTCCCCACCCAAAGA-----------------------------

CAAAAGCTCATCAAAGT-TGGAAAAGTTTAAAAAAGGTTTCAGGC----CTTCTGCATA-

----AGGCATGTTTTTT-----AGAGTAGTTTGCAAAGTAAGCAATTTGT-------CCC

AGAGTTCTCTCAATTGG----TCCACGGCAGCTCCATCC----------AAGTTGTTTCT

GTGTTTCTTG--------GATTTGGGAACCTCTTGCTGTATGGAA--CCAGACGGTGGGT

GTCC---------------------AGGGGGTGCAGGGTTTTGTCTTTCCACGGTCTTAG

CTTCCTTGTAAGGGTGGTTTCTGTGACCGTGAAGGGTTGCACGTGGGCCTGAACGCTGGC

CAGTGTGCGACGCAGGCTCATCCTGCTGGTCTGGAAGAGATC------------GTTTCC

GTGTTGCAAGTCAGCCAA---GTAACAGGCCTTAAGCACA---TCATAAGAGAGGGTGGA

GGTAGCATGCCCTTTGGCTCGGAGCTTCCCTTTTCCCACG------TGACCGCACGTGGG

GCAGGTAGTATCTTTCAGAGCATACAGCTTAGGAGCTA-GGTATACTGATTCTGAGCTGT

AGGCATCCCCTCCGCACTTTTCACACTGAGTTTCACATTCAACTAACCATGTTAGCTCAG

GTTTGGCTGGGTCAAATACTAAGCTGCCCCCATTTTTTTTGAGTCTTTTCTTACCTTTGG

CCTCCATGAGTCTGTACCCTTCCTCGGTGCTAAAGATGCTGTCCGTGTCCCCGTAGACAG

ATTTTAGAGGGCGCTGATCCAGGG---GCGTGCCAGCGTCATCCTCGTAGAGAAACTGAG

ACCATTCTGACACAAATGCCCTGGTCCAGGCCAAAACAAAGGAGGCAATTTGAGAGGGGT

ACCTGTTATTGGCTATCAGTGGGGAGGCCCACTCCAGGGTGTGGAGGCAGAAGTCATCAT

CCTCTGCATCTAGAAATATGATTGGCTTGTA---AGTGTATG--ACGTCA--------CA

TTACCGTGA-----------GAAATAAAAGGGGGTTCGGGGTCATCCTTCTCCGC--ATT

GCTCGTGGACGGCGGCGGAGAGGGCG-----------GAGC-------------------

------------------------------------------------------AGCGG-

CAGGTGGGTACGCTACGACAAA---TTCTGGCATGATTTCTGCGCACAGGTTAT------

------------------CTGTCTC----------TATGTAAGAAGAAGAT--------T

TGACTATGTAAGATCCGGAAGCAATTTCTTGTGACGTGTGGGGGTCAATTTGGTCAGAAA

ATACAGTTTTTTTGTTGTCTAACTTCGTGGCAAACGACCCATAGAGGGCGTTGGAGAGTA

GCTTGGCAATGCTGCGCATGGTTTGGTTCTTGCTTTTATCTGCCAGCTCTTTGGCCTGAA

TGTTAAGTTGCACATATTCTCTAGCAAGACATTTCCACTCTGGGAATATGGTGGCTCTCT

CATCGGGGACAAGGCGGACGGTCCAACCTCTGTTATGTAAAGTGATGACATCAATGGTAG

TGGCAATTTCTCCTCGCAATGGCTCATTGGTCCAACAGAGCCGCCCGCCTTTGCGTGAGC

AGAATGGGGGAAGCACATCCAAGTACTCATCCGCGGGGGGGTCTGCATCGATGGTCAAGA

TGCCCGGGAGCAGC-TCTGGGTCAAAATAGCTGATGGTTTTCTGG---CTTTGCATCTTT

ACTTCG---TAACACCTGACAGCCAGGGCCCGCTCGTAAGGATTAAGAGGCGGGCCGGCG

GGAAAAGGATGGGTGAGGGCGCTAGCATACATGCCACATATGTCATACACGTACACAGGT

TGCTTTAAAATGCCTATGTAAGTGGGGTAACACCTTCCCCCTCTGATGCTGGCGCGAACA

TACTCATACATCTCATTAGAGGGTGCCAGCACA------GTGGTGCCCATGTGGGGCTGG

TCGGGCTTTTCTGCTCTGT-----------------------------------------

----------------------------AGAGAATTTGTTTAAAAATAGCATGGGAGTTG

GAACTTATAGTGGGACGCTGGAAGACATTGAAGGACGCCGCAGGTAAGTTTACGGACTGC

ACCACGAAGGTCTGGTAGGCCTCGGTAAGTTTCTGTACCAGCTGGGTGGTCACTTCCACA

TCCAGTGCGCAGTAGTCAAGGGTGCTCTGAATGATGTCGTAAGCG---------------

---------------------CCCTTTTTGCTCTCTATCCACAGCTGTTTGTTCTCTGCG

TACTCTGCCTGATCTTTCCAGTACTTGACGTCGGGAAAGCCATC---AGAGTCTTGC---

TGGTAAGTGCCTAGCATGTAAAACTCATTAACGGCCTGGTAGGGACAGCACCCCTTGGCC

ACTGGAAGGCTGTAAGCCTTGGCAGCGTTTCTCAAGCTGGT---GTGAGTCAAAGTGAAA

GTGTCACGCACCATAAACTTTAT-------------G-TATTGGCTTTTTAAAT--CTGA

GCTCAGAAGCTGGCCCT--GTTCCCACTCGGTGAAGTCTTTT------CTTGGAAAGTAA

GCT---GGGTTTGGCAGGGAGTAAGTAACATCATTAAAGAGCAACCTCCCAGCTCGCGGC

ATAAAGTTTCTGGTAATTT---CAAAGACTGGAGGGATGTCCGCGCGTGTAC-------T

TACAACTTGGGCGGCCAAGAGTATTTCATCAAACCCTGTGATGTTGTGGCCTACCACATA

AACCTCAATGA--ACTTGGGAGTTCCCTTTAGCT--------TTGTTTTTT---TAATTT

CAAGGGCGGGGATGTCTTCTGGGCTCGC-CAGTCC---GAGGTTGGCGGCTTTTTCTACC

AGTTCAGGGTTTTCACAGAGGACGTGAGACCAAAGAGAGGCAGCG---ACCTTTTGCTGG

AGCAGGTCTCTAAACTGCTTGAACTTTATGCCCACTGCTTTTTTCTCTGGGGTGA---GG

CAGTAGAAAATGTCCT---CTTTGTTGTTCCAAGTGTTCCACTGAAGTTGTACTGCCAGT

TTTTGGGCAGTGTTTACCAAAGAAGGTGTTCCCACCAGCTGAAAGACTAGCATGAATGGG

ACCAGCTGTTTCCCAAACTTCCCGTGCCAAGTATAAGTTTCCACATCATACACTATAAAA

AGCCTCTCTGTTTCTGGTGGAGAGCCGATAGGAGAGAAAGATATCTTTTCCCACCAGTCT

GAAGACTGGGCGGACACATGGTGGAAGTAAAAATCCCTTCTTCTGAGAGTGCAGGTATGG

GCAACCTTGAAAAATGATCCGCAAAACTCACATTTTTGTATGGGAGATATTTCCTTTATT

-AAGAACACCTTTCCCTGTT-----TAACTAGAAACTTGAGGGGAAAA------------

------------------------CTAAGTAGTGGGCTTGAGGCTTGGAACTGCACTC--

------------------T-------CTTAACGGTTCCGCGCTTGTAGTAA---CAAATG

AGACTGAATGGGGCGGCCGCCTGCTCTATGAGATCGGGGAG------GTTGTTGACGGTC

ACGGGG---GCGCCGGCAAGGCAACCGGGCACGTCGTG---TAAGTTGGCGTGGCAGAGG

CTGGAGAGAGCTTGCTCAAAGTTAGAGTGGTACTTTATCTCTACAGATTGGCCATCATCT

A---ACACAGCGGAGATGCTTAATGTGGCTCGCTGGGCCACCACTGTGCCTT--------

--------------------------------------------------------TGTA

TTGCCT--------------CTTGCTTGGCCTCA---CGGAGGCAGTTGGACGT---GAT

CGTGGTGTGCTGGCAGAGGGCGGTGGTGGGACCTGAGCTGGGTGGCCAGGGCTACTACCC

TCCTGTTGATTGTCTGAATTTGCTTGTTTTGAGTAAACACTACCGGTCCCGTTACCTTGA

ACCTAAAAGAGAGTTCCATAGAATCAATCTCTGCATCATTAA-CAGCCACCTGTCTAAGA

ATCTCCCCCACATCTCCGCTATTGTCATGGTAGGCGATGTCGGCCATGAACTGCTCCATC

TCTTCCTC-----CTCTAACTCT-CCCAGCCCCGCTCTCTCCACCGTGGCAGCCAGGTCT

CCTGATACGCGCTCCATAACTTGACTAAA------GGCATTTTCTCCCATCTCATTCCAA

ATTCTGCTGTACACTACCTGTCCCTCGCTGTCTCTGGCTCGCATGACCACCTGAGCTAGC

TCTAGGTCTACCCATCGGGCACTAGGGGGATACAGCCTCAGATTGTGGTGTAAGTAATTT

AGGGTTGTGGCAATATGCTCGGCTACAAAGAAATACATGACCCAGCGCCGGATGGTAAGT

TCATTTATGTTGCCCAACATCTCTAGCCTCTGGATGACCTCATAGAAGTCTACTGCAAAA

TTAAAAAATTGCTCATTTCTGGCAGAGACTGTGAGTTCTTCTTGCAGAAGGCGGATCACT

TCTACTATAGTGTCCCGGACCTCTTCCTCAAAAGAACG---CGCGGGCGGAGA-----GG

GGGCCGGG---AGCTCCTC-CATTTCTACCTCTGGCTCGGACAGCTCTTCA---------

---------GCAGCCG--GCGGAGGAACT----CTGCGTCTGCGGCGCCTGACGGGAAGC

CTGTCTACAAACTGCTCTATCATCTCTCCTCTTCTTCTTCTCATCTC---CTCTGTCACG

GCCCTGCCATTT---TCTCTGGGTCTCAATTCAA----ACGCCCCTCCAGACAGC-----

------------GGGGTGCAGGGGGCGGGG-------CGCGGTAGGCTCAACGCACTAAT

TATGCTTT-TTGTTACAGTTTGACTGGGCAAAGACCTG------ATCGTGTGA--AACT-

---CGGACAGTTCCGGGTCAGCGAATTTCT--CCACGAAGGCGTCCAGCCAAAGACAATC

ACAAGGTAAG---CTGAGCATGGTGTGGGG------------------------------

---------------------------------------GTTTTTTGGGGTAACTAAAAA

GTTAAAAAAAGCAGTTTTTAACTTTCTGATAGTGCTCAAAATGGTGAGATCTTTTGCTCC

GGCTCTCTGAACTCTAACCCGAT-CGGCAAGTCCCCAGGC-CTGGCTTTG-ACACTGGGA

CAGGTTTTTG--TAATGGTCTTGCAGCAACCCCTCTACTGGCACTTGC------------

------------------------------------------------------------

------TCTCCCGCCATGCTAGTTCTTCCAAACCCCCGGAGGGGGCGGATCATAGCTAGG

TCTGCCACCACTCTTTCTGCCAGCACAGCTTGCTGAACCTGGGCAAGGGTTTCCTGAAAG

TT------------TTCAAAATCTATGAATCTGTGGTACGCCCCCGT-GTTGATAGTGTA

GGCACAATTTGCCATCACGGTCCAATTGAGCTT--TTGCTGAAGAGGCAAACGGATCTCT

GTG----TACTTTAGCCTGCTGTAGGATCGTGTTTCAAAG-ACATAATCATTACATGTTC

TGACTAGGTACTGATA-GCCAACCAGGAAGTGGGGTGGGGGATAAAGATACAAGGGCCAG

CCCTGAGTGGCAGGCTCTCTGGGGCTAAGGTTCATAAGCATGAGGCGGTGGTACTGG-TA

GATGTACTTAGACATCCAAGCTAAACC-CACAGCGGTAGTTGAGGCTCTCGTGTAGTCGC

GGACGCGATTGAAAATGTTTCTGAGAGGTCTAAATACTTCAATGGTGTACTGGGTTTGTC

CTGTGAGGCGGGCGCAGTCCAGCGCGTTCTGC----------------------------

------------------------------------------------------------

------------------------------------------------------------

------------------------------------------------------------

------------------------------------------------------------

------------------------------------------------------------

------------------------------------------------------------

----------------------AAAAACAA--------ACAA-GGAG-------------

--------------------------------------------TTTTACTCCA------

---------TTGCAG-ATGCATCCGGTACTGCGCCAGATGAAGCCCC-------------

-----------------------------TCCCAGCTT----------------------

-----------------------CTGCCAGCAGGCTAGAA------------GC------

---------------------------AGAAAGCGAGGGAATGG-CCCGAATACAGGGCG

GGGCCGCGCCAGAGA--CTCACCCTCGGGTGCTAATGAAACAGGAGGCATCAGAGGCCTA

CATCCCACCTAGTAATGTGTTCAGAGA---------CAACGAGGGAGAAGAGGCAGAGGG

GATGCGCCACTTAAAGTTTGAAGCAGGCAGAACCTTGAGAGGCAGCCACGGGGCCCGAA-

GGGTGTTGGGGGAACGAGACTT--TGAGAAGGATCCCAGGAATGGCA------TCAGCCC

AGCAGAGGCTCACCTCAAGTCCGCTGACCTAGTTACAGCTTATGAACAAACAGTGAATGA

GGAAGTGAACTTTCAGACCTCGTTTAACAACAATGTGCGCACGCTAATAGCCAGAGAGGA

AGTGGTGGTAGGGCTGATGCATCTTTGGGACTTTGTCCAGGCCTATTTAGAGAACCCAAT

T-------AGCAAGGCTCTGACAGCACAGCTTTTTCTCATCGTGCAACACTGCAG-AGAT

GAGGGGGT---ATTACGGGAAA--GCTTGCTTAATATTGCAGAGCCG----GAAAGCAGG

TGGCTGGTGGACCTGTTGAACCTGCTGCAGACAATAGTTGTGCAGGAGCGGGGCCTCTCG

GTGGGAGAGAAAGTGGCTGCCATCAATTACTCAGTAATTACTCTAAGCAAACATTATGCT

AGGAAGATTTTCAACTCTGTGTTTGTACCTATTGATAAGGAAGCCAAAATCAACACCTTC

TACATGCGGGCTGTGGTTAAGTTGCTGG------TGCTGAGCGATGACCTGGGCATGTAC

AGAAATGAGAG--GATAGAGAGAGCCGTGTCTGGGGCCAGGCAG-----CGGGAATACAA

CGACAGAGAATTAATGTCCAAGCTGAGGCAGGC----CCTAGCAGACAATGGCATCAGAG

AGA------TAGACTATGAACATCTAGAGGAAGATGATGAGGAGGATGAAGGAAG-----

--GTTCAGGCCGAGGGAGGTTTGGGGCGCCGGGACGGGGTGCGGAGCTTCTGCAGCCAGG

--TA------CCCGGAAGCTGA---TTTAGAAGAGGAG---GATGGG----------CCT

GATAGTT---------------------------CCATCTTATTCC--AGCAGCATGAGC

GCGGCACTCAATCCAGTGAAAATGGCGGCTATGCAGAGCCAGCCCACGGCCGAC------

---GACGGCTGGGCCGCTTCTATTAACCGTATAATGGCCTTAACTGCTGGAGACCGGCAC

AAGTTTTCTTCCCAGCCATTTGCTAAC-AGGCTGGATGCCATTTTGGAGGCTGTGGTGCC

GTCTCGCAAGGACCCCACACATGAAAAAGTTTTGAGCATTGTAAACGCCCTCATAGAAAA

TGGGGCAATTAGAAGAGACGAGGGGGCGGGGGTTTTTGACGCCTTGCTTGCTAGAGTATC

TAAGTACAACAGCATGAATGCCCAGAACAACCTGGAAAGGCTGGCTGGAGACGTGA----

--GGGAAGCTGTGGCCCAAAAGGTTAGAATCGCA---GAAGGAAACCTAGGGTCAATGGC

AGCTCTCAACAGCTTTCTCGCCCGCCTGCCAGCCAATGTTGAAAGGGGGCAAGAAAACTA

CACTGGCTTTCTATCTGCCCTCAAGCTGCTGGTCAGCGAGGTTCCTAA---TACAGAGGT

GTACCAGTCAGGCCCCCATTACTTTCTGCAGAGCAGCAG-AA--ACGGGACACAGACTGT

TAATCTCACAACTGCTTTTGAGAACTTGAAGCCTCTGTGGGGGGTGAAGGCCCCCACCAT

GGAAA-GGTTGAGCATCTCAGCCCTG-----CTCACTCCCAACACCAGATTGCTTCTTTT

GCTGGTG-TCTCCTTTCA-CAGACAGTGTCAGCATTAGCAGAGACTCTTACATAGGCTAC

CTGCTCACTCTATACAGAGAGGCTTTGGGGCAGGCACACCTG---GATGAGCGCACTTTA

CAAGAGGTCACCCAGGTCAGCCGCGCTTTTGGCAGTGACAGCATTG---ACAATTTGCAA

GCCACTCTGAACTTTCTTCTGACTAACAGGCAGAAAAAACTGCCCAGAGACTATTCTCTC

ACTCCAGAGGAGGAGAGGATTCTCAGGTTTGTGCAGCAGGCAGT-CAGTCTGAGAATGAT

GCAGGAGAATCTTAGCG--CCACTCAGGCCTTGGATGTAACTGCAGCGAACATGGAACCC

AGCTTTTATGCCACCAACCGGGATTTTATAAACAAATTGATGGACTATTTTCACCGGGCG

GCCACCATGGCTCCAGAATACTTTATGAGCGCGGTAATGAATCCGCGGTGGCTGCCTCCT

GAGGGGTTCTTCACTGGAGTGTTTGACTTCCCAGATAGGGA---AAAC---TATGCTTGG

GATGGGCTTGAGAGCTCTTTA------GATCT---TACATATAGGGACGCGGCCATGAGG

GCAGTAGAGGA---------AAAATTCATGGATGATGACCAGAGAACCG-----------

AGTCC---------CGCCTGAGCAGCCG------GGTGAGCACCC--------CCGTGGT

GTC------CAGGCGGGGGTCAGTGGCTTCCT-------C---TGCTGGGGACTTCCTGT

CTCTGG--TAAAAAATAACGCC-AGTAACAATAACAAGAAT---------------GCCA

TGCGGGAACTGGACACTCTAGCAGACAAATT-GGCTCGGTGGAAAACTTACAAAAGAGAA

GCAGAGGAAGCAAGGGC------------GGCAGTCCC---TTCAGTAGTCCGTCCGAAG

GAGAAAACTCCCTACAGGTCTCGCTCTCCCTTCCCCAGTGATGACAGTGATGATGGCATG

AGCAAGCCAGACCCTTACCTCAAGTTTGAAGGGCAGGG------------AAACCCGTTC

GCTCACCTGTGCCCCAAGAGAGGGCGGCGCATTTTCA-------------AACAATAAAG

CAATT----------ACTCACCAGAGCCATG----------ACGCAAGCGTTTTT-----

--TTATAGCT--------------CGATGGAG----------------------------

T--ACCCGTCGTCTCCTCCCCCGTCTTACGAGGCCGTGAT--------------------

----------GGCT---CAAGTGCCCTCGATCCTG-GCACCAGTGGTCCCTCCACGCTAT

AAGAGC-GCTACAGAAGGAAGAAACAGTATTCGTTATTCCCAGCTCCCTCCTCTTTTTGA

CACCACCCGCCTGTACCTCATTGATAATAAGTCAGCAGACATTCAGGCCTTAAACTACCA

AAATGACCACAGTAATTTCCTCACCACGGTGGTGCAGAACTCCAACTACTCTCCTATGGA

GGCCAGCACCCAGTCAATCCAGCTGGATGAACGGTCTAGGTGGGGAGGGGAATTTAAAAG

CAT--TTTGCACACTAACATTCCCAATGCCACAGAGTTCATGTTCAGCAACAGTTTCAGG

GCTCTGCTGCCGGCGGCCGCCGATGCTAGCGGTAA-------------------------

-----------AATTACTAGCTACGAGTGGTACACACTCTCTATTCCAGAGGGAAATTAC

TCAGAGGTCATGCTGTTAGATTTAATGAATAACGCGGTTGTTGAAAACTACCTGGCCCAC

GGTAGGCAGAAAAATGTAAAGGAGGAGGATATAGGCCTCAAGTTTGACACCAGAAACTTC

AGGCTAGGCTTTGACCCCGAAACTCAGCTTGTGATGCCCGGGTTCTACACTAATGAGGCT

TTTCACCCAGACATTGTCCTCAGCCCCGGCTGTGCTGTGGACTTCACTAAAAGCAGGCTG

AACAACTTTCTGGGCATTAGAAAGAAGTACCCCTACGAGGAAGGGTTTATCATAACCTGG

GATGACCTCCAGGGAGGGAACATCCCCGCGCTGCTCGACCTGCACACCTAT---------

---GACCCCAGTCAACC------------------CAACCAGAATGTCAC----------

------------------GCCCGTCAGA--------------------CAAGA-------

------------------------------------------------------------

------------------------------------------------------------

-----------------TTCCAAGCAAAGATCCTATCACGTGGGGGAA---GACCCTACA

GCG---GGGGCAACTTTCACCTCGTACCGCAGCTGGTACCTAGCCTATAACTATGGAGAG

CC---AAGC---GGCATCCGAAGCAAAACTCTTCTGGTGACCCCTGACATCACCTGTGGA

GTGGAGCAAATCTACTGGAGCTTGCCAGATATGGCAGAAAACCCTGTCACTTTCACCTCT

GGCC---ACAGTCCCAGCGCCTACCCCGTGGTGGGAACCGAGCTCTTGCCTCTGCTTCCA

AGGAGCTTCTACAACGGGTCCTCTGTGTACACCCAGCTGCTGCAAGAGAGTGC-CAGCCA

--AACCCAAGTGTTTAACAGATTTCCCGAGAATGCCATTTTGCAAAGACCGCC-AGCCCC

CACCATCATTAGCATCAGCGAAAACGTGCCCGCGCTGACCAACCACGGAACTATTGCTCT

CAAAAATAATATATCTGGGGTGCAGAGAGTGACGCTGACGGATGCAAGGAGGCGGGTCTG

CCCCTACGTGTACAAGAGTTTGGGGGTCGTGGTGCCCCGCGTGCTGTCCAGCAAAACCTT

TTAGTGAAT-----------------AACTTTTTCCTTTTTATTAGC-------------

--------CATGGCCATCCTTATCTCACCGTCTAACA-ACACCGGCTGGGGCCT-AGGCA

C-----TCACAAGC------TCTTTGGAGGAGCCAAGCAAAAGTCAGAT--CAGCACCCC

GTTTTTGTGCAAGCCCACTACCGCGCCCAGTGGGGAA---GCAGGGG-------------

--------C--CGCCGCGC---CAGGCGA---AAGGGCCGCAGAGTTA--GGGTGGT---

---CATGGACCCCCAAACAGAAGCGGAA-------ATGGTTGCAACTATTGATGAGGTGG

CCCGCAATGGCCCCCCTGCCGCGCG-GGCGGTCTTGGAGGC--GGCGCGGCGCGTGGGCG

CCTATAA-----------TCTGAGACGCGCGC----CTAGGCTCACTCCA----------

-GCTGGGCGTG-----------------------CTCTCAT-GCGAGCCAGGGCGCGGCG

GCTGGCCCGTACCACC--------------------------------------------

--------------------------GGCC------------------------------

---GCAGGCGAAAAAGAAACTAAAAAATACAATAAACTTGCT------------------

-----------------------------------------------GCTGCCCA-----

-----------------GTTTTACCACA------------------------------CT

CGAGTGATGGCCTATTTT-------------------T------TTACTG----------

-------------------------AAGCCTCGTCGCCATGGC--TGCCCTC--------

-----------------------AGCCGCGCCATCAAGCAAG-AGCTGC--TGGAGGACC

TCAAACCAGAGATCTATGTAGCACCGAGA-----------------CGGAGAGCGAGAGT

TAAAACAGAG-----------------GAGAAAGTTG--------ATGTGAAAACCCTAG

TAAAAGCTAGGAGCAAAAAGCGCAGGGCTGCCAAACAG----------GAGCTG--GAAG

AAGATGTGGAATTTGTTAGAAAGTTTGCCCC------ACGGCGCCCTTACCAGTGGAAAG

GCAGAAAGGTGCAAGCTGTGGTCCGACCGGGAGTACCAGTAGTCTTCACACCCGGCCAG-

AGAG--TAGGCAAAGCTATAAAGAGAGATTATGACGAGGTGCACGCAGATGAGGATATTC

TAGAGCAAGCCGGGGTAATGATTAATGAGTTTGCCTACGGAAAAAGGGCCAAGCT---TC

TAACGGAGAGAAATCCCACCCCTTCCCAGGTTCCC---ATTACTCCCCAGGAACCAGTGG

TTAG---------------GCCAGGAGAAGCTAAGTTGCTTCCTACAGTACAAGTGCTGG

TGCCCAGGGACGTTAAAAAGGAGGCAGTGCTCCCGGTGGGTA--AATCAGAAG-CGGGGG

ATGTAAAAATAGAAAACAAGGGCCTTGAACAGG--TGGCTCCTGGGCTCGCTGTGCAGAC

GGTAGACATCAAAGTTCCCATTAAGAG-AAAAATGGC--GGA------CGATGGGGTAGT

AGTT----AAAAAACTAAAAGAAGAGATAGA---GGAAGAGTTCAAACCGACAATAAAGA

TGGAGTACTCCGAGGGGCCCGTGG-------CAGAGCTGGTGCGCCCAAAGGCCGTGGCA

CGCAAGCGCAGGCAGCCAGC--------------TCCCCCGGTAGAAGTAATGGAAGTA-

CAAAGCATGGCGGCGCCTCCC------------------GCCCCCGCAGCAGTGGTGCAA

CCTGCCCCCG-------------------------------CTATGC-------------

-------CAATGGCAGTTGAAGTAACCACGCCTGTCAGAAGAGTGAGCCGGTGGG----G

TCCGGCCAATGCTATATTTCC----CGAATACCGCTACCACCCGAGCA--TTACAGCGGC

TAAGATTCGGGGACCTGCTCCCAGAGGGCGCGTGAGCCGATGGGGGCCCGCCAACAGTAT

CATCCCTGAAGTAAGGCTGCACCCCAGTATGGTGGGAGCC--------GTCACCAGGGGA

GCCCCT------AGGC------------------GCCGGCGGAGGACTAGGCAGACTCGC

AGAC-------------GGGGGCGGCTGAC-CCGTCGC-GGAGCCCTTATTCCCACTGAC

GTGCGCTACCATCCATCTATCACCCTCCTTTCACGGCGAGT-CTAA---------TTTG-

------TCC---TTTTTGGTTTG----------------AGGATGCGGGACAGAAATGTA

ACCCTTCGCGTCAGAGTTCCCGTGCGCACACCTTTTTCTGGGAGGGGGCGTCGCCGCACC

---CGTACTGTGA-AGCGCATGCGCTGTGGACG----AATGAAAGGAGGATTTTTACCTG

CTCTAATTCCCCTGATTGCTGCGGCTATTGGCGCTGTTCCAGGCATAGCGTCAGTAGCTC

TGCAAGCTGCCCGCCACTAGTCCACTTAAC------------------------------

----------------------CTGATTTAC------TGCCATTT---------------

----------TTCTTGC---GCCAGAAGAAGA----------------------------

-GATGGACGCTGTCAATTTTTCATCCCTGGCTCCACGATACGGGTCCCACCCCATGATGA

ATAGCTGGTCTGGCATCGGCACCAGTGGCATGAACGGAGGGGCTTTTAACTGGGGGGGTA

TCTGGAGCGGCAT---------------AAAAAACTTTGGCAGCAATGTAAAGAGCTGGG

GTAGCAAGGCCTGGAACAGCCAAACAGGCAAACTGCTGAGACAAAAACTAAATGACACCA

AAGTAAGGGAGAAGCTGGTGGAGGGCATCTCTACCGGGGTGCACGGGGCCCTGGATATAG

CAAACCAGGAAATTGCCCGCCAAATTGAAAAGCGCTTAGAGC---GCCGTGAGCCACTGG

AA---GAAGTAGAGG----------------TGGAAGAAGAAATTGAGGAAGTGAAACCC

AAACTAGAA------------GCCCCCTTGGTTGTGGAGCTGCCCAAAAAGCGTCCTCGG

GATGAGGAC---TTGCTCATTACAGCTGAAGAACCTCCCTCCTATGAGGAAGCCGTCAGT

ATGGCTCCAGCCCCACGC-CCCATGACCCGGCCTCACCCTTCCATGGCCCGCCCAGTGCT

GGTAGAAAAACCCGCCCGCCCAGTGGTGGTAGACAAACCCACAACTCTGGAGCTTAAACC

CAGCGACCTGCCTCCTCCTTACAG---CCCGGCGCCCGCCCCTGTAGTAGT------AAA

AGCCCCGCCTAAAGCGCCCGCTGTAGTGGTT------CCCTCAGTGCCGGCCGTGCCAGC

CGCCCCAGTA---------GTGGTGGCCCCCAGCCGCTCTCGCGGGTGGCAGGGAACCCT

GGCCAACATTGTAGGAGTGGGATTAAGGGGAGTGAAACGCCGCAGATGCTACTAATGTTT

C--ATTAAAT-GGTGAAAAGTGA-CTTCAG---------TTGCCGACCCTGTCTTTTTT-

------------------TGACCGCGCCAGCTGA-------------------GAAGATG

GCGACCCCGTCGATGCTGCCACAATGGTCTTACATGCACATTGCGGGCCAGGACGCCGCG

GAATACTTGTCTCCCGCCCTGGTTCAGTTTGCCCAGGCCAC-CAGTTCTTACTTTAAGTT

GGACAACAAATTCAGAAACCCCACTGTGGCCCCCACCCATGACGTGACCACAGAAAGGTC

ACAGCGGCTGCAGCTGCGCTACGTGCCAGTCATGCAGGAGGACGGGCAGTACACTTACAA

AACCCGCTTCCAGCTGGCCGTGGGGGACAACCGGGTGCTAGATATGGCCAGCACCTACTT

TGACATTAGGGGTACCCTGGACCGAGGTCCCTCTTTCAAGCCCTACAGCGGCACGGCCTA

TAATGCCTTGGCCCCCAAAGCTGGAGCCAACAACTGCC-------------------TCT

TTAACGGACAGGGAG---------------------------------------------

-----CCAACA------------------------TTAACACTCTAGCTCAGGTGTCTGT

ACCCGGAAACATAACTGCCCCAGATGG---------------------------------

-----G--------TTAGCGGGTGTAGAAGACTCTACTTTCCAACCCCAACCTCAGCAAG

GTCCAGAGAGCTGGACGACTGGAGAG---CTGGGAGCTGTAGGCGATGCTGGAGGAAGAG

CTCTAAAAGTCACAACTCC---CAGACAACCCTGCTATGGTTCCTATGCTCCCCCCACTA

ATGCAAACGGAGGACAGGCTA------------------AGGGTGC--------------

-AGTAA----------CTCCTGTG-----TATTAT---------------AAGAAAACAA

CCACAAATGGAGCAACTGAGGTAGATGCAGTGCTTTACACTGAGGACGTCACACTGGAGG

CGCCAGACACACACTTAGTCTATAAGGTGGAACAAGGTGCCGCGAC------GGGAATTC

CAGGGCTTGGTCAACAAGCAGCTCCTAACAGGCCCAACTATATAGGCTTTAGAGATAACT

TCGTAGGTCTCATGTACTACAACAGCAACGGCAACCTGGGAGTACTAGCTGGACAGTCCT

CTCAGCTCAATGCCGTGGTAGACCTGCAAGACAGAAACACTGAGCTATCCTATCAGCTGC

TGCTAGATGCTCTCACAGATAGAACCCGTTACTTTTCAATGTGGAACCAAGCTGTAGACA

GTTATGACCATGATGTTAGAATTATTGACAACCATGGAGTGGAAGATGACATGCCCAATT

ACTGCTTTCCTCTCAGTGGAATGGGGCCTTTGGTAGATGCCACAGGTATGAAATTAAATC

AAC------AAAACAAT------------GGCTTTGAAGCAAATGGAACAAATCAGGG-T

CCCATACAGAAGCTAGGGTTTGGAAACGTAGAAGCCATGGAGATCAACTTAAACGCTAAC

CTCTTCAAAGGCTTTCTTTACTCTAACGTGGCGCTGTATTTGCCAGACACATACAAATAC

-------ACTCCTGAAAATGTCACAGTG--CCGGCTAACACTAA---CACCT---ATGCT

TACATGAACGCCCGCCTTCCCGCTGCTAATTTAATTGATACTTTCGTAAATATTGGAGCG

AGGTGGTCACCTGATGTAA-TGGACACTGTTAACCCTTTCAACCACCACAGAAATGCAGG

ACTGCGGTACCGCTCCCAGCTGCTGGGCAATGGTCGCTATTGCTCGTTTCATATTCAGGT

GCCTCAAAAGTTTTTTGCAATCAAAAACCTCCTACTCCTTCCGGGGACGTATACCTACGA

GTGGTCATTCAGAAAGGATGTAAACATGATTCTGCAGAGCAGCTTGGGAAATGATCTGAG

AGTAGATGGGGCGACTATCAACATTCAAAGCATCAATCTCTATGCAAGCTTCTTCCCAAT

GGCTCACAACACAGCCTCTACTTTGGAAGCCATGCTGCGGAATGATGTAAATGACCAAAC

ATTTGCAGATTATCTCTCAGCCGCTAACATGCTGTACCCTATCCCGGCCAACACAACCAA

CCTGCCTATCTCAATTCCGGCCAGAAACTGGGCAGGTTTCAGAGGATGGAGTTTTACTAG

AATCAAGCAGAGAGAAACTCCTGCGCTGGGGTCTCCCTATGATCCTTATTTTACCTACTC

TGGAACCATTCCGTACTTAGATGCTACTTTTTACCTTAGTCATACTTTCAGGAGAGTATC

TATAATGTTTGACTCCTCAGTATCCTGGCCAGGAAATGACCGGCTTCTAACGCCCAATGA

GTTCGAGATCAAAAGATATGTA------GATGGGGAGGGGTACAATGTGGCACAGTCTAA

CATGACTAAAGACTGGTTCTTGGTGCAGATGCTTTCTCATTACAACATTGGATATCAGGG

TTACCACCTGCCAGAATCATACAAGGACAGAATGTATTCTTTCTTGAGAAATTTTGAGCC

CATGTGTCGCCAGCTTGTGGATACACA----AAACT---ATGCTGAGTACAAG--GCAGT

TACAGTTGCAAACCAG-----------------------CAC-------AACAACTCTGG

CTACGCCAGTGCCCTGTC---CGGCTCCAACGCCAGAGAG----GGGCACCCCTACCCTG

CTAACTGGCCCTATCCCCTCATTGGCGCCAATGCTGTA---------CCCTCAATCACCC

AGAAA--AAGTTTTTGTGCGATAGGACCCTGTGGAGAATTCCATTTTCTTCCAACTTTAT

GTCCATGGGCACACTGACCGACCTCGGTCAGAACCTTCTGTACTCCAACTCTGCTCACG-

CGTTGGACATGACCTTTGAGGTTGACGAGATGAATGAGCCCACTCTGTTGTACGTTTTGT

TTGAAGTGTTCGACGTGGCACGGGTCCACCAGCCTCACCGGGGGGTCATTGAGGTAGTCT

ACCTCAGAACACCCTTCTCTGCCGGCAACGCCACTACTTAAAAAGA--------------

------------------------------------------------------------

-------------------------------------------ACATGGCGCAAGGAGG-

-------------------------------------------GTCCTCTGAAGAAGAGC

TTCGGGCTATTATTCAGAACTTAGGCGTGGGGCCCTTTTTCTTGGGAACCTTTGATAAGA

GGTTTCCTGGTTTCATCT-CTTCTCAGAGAATGGTCTGTGCCATTGTCAACACAGCAGGA

AGAGAAACTGGAGGGGTGCATTGGCTGGCCATGGCCTGGAACCCCAGAGCTAAAAAATTT

TACATGTTTGATCCTTTTGGTTTTTCTGATCAAAAGCTTAAGCAGATCTACAGCTTTGAA

TATGAGGGCCTACTGAGGCGAAGCGCTATTGCCTCAACTTCAGACAGGTGTGTAACTCTG

GTAACCAGCAATGAAAGCATTCAGGGTCCGAACAGTGCAGCCTGTGGTCTGTTCTGTTGC

TTGTTTCTGCATGCTTTTGTAAATTGGCCAGATGACCCCTTTGACAACAATCCCACAATG

GGACCTATTCAAGGAGTGCCCAACTACAAGCTAATGAGCCCGGC-TGTGCAAA-CTACTC

TCTGGCTAAACCAGGAAAAACTGTACCAGTTTTTGCAGACTCATTCTTCCTACTTTAGGG

CCCATGCGCGAGAAATCAAGGCCAGGACTGCCTTTGACAAATTAACT-------------

----CAATAAA-----CCG-------TTTATTCGAAAAT--AACACAAGCAT--ACAGTG

TGGTTCTTT------------------------------------AAAACAGAGAAGCAT

CGTCATCGTCT-----------TGCCCCTGA----------------GGCAAAATAGTAG

TTTGATACTTGTA-----CTCATTCGACCACTTGAACTCTGGGAACTTCAGCGGGGGT--

----GGAGTGCCAATGCAGGTAG---CCCAG-----ATTTTCTTAGCTATCTGCACACAG

GACACCAGGTCTACAGTGGAGATTTTAAAATCACAATTTTTCTGAGGAGCCGCCTTGC--

-----TATTGCGGTAAAC------AGGG--TTGCAGCACTGGAACACCAGCATGGCCGGG

TTGTTTAAAGTGGC---CAGCATTTTAGGGTCATGCACCTGACTTTTGTCTATGTCAGCT

GCGG-ACGGC-----ATAGAAAAGGGA---GTTATTTTGC----------AAGTTT---G

CCGCCCAACC---------------------ATGGGAAGGCTGTTG---TTCCAGTTG--

CATTCA-----CATTTCAGTGGAAT-TAGCAAGTGGGTCTCA------GCAGTGGTCATC

TGGGGATAGCAAGCCTTTTGGAAA-------GCCATGATCTGCTGAAAGGCCATCAGAGC

CTTAGGCCC----GTCACTGTAGGCCATGCCACAAGAGCTGCCACT-------AAAGTTT

CCCCCGGACATATT---------TACATCCATGAAGCAGCAAAAAGCATCCTCATTT---

-TTCATCTGCA--CTACATTGCGC--------CCCCAGCGGTTGGTGACAAT----TTTA

GCCTTTTCAGGATTTTCTT----TCAGCACCCTCTGCGCG-----TTCTCGCTGT-----

----TCACATCCATTTCTATAAGCTGCTCTTTCTGTAT--------------CATGGGAG

AGCCGTGAAGGCACAGCAGGTGGTCTT-------------------G--GCAACCATGCT

----------GCCACA----CCACGCAC-----CCGCTAGGGTTA---A-----------

-TGCCGGGAGCGAGCTCTACCGCTTTCAGCACAAAATCGAGCAAGAGCCTAGCAGTAACA

TGC----TGAAAG----------------------GTTTTTGGGGTGGTAAAGTTGTAGG

TTACCA---------CGGTTTTGGTTCCCACCAAGAAGCCC----TGCACCGCTTTCTTA

AAGCATTCCAGGGTGCCAGAATC------AGGCAGCAAGGT-------------------

-T--------AAATCCTTATGGTCAACCT----GGTAGGCA--GTAAGGATTTTCAGGGC

ATACTCCAT---GCCCTGCTGCCACTGGTGCTCATTGGCCTTGGTG--------------

------------------GGTTGAATGGCCCGAGTGGCCTTAATGATGGGCTC-------

-------------------------------------AGGCGGG---------GGAAACT

TATTAAGCGGAGCAGGACC-------------ATCCTCCTTTATAAACTGAATCTCCCCA

TTGGCATCCCGTTCCACC-CTCTGGTTGCAAAAAGTCCTGATGTAGAACTCTCCAGGGGT

GCCTTG-------A---GTCTTCAGAGCCTCGTTCACTTT-----------CTT-GGTAG

CATCCAGCATGGCGT---------------------------------------------

----------------------GGTAGCCTCTC------TTTGGCTCTTCC---------

CGGGG-CTCTTGTTTAACCTTCACTTTGCCATCGA----TGGTTTTGCGCTGGCGC----

-----------------------------------TTCCTCGGCG---------------

--------------GCGCGGGAATCTCATCTT---------C---GCTGG--AGCTT---

GAGATTA------------------CAACAACGTTGTTCTGGCT----------------

-----CATCTTTT---------------T-CCTAGATGTCA----GAAGAGCCTGTCAGT

GGAACTGCC--------------------------------------------------G

AGAACACGCAGGAGGAA----------------------------ACTTTGCTTGC----

---------ACCTCCCA-------------------------------------ACTCTC

CAGTTCTTG---------------------------------------------------

-------AGTCA------------------------------------------------

G--------------TTCCACT------CACCCCTGAGGTT-------------------

-----------------------------------------------GAACTTGA-----

-------------G---CCCGA---GCC--------------------------------

------------------------------------------------------------

--CGAC--------------------------------------------AAATATCTAA

GCGAGGATTTGC------TATGTAAACACTTACAGCGCCAAAGCAAAATTGTTTTGGATA

GCATCCAGGACCGCCTACA-ACTGCCTGCC-AGCATTTCAGAACTGAGCTGTGCTTAT--

GAAAGAAACCTTTTTTCTC-------CCAACATCCCGCCAAAG-----AAACAAGAAAAT

GGAACTTGTGAGCCAAATCCCAAGCTCAACTTCTACCCTACTTTTGTGGTGCCAGAAACT

CTGGCAACTTATCACATTTTTTTTGTTAACCAA-AAAATCCCGATGTCCTGCAAAGCCAA

TAGAGCAAAGGCAGACAAAGCCTTGGCTCTGCACGAGGGAGATTGCTTACCTGATTATGA

GACGATGGACACTGTAAGCAAGGTGTTTGAGGGTCTAGGCGGAGAGGTCGT---GGCCGA

GAACG---CGCTGAA------AAACAGTGAA---------------TCGGTTTTAGTGGA

GCTGCAGCATGACAATCCCCGCCTGGCAGTTATGAAGCGCAACCTGA---CTCTCACACA

CTTTGCCTACCCCGCTGTGCACCTCCCCCCTAAAGTAATCACTACCATGATGGACACGCT

G---------CTCGTGAAAAGAGCGTCTCCCAGCTC---------AGATGTATCGGAGAT

AGA---CCCTGAGGGTGGTGAACAGGTAGTAACAGATGCTGAACTAAGCAAGTGGCTGCA

----GACCTCAGAC-----CCTGAGGTGC------------TAGAGAAACAAAGAAAAAC

TGTCATGGGGGCGGTTCTAGTGTCAGTAGTGCTCGAGTGCATGCAGCGCTTTTTTACTTC

TGCAGACATGATAAAAAAAATAGGAGAATCTCTGCACTACACTTTCAACCACGGCTATGT

GGCTCTGGCTAGCAAAATTTCCAATGTAGAGCTGACCAATGTAGTCACGTATATGGGTAT

TTTGCATGAGAACCGCCTGGGTCAAAA-CGTGCTTCACCATACCATACATGGAGAGGCAC

AGAGAGACTACATCAGAGACACTATCTTTCTTGCCTTAGTCCATGCATGGCAAACCGCCA

TGGGCGTGTGGCAACAGTGCCTTGAGGTGGACAACCTCAAAGAGCTAGTAAAACTTCTTC

AGAGGCAGAAAAAATCTCTGTATACACAAACTTCTCAGCGCTTCATAGCTAAA-GACCTG

GCTGATACAGTA-TTTCC--TCCTAAGCTTCTGGGCGCGCTGCACAAAGGCCTGCCG-GA

CATAGTCAGTCAGAGCATGATGCAGAATTTTAGGTCTTTTATCCTAGAGAGGTCGGGCAT

CCTACCAGCCATGAGCGCGGCAATGCCCACTGACTTCATTCCAATCTTTTTCAAAGAGTG

CCCCCCCACTCTGTGGCCTTACACCTACCTGCTCAGGCTAGCTAACTACTTTATGTACCA

CAATGACCTGTGC------TTCTCGGTTGGTGGAGAGG------GG---CTGATGGAGCA

CTA---CTGCAGGTGCAACCTGTGCACCCCCCACCGCTGTCTGGCCACAAACCCCGCCAT

GCTCAACGAAACCCAACTCATTGGTACCTTTGATATCCGCGGGCCCGGTGGGGAAA--AC

GGAGAGCAGTCTTCCTC-AGGTCTTAAGCTGACGGCAGGAATGTGGGCTTCCGCTTTCCT

GCGAAAATTTGAAAGTGCTGATTACCACGCCCATCAAATTAAATTCTATGAAAACC---A

ATCAAAGCCCCCCGCAGTAGACCCCAGCCCCTGCGTCATCACACAAACAAACATTCTGGC

CCAATTGCATGACATTAAAAAAGCCAGAGAAGAGTTCCTCCTCAAAAAGGGGCAAGGTGT

TTATCTAGACCCTCACACCGGAGAACCGCTAAACGC------------------------

--------------------------------------------------TCCAGATCCT

TCTATAGAGAGCG--------GACTTGAAT------------------CCCGCAAAGATG

GAAGA---GACTCACAGC-----------TTAAGTGTGGAAGAAATCTCCG---------

-------------------------------------AGG---------------AGGAC

CTAGAAAGCCT------------TCCAG---AGCTCACAGTGCCAGAGAGCGAGGAA---

---------------GCACTCCCA------------------------------------

---------------------------GAG---------------------------CAG

CGAGCT---------------------------------------------CCTAGATGG

GATCAGAAAACTAAGACGCCCGG-------TAAG---CCCCCTC----------------

------------GGAACTACAAATCATGGCGGGCTCACAAATATCGCATCCTTAACTGCC

TGGGCGCGAGCGGAGGGAACGTGGCCTTTACCCGAAGATACATGCTATTTAGGGAGGGGG

TCAACCTTCCCAACAA-CATCATTCATTACTATAA--CTCTCGCTACCACAGC-------

--CAAGAAGCACC----------------------------GGCGGCC------TGCCAC

CCCTCCAAAACGCAACCGGGTGGCAAGCCT------------------------------

-CACCACCTCAG-------------AGGACACCCAGCAAACCCGACA-GCTCAGAAACCG

CATCTTCCCCACTCTCTACGCCATCTTTCAACAAAGTCGCGGTTCCCCCACGGCC-----

----TTTAAAATAAAAAACCGCTCTCTGAGGTCTCTGCTCAAGA-GCTGTCTATACCACA

AGTCAGAGGCTCAG-CTGCTGCGCACGGAGGACGACGCCGAGGCTCTCCTCAACAAATAT

TGT-----------CAGGCGGAAGGTCTCAAC----------------------GAAGAG

TGA-----------------------------------------------GCGATGTCCA

AAGAAA-TACCCACCCCTTATATGTGGAGCTACCAACCGCAATCTGGTCACGCGGCGGGC

GCGGCTCAGGACTA---TTCCACCCAGATGAACTGGTTTAGTGCTGGACCATCAATGATT

AATCACGTTTACCAGATAAGAGACTTGCGCAATAAAATTTTAACGGCTCAAGCAAACATT

ACAGAAACTCCCAGAACTATCATGGACCCACCTGTCTGGCCGGCT--GCCATGGTTACGC

AAGAGGTGCCCCCACCCACA-ACGGTCACTTTGCCGCGAAACTACGCCCTAGAAACTGCC

ATGACAAACTCGGGGA--TCCAGTTGGCGGGAGGCCGCCG----------------C---

-CTGTGTCCTCT-GAGCCACACCCAGAGGCA-------------GATAAAAGGGCAAGGA

ATTCAGCTCAGTGA---GGACATTCCCAGCG-CTACCTGGAT---------CCGGCCAGA

CGGTATCTTTCAGC--TAGGTGGAGGTTCTCGCTCTTCTTTCAACCCCACGCAAG---CC

TATCTGACTCTGCAGCAAGCCTCGACAGCGCCTAGGGCTGGAGGAATCGGCTCTGTGCAG

TTTGTG-CGTGAGTTTGTGCCTGAGGTTTATCTTA-ACCCTTTTTCTGGCCCCCCAGATA

CCTTCCCCGACC---AGTTCA------TTCCTAACTACGATATTGTAACCAACTCTGTCG

ATGGCTATGACTGAGCCCTG---C--------CTGGAACAGGTGGAACT-TGACTGCCTC

TGTGCCCGCCATAAAGAAACCTGCACTCAGCCAAGA-TGCTTTGCAAAGGACAATCTCCG

-CGCAAAGTGGTTTTACC-------ACCCCAGTGTTTTCG---CAGACATGGAC-ATTGT

GGACTCTTACCAAGAGGG-----TCATGGGCTAACTATCAGTGTTGA-----GTGCACCT

ACCATTGTTCTTCCCTGAAAGTGAGAGGGCAAGAGCT---TCGCTGCTCTTCCTCTCATA

AAGGCTCTGA-CATCAAAATTCGTTGCCTCTGTAAAAAACCTGTTCCCCATTCTAGCTTG

G---TGGATGCAGCCTGTACTATGTATAACCTTCATTAA----------------ATTTT

TTC--TACAGCTACT---------CCCGCTATGGCTCTGTTTCTTC---TTCTTCTGGCC

GCCGTCTCCCTCGCGGCGAGTGAACC-------TGAAACTCAGCCAGAGCACAT------

------------------TGTCAGCTGCTGTGCGGGAAGTCCCTGTAGCTTAGACCTGA-

--CTGTCAGCTCAGCAGCTACTG-TTAACTGGACCGACCCAGAGACTGGAAGCGCGCCAG

CATGT---------------TTCAGCTCCGGCTTGTGCAATGCGACGCAACACGGTCTCT

TCTTCTCTGCTGACTTCAGTGAAGATGGGCCA------------------TATCTCGCTT

CTATT--------------------CATGATCAGGGCTATCTCGGGCAAGAGCACTTT-T

A---------CCTCCTCTACTTC-----AGAGACTTGTGTCCCAT-AGGGGGCAAAGATA

AAGATTTTCCACTGCATGCAGA-GGAAAGTACTGAGGTTCCAA-------TTGCCAGGCC

TTTTCATGAAA---------------TGAACTTGCAGACTGTAATACAGAGTGGA--ACC

CTGTTTAATC----CACTTCTCCTGGAACTGCCCCCAGAAACAACTCAGAATGTCAGAAA

TGTGCGCTGGTACAA----AATCATTGGGGACTTTCAAGCAGAGAAAGTCTCTCGGGTGC

GCAGCAAAG----------GGCGTATAGACAACGTGCAGCCCACTCTTG-----CCAGAG

CTCTCATGAGCGGAGACTTGTGGGTGCTGCACGTGTCTC----CTTATACTCTGGGCCCA

TGGCTAGCTATTA--TTCAACATGCTGGC--GGGCGTTTTGAGTTTGTGAAGTTTAACAT

TACCGTCCCTGCCTGGCAAGAGCAGCT---AGTGCACATATTTTCAA-------------

-------GTACCATAGAAGAACACACCAACTAT---CCAGATCCAACTGATTTTACTAGA

AAACTCAACTGGGCTCTCTACAAGCAG------CAAAAGAGAGGTGCCTTTAGAGTGGTG

TGTAATGTCACCA---GTGCTTTCCCAAATTGCTTGGGAGAAATAACTTCCTCAGACACT

TCTGTTCTAATCGGGGAAAGAAGAGACAGCCTTGCTGTTTCTGTGCTTTCCTTTTTTCC-

--CCTTGA---------------CCTG------CCCGAAACT--CCCAGGTTTGAGGCCC

TTACGAGCCAAGAACCTGCCATGCCTACCTATGTCCCTTTGACTTTCTTAGTTAT-TGGG

GTTTTG---ATGACTCTTCTAACTCTCCTAGGACTATG-CTGCTGCCTTAGAAA--CAAG

ATCCGCCCTATT--------------TACTTTCCCCCCACCACTTCTCTGTAACAATAAA

A-------TCTACCTGATTT--TTTGCAT-----TACTTCCGTGTCACGCGTCTCTATGT

CGATCACGGCTCCTTCCT--------------------------CCCAGCTTTGGT---A

ATTCCACTTCTCCGTCCGTGCCAATTTGCGCAGCCCC--TTGAATGGCAATTTGGTTTTT

CCCA-----------ACAGCTCTCCGTTGACCATCACCAACGCCA--TGAAGAGA-GCGC

GA---GCTTTACCTGCAGATTACGACCCAGTTTACCCTTATGAG----------CAACCA

GCGCCGCCTAC----------ACAGCCGCCCTTTTTTAA-------CAGGAACAAAGGAT

TGACAGAGTCCCCCCCGG-GCACACTGGCGTTAAA----------TGTCACCAGCCCCTT

AGGTTTTACAAACACTG-----------------------GTCAACTTAAAGTGAATACA

GGAAATGGACTTAAAC--TCGATCAGGGAAACTTACAAGTGAGCCTCGGAG-CAGGGCTC

ACTACC-AACAGCCAGGGAGAAATTAC-----------TCTGCAGCAGCCCAACACCGCC

C---TAACCTTTACCTCACCTCTACA----------CAAAAACAAC--------------

-GATGAGGTTTCCT---TAACCATTGGAGATGGCTTACGT--------GATGACAATGGC

ACTCTCAAAGTTACC-----------TTTCCTCCCCCTC---------CCCCACCTTTGT

CTTTTA------CCAACCC-TCTGTCATTAAGTAACAACT---CAGTATCGCTGCAGGTA

GGCGCAGGC--CTTCAGGT------------------------TTCGGGAACAGGGCTGT

CTACTGTGCCTGA---AACTTACAATGACCC--TCTTGAAAAAACTGACCAAGGGGTTAA

TCTAAAGGTGGGGGCAGGGCTCACCGTCTC--C---AGTGGGCAGTTGCAAGC-------

--------CGTTCCT------------CCTCCAAGTG---TCACTTACTCTGCACCTCTC

GCGAAAAATAAC--AACACAG------------------TGAGCTTAACCATTGGAACAG

GACTTCAAAT---------------ACAAGGAAATGCC--CT------------------

-------------------------CACCGCCC---CT---CCTCCTC---AAATTCCTC

CCCCA-CTG-------TCTTTTTCTGCCCCG--------TTGA----CAAAAAACAAC--

-AAC-AGTGTA-------------------------------------------------

----------------------ACTTTGCAAGTGGGAAGAGGTCTAGCTGTGCAAAATAA

TGCTTTGGAATCGACAGCGGAAACTCTCT-CATTT---------ACCGC--CCC------

---------------------------------------CTTAGCCAAAAATGG------

AAC----------TA---------CTGTCTCTTTAAGTGCCA-ATAACGGCCTAACGGTA

AACGG-----AGGCAACCTAGCTGTTAATGTTGGACAAGGTCTTCA-----------GGT

GACCAATGGAGCGG--TAACCGCAAAAATAGGCCCCACTCTAACATTCA--------ACA

ACGGAG----CCATTGAAGTA--------------------GTTAGGCCTCC--------

---TTCTTCTACTAAC------------------GTCACCTTGTGGACAGGACCTTCTCC

TAGTGTAAACGCATCTATC---AATGGCACACCAGTTATAAGGTCTTTTGTCTGTTTGTC

CAGAAATTACAATTTGGT-CACCCTAACAGCTA-----ACTTCACTGGGGAAGGAACGTA

CAGATTGGTTTACCCCACCCAATCAAAGTTTAGTTTAATCATGGATTTTGATCAATTTGG

GCAGCTCAT---------------TTCTACGGGAAACATAAACTCTACTACTACTTGGGG

AGAAAAACCTCTGGGAGACAAT--ACAGTGCAAGCTCATCCCAGTCACACATGGAAACTC

TGTATGCCTAATGCTGCAGTCTA--------CAACAAAGCATCTACTCCTTGTGTGTTTT

ATAACAGAATCAGTCTTGACACCAAAACTCTGGATGATACCGCGAACAGAAATATAGATT

GTTTAGTGCTCTTGAATACCTCCCCCACTT---CAATAGCTGCCT---ATAGTATAACTT

TTCGCTTTCTGAACTTCAACAGACTCAGTG---GAGGTACTTTGTTTATAACTG--ATAA

TATCAGCTGTTCTTTTGTTGGCGAAAACCAATAAAGACAT--------------------

-----------------ACAAAAAATACATTTAAAAATTATTAA---ATTTTATT-----

----------GAAAACGCGA----------GTGTTGATACAACCCCCCTCAGGAAAGGAA

ATTTTATA-CACAGTTCTTTCCCCACAACAGTTTACATACAGTGAATGTTGGGACAATTT

-----TTCACCCACAGGTCTATAGTACACAA--ACTCTTTACAGGCCAGCCTTGGATCAG

TTAGGGAAATAAATTCTACATCTGAAG-----------ATTGCAGACAA-TAGCGAGAGT

CCA--AGC-TTGCACGACCGCTGGCCCGAATCTAAGAAGGAATAAAAGCACTCAAGTTTA

TGCATGAAAGGCTTCGAATTAGGCGG-----CATTTTCCATACAGCAGAGATTGCCTGAG

AGCGTGTCTTC-TTCTGTCTTCAGCCC-GGGTGGTCAC-ATGCAGAAAGTCCATAACATC

CAGAGAT------TCTATAGTTTCCAGCAGGCGCCTAATAGTGCCCCTAACCT--TAGCA

GCACACTTTCTCAGAGACTGTAACCTATCTGCAGCACAGCGGTCACAGAACAACAC----

------------TAACCAAAAATTGTAAGTCCCCTTAAGAAAAAAGCCCATCTCTGGGCT

TAGGCAAGTGCGAATAACATCAAT-ACAGGCAGCCTGAGCATC-AAAAGGAAAACACAAT

CTAAAATATAAATAATGTCTGGCGTTGTACACCACACTGCCCACATA-ATTGATGTCATC

TGGGCGGCCAGAATTAACAAATTCTCTGAACCACATGTGCAGCCTATTCATGCGGGC---

-------------CCCCTTTATAAAATCTAGAGCCACCTTCCTAATGACCTCTCTGCTGC

ACAGGCATTGCAGGGAGTGCTGGTCAT--CGCAGTGGCAGTGTAGGCGCCACAGCTGAG-

-GTTCTGCCAAGCATGCATGTCTGTGTAATTGTAACTCTAAACCAGCGCAGCAGGTAAAC

TTGCCCAACA--GTATTTTTTCATGTATAGTTAAAATTTCATTCCAAGGAATAGGAAGCT

CATAGCACAAAGCAAAGCTATTACAATAGGCGGGAGCTCTCA------CCACTGTAGTCA

CGTGAGAGGGGGC---TTCAGT--TCCAGACCGTGGGCAG----------CTCAT-----

-CTATGAACAAAAACTAAAAGTTTCAGATATTTGTACTGAAA----ACAGCTCCCTGTCT

CCAAAATCAAACTTTAGATTCTCATCAGCCTGCAGTGCAGCATAGAGCTC----TGAAAT

TAGAAGAGTTTTGTACTTAGACA-AAGCTGACGCTCTGTC---TTCAGCAAGTGGGCTAT

GAAAAACAAA---TTTAAAAGTGATACCG-GGCTTGTCTCTGTCAGTGGTTTTCAGCATC

ACCTGCTCAAAGTGAAAGCCGGTGACATCAAACATCATGTCAAAAAGAAAAGCATAGGG-

-GTCTTCCACATA----GCTTGAGACTTGTCTAACAAAATCTTCAAAAAAAGTAACAGTT

CCTCTAATGCATGGAGGTCGGCTGGGGCAGAGAGGCGACGCCATCTGG---A-AGATCTG

AAATACATT--TAACA---GAAACTTGCTCTAGATCTACAGTACTGAGATAGACCCCTTT

GGCAGCACAGGAAGACTGCAAAAAGTTATCCACAAAGTTCGC----AATGCTGTTTTGTA

TAATTTCAACCAGAGCAGAAGTAGGCTCTAAGAACA------GCCAGCAAACG--CCCAC

AAACATGTCAATTTCAGAG----CTCTGCTGCTCTTCTAGAGTA--------ACACAAAT

ATTTTGCAGCCATAATTTCAGAAACTGATCCACGCTAGCTACTAAAAGCTGGGCAATCTC

AGGATTTACAGAGA---GTAATTTGTTAACCAATTCAGCCTGCAGATTAACGAGAAAAA-

---------------------------GCATGCACTTGGGGCATATATCATCAGCTGACG

CGG------------------------------------------------CAGGCATCC

TA-------------------------------------------------------TAA

G------------AAAAAGGAAAAA-----------------------------------

-----------------------------TCAGCAACTAAAGAAGTGAACTTTAACCTCA

TTAAGCCAAAAAGGAT---------------GTAAGTCCTCATTAATGCCAGCACCCAGG

CCCTCAATCCAGCCC-CGTATGT-AAACCTTAATAAGAGATTCAACCTCAATTTTCAGAG

CAGTAGACATTTCATGCGTACTAGCAAACACTATAGAGCTAACAATTGAATTGCCACTGT

GAGAGGACAAGCGCCTAAC-AGCTGTCAAATGAAAAGATGAATACAAAAACATAGAAATG

CCGCGGCAAAGATCTGTTTCTTGGTCT-----------TTGTCTCTTATTAAAGTAGCCA

GGTAATTTTTTAAAGTGAGATGGCATTCATAGAAG-TATCGAGAAGAGCCGGAAGC-CAT

TCTG-AAAAGAAA-AAGGAAAGACTTCTAAAAAACATGCAGAGCACCGCCAC-CAAAAA-

ATGGA----------AAAGCCAAACT--CAACA-TACCTTACTCAGTG--ATGACCAAAA

TGTTAGGTTCC---------CAGGAAACTGTGAGGTT--TTCCAGCAGCAGGTGCGAGTC

CTCAGGCAAAACCAAACGAAAGTAGCTTCGCAGGTGGTCCTCAACTCCCTGAGCAATGTT

CTGCAGGCCAGGCACCGAATCAAACGCCTCCGCGTCGCTGC-CAATGAAAAAATAAAACT

CCAGGCTATTACCCCCTAACAC-AGACACAGCAGCTA------CATAAGTAGGTGAAGGA

CGAACCGCCTGAGCCC-TAACTATGGCAAAAATTAGACGACGCAGCTCTGAGGGTAAAAA

TTCTACC-AGCTCCATGTGAGAACACACAGC--CTCAATAATGTCTTTGTGAATTCGAAC

ACAAAAACAGGACCCCATCGAAAGACCCAAAGCAGCCATA--CTCACAGTAATCGGAGAG

GTAGAGCA-GAGCTGAGAGGCTGTGAATGGCAGCACCGGAGAATAAGTGCTGTGGAAC-A

GAAATGTACAGCAGTATATATATCATAAAATGGGTGGAGAAACGAAAAAGGAGACGGAAA

GTGACCTTTAGAACGGGCAGTTCCTGAAATGTGGACAGCGCCCAGGATTTACCGCAACCG

CATGTTCCCCTTGTCAGCATTAATCACTTTCTGGGCACACAGCCCGGGAAGTACCGGGAC

TCTCCCGTAAA--TCAGGGTCAT-AAATTTCGCTCTCGTGAAATGGAGCGAGAAACCACA

AAGGCGCCTCGCCCGCTGGCCACC-TGCCCCGGTTTCGGTCTCCGCCCCTCATAAAA-CT

TTACGGCGGAAAATCACCCGCCTTTCCGCGGAACGCCCCCGTCGTAAAATTCC---CCGC

GCG-CTCTCGTCGCACGCACACCCAAAAATGAGGAAGGGGCTCACAGAGACCACCAGGGA

CCCCCAA-TTTCTAGGCTGC--CCGCCAAATTTAGGCCACACCTCTTTGTCCTGTATATT

ATTGATGATG--------------------------------------------------

------------------------------------------------------------

------------------------------------------------------------

------------------------------------------------------------

------------------------------------------------------------

------------------------------------------------------------

------------------------------------------------------------

------------------------------------------------------------

------------------------------------------------------------

------------------------------------------------------------

------------------------------------------------------------

------------------------------------------------------------

------------------------------------------------------------

------------------------------------------------------------

------------------------------------------------------------

------------------------------------------------------------

------------------------------------------------------------

------------------------------------------------------------

------------------------------------------------------------

------------------------------------------------------------

------------------------------------------------------------

------------------------------------------------------------

------------------------------------------------------------

------------------------------------------------------------

------------------------------------------------------------

------------------------------------------------------------

------------------------------------------------------------

------------------------------------------------------------

------------------------------------------------------------

------------------------------------------------------------

------------------------------------------------------------

------------------------------------------------------------

------------------------------------------------------------

------------------------------------------------------------

------------------------------------------------------------

------------------------------------------------------------

------------------------------------------------------------

------------------------------------------------------------

------------------------------------------------------------

------------------------------------------------------------

------------------------------------------------------------

------------------------------------------------------------

------------------------------------------------------------

------------------------------------------------------------

------------------------------------------------------------

------------------------------------------------------------

------------------------------------------------------------

------------------------------------------------------------

------------------------------------------------------------

------------------------------------------------------------

------------------------------------------------------------

------------------------------------------------------------

------------------------------------------------------------

------------------------------------------------------------

------------------------------------------------------------

------------------------------------------------------------

------------------------------------------------------------

------------------------------------------------------------

------------------------------------------------------------

------------------------------------------------------------

------------------------------------------------------------

------------------------------------------------------------

------------------------------------------------------------

------------------------------------------------------------

------------------------------------------------------------

------------------------------------------------------------

------------------------------------------------------------

------------------------------------------------------------

------------------------------------------------------------

------------------------------------------------------------

------------------------------------------------------------

------------------------------------------------------------

------------------------------------------------------------

------------------------------------------------------------

------------------------------------------------------------

------------------------------------------------------------

------------------------------------------------------------

------------------------------------------------------------

------------------------------------------------------------

------------------------------------------------------------

------------------------------------------------------------

------------------------------------------------------------

------------------------------------------------------------

------------------------------------------------------------

------------------------------------------------------------

------------------------------------------------------------

------------------------------------------------------------

------------------------------------------------------------

------------------------------------------------------------

------------------------------------------------------------

------------------------------------------------------------

------------------------------------------------------------

------------------------------------------------------------

------------------------------------------------------------

------------------------------------------------------------

------------------------------------------------------------

------------------------------------------------------------

------------------------------------------------------------

------------------------------------------------------------

------------------------------------------------------------

------------------------------------------------------------

------------------------------------------------------------

------------------------------------------------------------

------------------------------------------------------------

------------------------------------------------------------

------------------------------------------------------------

------------------------------------------------------------

------------------------------------------------------------

------------------------------------------------------------

------------------------------------------------------------

------------------------------------------------------------

------------------------------------------------------------

------------------------------------------------------------

------------------------------------------------------------

------------------------------------------------------------

------------------------------------------------------------

------------------------------------------------------------

------------------------------------------------------------

------------------------------------------------------------

------------------------------------------------------------

------------------------------------------------------------

------------------------------------------------------------

------------------------------------------------------------

------------------------------------------------------------

------------------------------------------------------------

------------------------------------------------------------

------------------------------------------------------------

------------------------------------------------------------

-----------------------------------------------------

>CAdV1

------------------------------------------------------------

------------------------------------------------------------

------------------------------------------------------------

------------------------------------------------------------

------------------------------------------------------------

------------------------------------------------------------

------------------------------------------------------------

------------------------------------------------------------

------------------------------------------------------------

------------------------------------------------------------

------------------------------------------------------------

------------------------------------------------------------

------------------------------------------------------------

------------------------------------------------------------

------------------------------------------------------------

------------------------------------------------------------

------------------------------------------------------------

---------------------------------------------------CATCATCAA

TAATATACAGGACAAAGAGGTGTGGCCTAAATGTTGTTTTTTTTATACAGGACAAAGAGG

TGTGGCCTAA----ATGTTGTTTTTTTTTAAAAAGTTTTTGTTTGATTGTTTTGACAAGG

TCACACCCTGTTCAGGGCGTT-TCCCACGG-GAAAGACCATGACGTCAATT-GGGTGTTT

T------TGTGGACTTT----GGCCCG-----------CGAGCCCGTTTTATGACTGTTT

ACTGAAATTGTGGTTTT-TGGGTGGAGTGTTTACTTATGTCAGAGGAAATTTGCTGGGTT

TATTGTT--CTAGCCTTGGAGCCAATTT--TCCACACCCGGCGCCAGACTGTCTGACATT

GTTTTGAGAAACTC-----TCCGTTCCCAGGGACACAGGACCGCACCCGCGAGAATG--G

TCACGCCTTCTG-------TTTTTTCT-----------GCTTTAAATTCACTGCAG-AGA

CGAAAGAGGAGT-----ACTCTTGAGTGCGCAGCGA-GAAGAGTTTTCTCTCAGCG----

-------AA-TCTGCCAAAA----ATGAAGTTAACTCTTGAGCCGGCACCGCGCT---GT

CTTCATGAATATGTTTCTCAGCTACTGGAAGATTGGCAGCCGGAATGT------------

CTCTCCTGTGAGTACTCCCATGGT---GGCTCGTCACC---AC-CGAGTCTTCATGATCT

TTTTGATCTTGAGCTGGAAA-------------------------------------ATT

CTCGTTCTCCTTCTCCGTTGCTGTGTGA------TTGGTGTGCGGAGGC-----------

TGACAGTGAATCTACAATTTCCACGGAGACTGATGTTGGCTTTA-------CTTTGAACA

CTCCGCCGGTTTCTCCTCTTCCTTCGTATTCCACTTCTCCTGC---AAGTATTCCTGAGG

ACATGCTGTTGTGTCTAGAGGAGATGCCTAC-------CTTTGATGACGGGGATGAGGTT

CGGAGTGCGACCACCTCCTTTGAGCACTG--GGAAAATAACTTTGACCCTAATGTTGGGT

CTTTTTTTGGTTGTCTGCGTT-------GTGCCTATTACCAGGAGCAGGGGGAGAATTCC

------ATTTGTGGGCTTTGTTACTTAAAGGCCCTTGCTGA----AGGTAAGATAT----

TTTAAAT-TATGGGTGGGTCAAT------------AGTTTCA------CAAAGGTTCT--

--------TATGTG----------TTCCTCTCTG--------TT---TTAGTGCCCTGCG

CCA----------------TGCCTGTTCGCGCAGAACCTGTTGATGCTGAT------GCC

GGAGAGGA---TGATGAGGTCAT-----------TTTTGTGTCTGCTAAACCTGGTTCCA

GAAAGAGGT------CAGCGGTGACTTCCCGC---------------GGCTCAGTTGAAA

GCAGTAAACGCCCTTGCTTGCCAG-----------AGCCTGAACAAACTGAACCTTTGGA

TCTTT----------------CCCTAAAGCCACGCCCG----------------------

------CAATAAATTACTCTTGA-------------------------------------

---------------GTAAACAATGTGTTCATTTCTGTGTTTCATT--TGTGGGCGGGTC

TAGAGTATATAA--------------TGATCCAGTTCATTCTGTGACTCATTAGGCTTCT

AGATCTAGC--------TAGGAGCAGGTAG-TATGGACCCTCTT-AAGATCTGTGAAAAC

TA-TTTGACTTTCAGGTCTAT-AATCAAAGGTAGCACTTTTTCGCCAGGCGTGTTTAGGC

GGTGGCGCTTC---CATGC----TTTGGCCGATGTTGTCGGTAACATAGTGGAACGGGAG

GAAGGCAGGTTTTGGGAAATTGTTCCTGAAACCCACACTTTGTGGGCGCTTTTTCGAGGA

GGCTTCACCGTTGCTCCTTTTACTGAAATTCTTACATCACTGCAGCTTGAAAACAGAGGC

AGGCAGTTGGCTTTTTTAGCTTTTTTGTCTTTTTTGCTTCGCAACTGGCCTTCAGAC-TC

AGTAGTGTCTGAGGACGCTAGACTTGACTTGGTCTGTGCGCCGGCATGGAGCAGGATTCA

GATCTGGAGTCA------GGC---------------CGCGCGACTAATCAACGA------

--------------------------------------CCTCCCAGAGTCCGT-------

----------GTTCGA---GGGGCAGGGGTCCGTGGTAGAGGA------AG---------

--------------AGTGCGGAGAAGAGCACTTAGCGAG-GGACAGCGACGATCCCCTTT

TCGACTAGACGATCTGCAGCTTCCAGATTCCCTGTATGTT------ACGCGAGCTCTGCA

ACGGGACCATGCTTTGGAA----ATGCCTAGAGGGCAGGTAGATTTTAGCTTGATTGAGG

CTGAGGAGAGGAGGGCCGGTCCTACGGACGAGTGGTATTTTGAATCTGTTAAAACTTACA

GG---GCTAAGCCGGGGGATGATCTCCAGACCTTAATCAAAAATTATGCCAAGATTTCTC

TGGAATGTGGGGCTGTGTATGAAATAAACTCTAAGATTGTGGTGACGGGGGCTTGTTATA

TAATTGGTAATTGTGCCGTACTTAGGGCTAACTTGCCGGTAGGAACTGCAATGTTTGAGG

TTTTGAATGTA---GATGTTATTCCGTCAATTGGTTTTATGGAAAGGATAGT-GTTTTCA

AATATTCTTTT-------------TGATTGCAGGAGTACCACAGCTGTAGTGTGTTGCAT

TAGTGAAAGAAATACGCTCTTTCACAATTGTGTCTTTTCCGGTCCACACATGCTGTGCTT

G--GATATTAGGGCGGGGG------------CCGAAGTGAGGGGATGTCATTTTGTGGGT

GCGGTGTGTGCTCTGCGTAGCA---AGGGTTTGTA-TAGTGTTAGAGTGAGAAATAGCAT

CTT-TGAAAAATGTGCTTTTGGGGTAGTGAGCGGCTCCAAGGCTTCCATTAGCCACAGTA

TGTTTAAAGACTGTGCTTGCT---GTATTATGTTTGGGGGACAGGGCACTATCGCACATA

GTCATTTTATGGCGACCACTTGCACTGATACACCTATGAACCTGCAATTATGTACTTGTG

AGGGGAATGGAAGTCATGTGGTTCCTCTGGGAAATATTCACTTTGCTTCT---AATCGGG

AAGCCCCGTGGCCTACATTTAACGCGAACGTTTTGGTTCGGGTACGTTTATAC-ATGGGC

CGGCGCCGGGGAGTGTTTCATCCTA---AGCAGTCAACATTTTC-TATGTGTGTCATTGC

CGCTCCAAGAGGGGTTGTGCAAAGA---ATCTATC--TGTTCAGTGTGTATGATGCAACC

TGCGCCATTTTGCAACT-------GGGCGAAGCAGGT---GATGCTGCTACCGAAAGACT

GTGTACTCGCGGTATGAGGCATAACACCCCTTCTTTGAGGGCTGCTTATGTTACTGAC--

-ACGAGGATTGATCGGGAAATAAAT--TCCCAAGACACGGCGGAGTTTTTTAGCAGTGAT

GAAGATAACTTA--TAA--GGTTGGTA------------TGGGCGTGGTTTTTGGT----

-------AAGTATAAAAGGGGCGAGGCACGTGGCTAT-AAAT--------ATATTTACAG

CCATGGACCC---------TCAACAGAAGGGGATTGTGAATACATGTTTTCTAACAACGC

GCATTCCATCCTGGGCAGGAGCGAGACAAAATGTGACCGGATCAGATTTAG--GAGGAAA

GCCCGTGCCTTCAGATG-----TGCTGGAGAGTGGACGTCCG----C-------------

-----TTGCAGCTCCACGCGTCAGGACTTTGTATGA-GGAG-CAGCAA------------

------------------------CTGAATATGCTTACGGTGAATGTT------ATT---

----------CTGGACGATTTGAAGACCCAGGTGGCCGCCATGCAGAACTC--TGTGACA

GCCATT---------------CAGGAAGAACTTAAAG-------------ATCTAA----

-AGCAACGAGTCGCAGCCCG--------------------------------ATAA----

----TGTTTT--CAATA-A-AAATTTATTT--------TTTCTTT-GAATGATAATACCG

TGTCCAGCGTTGTCTGTCGGTAAGGGTTCTATGAATTCTTTCTAGGGCTTGATACAATTT

ACCTTGAATGTTAAGATACATGGGTATAAGCCCCTCTTGTGGGCTTA-AGTACATCCAAT

GCATACAGTTGTCAATGGGTTCAGTATTATATACAATCCAATCAAAGTTAGTATTTAGGC

GGTGGTAGTTAAAGATATCTTTAAGCAAGAGGGAAATAGCAGTGGGTTGTCCCTTGGTGT

AGATGTTTATAAATCTATTCAGTTGAGAAGGGTGCACTTTTGGACTTATCATGTGCACCT

TTGCTTGGATCTTTAAGTTGGCAATGTTTCCCCCCTGATCTCGTCTTGGATTCATGTTGT

GAAGAACCACTATAACAGAATATCCCGTACACTTTGGGAACCTGTCTAACAACTTAGAGG

GGAAGGCGTGGAAGAATTTTGCTATGCCCCTGTTTCCGCCTAGGTCTTCCATGCATTCAT

CCATTACAATGGCTATTGGACCTTTAGAAGCTGCATTTGCAAAG-ATATTGTTAGGGCTG

GTGACATCGTAGTTTTCATCTTTGGTAAGCTGAGCATAAGACATTTGTATAAACCTGGGC

ATGATGCTTCCGCTCTGAGGAACTACCGTGTTTTCTGGACCAGCAAGGAAGTTGCCCTCA

CATATTTGGGTTTCCCAGGCTGCAATTTCTTGGGGAGGGATCATGTCCACCTGGGGAGTG

ATAAAAAAAACTGTTTCTGGTGTTGGGACTATAAGCTGGGCGGACATGAGGTTTCTAAGG

AGTTGGGATTTTCCACATCCTGTGGGTCCATATACCGCAGCGATAAAGGGTTGCTTGTAG

TAGTTTAGGGATGTGCAGTTGCCTTCTTTATTAAGG------------------TATTTA

CTGGCCCTAATACAACATTGTGTTATGCTATCTTGAATGTCTAGCAAATCATTCAAT---

-----------AAAGAATCACCTCCCATAGA-----------------------------

AAGTAGTTCCTCAAAAG-AGGTAAAGTTTTTAAGGGGTTTTAGTC----CTTCTGCATA-

----TGGCATTTCTGTT-----AGACTTTGTTGGAGTGTTTGCAACCTGT-------CCC

AAAGATTTTTTAAGTCA----TCCACTGCAGCTCCATCC----------ACGTAGTTTCG

TTGTTTCTTG--------GATTTGGATACTTGCGACTGTATGGGA--TAAGCCGATTCAT

GTCT---------------------AGAGCATGTAGGGTTTTGTCCTTCCATGGCCTCAG

CTTCCTTGTTAAGGTGGTTTCTGTCACTGTGAAGGGCTGGACATGGGTCTGCACACTGGT

GAGGGTCCTGCGAAGGCTCATTCTGCTGGTTTTGAAGATGTC------------TGAGCC

CTGCTGCAGGTCGGCATA---GTAGCAAGCCTTTAGCACA---TCGTAGGAGAGGGTGGA

GGTGGCATGACCTTTTGCCCGTAACTTGCCCTTGCCCACG------TATTGACATTCTGG

ACATGTGGTGTCCTTTAAAGCATATAATTTTGGCGCCA-GGTACACTGATTCTGAACTGT

ATGCATCCGCCCCACATTTCTCACATTTAGTTTCACACTCCACTAGCCAAGTGAGTTCCG

GGTTGTTGGGGTCGAAGACTAGATTTCCTCCATTTTTTTTGAGTCGTCTCTTACCTTTTT

CCTCCATTAGTCTGTAACCCTCCATGGTGGTAAAGAGGCTGTCGGTGTCCCCGTAGACAG

ATTTTAGGATTCTCTTTTCCAGTG---GAATTCCAGCGTCGTTTTCATAAAGAAACTGAG

ACCACTCTGAGACAAAGGCCCGGGTCCATGCTAGAACAAAGGAGGCAATTTGAGAAGCAT

ACCTGTTATTGGTTATTAGGGGGGTGGACTTTTCCAAAGTGTGTAAACAAAAGTCATCAT

CCTCAGCATCAATAAACATGATTGGTTTGTA---ACGATATG--ACGTCA--------TG

TTAAAATTT-----------TGCATAAAAGGGCCTTCAGCGTGCGGGTTTTCAGTTGGAT

ATTCTTCGCTGTAAGAAGGCGGTGCC-----------AACCGGTGGACGTCAA-------

------------------------------------------------------AATTGA

CAGGTGGGTACGCTACGACAAA---TTCTGGCATGATTTCTGCGCACAAATTGT------

------------------CTGTTTC----------TATGTATGAGGATGAT--------T

TGACTACATAGGCTCCAGAAGCAATTTCTTTAGCAATGTTTGACTCTATTTGATCTGAAA

AAACTGTTTTTTTGTTATCTAGCTTGGTAGCAAATGAGCCATACAGGGCATTTGACAGTA

GCTTGGCGATGCTTCGCATGGTTTGATTTTTGCTTTTGTCTGCTTTTTCTTTGGCGTTAA

TGTTGAGCTGCACATACTCTCTGGCAAGACATTTCCATTCTGGAAACACAGTGGTTCTCG

TGTCCGGGATAAGGGTAACCTTCCATCCGCGGTTGTGTAGGGTAATGACATCAATGGAGG

TTGTAACTTCCCCGCGCAGGGGCTCATTTGTCCAGCAAAGTCTGCCCCCTTTACGTGAGC

AGAAGGGGGGAAGCACGTCCAAGAACTCTTCTGCCGGTGGATCGGCATCAATGGTAAAGA

TGCCTGGAAGAAGA-TCTTCATCAAAGTAGCTAATAGTTTTATGC---TCTTGCATTTTA

TGTTCG---TAAGCTTTTATGGCCACTGCTCGCTCATAAGGGTTTAGCGGCGAGCCGGCG

GGAAAAGGATGTGTCAGAGCGCTAGCATACATGCCACATATGTCATAAACAAATACTGGT

TCTTGCAACACTCCTATATAAGTGGGGTAGCATCTACCTCCCCTGATGCTAAGTCTTACA

TATTCATACATTTCATTGGAGGGAGCCATGATT------ATCGTGTTTAGATGGGGGGCG

TTTGGTTTTTCTGCTCTGT-----------------------------------------

----------------------------ACAAAATTTGTTTAAAAATAGCATGGGAGTTT

GAGCTGATGGTTGGACGCTGGAAGACATTAAAATAGGTTTCAGGTAAGTTAACAGAGTTT

TTTATAAAAATCTGGTATGATTCTATAAGCTTGTTAACTAGCTGGGCTGTTACTTGTACG

TCGAGTGCGCAGTAATCAAGGGTTTGCTGGATTATGTCGTAAGCG---------------

---------------------CCCTTTTTCTCTTTTTTCCACAGGTCTTTATTAAAGCAG

TATTCCTCCTGATCCTTCCAGTACTTCAAATCCGGAAAGCCGTC---GGCATCTTGC---

TGGTAAGAGCCTAACATGTAAAACTCATTAACGGCCTGATAGGGGCAACACCCTTTGGAA

ACCGTTAGGCTGTATGCTTTGGCAGCATTTTTGAGGCTTGT---GTGCGTTAGAGAAAAA

GTGTCACGCACCATAAATTTTAT-------------G-TACTGGGTTTTTAAGT--CGGA

TGCCAACACCTGGCCCT--GCTCCCAGTGTCTGTAATCTTTG------CTAGGCACGTAG

CTG---GGATTAGGCAAAGAGTAAGTGATATCATTAAATAGCAGCCTTCCAGCACGTGGC

ATGAAGTTCCGAGAGATGT---CGAACACGGGTGGGATTTCTGCCCGTGTGC-------T

AACCACTTGAGCCGCTAATAAAATTTCATCAAAACCTGTGATATTGTGACCCACTACATA

TACTTCAATAA--ATCTTGGAGTTCCTTGAAGTT--------TAAATTTTT---TTAATT

GATTTGCAGTGATGTCTTCAGGGTTCTC-AAGGCC---CAATGCGGCGGCCTCTTCCATA

AGCTTGGGATTTTGACAGAGTACGTGGGACCATAGGTTGTTAGCG---AAATGCTGCTGT

AAAGTGTCTCTAAATGTTTTGAATTTTACTCCTATGGCTCTTTTTTCGGGAGTGATGCAA

TAGTAAAGAGCGGTTTGTTCATTACTGTTCCAAGTGTCCCAGTTTTGTGTTGTTGCAAGA

TTTTTAGCAGCATTTACCAGATGCTCATCTCCCAGTAATTGAAACACTAGCATAAATGGC

ACTAGTTGCTTTCCAAACTTGCCATGCCAGGTATATGTTTCCACATCATAAACAATAAAC

AGCCTCTCTGTGTTTGCCGGAGCTCCAATTGGACTGAAGGAGATTTTTTCCCACCAGTCT

CCGCTGTGTGAGGCCACGTGATGAAAGTAAAAGTCCCGTCTTCTAAGTGTGCAGGTGTGA

GTAACTTTGAAAAAAGACCCGCAAAACTCACACCTTTGCATTGGGGAAATGTCTTTTATT

-AGGAAAACTTTGCCATGTT-----TAACCAAAAACTTGAGTGGAAAG------------

------------------------CTGAGCAGCGGAATTTCCGCTCGAAACTCCACCT--

------------------T-------TCTAACTGTCCCTCGCTGATAGTAG---TATATA

AGACTGTAGCTTGCCGCAGCCTCTTCTATGAGGGTGGGTAG------GTTGTGGACGG--

-TGATA---GGTTTGTTTAAGCAGGCGGGTAAGTCATA---GAGGTTGGTGTTGCAAAGG

TTAGTGAGGGCACTGGCCAAGTTGGAGTGATATTTAATTTGCACACACTGTCCATTGTCT

A---ATATGGCCGAAATGCTTAGGGTGGCACGCTGAGCTACTACAGCACCTC--------

--------------------------------------------------------TGTA

CTTGCG--------------CTTGGTTGAGTTTA---TGGCGGGAGTTGCACCT---GCT

CGTGCTGAGCTGGCAGAGGCCTGTGTTGCATCCTGAGCTGGGAGGCTAGGGCCACTACTC

TCCTGTTGATTGATTGAATTTGGCGGTTCTGTGAAAAGACCACTGGTCCCGTGACTTTGA

ACCTAAAAGAAAGTTCCATAGAATCAACCTCTGTGTCATTAA-TGGCCACCTGCCTTAGA

ATTTCGCTCACATCCCCACTATTTTCATGATAGGCAATGTCTGCCATAAATTGTTCCATT

TCTTCTTC-----TTCTAGCTCT-CCTAGCCCGGCCCTTTCTACTGTAGCGGCCAGATCA

CCAGACACGCGAGCCATAAGCTGGCTGAA------AGCATTTTCTCCCATTTCATTCCAC

ACTCTACTATAAACTACCTGTCCTTCATGATCCCTAGCCCGCATGACCACCTGGGCTAGC

TCAAGATCAACCCAGCGGGAGCAGGGTGGGTACAGTCTAAGGTTGTGATGCAGGTAGTTA

AGTGTGGTGGCGACGTGCTCGGCCACAAAAAAATACATGACCCAACGGCGAATGGTGAGC

TCATTAATGTTGCCCAGCATCTCCAAGCGCTGTATAACTTCATAAAAGTTAATTGCAAAA

TTAAAAAACTGTTCATTGCGGGCAGAAACTGTAAGTTCCTCTTGCAGGAGGCGGATAGCT

TCTACTATAGTGTCTCGGACTTCTTCTTCAAAGGAGCG---GCGAGGAGGGGA------G

GCAGGAGG---AA------AGGCCTCTACTTCGGGTTCAGAGAGCTCCTCA---------

--GACATCGGAGGCGG---CGGGGGAGCC----CTGCGTCTGCGTCTGCGCATTGGAAGC

CTGTCTATAAATCTCTCTATCATTTCTCCCCGTCTGCGTCTCATTTC---TTCTGTAACT

GCACGTCCATTT---TCCCTGGGCCTAAGCTCGA----AGGCTCCTCCCGATAGT-----

------------GGGGTGCAGGGCGCGGGC-------CCGGGTAGGCTCAGAGCGCTAAT

TATGCTCT-TTGTTACAGATTGACTTGGTAAAGACTGA------ATAGTCTGA--AACT-

---GTGACAGACTCGGATCAGTGAATTTCT--GCATAAAGGCGTCGAGCCACAAACAGTC

ACAAGGTAAG---CTTAGTATGGTGTGTGG------------------------------

---------------------------------------GTTTCGAGGGCTGACTAAAAA

GTTAAAAAAAGCTGTCTTAAGCTTGCGGATGGTGGTTAGAATCACTATGTCTTTGTTGCC

CGCATTCTGGATTCGCATGCGGT-CTGCCAAGCCCCATGC-ATGGTTTTG-ACACTGGGA

CAGGTTTTTG--TAGTGATCTTGCAGAAGCCCCTCTACGGGAACCTGTCT----------

------------------------------------------------------------

--G---TCACCTGCCATATTCGTAGTGCCATAGCCCCTCATGGGTCTAATGAGAGCCAGG

TCGGCTACCACGCGCTCTGCCAGAACCGCCTGCTGAACCTGAGCCAGGGTCTCTTCAAAG

TT------------CTCAAAGTCAATGAACCTATGGTAAGCCCCAGT-GTTAATGGTGTA

TGAGCAGTTGGCCATCACCGTCCAATTTAGTTT--TTGTTGCAAGGGCAGGTGTATCTCT

GTA----TATTTTAGTCTGCTGTAGGACCTGGTGTCAAAA-ACATAGTCATTACACGTTC

TAACTAGGTACTGGTA-ACCCACCAGGAGGTGGGGCGGTGGGTACAAAAAAAGCGGCCAC

CCTTCTGTGGCAGGTTCTCGAGGGCTCAAATTCATAAGCATGAGGCGATGGTATTGG-TA

TATGTATTTGGACATCCAGGCCAGCCC-TACAGAGGTGGTGGAAGCTCTGGTATATTCTC

GGACCCGGTTAAAAATGTTTCTGATAGGCCTGAACACCTCAACGGTGTATGGGGTTTGTC

CGGTTAGGCGGGCACAGTCCAGCGCGTTCTGC----------------------------

------------------------------------------------------------

------------------------------------------------------------

------------------------------------------------------------

------------------------------------------------------------

------------------------------------------------------------

------------------------------------------------------------

----------------------AAAACAAACAAGGAG-TCGATGGCA-------------

--------------------------------------------CTTTCATTCGCTCCA-

---------TTGCAG-ATGCATCCGGTTTTGCGACAAATGAAGCCCC-------------

-----------------------------TGCCCGCCT----------------------

-----------------------CTGTCGTCAAGCTAGAA------------GC------

---------------------------AGAGAGCGAGGGCTTGG-CCCGATTACAGGGGG

GAGCTGCACCAGAGG--TGCACCCGCGGGTGCAAATGAAGCAAGAGGCGGCGGAGGCATA

CATTCCCGCCAGCAATGTGTTTAGGGA---------TAATGAAGGAGAAGAAGCAGAGGG

ATTGCGACACTTAAAGTATGAGTCAGGCAGAATGTTACGTAAGGACCATCCTTCCAAGC-

GGGTGCTGGATGAGCGGGACTT--TGAAAAGAGCCCTGAAAATGGCA------TCAGCGC

GGCGGAAGCGCACTTAAAGTCTGCAGACCTAGTAACAGCCTATGAGCACACTGTGAAGGC

AGAAGTTAACTTTCAAACAACCTTTAATAACAACGTGCGAACCCTTATAGCCAGGGAAGA

GGTGGTTATTGGCCTGATGCATTTATGGGACTTTGTAGAGGCCTTTCTAGAGAACCCAGT

G-------AGCAAGGCTCTCACTGCCCAACTTTTTCTTATAGTTCAGCATTGTAG-GGAC

GAGGGGGT---ACTACGGGAAA--GTTTGCTTAATATTGCCGAACCC----GAGAGCCGG

TGGCTGGTAGACCTGTTAAACCTGTTACAAACAATCGTTGTGCAAGAGCGAAGTTTGGCA

GTTGGCGAAAAAGTTGCGGCTATAAATTACTCTGTGATTACCCTAAGTAAACACTATGCA

AGGAAAATATTTAACTCTGTGTTTGTGCCCATCGACAAGGAAGCCAAGATTAACACCTTT

TACATGCGTACTGTTGTTCAAATTTTAG------TATTAAGTGATGACCTTGGCATGTAC

CGCAATGAGCG--CATAGAGAGGGCAGTGTCTGGCGCCAGGCAG-----CGGGAGTTAAA

TGACAGGGAGCTTATGTATAGACTGCGGGAAGC----CTTGACTTCCAACGGTCTATCGG

AAA------TAGAAGGCGGTGAA---GACAGTGCTAGGGTTA--GATCTAAAGAG-----

--AATTGGGGGGCCGGAGC------GGGCGTAGGT-GCGGCAAGTGCGCGCTAT------

--CCCCACCTGCTAGATTATGA------GGAAAAGGAG---AAC-------------CCT

GACGGCT---------------------------CCGTCTCATTTC--AACAGCATGAGC

GCGGCACTCAATCCCATGAAAATGGCGGCCATGCAGAGCCAGCCTACAGTCGAC------

---GACAGTTGGGCCGCTTCTATTAGTCGCATTATGTCCCTAACAGCGGGGGACCGACAC

AAGTTCTCCTCTCAGCCATTTGCAAAT-AGGCTGGATGCTATTTTGGAGGCCGTGGTGCC

TTCTCGCAAAGACCCTACCCATGAAAAAGTGTTAACTATAGTAAATGCTTTAATAGAAAA

TGGCGCCATAAGGCGGGATGAGGGGGCGGGAGTGTATGATGCCTTACTTCACCGCGTGTC

CAAGTACAACAGTATAAATACACAAAGCAACCTAGAGAGGCTGGCGGGAGACGTAA----

--GGGAGGCGGTGGCACAGCAGGTGCGCATAGCT---GCGGGTAACCTAGGTTCCCTAAC

CGCTCTCAATAGCTTTTTAGCGCGTCTGCCTGCTAATGTGGAGCGGGGCCAGGATAATTA

CACTGGCTTTCTATCTGCCCTTAAGTTACTGGTAAGCGAGGTGCCCAA---TACAGAAGT

GTATCAGTCTGGACCCCACTACTTTTTGCAAAGCAGTCG-CA--ATGGCACCCAGACAGT

TAACCTTACGAACGCTTTTGAAAACCTAAAACCCCTTTGGGGAGTAAAAGCTCCCACTAT

GGAAA-GGCTTAGCATTTCAGCTTTG-----CTTACTCCTAATACTAGACTGTTGCTCTT

GTTAGTG-TCCCCCTTTA-CGGATAGCGTTAGTATTAGTAGGGACTCCTATTTGGGCTAC

TTGCTTACTCTGTACAGGGAAGCCTTGGGGCGCAATCATTTA---GATGAGCGGACTTTG

GAAGAGGTTACAGAGGTTAGCAAAGCCATGGGCAATGAAAATATAA---ACAACTTGCAA

GCAACCCTCAACTTTTTGCTAACCAATAGACAGAAGAAAATTCCCAAAGACTACTCCCTA

ACCCCCGAGGAAGAAAGGATAGTAAGGTTTATACAACAAGCCGT-TAGCTTGCGCATGAT

GCAGGAAAACCTAAGCC--CCACTGAGGCCTTGGACGTGACTGCGGCTAACATGGAGCCT

AGCTTTTATGCGAACAACAGAGACTTCATTAATAAGCTCATGGACTACTTTCACCGGGCA

GCGGCTATAGCTCCAGACTACTTTTTGGGCGCAGTCATGAATCCTCGCTGGCTCCCTCCA

GAGGGTTTCTTTACTGGGGTTTTTGACTTTCCCGAAAGAGA---CAAC---TACATCTGG

GATGGGCCGGATAGCTCTTTG------GATTT---AACGCGCCAAGATGC---CATGCGG

TTTTTGGAGGA---------AAAGTTTATAGACGATGACCAAAGAACGG-----------

AGTCT---------CGCAGCGTCAGCAG------AGTCCCGACTC--------CAGCAAG

CTC------TAGACGGTCATCCGTGGCCATG----------GCGTCTGATAGCCTTATTA

GACCAA-----TGAATAACGA-------CAAGAAC---------------------AGCT

TAAGAGAAATAGAAGTGCTAGCAGATAAGCT-GGCTCGATGGAAAACCTACAAGCGTGAG

TCAGAGGAGGCGCGGGA------------GTCCCTGCCTGT---GGTAGTAAGACCCAAG

---------AAATACAG------TTCCGCCATTTCCAGCGATGAAAGTGATGATGGGATG

AGCAAGCCGGACAAGTTTCTCAAGTTTGAGGGGAGTGG------------AAACCCATTT

GCCCATTTGCGTCCCAAACTGGGCCGTTGTTTGTAAA-------------GTAAATAAAG

--------------TACTCACCAGAGACATG----------ACGCTAGCCTTTTT-----

--TTATAGC----------------AATGGAG----------------------------

T--TTCCGTCGTCTCCACCCCCGTCTTATGAAACGGTGAT--------------------

----------GGCA---CAAGTGCCTTCAATCCTG-GCACCGCTGGTACCTCCGCGGTAT

AAAGGG-GCTACAGAAGGAAGAAACAGTATTCGTTATTCCCAGCTGCCGCCTTTGTTTGA

CACTACAAAGCTGTACCTTATTGACAACAAGTCTTCAGACATTCAGGCTCTCAACTACCA

AAATGACCACAGTAACTTCTTGACAACTGTAGTGCAAAATGCTAATTACACGCCCATGGA

AGCCAGCACCCAGTCTATACAGCTGGATGAGCGTTCGCGCTGGGGTGGGGACTTTAGGTC

CAT--TCTGCATATGAACATGCCCAATGTGACAGAGTATATGTTTAGTAATAGTTTTAAG

GCATATCTTCCTGCTACGGCAGACGCGTCTGGCAA-------------------------

-----------AGTGCTCACCTATGAATGGTATACATTAACCATTCCCGAGGGCAATTAT

TCTGAGGTGATGCTTTTGGACCTGCTCAATAATGCAGTAGTTGAAAACTACCTGGCACAT

GGACGTCAGCATAATGTGAAGGAGGAGGACATTGGCCTCAAGTTTGACACAAGAAACTTT

TATTTAGGATTTGACCCTGAAACTGAATTGGTCATGCCTGGTTTTTATACTAACGAGGCA

TTTCACCCTGACATAATACTGAGTCCAGGCTGTGCAGTGGATTTTACCCACAGTAGGCTT

AACAACTTTTTAGGCATTAGAAAAAGGCTGCCCTACCAGGAGGGATTCATAATTAAATGG

GAAGACCTACAGGGTGGTAATATTCCAGCCCTGTTAGACTTGGAAATTTAC---------

---AACCCCGACACTCC------------------AGGCGACAACATCAC----------

------------------ACCACTACTA--------------------CAAGA-------

------------------------------------------------------------

------------------------------------------------------------

-----------------CTCCAAGGCGAGGTCCTATCATGTGGGCGAA---GATCCCAGC

GCG---GGCAGTACCTTCACTTCATATCGCAGCTGGTTTCTGGCCTATAACTATGGACCC

GT---CGAT---GGTATCAAGAGCAAAACTGTTTTGGTGGCTCCTGACATTACTTGCGGA

GTTGAGCAAATTTACTGGAGCCTGCCAGATATGGCGGTGGATCCAGTAACCTTTACTTCT

AGTC---ATAATCCTAGCAGCTACCCAGTGGTAGGTACAGAACTGTTACCATTACTACCC

CGAAGTTTTTACAATGGATCTTCAGTGTACAGCCAGCTATTGCAAGAAAGCAC-TGCTCA

--AACACATGTTTTTAATCGCTTTCCTGAAAATGCCATTCTGAAGAGGCCTCC-TGCCCC

AACAATTATCAGCATTAGCGAGAACGTACCCGCCCTCAGTAACCATGGGACGCTGCCCTT

AAAAAACAACATTCCTGGGGTGCAGCGGGTAACCATTACAGATGCAAGAAGAAGAGTGTG

TCCCTACGTGTACAAAAGCTTGGGGGTCGTTGTACCTCGCGTGCTGTCCAGTAAAACATT

CTAGTGAC------------------AAACTTTTTCTTTTTATTAGC-------------

--AA-AGCCATGGCCATCTTAATATCACCGTCTAACA-ACACCGGCTGGGGCCT-AGGCA

C-----TCACAAGC------TATTTGGAGGAGCCAAACAAAAATCAGAT--CAGCATCCC

GTTTATGTCCAAGCTCACTACCGGGCCCCGTGGGGCG---GCAAAGG-------------

--------G--CGCCGTCG---TCCAGGT---CGAGCCCGCGGGGT--------------

CCCCCTTGATCCTAAAACTGAAGCAGAG-------GTGGTTGCCACCATCGACGAGGTGG

CACGAAATGGTCCCCCGGCAGCGCG-CCTGGTGCTGGAAGC--CGCTCGGCGAGTGGGGG

CCTACAA-----------CCTCCGTCGAGCGC----GCAAGCTAACCCCA----------

-GCTGGGCGTG-----------------------CCATGGC-AGCCATGCGCGCACGCCA

GATGGTAAATCAGGCT--------------------------------------------

--------------------------AAAAGAA---------------------------

---GGAAGCGCAGG-GTCCGCTCTA-----AATAAAGTG---------------------

------------------------------------------------TTTCCCC-----

-----------------ACGTACACACA------------------------------CC

CGAGTGATGGCCTATTTT-------------------T------TTACTG----------

-------------------------AAGCCTCATCGCCATGGC--AGCAATC--------

-----------------------AGTCGCGCCATCAAGCAGG-AACTGC--TGGAAGACC

TCAAACCTGAAATGTATCTACCACCCAAGTCCACCAG--------ACGGAGAGCTAAAGT

TAAAACAGAG-----------------GAAAAAGTTG--------ATGTGAAAACCCTAG

TCAAATCCAAGAGCAAAAAGCGCAGGGCGGCCAAGAAT----------GAGCTG--GAAG

AAAATGTAGAGTTTGTCAGACGCTTTGCTCC------CCGACGCCCCTACCAGTGGCGGG

GCCGACAGGTTCGCGCCTTGCCCAGACCCGGCATACCCGTGGTGTTTACTCCCGGGCAG-

CGGT--CAGGCGTCGCTTCCAAAAGGTCTTATGATGAAGTGTATGCAGATGAGGATGTGT

TGGATCAATCTGGGAATATGATTAATGAGTTTGCTTATGGCAAGCGAGTTAAAAT---GC

TTACCCATAAAAATCCCACTCCCTCACAAGTGCCC---ATCACTCCCCAGGAGCCAATAG

CGCG---------------TCCTGGGGAGGCGGGCCTGCTGCCCACAGTGCAAGTGCTGG

CTCCACGAGATTCAAAGCATGAAACTATGTTGCCCGTGACAA--AATCGGAAG-GAGGAG

ATGTGAAAGTGGAAAACAAGGGTTTTGAGCAGA--TCACTCCACAGTTGGGGGTGCAAAC

TGTAGATATTAAGGTACCCGTGAAGCGCAAGAGCGA-------------GGTAGAGGATG

AAATTTTAAAGAGAGCTAAGATGGA------------ACCATTTGAAACCACTGTAAAAA

TGGAGTACTCTGAACAGCCCCAGGTTGA---GGTTTTTGATACAGGGGTAGAACCTAGCT

CTTTTTTTGAAGT-GCGGTC---------------CCA--AGCGCGTCCCATTGCGGTAG

CTAGAAAGCGTCGT----------------------GTCCCTACCGTAGAGGTTATGGAG

GT--TCAGCAAAGCGAT----------CATACTGCTCCGACTGCCAGTGCCGCTCCAGTT

GCCAATGTCATAG----TAGGGCCGCATTTGAG-CCGTCGCCCTTCA-AGATGGG----G

ACCTGCCAATGCCATCTATCC----AGATTATGTATATCACCCTAGCA--TCTCTGCTAA

AAAAACCATGGGCCCTAGGCCAACAGGAAGGGTTAGCCGCTGGGGACCTGCCAACAGCAT

TTTTCCTGAGGTGCGCCTGCATCCCAGCATGGTGTCTGCA--------GTGACCAGAGCC

GCTCCG------CGCAAATCTACTAAAAGCCGAAGACGCCGGCGTGTAA---GAACCCGC

CGGGCT------TTTGTGTTGCCTGCAGGCACCAAAACTGGCGTGATGCTTCCTCAGAAT

ATACGTTACCACCCCTCCATCC------TGTTTCGCCGAGC-CTAA---------TCTG-

------TAT---GTTTT-CCTTGC------------ACAAGGATGGCCGGCAGAAATGTC

ACCCTTCGTCTGAGAGTCCCAGTGCGCACCAAAATTACAGGCGCAGGTCGTCGGCGCGG-

--ACGGCGCACGC-GC---ATTCGTTGCGGGCG----CATGAAAGGGGGCTTTTTGCCAG

CCCTCATCCCTTTGATAGCCGCTGCTATTGGCGCAGTACCAGGAATAGCTTCGGTAGCGC

TCCAAGCTGCTCGGCACTAGC-TTTT-ACAGA----------------------------

----------------------CTGACTCAC------TCCCATTT---------------

----------TTCTTGT---GCCAGAAGAAGA----------------------------

-AATGGATGCTGTCAATTTTTCCATCTTGGCACCGCGCTACGGGTCCCATCCTATGATGA

GTGCTTGGTCTGGCATCGGTACCAGCGACATGAACGGAGGGGCCTTTAACTGGGGGGGCA

TTTGGAGCGGCAT---------------CAAAAACTTTGGCAGCAATGTAAAAAATTGGG

GCAGCAGGGCTTGGAACAGCCAAACGGGCAAGCTGCTCAGGCAAAAATTAAATGACACTA

AAGTTAGAGAAAAGCTGGTGGAGGGAATCTCCACGGGTGTGCACGGGGCACTTGACATTG

CTAACCAGGAGATAGCAAAGCAAATAGAGCGGCGCCTGGAGC---GTCACGAGCCCTTAG

AGCCGGAAGTGGAGG----------------AAGAGA------CAGTTGAGACGAAATCT

GAAGCAAAG------------GCCCCGCTGGTGGTAGAAATGCCTCTAAAGCGCCCCCGC

GATGAAGAT---TTGGTCATAACCGCAGACGAGCCTCCCTCGTATGAAGAGACCATTAAA

ACAATGGCC------CCC-CTGGTGCC----CATGACCCGTCCTC-----ACCCTTCCAT

GG------------CCAGGCCTGTGATAGCAGACCGACCCACCACTTTGG-AACTAA---

-----------AGCCCTCCGAT-------CAGCCGCCTCCATACTCCCC-----------

----------------------GCAGTCA-----------------------------TC

TAATATGCCC---------GTGACCGCGCCTGTAAGGAGCAGAGGCTGGCAAGGAACTTT

GGCTAACATTGTAGGAGTGGGGCTAAGTAATGTGAAGCGCCGTAGATGCTTCTGAC---T

GTGATTAAAT-ATATTTAAGTG----TGAAGT-------TTGCCGACCCTGTCTTTTTT-

------------------TGACCGCGCCAGCTGA-------------------GAAGATG

GCAACTCCGTCGATGCTGCCACAATGGTCTTACATGCACATTGCTGGCCAGGACGCCGCC

GAATACTTGTCTCCCGCCCTGGTTCAGTTTGCCCAAGCAAC-CAGTTCTTACTTTAAGTT

GGACAACAAGTTCAGAAACCCCACTGTGGCCCCCACTCACGATGTAACCACTGAAAGGTC

TCAGCGCTTGCAGTTGCGCTTTGTGCCAGTTATGCAAGAAGATGGCCAGTACACTTACAA

AACCCGGTTCCAATTGGCAGTGGGAGATAACAGGGTTCTGGACATGGCCAGTACCTACTT

TGACATTAGGGGCACCCTAGACAGAGGCCCCTCCTTCAAGCCCTACAGTGGGACGGCTTA

CAATGCTCTCGCTCCCAGAGCTGGGGCTAATAACTGCC-------------------TAT

TTAATGGATCAGGTG---------------------------------------------

-----CCAACA------------------------TTAACACTTTAGCCCAAGTGCCATT

TGCGGGCGCCATTACCGTTA---ATGG---------------------------------

-----T--------CAAGCCGCAGTCACAGACAACACCTACCAGCCAGAGCCCCAGCTGG

GCCCTGAAAGTTGGGTGGATGGCACC---TTGGCAGACCTAGGAGATGCGTCTGGCCGCG

CCCTGAAAGCATCGACCCC---ACGCATGCCTTGCTACGGTTCTTATGCTCCCCCCACCA

ATGAAAACGGAGGTCAAGCAA------------------CTGGGGC--------------

-CGTGG----------AACGAAGA-----TTCTAT---------------AAAGTGACCG

CCAACAATAAT---AATGAAGCTGATGCCCTACTATATACAGAAGATGTGAACCTCCAAA

CCCCAGACACCCACTTGGTGCATCAGGTGTCAGACGATCAGGTTAC------AGGTGTAC

AGGGACTGGGGCAACAAGCTGCCCCAAACAGGCCAAATTACATTGGCTTTAGAGATAACT

TTATAGGTTTAATGTATTACAATAGTAATGGAAACCTAGGGGTGCTGGCGGGTCAATCGT

CTCAACTAAATGCCGTGGTGGACTTGCAAGACAGAAACACAGAGCTTTCTTATCAGCTGT

TGCTAGATGCCCTTACAGACAGGTCTCGCTACTTTTCCATGTGGAACCAGGCAGTAGATA

GCTATGACCAGGATGTCAGGATTATTGACAATCACGGCGTGGAAGACGACATGCCAAACT

ATTGCTTCCCACTGAGCGGCATGGGACCATTAACTAACATGACAGCTATGAAGGTCAAT-

-AA------TCAAAA----------------CTTTCAAACGGACAACACTAACGTGGG-T

CCCATTCAAAAGATTGGTTTCGGAAATGTTGAGGCCATGGAGATAAATCTCAATGCTAAC

CTCTTTAAAGGTTTTCTCTACTCCAATGTGGCCCTATACCTACCTGATGCCTATAAATAC

-------ACACCTGATAACATTGTAGCT--CCTGCTAATGCAAA---TACCT---ATGCT

TACATGAATGTGAGATTGCCCGCTGCTAACCTTATAGACACATTTGTAAATATTGGCGCC

AGATGGTCACCTGATGTAA-TGGACTCTGTTAATCCTTTTAACCACCACAGAAATGCAGG

ACTCCGCTACCGATCACAGCTGCTTGGCAATGGCCGCTATTGCTCGTTCCATATTCAGGT

CCCTCAAAAATTTTTTGCAATCAAAAATCTTCTCCTTCTACCGGGTACGTACACGTACGA

GTGGTCTTTCAGGAAGGATGTAAACATGATCCTTCAGAGCAGCTTGGGCAATGACCTCCG

AGTGGATGGAGCCTCTATCAACATTCAAAGCATCAACCTATATGCCAGCTTTTTCCCCAT

GGCACACAACACAGCCTCCACTTTGGAAGCCATGCTGCGCAATGATGTAAATGACCAGTC

CTTTGCAGACTACCTGTCTGCCGCCAACATGCTTTATCCGATCCCTGCCAACACTACAAA

CCTACCAATCTCCATTCCTGCCAGAAATTGGGCCGGATTCAGAGGGTGGAGCTTTACCAG

AATTAAGCAGCGGGAAACTCCAGCCCTGGGCTCACCTTACGACCCCTACTTTACTTACTC

GGGTAGCATTCCCTACCTGGATTCAACTTTCTATCTTAGCCACACCTTCAGAAGAGTCTC

CATCATGTTTGACTCTTCTGTATCTTGGCCGGGCAATGACAGGCTCCTCACTCCAAATGA

GTTTGAGATTAAAAGGTATGTG------GACGGTGAAGGCTACAACGTGGCCCAGTCCAA

CATGACAAAAGATTGGTTTCTGGTTCAAATGCTGGCTCATTACAACATTGGCTATCAAGG

CTACCACTTGCCCGAGAGCTACAAAGACAGAATGTACTCATTCCTCAGAAATTTTGAGCC

CATGTGCAGACAATTGGTAGATGTAAC----TAACT---ATGCTACCTACCAG--TCAGT

CACCGTAGGTCACCAG-----------------------CAT-------AACAATTCTGG

ATATGCTAGCCCCCTTTC---AACCTTTAACCCAAGGGAG----GGTCACCCCTATCCGG

CAAACTGGCCTTATCCCCTAATCGGGGTCAATGCTGTG---------CCTACTGTTACCC

AAAAA--AAGTTCCTTTGTGACAGAACCCTATGGCGCATCCCCTTCTCTTCCAACTTTAT

GTCTATGGGCACCCTCACTGACCTTGGTCAAAACCTGCTGTACTCCAACTCCGCTCACG-

CCCTTGACATGACTTTCGAGGTTGATGCCATGAATGAGCCCACTCTGTTGTACGTTTTGT

TTGAAGTGTTCGACGTGGCACGTGTTCATCAACCCCACCGAGGGGTGATTGAAGTAGTGT

ACCTCAGAACTCCCTTCTCCGCCGGCAACGCCACGACCTAAACTAAT-------------

------------------------------------------------------------

---------------------------------------------ATGGCGGAAGGAGG-

-------------------------------------------GTCATCAGAAGAAGAGC

TGCGAGCCATAGTACGTGATTTAGCCGTGACCCCATTTTTCCTGGGCACCTTTGACAAGA

GGTTCCCAGGCTTCATCT-CCTCTCAAAGAATAACTTGTGCCGTAGTAAACACCGCGGGT

CGAGAGACTGGAGGCGTGCACTGGCTGGCCATGGCTTGGAACCCCCGCTCCAAAACATTT

TATATGTTTGATCCATTTGGATTTTCAGACAGCAAGCTGAAGCAAGTGTACAGCTTTGAA

TATGAGGGCCTTCTGAGGCGCAGTGCCATAGCTTCCACCCCAGACAGGTGTGTTACCTTA

GCAAAAAGTAATGAAACAATTCAGGGACCCAATAGCGCCGCCTGTGGACTGTTTTGTTGT

ATGTTTTTACATGCTTTTGTCAACTGGCCAGACAACCCCTTCAACCACAACCCTACCATG

GGACCCCTTAAAAGCGTGCCCAACTATAAACTATATGATCCCAC-AGTGCAGC-ATGTAC

TTTGGGAAAACCAGGAAAAATTGTACAAGTTTTTAGAAAAAAACTCTGCTTATTTTCGCG

CCCACGCCGCCGCAATCAAAACCAGGACTGCTTTTAATAAGTTGAAA-------------

----CAATAAA-----CCG-------TTTATTCGAAAAAT-AGAAATGGTT---ACAGTG

TGAT-TATT------------------------------------AAAAGAGGACAAGGT

CATCGTCATGC-----------TGGCCCTGG----------------GGCAAAATGGTAG

TTTGATATCTATG-----CTCATCAGACCAATGAAACTCCGGGAATTTAACGGGGGGC--

----CTTTCGCCCACTGTGGACA---ACCAT-----ATCTGCTTAGCAATTTGAAGACAC

GACACCAGGTCCACACTGGAAATTTTAAAATCACAATTTTTCTGGGGAGCGGCCTTGC--

-----TGTTGCGGTAGAC------TGGA--TTGCAACATTGGAACACTAGCATAGCGGGG

TTATTAAGGGTTGC---TAACATCTTCTGATCGTCCACTTCGCTTTTATCGATATGGCTC

GCAC-TTGCG-----AGGGAAAACGGG---GTTATCTTAC----------AGGTCT---G

CCTGCCCAGT---------------------AGAGGCAAACTGCTG---TTCCAATTG--

CACTCA-----CACTTAAGGGGCAT-CAGTAAATGACTCTCA------GCGTTTGGCATA

GAAGGATAGCAAGCTTTAAGAAAA-------GCCATGATCTGCTGAAAAGCCATAATGGC

CTTGGGGCC----ATCCGTATAAAACATACCGCAGGAGGCCCCGCT-------AAAGTTA

CCACCACTCATATT---------AACATCCATGCTGCAGCAAAAAGCATCCTCATTC---

-TTGAATTGCA--CTACATTTCTG--------CCCCACCGGTTAGAAACAAT----TTTT

GTTTTTTCTGGGTTTTCCT----TGAGAGCTCTTTGAGCA-----TTTTCACTAT-----

----TAACATCCATCTCTACTAACTGCTCTTTTTGAAT--------------CATTGGTG

AACCATGGAGACACATTAAGCTACTTT-------------------G--ACATCCATGTT

----------GCCAAA----CCACACAT-----CCACTTGGGTTA---A-----------

-CCCCAGGGGCTAGCTCCGCCGCCTTGATAACAAAATCAAGTAGCAGACGTCCAGCCACA

TGC----AGAAAA----------------------GTTTTTTGGTTGGTAAAAGTGTAGG

TAACGT---------GCATCTTAGAGGTGGTAATGTAAGCC----TGGACTGCTTTTTTA

AAGCATTCCAGCGTGCCCTCGTC------TGGTAGCAAAGT-------------------

-A--------AGTTTGCTATGGTCTACCT----GGTATGCA--TTAAGAACCTTCAGGGC

AAACTCCAT---AGCAGACTGCCATTTGT-------------------------------

--------------------GCTCA---CTAGCTTTAAAAATCTTAGAGGCC--------

-------------------------------------ACTGAAGCC-------AAAGGCG

AGCCAGACTTGTTACAAATGGATT----TCTGCTTGGCTGGGGTAAAAATCACATTTCCC

TCTTTGTCAACGTCCACC-GTTTGGTCACAAAATGTCTGCAC-TATAATGCTCCCGGGAT

CTCCCC-------G--CGCCTTGAGAGCGGCAGTAGCTTG-----------GCG-GGCTA

TCTCAACCATGGCTT---------------------------------------------

----------------------GGTGGCCCCTT---------GGCTCCTC----------

-AACG-GCTTTCCGCTGGCGCTTCTTTGGGG---------------GCGCCG--------

------------------------------------TAGGCGGTG---------------

--------------CCACAGGAACCTCTTCAT---------C---GCTGG--AGCTTTCA

GAGATTG------------------CAACAACCTTCTTGTGGCT----------------

-----CATCTTTT---------------T-CCTAGATGTCA----GAAGAGCCCGTCAGT

GGGACTACC--------------------------------------------------G

TGGAGATA---GAGGAG----------------------------GACACTCACAC----

---------GCCTCCAA-------------------------------------ACTCTC

CTGTCCTCG---------------------------------------------------

-------AAACA------------------------------------------------

T--------------TCTCTCTAAGCCCCGAACCAGAGGCT-------------------

-----------------------------------------------GAGGCCTG-----

-------------T---CCAAA---TAC--------------------------------

------------------------------------------------------------

--TGAC--------------------------------------------AGATACCTAA

GCGCCAATTTAC------TTTGCAAGCACCTGCAACGTCAAAGCGCAATTGTCCTCGATA

GTATCAAAGACCAGCTTCA-AGTGCCAACC-AGTGTATCAGAACTAAGCTGTGCCTAT--

GAACGAAGCTTGTTATGTC-------CAAACATCCCACCAAAG-----CAACAAAGCAAT

GGAACATGTGAAGCAAATCCCAAGCTTAACTTTTACCCAACTTTTTTGGTGCCCGAGACA

CTGGCAACTTATCACATTTTTTTTGTTAATCAA-AAAATACCAGTATCTTGCAAGGCTAA

CAGAGCTAAAGCAGACAAAGCCCTTACTCTGCAAGAGGGAGATTGCTTACCTGACTATGA

AACAATGGACACTGTGAGCCGGGTATTTGAAGGCTTAGGCGGAGAGGTGGT---CGCGGA

AAACG---CGCTGCA------AAACAATGAC---------------TCTGTATTAGTAGA

ACTTAAGGAAGACAATCCCCGACTAGCTGTACTTAAAAGAAACCTCA---GCGTTTCACA

CTTTGCCTATCCGGCCGTACACCTACCACCTAAAATTATAACCACTGTCATGAACAATCT

A---------CTAGTAAAAAGAGCAAACCCAAGCGC---------CGACGTGTCAGAGTT

AGA---TCCCGATGGGGGTCAAGAGGTAGTGTCGGACACTGAACTAAGCAGGTGGCTTAA

----TACTTCTGAC-----CCAGAAACCC------------TGGAAAAGCAGCGAAAACT

AGTAATGGGAAGCGTATTGGTTACCGTAGTGCTTGAGTGCATGCAAAGGCTTTTCACTTC

AAAAGACATGGTAAAAAAAATCGGAGAGACCCTCCACTACACCTTTAGGCATGGGTACGT

GTCCTTAGCTTGCAAAATCTCTAATGTGGAGCTAACCAATGTGGTCACCTATATGGGGAT

TTTGCATGAAAACCGCCTGGGTCAAAC-CACCCTGCATCATACGATACAAGGTGAGACCC

GCCGAGATTACATTCGTGACTCCATTTTCCTCATACTTATCCATACCTGGCAAACCGCTA

TGGGCATTTGGCAACAGTGCCTAGAGGAGGAAAACCTAAAAGAGCTGGCCAAGCTAGTGC

AAAAAATTAAAAAGCCACTGTACACTGAGACCTCCCAGCGCCTTATGGGCAAG-CAGCTG

GCAAACGTAGTC-TTCCC--ACCCAAACTGCTAGAAACCTTTAATAAGGGCTTGCCC-GA

TATTGTCAACCAGAGCATGATGCAAAACTTTAGATCTTTTATTTTAGAAAGGTCCGGAAT

TCTACCATCCATGACATGTGCTCTACCGACAGATTTTATTCCCATTCACTTCAAAGAGTG

TCCCCCTACTATGTGGCCTTACACTTACTTATTAAGATTAGCAAACTTCTTTATGTATCA

CAATGACCTATGC------TACGACATGGAAGGCGAGG------GC---CTACTAGAGCA

CTA---CTGTCGTTGCAACCTCTGCACACCACACCGTTGCCTAGCCACAAACCCCGCAAT

GCTTAATGAAACTCAGCTAATAGGTACCTTTGATATCCGGGGTCCCGGCGGGGAAA--AC

GGAGCAGAGTCTTCCTC-TGGCCTTAAGCTCACCGCTGGAATGTGGACTTCCGCGTTTCT

GAGAAAATTTGAAAGTTCTGATTATCACGCCCATAAAATTCACTTTTATGAAAACC---A

ATCAAAGCCCCCTTCAGTTGAACCCACTCCCTGCGTCATTACCCAAAGCAGTATTTTAGC

CCAATTACATGACATCAAAAAAGCTAGGGAGGAGTTTTTACTCAAAAAAGGGCAGGGACA

GTATCTAGACCCTCATACTGGGGAACCGCTCAATGC------------------------

--------------------------------------------------CGCGGGTCCT

TCCGTAGAGAGTG--------GCCATGAGT------------------TCCAAGGAGATG

GAAGAC---ACAGAGAGC-----------CTAAGCGTGGAAGACATTTCCGG--------

--------------------------------CAGCGAGG---------------AGGAC

CTAGAAAGCCT------------CCCAG---AGCCCATGCCGGAGGAGAGCCAGATG---

---------------TCAGAGGAA------------------------------------

------------------------------------------------------------

CGACCT---------------------------------------------CCTAGATGG

GACCAGAAAAAGAAACTTAAGGG-------TAAG---CGGCCCC----------------

------------GCAACTATCAATCCTGGCGAGCACACAAGTTTAAGATCCTTAGCTGCT

TGGGCGTCAGTGGGAACAGTGTCGCCTTTACCAGAAGATACATGCTTTTTCGAGAGGGGG

TTAACCTCCCCAACAA-CATCATTCACTACTATAA--CTCTCGCTACCGCAGCAGG----

--ACAGAAACTC----------------------------AAGCGCA-------AACCAG

TCCCACTGAAACGCCCA--AAGCCAGC---------------------------------

-CGCCGGTG----------------AGGACAACCAGCAGACAAGGCA-GCTGCGCAATCG

CATTTTTCCAACACTGTATGCCATCTTTCAGCAAAGCAGAGGCTGCCCCACGGCA-----

----TTAAAAATAAAAAACAGATCCCTCAGATCCCTTCTGAGAA-GCTGTCTGTACCACA

AATCAGAAGAACAA-CTCGTACGCACGCGAAACGACGCCGAGGCTTTGCTCAACAAATAC

TGT-----------CAAGGACTCGAGTCCAAC----------------------ACAGAC

TGA-----------------------------------------------GCAATGTCCA

AAGAAA-TACCAACCCCTTATATATGGAGCTACCAACCGCAAACAGGACATGCTGCAGGC

GCCTCCCAGGATTA---CTCCACCCAAATGAATTGGTTTAGCGCCGGCCCATCGATGATT

AGTCATGTTTACGGAATTAGAGACTTACGCAATAAAGTTTTGATGACCCAGGCACAAATA

ACAAAAACTCCCAGAACAATAATGGACCCTCCAATTTGGCCTGCT--TCCATGCTTGTAC

AAAAGCATGCCACACCCAAA-ACAATCGCTCTGCCCAGAAACCACACCCTAGAACAGGCT

ATGGTGAACTGTGGAG--CACAGCTGGCGGGAGGACGACA-------------------G

CCAAGTCCCTCC-CACATAGATATAAAAGACACAA--------TGCTTGCCGGTACGGGC

ATTCAGCTGGGCGA---GGACATCCCAAGCG-TCTCCTGGAT---------AAGGCCCGA

CGGCATCTTCCAGC--TAGGAGGGGGATCCCGTTCATCTTTCAGCCCAACGCAAG---CA

TTCCTCACTCTCCAGCAGGCATCCTCAACACCGCGCACAGGAGGCGTAGGCAGTTACCAG

TTTGTA-CGTGAATTCGTGCCAGAGGTATATCTTA-ACCCTTTTTCAGGACCCCCGGACA

CCTTTCCTGACC---AATTCA------TCCCAAACTACGACATTGTAACCAACTCTGTTG

ATGGCTATGACTGAGGAAAG---C--------GTGGACCAGGTGGAAGT-GAACTGCCTA

TGCGTCCAGCATGGCCAAAGCTGCAATAATACCCGC-TGCTTTGTAAAGGAGGGTTTACG

-CGCTAACTGGTTTTACA-------ACCCAGTACTTG------AAGAGTTTGCT-ATTCC

AGACTCTTACCAAGAGGG-----ACATGGTGTGAATGTTAAAATAAC-----GTTTTCCC

ACCGCTCCAGAAA-----------------------------------------------

------------------------------------------------------------

------------------------------------------------------------

------------------------------------------------------------

------------------------------------------------------------

------------------------------------------------------------

-------------------------------------------CACAGGCCAAATTCCGC

TCTGT---------------CTCTCCAATGGCACATGCCACATAAGCGAAAAAGGCCTGC

ACTTTTCTGCAAATTTTTCCAAGGATGGCCTA------------------TACATCGCCA

TCATT--------------------AATGAAACAAACTATCATGCCGCTGAACATTAC-T

A---------CCTTGTCTATATT-----TATGAGAACTGCC-------------------

---------ACCAGATGCCGTA-TGATTC-------------------------------

------------------------------------------------------------

---------------------------------CCCACGGCACACC--------------

-------------------GGCCACAA-CGG-----------------------------

------------------------------GACCT-CATTC-AACTGGT-----CCATGG

-------------------------------------------------------GACTA

TGGCTGGTA---------------------------------------------------

---------------------AAAT------GCAGCCATA--------------------

------------------------------------------------------------

------------------------------------------------------------

----------------------------------------------------ACAAAACT

TTCTTTCT---------------------------------------GCCATTTGTTCTA

GACTCTGCAAA-----------------AAGTGCTCCCATTATTATGAC-----------

--------------------TGAGACTGCTATAACTATATACATTTCC------------

ATGATA---TTTTTAATTGTAAGCCTGCTAACCTTTCT-AAATGTTTTAAT------AAC

GCTAAACAA-----------------TAAATATAAACACTATGGAGTTTAA-AAATAAA-

--------CTTACCTAATTT--TTGTCAA-----GACTTCTGGGTCCTGTGTCTCTATGT

CCACAAGGGCCCCCTCTT--------------------------CCCAACTTTGAT---A

CTTCCACTTGTGTGTGCGGGCCAACTTGCGCAAATGC--TTAAAAGACAATGTGGTCTCT

CCCG-----------ACAGCTTCCCGTTCACCAACACCAAAGCCA--TGAAGCGG-ACAC

GAAGGTCTCTACCTGCAAATTTTGACCCCGTGTATCCATATGAC----------GCCCCA

AAACCCTCCAC----------TCAACCCCCTTTTTTTAA-------TGATAGAAAAGGTC

TCACTGAATCATCCCCCG-GAACTCTGGCTGTGAA----------TATCTCGCCTCCACT

AACCTTCTCTAACCTAG-----------------------GCGCGATCAAGCTTTCCACG

GGTGCCGGACTCATCC--TCAAAGAGGGAAAATTAGAAGCCAACATAGGAC-CGGGTCTT

ACCACA-AATCAAGAAGGACAAATCAC-----------TGTTGAAAAAGACAGTGACGGC

C---TAACATTCACTTCCCCCCTACA----------CAAGATTGAA--------------

-AACACCGTATCTC---TAAGCATAGGCGAAGGGTTAGAA--------GATGAAAGTGGC

ACACTCAAAGTGAAT-----------TTCCCTAGTCCCC-CACCCCCTCTACTATTTTCC

CCTCCA------CTTGCAG-AGGCGGGGGGTACTGTTTCA---CTACCCTTGCAGGAGTC

CATGCAAGTAACTGAAGGAAAG---------------------CTCGGCGTAAAGCCTAC

CACCTACTC----------------TCCGCC--CCTTCAAAAAACTGACCAGCAAGTAAG

CCTGCGCGTAGGTCCGGGTCTCACCGTACT--A---AACGGACAATTGCAAGC-------

--------AGTTCAA------------CCTCCAGCAA---CAACCTACAAGGAGCCTCTC

TTAGAAACTGAG--AACTCTG------------------TTTCTCTTAAAGTAGGTGCCG

GCCTTGCCGTGCAGGATGGAGCCCTAGTGGCAACCCCT--CC------------------

-------------------AAACGTCACCTTTTCCGCA---CCCCTAG---AAAAAAATG

GAAATGCAG-------TGTCAGTGCGCGTAGGCGCGGGCTTAT----CTATACAGGGT--

-AATGCCTTAG-------------------------------------------------

----------------------TGGCCACAACCTCCCCAACCTTGACCTTCGCCTATCCA

TTGATTAAGAATAACAACCATATAACTCTAAGTGCTGGG-----AGTGG--CTT------

---------------------------------------AAGAGTATCCGGAGG------

-------------TA---------GCCTTACTGTGGCCACTG-GACCTGGACTTTCCCAT

ATAAA-----TGGAACAATAGCTGCTGTAATAGGTGCAGGTTTAAA-----------ATT

CGAAAACAATGCCA--TTCTTGCAAAATTAGGCAATGGTCTAACCATCA--------GGG

ATGGCG----CCATTGAAGC-----AGTGGCACC-------ACAACCCTCAT--------

-----TTACCCCTG---------------------TAACTCTATGGACTGGACCTGATCC

AAACGTTAACGCCTCAATT---AATGGCACTCCGGTAATTAGATCATTTATATCATTAAC

TAGAGACAGTAACTTAGT-CACAGTCAATGCTA-----GCTTTACTGGAGAAGGAAGCTA

TCAATCAGTGAGCCCTACCCAATCTCAATTCAGCTTAATTCTAGAATTTAACCAGTTTGG

ACAGCTAAT---------------GTCCACAGGGAACCTTAATTCCACTACCACTTGGGG

AGAAAAACCCTGGGGCAATAAC--ACTGTACAGGTACAACCAAGTCACACCTGGAAACTA

TGCATGCCTAACAGAGAAGTGTA--------CTCCA---CCCCCGCCGCTACCCTTACCA

GTTGT------GGACTAAACAGTATTGCACATGACGGTGCCCCCAACAGAAGTATTGACT

GCATGCTAATTATAAACAAACTACGTGGCG---C---TGCAACTT---ACACTCTCACAT

TTAGATTTTTAAATTTTAACAAACTAAGCT---CAAGTACCGTGTTTAAGACTG--ATGT

ATTAACCTTCACCTATGTGGGGGAAAATCAATGAATACAGTTTGCCCAATGTAAACGATA

AGC--A-------ATAAAGGTCATAAACAGATGAACTTTTTAAG---ATTTTATTT----

-------TTCATACACTCGA----------GCTGTAATGCTACCGCCTTCAGGAAAAGTC

ACTCGGTA-CACGGTTCTTTCACAAGAGCACAAGACATA-AGTATTAGTTAACAA-----

-----TTGATTTGAGGGATAATAGTACACAT--TTTCTTGAGTGGCAAAACGAGGATCAG

TAATGTCAACAAAACC---ATCAAC-------------------TGGAA-TGCAAGAAT-

-----AGTCAAGCACGGTGG---GTTCA-ATCTAAAAATGAAGAAACGCGTTGAGGTTCA

CTAAACACAGATTTTGAATGTGTCGA-----CACCGTCCATACATCATTGCTTGTTTCAA

AGCAGTTTGTC-TTCTCTCCTCTGCCT-TGGAAGTGGT-CCGATGTAGC--ACTACAGG-

TATCTTT------TCAACCTCTCTCAGCACCCGATCTATTATAGATCTTACCC--AAAAA

GCGCAGCTTTTAAGAGCACAGTAGTTTTGGAGGCTGCAAGATTTACACTTAAGCAC----

------------CAGCCAGTAATTATAAGTACTTTTAAAAACCACCCCTAGCTCGGGGCT

AATGCATCTTTGAATGGCAAGCAT-GCAAGCCTGATGCACGGT-GCTAAAAAAAGACAGT

CTAAAGTAAATGTAATGAATGTTTCTAAACATTATACTCCCCACATA-GTTAATCTCATC

AGGTCTGCTAGAATTCACAAACTCTCGGTACCAAAGATACTTTTTGTTCACAAACCC---

-------------ACCCATTACAAATTCTTCAAGAAATTCTTTTAAAACTTGCGTACGAG

ACAGACATTGCAAAGACAAACTGTCAG--AACAGTGACAATGTACTTGCCACATTTGGG-

-CATCTGCTAGACAGCAGTGGCTATTTAAAATTAACTCAGCACCCCCATTACATGTAAAG

CCCCCAAAAA--GCAGTTTTTCATGTCGATACAGCAGGTCATCCCATAAAATGGGTATTT

CCAAACACAAAGCAAAGCAATTACAAAAAATTGGAGCTCTCA------CCACAGCTGTCA

CATGGGAGGTTGT---CTCAGCGTTGCAGCTGCCTTCCAT----------CCTACAA---

-TTATGAAACATAACTAAACATTTCTAACAATGATACAGAAA----CAATGTTCCGTCCT

AAAAAGGCATTGTTGACATTAGAGTGATTATTAACTGCCTCAGAAATTGC---TTGCGCC

AAAGAACGTTTGAAAT-ATGTTA-CTGCATCAACGTGCTCAGAAACAAGTTGAGAG---T

GACAAACCAC---TTTAAAGGTTAAGCCA-GGCTTGTTTTTATCAATAGCCTTGACCAAA

CTCTCAGAAAACATTTCAGCTGTGACATCATCAGTAGTGTTGAACAGAAAATCCGCAGG-

-AGATTCCAGCAT----TGTTCCAAGCTGTCTAACAAAATCTTCCTCATAATGAACTGTC

CCCTTTAGAGA---AACACGTGGCAGAC--GATGGTGG-GCCATCGCG---T-TAACCTG

AAATACATT--TCACA---GTGAACAAACCTAACTCCGCCGCGGTAAAGTTGTGTCCATG

ACTAGAACAAAAATCTTTAAAAAAGCTATCTAAGTACTTAAT----CATTCCATCAGTTA

CAATTTTCAGCAGCTGCGTGGTGCATGCCACATAGT------GCCAGCTAATA--GCTAC

AAAAAACTCCATTCCCTCC----TTG--GCAGACAGCTCTTGC---------ACACAAGC

AGTGACTATCCAGCGCTTAAGAAACGTTTCAAGCCCAGCGCAAACAAGAGCTCCAATGTC

TTTATCCAAGGAAA---GCAATAATGAAGAAAGCGCGGGCTCGATTGTAATATTGAAGC-

---------------------------ATAGACATTTCAGATG---CTCGCTAGCTG---

CAG------------------------------------------------TCGCCATT-

--------------------------------------------------------ATGA

GGT--------CTGTAATGTAAAAACAA--------------------------------

-------------------------TTCATCAGCGGCTGAAAAAATGAACCTTAACCTCA

CTAAGCCACTGCATGT---------------GCAAGTCATCATCAATGCCAGAGCCCAAA

CCCTCAATCCAACCC-CGAATAT-ACACTCGAATTTGAGACTCCACCTCCTTCTTCAAAT

AAGAAGACATAATGTTTGGACAACTGTAAACAATTGCTGTTACAATAGTGGGCCCACTGT

GACTAGAAAGCCTTCGCAC-AGCCCTGAGATTATAAGTAGACTGCAACAACATTGAAACC

CCGCGACACAAATCTGTCTCGCGGTCT-----------TGCTCTCTTATTAAATTGACCA

AGTGGTCCTTCAAAGTTATGTTGCACTCATAGAAG----TACACACCTCCGGCATC-CAT

TCTG-AAAAATAACAAAACACCACTAA-GCATAGCA---------CCGTTAC-CCAA---

AGGAAACTAA-ATTAA--CGAAGGCA--ACACT-TACCT-ATTCTGTT--GTCACCAAAA

GATTGGGTTCC---------CAAGTCACAGACAATCC--AAGAGCAAGGTGGGCGCAGTC

TTCTGGAATAAGCTGTCCAAAGTCGTGCCGGAAAGCTTCCTGAAGATCAGCAGCAGTAGC

CTGAAGATCTGCAACGGAGCCAAAACCTTCAGTGTCACTTC-CAATAAAAATATAAAACC

CTAAGTAGTCCCCATCTAAAGC-AGAAACAGCGGCAG------CAAATGTAGGCCATGGC

CTCAGTTCCTGAGCCC-TAATAGTCCTAAACAGAACATGACGCAGTTCAGTAGGCAAAAA

GTCTGTG-AGCTCCAGATGAGCACACACACT--CTCCACCAGGCTCTTATGAACTCGAAG

TAAAAAACATGCTCCCATTGACACTCCTAAGGCTGCCATA--GTACTCACGGACACC--G

CTGCTGT-------------CGAGAATGAAGTCT---CAAA-----------AGAACT-G

TCAA---AGTCTCCT--GCCATGCGCTTAAATACTGCCCTA------ACCAGGG------

---------CGTCTCAGGAA---ATCAGCCCAAGGGCGGACATTGACC-CG-----GGCA

TCCTGTCCTAATATCAACAAACACATCCTTTT-----TTAA-----------CC------

ACACCCAAAAA-------------------CCGGGCCAA------AGTCCACAAA-----

--A-----ACACCCAA----TTGA-CGTCATGGTCTTT--CCC-----------------

-----GTGGGAAAC-----GCCCTG--AACAG---GGTGTGACCTTGTCA--------AA

ACA-ATCAAACA---AAAACTTTTTAAAAAAAAACAACATTTAGGCCACACCTC------

TTTGT---CCTGTA--TAAAAAAAACAACATTTAGGCCACACCTCTTTGTCCTGTATATT

ATTGATGATG--------------------------------------------------

------------------------------------------------------------

------------------------------------------------------------

------------------------------------------------------------

------------------------------------------------------------

------------------------------------------------------------

------------------------------------------------------------

------------------------------------------------------------

------------------------------------------------------------

------------------------------------------------------------

------------------------------------------------------------

------------------------------------------------------------

------------------------------------------------------------

------------------------------------------------------------

------------------------------------------------------------

------------------------------------------------------------

------------------------------------------------------------

------------------------------------------------------------

------------------------------------------------------------

------------------------------------------------------------

------------------------------------------------------------

------------------------------------------------------------

------------------------------------------------------------

------------------------------------------------------------

------------------------------------------------------------

------------------------------------------------------------

------------------------------------------------------------

------------------------------------------------------------

------------------------------------------------------------

------------------------------------------------------------

------------------------------------------------------------

------------------------------------------------------------

------------------------------------------------------------

------------------------------------------------------------

------------------------------------------------------------

------------------------------------------------------------

------------------------------------------------------------

------------------------------------------------------------

------------------------------------------------------------

------------------------------------------------------------

------------------------------------------------------------

------------------------------------------------------------

------------------------------------------------------------

------------------------------------------------------------

------------------------------------------------------------

------------------------------------------------------------

------------------------------------------------------------

------------------------------------------------------------

------------------------------------------------------------

------------------------------------------------------------

------------------------------------------------------------

------------------------------------------------------------

------------------------------------------------------------

------------------------------------------------------------

------------------------------------------------------------

------------------------------------------------------------

------------------------------------------------------------

------------------------------------------------------------

------------------------------------------------------------

------------------------------------------------------------

------------------------------------------------------------

------------------------------------------------------------

------------------------------------------------------------

------------------------------------------------------------

------------------------------------------------------------

------------------------------------------------------------

------------------------------------------------------------

------------------------------------------------------------

------------------------------------------------------------

------------------------------------------------------------

------------------------------------------------------------

------------------------------------------------------------

------------------------------------------------------------

------------------------------------------------------------

------------------------------------------------------------

------------------------------------------------------------

------------------------------------------------------------

------------------------------------------------------------

------------------------------------------------------------

------------------------------------------------------------

------------------------------------------------------------

------------------------------------------------------------

------------------------------------------------------------

------------------------------------------------------------

------------------------------------------------------------

------------------------------------------------------------

------------------------------------------------------------

------------------------------------------------------------

------------------------------------------------------------

------------------------------------------------------------

------------------------------------------------------------

------------------------------------------------------------

------------------------------------------------------------

------------------------------------------------------------

------------------------------------------------------------

------------------------------------------------------------

------------------------------------------------------------

------------------------------------------------------------

------------------------------------------------------------

------------------------------------------------------------

------------------------------------------------------------

------------------------------------------------------------

------------------------------------------------------------

------------------------------------------------------------

------------------------------------------------------------

------------------------------------------------------------

------------------------------------------------------------

------------------------------------------------------------

------------------------------------------------------------

------------------------------------------------------------

------------------------------------------------------------

------------------------------------------------------------

------------------------------------------------------------

------------------------------------------------------------

------------------------------------------------------------

------------------------------------------------------------

------------------------------------------------------------

------------------------------------------------------------

------------------------------------------------------------

------------------------------------------------------------

------------------------------------------------------------

------------------------------------------------------------

------------------------------------------------------------

------------------------------------------------------------

------------------------------------------------------------

------------------------------------------------------------

------------------------------------------------------------

------------------------------------------------------------

-----------------------------------------------------

>CAdV2

------------------------------------------------------------

------------------------------------------------------------

------------------------------------------------------------

------------------------------------------------------------

------------------------------------------------------------

------------------------------------------------------------

------------------------------------------------------------

------------------------------------------------------------

------------------------------------------------------------

------------------------------------------------------------

------------------------------------------------------------

------------------------------------------------------------

------------------------------------------------------------

------------------------------------------------------------

------------------------------------------------------------

------------------------------------------------------------

------------------------------------------------------------

---------------------------------------------------CATCATCAA

TAATATACAGGACAAAGAGGTGTGGCTTAAATTTGGGTG---TTGCAAGGGGCGGGGTCA

TGGGACGGTC----AGGTTCAGGTC-------ACGCCCTGGTCAGGGTGTTCCCACGGGA

--ATGTCCAGT----GACGT-----CAAAG-GCGTGGTT-TTACGACAG---GGCGAGTT

C------CGCGGACTTTT---GGCCGG-----------CGCCCCGGGTTTTTGGGCGTTT

ATTGATTTTGCGGTTTAGCGGGTGGTGCTTTTACCACTGTTTGCGGAAGATTTAGTTGTT

TATGGAG--CTGGTTTTGGTGCCAGTTCC-TCCACGCCTAATGTCAAAGTTTATGTCAAT

ATAAC-AGAAACAC-----TCTGTTCTCTGTT-TACAGCACCCCACCCGGTGGTTTTTCG

CCACGCCTTTGGGT--TAATTTTATTTCCCTATACGCGGCCTTAAATTCTCAGTGC-AGA

CGAAAGAGGACT-----ACTCTTGAGTGCGCAGCGA-GAAGAGTTTTCTCTTCGCTGTGT

CTCATATAT-TTTCTGAAAA----ATGAAATATACTATTGTGCCGGCGCCGCGCA---AT

CTCCATGATTATGTTTTAGAGCTACTGGAAGAGTGGCAGCCGGACTGC------------

CTTGACTGTGAGTATTCTCATGGC---AGCCCCTCGCC---GC-CTACTCTGCACGATCT

TTTTGATGTTGAGCTGGAGA-------------------------------------CTT

CTCACAGCCCTTTTGTGGGCCTGTGTGA------TTCCTGTGCGGAGGC-----------

TGACACTGATTCGAGTGCGAGCACTGAGGCTGATTCTGGGTTTAGTC----CTTTATCCA

CTCCGCCGGTTTCACCTATTCCACCGCATCCCACCTCTCCTGC---TAGCATTTCTGACG

ACATGTTGCTGTGCTTAGAGGAAATGCCCAC-------CTTTGATGACGAGGACGAGGTT

CGAAGCGCGGCGACCACCTTTGAGCGGTG--GGAAAACACTTTTGACCCCCATGTGGGTC

CTATTTTTGGCTGTTTGCGCT-------GTGCTTTTTATCAAGAGCAGGATGATAATGCA

------CTTTGTGGGCTTTGCTATCTAAAGGCCCTTGCCGA----AGGTAAGTTTTAA--

TTTAAATGTTTGGGCAGGTTAAA------------TGTTTGGGCAGGTTAAATGTTTTAG

-------GTGTGTA----------TTGATTTTTAATTTTGCTTT---TTAGTGCCTTTTG

CTA----------------TGCCTGTACGTTCAGAACCCGCTTCGGCTGGA------GCT

GAGGAGGAAGATGATGAAGTTAT-----------TTTTGTGTCTGCCAAACCTGGGGGCA

GAAAGAGGT------CAGCAGCTACTCCCTGT---------------GAGCCAGATGGGG

TCAGCAAACGCCCTTGCGTGCCAG-----------AGCCTGAGCAAACAGAACCTTTGGA

TTTGT----------------CTTTGAAGCCACGCCCGAACTAATCT-----CCTTGAGC

ACAAAGCAATAAAGTAATCTTGT-------------------------------------

---------------TTAACAAGTTTGCCTACATTTGTGGTTT-TA--CGGGGCGGGGCG

AGGAGTATATAA--------------TGCCAAAAGCCAGTGCCTGCTTCATTAAGCTTTT

AGACTGAGC--------TAAGAGCAGGTAG-TATGGACCCTCTT-AAGATTTGTGAAAAC

TA-CCTTACTTTTAGAGCTAT-AATTAGGGGAAGTACTTTGTCGCCTGGATTTTTTAGGC

GGTGGTGTTTT---CCTGC----CTTGGCTGATGTGGTGGGCAATATAGTGGAACAGGAG

GAAGGCAGGTTTTGGCAAATTTTACCTGAAAACCACGCTTTTTGGGGTCTTTTGCGCAGG

GGCTTTACTGTTGCTTCTTTTACTGAAATTATTACAGCAGCTCAGCTGGAAAATAGAGGT

AGACAGTTGGCCTTTTTAGCTTTTATATCATTTTTGCTACGCAACTGGCCTTCTGAC-TC

TGTAGTGCCTGAAGCTGACAGACTTGACCTGGTCTGTGCGCCGGCATGGAGCAGAATGCA

GATATGGAGCCA------GAC---------------CGCCAGGTTAATCAACGA------

--------------------------------------CCTCCAAGATTCCGT-------

----------GCTCGA---GGAGCAGGGGTCCGCGGAAGAGGA------AG---------

--------------AGTGCGAAGAAGCGCTTTTAGCAGG-GGACAGCGACGACCCATTAT

TCGGGTAGATGACTTGCAGCTGCCCGACCCCCTGTATGTT------ATGCAAGCTTTGCA

ACGGGACCACACTTTAGAA----ATGCCCAGAGGGCAGGTAGATTTTAGCTGGATTGAGG

CTGAAGAGAGGCGGGTAGGTCCCACAGACGAGTGGTACTTTGAGGCTGTGAAGACTTACA

AA---GCTAAGCCGGGAGATGACTTGCAAACTATAATCAAAAACTATGCCAAGATTTCCT

TAGAATGTGGGGCCGTGTATGAAATTAATTCTAAGATTAGGGTTACGGGGGCTTGCTACA

TTATTGGTAATTGTGCCGTGCTTAGGCCTAACCTGCCTGCTGGAGAAGCAATGTTTGAGG

TTTTGAATGTT---GATTTTATTCCTTCTATTGGTTTTATGGAAAGGATAGT-GTTTTCC

AATGTTATTTT-------------TGATTGCAGGACCACCGCAACTGTAGTGTGTTGCAT

TAGTGAAAGAAACACCTTGTTTCACAATTGTGTTTTTTCTGGCCCTCACATGTTATGTTT

G--GACCTTAGGGCGGGGG------------CGGAGGTGAGGGGCTGTCACTTTGTGGGG

GCGGTGTGTGCGTTGCGTAGCA---AGGGGCTGTA-CAGTATTCGAGTCAAAAATAGCAT

TTT-TGAAAAGTGTGCTTTTGGGGTGGTGACCGGGTCAAAGGCTTCTATTAGCCATTGCA

TGTTTAAGGATTGTACCTGCT---CTATTATGCTGGGGGGTCAGGGCACTATTGCCCATA

GTCAGTTTATTGTAACTACTTCTGCTGAGGCCCCCATGAACCTGCAACTGTGCACTTGCG

AGGGTAATGGAAGTCATGTAGTTCCATTGGGGAATATTCACTTTGCTTCT---CACCGGG

AAGCTTCGTGGCCTACGTTTTATGCAAACACCTTGGTTCGGGTGCGCTTGTAT-ATGGGC

CGGCGCCGGGGAGTTTTTCACCCCA---AGCAGTCTACTTTGTC-AATGTGTGTAATTGC

AGCCCCTCGGGGGGTTGTGCAGAGA---ATTTATT--TGTTTGGTGTGTATGATGCTACT

TGTGCCATTATGCAACT-------GGGCGAGGCAGGC---AATGCTGCTAGTGAAAGACT

GTGTACTTGCGGGTTCAGACACAGCACCCCTTCCCTGCGGGCCACCTATGTAACTGAC--

-ACCAGGATTGACCGGGAGCTGAAC--TCTCAAGACACGGCTGAGTTCTTTAGCAGTGAT

GAAGATAATTTT--TA---GGTGAGTAG----------ATGGGCGTGGTTTGGGG-----

-------GAGTATAAAAGGGGCGCGGTACGTGGCT-----GT--------GTATTTACAG

CCATGGACCC---------TCAACAGAAGGGGCTTGTGAACACGTGTTTTGTGACTACGC

GTATTCCGTCTTGGGCAGGAGCAAGACAGAATGTCACCGGGTCAGATTTAG--AAGGAAA

GCCCGTGCCCTCAGATG-----TGCTGGAAAGTGGACGCCCG----C-------------

-----TTGCAGCCCCGCGCATCAGAACTTTGTATGA-GGAG-CAGCAG------------

------------------------CTGAACATGCTTGCGGTGAATGTT------CTT---

----------TTGGATGAGCTGAAGATCCAGGTGGCTGCCATGCAAAACTC--TGTGACT

GCTATT---------------CAGCGAGAAGTAAATG-------------ATCTAA----

-AGCAACGAATCG---CCCG--------------------------------AGAT----

----TAATGT--AAAAATA-AAATTTATTT--------CTTTTTT-GAATGATAATACCG

TGTCCAGCGTTGTCTGTCTGTAATAGTTCTATGAATTCTTTCCATGGCTTGGTACACTTT

AGCTTGAATGTTAAGGTACATGGGGATGAGTCCCTCGTCGGGGCTCA-AGTACAGCCAGT

GCATACAATTGTCAATGGGCTCAGTATTGTATACAATCCAATCAAAGTTAGTATTGAGGC

GGTGGTAGTTAAAAATATCCTTAAGCAGGAGGGAAATAGCAGTGGGTTGTCCCTTAGTGT

AGATGTTAATAAACCTATTAAGTTGGGAAGGATGCACCTTAGGACTGATGATATGCATTT

TTGCTTGGATTTTTAAGTTGGCAATGTTTCCCCCCTGGTCTCGTCTGGGATTCATGTTGT

GCAAGACCACTATAACAGAGTAGCCTGTGCACTTGGGAAACCTGTCTAGCAGTTTAGAGG

GAAAGGCGTGAAAGAACTTGGCTATGCCCCTGTTGCCCCCTAAGTCTTCCATGCACTCAT

CCATTACAATAGCTAGTGGCCCTTTAGAAGCGGCCCTTGCAAAA-ACATTGGTAGGGTTG

GTAACATCATAGTTGGCCTCATTGGTGAGTTCTGCATAAGACATTTGCACAAACTTGGGC

ATGATGCTGCCACTCTGAGGAACCACCGTGTTTTCGGGGCCTGCAAGGAAGTTTCCCTCA

CATATTTGGGTTTCCCATGCTGCAATTTCCTGGGGAGGAATCATGTCCACCTGGGGGGTG

ATAAAAAAGACTGTTTCTGGGGCGGGGACTATAAGCTGGGCAGACATGAGGTTCCTAAGA

AGCTGGGACTTGCCACACCCTGTGGGCCCATACACTGCAGCAATAAAGGGTTGCTTGTAG

TAGTTAAGGGATGCACAGCTTCCATCTTTATTAAGG------------------TACTTA

CTGACCTTCATGCAACACTGGGTTATGCTATCTTGAATATCTAGTAGGTCATTTAAC---

-----------AGACTGTCTCCCCCCATAGA-----------------------------

AAGCAGTTCCTCAAAAG-AGGCAAAGTTTTTGAGGGGCTTAAGTC----CCTCTGCATA-

----TGGCATGTTTGTT-----AAGCTTTGCTGAAGAGTTTGCAACCTGT-------CCC

AGAGGCTTTTTAAGTCA----TCCACTGCAGCTCCATCC----------AGGTAGTTTCT

GTGTTTCTTG--------GGTTTGGATGCTTGCGGCTGTATGGAA--TGAGACGATGCAT

GTCT---------------------AGAGCATGCAGGGTTTTGTCCTTCCATGGCCTCAG

CTTCCTTGTTAGGGTGGTTTCTGTCACTGTGAAAGGTTGCACGTGGGCCTGCACGCTGGT

GAGGGTCCTGCGCAGGCTCATCCTGCTGGTTTTGAAGACGTC------------CGAGCC

CTGCTGCATGTCAGCATA---GTAGCAAGCTTTTAGCACA---TCATAAGAGAGGGTGGA

GGTGGCATGACCTTTGGCCCTGAGCTTGCCCTTGCCCACG------TGATGGCACTTAGG

GCAGGTGGTGTCCTTGAGAGCATACAACTTGGGCGCCA-GGTACACTGATTCTGAGCTGT

AAGCATCCGATCCACACTTCTCACACTGTGTTTCACACTCTACTAGCCAAGTGAGCTCTG

GGTTGCTGGGATCAAAGACTAGCTTGCCTCCATTTTTTTTGAGTCGTTTCTTACCTTTTT

CTTCCATCAATTTGTAACCCTCCATGGTGGTGAAGAGGCTGTCAGTGTCTCCGTAGACAG

ATTTTAGGACCCTGTTTTCAAGTG---GGGTTCCCGCGTCGTTTTCGTAGAGAAACTGAG

ACCACTCTGAGACAAATGCCCGGGTCCATGCTAGAACAAAGGAGGCAATTTGTGAGGGAT

ATCTGTTATTGGCTATTAGGGGGGTGGACTTTTCTAAAGTGTGTAAACAAAAGTCATCAT

CTTCAGCATCAATAAACATGATTGGCTTATA---ATGGTATG--ACGTCA--------TG

CTAAAATTA-----------TGCATAAAAGGCGCTTCGGCGAGGGGGTCTTTAGTTGGAT

CTTCTTCGCTGCAAGAAGGCGGTGCC-----------AACTGGCGAACGTCAG-------

------------------------------------------------------AATTGA

CAGGTGGGTACGCTACGACAAA---TTCTGGCATGATTTCTGCACACAAATTGT------

------------------CTGTTTC----------TATGTATGAGGATGAT--------T

TGACTACATAGGCACCAGAAGCAATTTCTTTAGCAATGTTTGACTCTATTTGATCTGAAA

AAACTGTTTTTTTGTTGTCTAGCTTGGTAGCAAAAGACCCATAGAGGGCATTTGACAGCA

GCTTAGCAATGCTGCGCATGGTTTGATTTTTGCTTTTGTCTGCTTCTTCTTTGGCACTGA

TGTTGAGCTGCACATACTCTCTGGCGAGACATTTCCACTCTGGAAACACGGTGGTTCTGG

TATCAGGGATGAGGGTAACCTTCCACCCGCGGTTGTGCAGGGTAATAACATCTATGGAGG

TTGCAATCTCCCCGCGTAGGGGCTCGTTGGTCCAGCAAAGCCTGCCCCCCTTGCGTGAGC

AAAATGGGGGCAGCACGTCCAAAAACTCTTCTGCTGGTGGGTCAGCATCAATGGTGAAGA

TGCCCGGAAGAAGG-TCCTTATCAAAGTAGCTAATAGTTTTGTGA---TTTAGCATTTTT

TGCTCA---TAAGCCTTTATGGCCAGCGCCCGCTCATAGGGGTTTAGCGGTGAACCGGCG

GGAAAAGGGTGTGTAAGGGCACTTGCATACATGCCACATATGTCATAGACAAACACTGGC

TCTTGCAACACTCCAATGTAAGTGGGGTAGCACCTGCCTCCTCTGATGCTAAGCCTTACA

TACTCATACATTTCATTGGAGGGCGCCATGAGG------ATGGTACTTAGGTGGTGTGTG

TTAGGCTTTTCTGCTTTAT-----------------------------------------

----------------------------ACAAGATTTGTTTAAAAATGGCATGGGAGTTG

GAGCTGATGGTTGGACGCTGGAAGACGTTAAAGGAGGTTTCAGGTAAGTTTACAGAGTTT

TTAATGAAGATTTGGTAGGATTCTATAAGTTTGTTTACCAACTGGGCTGTTACTTGAACG

TCGAGTGCGCAGTAATCAAGGGTTTGCTGAATTATGTCGTAAGCG---------------

---------------------CCCTTTTTTTCTTTTATCCAAAGGTCTTTGTTAAAGGAG

TACTCCTCCTGATCCTTCCAGTACTTTAAGTCCGGAAAGCCATC---GGCGTCTTGT---

TGGTAAGAGCCAAGCATGTAAAACTCATTCACAGCCTGGTAGGGACAGCAGCCTTTGGAA

ACCGTTAGGCTGTATGCTTTGGCAGCATTTTTGAGGCTTGT---GTGTGTTAAAGAGAAA

GTGTCACGCACCATAAATTTTAT-------------G-TACTGGCTTTTCAAGT--CAGA

CGCCAGTACTTGGCCCT--GCTCCCAGTGCTCGTAACTCTTG------GCAGGCACGTAG

CTG---GGGTTAGGCAGAGAGTAAGTAATATCATTGAACAGCAACCTTCCAGCCCGCGGC

ATGAAGTTGCGGCAAATTT---CAAACACGGGTGGTATTTCTGCCCGCGTGC-------T

AACCACCTGAGCTGCTAGCAATATTTCATCAAACCCCGTGATGTTGTGTCCCACTGCATA

CACCTCAATAA--ATCTTGGGTTTCCTTTGAGCT--------TGAATTTTT---TTAACT

GATCTGGAGTAAGCTCTTCAGGGCTTTC-TAGTCC---CAAAGTAGTGGCTTTTTCTTGA

AGCTGGGGATTTTGGCAAATCACGTGGGACCAGAGGCTGGCGGCA---ATATGCTGCTGC

AAGGTGTCTCTGAACTTTTTGAATTTAACTCCTATGGCTCTTTTTTCTGGAGTGATGCAA

TAGTACAGAGTGTCCTGCTCTTTACCATTCCAAATTTCCCAGTTTTGCTCTGTTGCAAGA

GTTTTGGCAACATTGACCAGGTGGTCGTCCCCCAGCAGTTGGAAAACTAGCATAAATGGC

ACAAGTTGCTTTCCAAACTTGCCGTGCCACGTGTACGTTTCCACATCATATACAATAAAC

AGCCTTTCTGTGTTGGGCGGGGCCCCAATGGGGGTGAAAGATATTTTTTCCCACCAGTCT

GCGCTGTGTGCGGCCACGTGATGGAAGTAAAAGTCCCGTCTTCTGAGGGCGCAGGTGTGA

GTAACTTTAAAAAAAGAGCCGCAGAACTCACACTTTTGCATTTGAGAGATGTCTTTTATT

-AAGAAGACTTTACCTTGCT-----TAACTAAAAACTTGAGTGGGAAG------------

------------------------CTGAGCAGCGGAACCTCCGCTTGAAACTCTACCC--

------------------T-------CCTCACTGTTCCTCGCTGGTAGTAG---CAAATT

AGACTGTAGGGTGCCGCGGCCTCCTCTATGAGGGCGGGCAG------GTTGTGGGCAG--

-TGATG---GGCCTGTTTAAGCATGCGGGTAGGTCATA---GAGGTTAGTGTTGCAAAGG

TTAGTAAGCGCGCTGGCCAAGTTGGAGTGATACTTAATTTCCACACACTGTCCATTATCC

A---ACACGGCGGAAATGCTTAGTGTGGCGCGCTGGACCACTACGGCGCCCT--------

--------------------------------------------------------TGTA

CTTGCG--------------CTTGGGTGGGTTTACTATGGTGGAAGTTGCACCT---GCT

CGTGGTGAGCTGGCAAAGGCCTGTGCTGCATCCTGAGCTGGGAGGCTAGGGCCACTACTC

TCCTGTTGATTGATTGGATTTGGCGGTTCTGTGAAAAGACCACTGGTCCCGTGACCTTGA

ACCTAAAAGAAAGTTCCATAGAGTCAACCTCTGTATCATTAA-TGGCCACTTGTCTGAGA

ATTTCGCTCACGTCCCCGCTGTTTTCATGATAAGCAATATCTGCCATAAACTGCTCCATT

TCCTCCTC-----CTCTAGCTCC-CCTAGCCCAGCCCTTTCTACGGTGGCAGCCAGGTCT

CCCGACACGCGGGCCATAACCTGGCTAAA------GGCATTTTCTCCCATTTCATTCCAC

ACCCGACTGTAGACCACCTGCCCCTCGTGGTCCCTAGCCCGCATGACCACTTGAGCTAGC

TCTAGGTCAACCCAGCGGGAGCAGGGTGGGTACAGCCTAAGGTTGTGGTGCAGGTAGTTG

AGGGTGGTGGCTACGTGCTCAGCTACAAAAAAATACATGACCCAACGGCGGATGGTGAGC

TCATTAATGTTGCCCAGCATTTCCAGGCGCTGGATGACCTCATAAAAGTCAACTGCAAAG

TTAAAAAACTGTTCATTGCGAGCAGACACTGTGAGTTCCTCTTGCAGGAGGCGGATAGCT

TCTACTATAGTGTCTCGGACTTCTTCTTCAAAGGAACG---GCGAGGAGGGGA------G

GCAGGAGG---AGGAGGAGAGGCCTCTGCTTCGGGCTCAGACAGCTCCTCA---------

--GACACCGGAGGCGG---CGGGGGAGCC----CTGCGTCTGCGCCTGCGCACTGGAAGC

CTGTCTATAAACCTTTCTATCACCTCTCCCCGCCTGCGCCTCATTTC---CTCTGTAACT

GCACGTCCATTT---TCCCTGGGCCTAAGCTCGA----AGGCTCCCCCAGAGAGC-----

------------GGGGTGCAGGGCGCGGGT-------CTGGGCAGGCTCAGGGCGCTAAT

TATGCTTT-TTGTTACAGATTGGCTGGGTAAAGACTGA------ATTGTTTGA--AACT-

---GTGACAGCTCCGGATCAGTGAATTTTT--CCACAAAGGCATCAAGCCACAAACAATC

ACAAGGTAAG---CTTAGCATGGTGTGCGG------------------------------

---------------------------------------GTTGCGGGGGCTAACTAAAAA

GTTGAAAAAAGCTGTCTTAAGCTTACGGATGGTGGTTAGAATAACTAGGTCTTTGTTGCC

CGCCTGCTGGATGCGCATGCGGT-CTGCCAGTCCCCATGC-TTCGTTTTG-ACACTGGGA

CAGGTTTTTG--TAATGGTCTTGCAAGAGCCCCTCTACGGGCACCTGCCT----------

------------------------------------------------------------

--G---TCGCCTGCCATGTTGGTGGTGCCATAGCCCCTAAGTGGCCTAATAAGGGCCAAA

TCGGCCACTACGCGCTCTGCCAGGACCGCTTGCTGAACCTGCGCCAGGGTCTCTGCAAAG

TT------------TTCAAAGTCAATGAACCTGTGGTAAGCTCCAGT-GTTAATGGTGTA

TGAGCAGTTGGCCATCACCGTCCAGTTTAGTTT--TTGCTGCAAGGGCAGGTGTATCTCT

GTG----TACTTTAGCCTGCTGTAGGACCTGGTGTCAAAG-ACGTAGTCATTGCACGTTC

TCACTAGGTACTGGTA-GCCCACCAAAAGGTGGGGCGGGGGGTACCAAAACAGTGGCCAG

CCCTCGGTAGCGGGCTCTCGAGGGCTGAGGTTCATAAGCATGAGGCGGTGGTATTGG-TA

TATGTACTTGGACATCCATGCCAGCCC-TACAGAGGTGGTGGAAGCTCTGGTGTACTCTC

GAACCCGGTTAAAAATGTTTCTGAGAGGCCTGAACACCTCAACGGTGTATGGGGTTTGTC

CGGTTAGGCGGGCGCAGTCCAGCGCGTTCTGC----------------------------

------------------------------------------------------------

------------------------------------------------------------

------------------------------------------------------------

------------------------------------------------------------

------------------------------------------------------------

------------------------------------------------------------

----------------------AAAACAAACAAGGAG-TCGATGGCA-------------

--------------------------------------------CTTTCATTCGCTCCA-

---------TTGCAG-ATGCATCCGGTACTGCGACAGATGAAGCCCC-------------

-----------------------------TGCCCGCCT----------------------

-----------------------CTGCCAGCAAGCTAGAA------------GC------

---------------------------AGAGAGCGAGGGTTTGG-CCCGCTTACAAGGGG

GTGCTGCTCCAGAGT--TGCACCCCCGGGTGCAGATGAAGCAAGAGGCGGCGGAGGCTTA

CATCCCCACTAGCAATGTGTTTAGGGA---------TAATGAAGGAGAAGAAGCAGAGGG

CTTGCGACACTTAAAGTATGAGTCGGGGAGGCTGTTGCGTAATGACCACACTTCCAAGC-

GGGTGCTGGGCGAGCGAGACTT--TGAAAAGGACCCCCAGAATGGCA------TTAGCGC

GGCCGAGGCACACCTAAAGTCTGCAGACTTAGTCACGGCCTATGAGCATACTGTGAAGGC

AGAGGTTAATTTTCAGACCACCTTTAACAACAATGTGCGTACCCTCATCGCCAGGGAAGA

GGTGGTCATTGGCCTGATGCACTTGTGGGACTTTGTGGAGGCCTTTCTAGAAAACCCAGT

G-------AGCAAGGCCCTCACTGCCCAGCTCTTTCTTATAGTGCAACACTGCAG-GGAC

GAGGGGGT---GCTGCGGGAAA--GCTTGCTAAACATTGCCGAGCCT----GAGAGCCGG

TGGCTGGTGGACCTGTTAAACCTGTTACAGACTATTGTGGTGCAAGAGCGAGGCTTGGCA

GTTGGTGAAAAGGTAGCGGCTATAAACTACTCTGTGATTACCCTAAGTAAACACTATGCA

AGAAAGATATTCAACTCTGTGTTTGTGCCCATCGACAAGGAAGCCAAGATTAACACATTT

TACATGCGCACTGTTGTTAAGCTGCTGG------TGCTGAGTGATGACCTTGGCATGTAC

CGCAATGAGCG--GATTGAGAGGGCAGTGTCTGGTGCCAGGCAG-----CGGGAATTAAA

TGACAGGGAACTTATGCACCGACTGCGGCAAGC----CTTGGCTTCTAACGGACTGCCGG

AGC------TAGAGGGC---GAA---GACAGTGCTAATATTA--GGTCCAAAGAG-----

--GAGTGGGGGGTCGGAGC------GGGCGGAGGC-GTTGCGAGCGCGCGCTAC------

--CCCCACCTGCTAGACTATGA------GGAAGAGGAG---AAC-------------CCT

GATGGCT---------------------------CCGTCTCATTTC--AACAGCATGAGC

GCGGCGCTCAACCCCATGAAAATGGCGGCCATGCAGAGTCAGCCTACAGCCGAC------

---GACAGTTGGGCCGCTTCTATTAGTCGCATTATGTCCCTAACGGCGGGGGATCGGCAC

AAGTTTTCATCCCAGCCTTTTGCTAAC-AGGCTGGATGCTATATTAGAGGCCGTGGTGCC

TTCCCGCAAAGACCCTACCCATGAAAAAGTGCTAACTATAGTAAATGCCCTAATAGAAAA

TGGTGCCATTAGGCGGGACGAGGGGGCGGGGGTGTATGACGCCTTGTTGCACCGCTGTGC

CAAGTACAACAGCTTGAATGCCCAAAGCAACCTAGAGAGGTTGGCTGGAGACGTGA----

--GGGAGGCGGTAGCGCAGCAAGTGCGCATCGCC---ACGGGCAACCTTGGGTCCCTAAC

CGCACTTAATGGCTTTTTGGCACGCCTGCCCGCCAATGTGGAGCGGGGCCAGGAGAACTA

CACTGGCTTTGTGTCAGCCCTTAAGCTGCTGGTAAGCGAGGTGCCCAG---TACAGAGGT

GTACCAGTCTGGGCCTCACTACTTTTTGCAAAGCAGTCG-CA--ACGGCACCCAGACAGT

TAACCTTACCAATGCCTTTGAGAACCTAAAGCCCCTTTGGGGAGTTAAGGCCCCCACTAT

GGAAA-GGCTCAGCATCTCAGCCCTG-----CTCACCCCCAACACTAGACTGCTGCTTTT

GCTAGTG-TCTCCCTTTA-CAGACAGTGTTAGCATTAGCAGGGACTCCTACTTGGGTTAC

CTGCTTACCCTGTACAGGGAAGCCTTGGGCCGCAATCATTTG---GATGAGCGTACTCTT

GAAGAGGTCACAGAGGTCAGCAGGGCCATGGGCAGTGAGAACATAA---ACAACTTGCAA

GCCACCCTTAACTTTTTGCTAACCAATAGACAAAAAAGAATTCCCAAAGACTACTCCCTA

ACCCCAGAGGAGGAGAGGATAGTGAGGTTTGTACAGCAGGCCGT-GAGCTTGCGCATGAT

GCAGGAAAACTTGAGCC--CCACTGAAGCTTTGGACGTAACCGCGGCCAACATGGAGCCC

AGCTTCTATGCCAGCAACAGGGATTTTATTAATAAGCTTATGGACTACTTTCACCGCGCT

GCGGCTATGGCTCCAGACTACTTTTTGGGGGCAGTGATGAATCCCCGCTGGCTCCCCCCT

GAGGGCTTCTTTACTGGGGTTTTTGACTTTCCTGAAAGGGA---CAGC---TACACCTGG

GATGGACTGGATAGCTCTTTG------GAGCT---GACGCGCCAGGATGC---CATGCGG

TTTCTGGAAGA---------CAAGTTTATGGACGATGACCAGAGAACAG-----------

AGTCT---------CGCAGCCTCAGCAG------AGTTACCACCC--------CAGCTAG

CTC------TAGGCGGTCCTCGGTGACCATG----------CCACCTGGGGGCTTTATGG

GCATGA-----TAAATAACAA-------CAAAAACGATAAC---------------AGCC

TCAGGGAGATAGATGGATTAGCAGACAAGCT-GGCTCGGTGGAAAACATACAAGCGTGAG

TCAGAGGAGGCTCGTGT------------GTCCCTGCCTGC---GGTAGTAAGGCCTAAG

---------CAGTACAGGCCCCGCTCCCCTATCTCCAGCGATGACAGTGATGATGGGATG

AGCAGGCCAGACCCGTTTCTCAAGTTTGAGGGGAGTGG------------AAACCCCTTT

GCCCACCTACGCCCCAAGCTGGGGCGTGGCCTGTAAA-------------GTGAATAAAA

--------------TACTCACCAGAGACATG----------ACGCTAGCGTTTTT-----

--TTATAGC----------------AATGGAG----------------------------

T--TTTCGTCGTCTCCTCCCCCGTCTTATGAAACAGTGAT--------------------

----------GGCA---CAAGTGCCTTCGATCCTG-GCGCCGCTGGTACCCCCGCGGTAC

AAAGGG-GCTACAGAAGGAAGAAACAGTATCCGTTATTCCCAGCTGCCGCCTCTGTTTGA

CACTACAAAGCTGTACCTCATTGACAACAAGTCTTCAGACATTCAGGCCCTCAATTACCA

AAATGACCACAGCAACTTTTTAACCACTGTGGTGCAAAATGCTAATTACACGCCCATGGA

GGCTAGCACCCAGTCCATCCAGCTGGACGAGCGGTCGCGCTGGGGTGGGGAATTTAAGTC

CAT--TTTACACATGAACATGCCTAATGTGACAGAGTACATGTTTAGCAATAGCTTTAAG

GCATACCTTCCCGCCACAGCAGACAACTTTGGCAA-------------------------

-----------GGTGCTCACCTATGAGTGGTACACCCTAACCATTCCCGAGGGTAACTAT

TCTGAGGTTATGCTTTTGGACCTGCTGAACAACGCGGTGGTGGAGAACTATCTGGCCCAC

GGGCGCCAGCATAATGTTAGGGAGGAGGACATGGGCCTCAAGTTTGACACCAGAAACTTT

CACCTGGGCTTTGATCCCGAGACCAAGCTGGTCATGCCGGGCTTCTACACTAACGAGGCC

TTCCACCCTGACATAGTGCTAAGTCCAGGCTGTGCGGTGGACTTTACCCACAGCAGGCTC

AACAACTTTTTAGGCATTAGAAAAAGGCTACCATACCAGGAGGGATTTGTGATTACCTGG

GAGGACCTGCAGGGTGGCAACATTCCAGCCCTGTTAGACCTGGAAAATTAC---------

---AACCCAGACATTCC------------------CGGCGCCGATATCAC----------

------------------ACCACTGATG--------------------TATGA-------

------------------------------------------------------------

------------------------------------------------------------

-----------------CTCCAAGGGAAGGCCTTACCATGTTGGTGAG---GATCCCAGC

GCG---GGCAGTACCTTCACCTGGTACCGCAGCTGGTTTGTGGCCTATAACTACGGTCCT

GC---CGAT---GGCATCAAGAGCAAAACTGTACTGGTGGCCCCTGACATTACTTGCGGA

GTGGAGCAAATTTACTGGAGCCTGCCAGATATGGCGGTGGACCCAGTAACTTTTACCTCT

AGCC---ACAATCCCAGCAGCTACCCAGTGGTGGGCACGGAGCTGCTACCATTACTGCCA

AGAAGCTTTTACAATGGATCTTCAGTGTACAGCCAGCTGTTGCAAGAAAGCAC-CAGTCA

--AACTCAAGTGTTTAACCGCTTTCCGGAGAATGCAATTCTTAAAAGGCCCCC-TGCTCC

CACCATTATCAGCATCAGCGAGAACGTGCCCGCCCTCAGTAACCATGGCACCCTGCCCCT

AAAAAACAACATTCCCGGGGTGCAGCGGGTGACCATTACGGATGCAAGAAGAAGAGTGTG

TCCCTACGTGTACAAAAGCTTGGGGGTCGTTGTGCCTCGCGCGCTGTCCAGTAAAACCTT

CTAGTGAC------------------AAACTTTTTCTTTTTATTAGC-------------

--AA-AGCCATGGCCATCTTAATATCACCGACTAACA-ACACCGGCTGGGGCCT-AGGCA

C-----TCACAAGC------TCTTTGGAGGAGCCAAACAAAAATCAGAT--CAGCATCCC

GTTTATGTCCAAGCTCACTACCGGGCCTCGTGGGGCA---GCAAAGG-------------

--------G--CGCCGCCGCCGTCAAGGC---CGAGCCCGCGGGGC--------------

CCCTCTTGACCCTAAAACTGAAGCAGAA-------ATGGTTGCCACCATCGACGAGGTGG

CGCGCAACGGTCCCCCGGCGGCGCG-CTTGGTGCTGGAGGC--TGCCCGGAGAGTGGGGG

CTTACAA-----------CCTCCGTCGAGCGC----GCAAGCTAACCCCT----------

-GCGGGGCGTG-----------------------CCATGAT-GGCCATGCGCGCGCGTCA

GATGGTAAAGCAGGCT--------------------------------------------

--------------------------AAGAAAA---------------------------

---GGAAGCGCAGG-GTTCGCTTTAGA--CAATAAAGTC---------------------

------------------------------------------------TGTCCTC-----

-----------------ACATACACACATA----------------------------CC

CGAGTGATGGCCTATTTT-------------------T------TTACTG----------

-------------------------AAGCCTCATCGCCATGGC--AGCAATC--------

-----------------------AGTCGCGCCATCAAGCAGG-AACTGC--TGGAAGACC

TCAAACCTGAAATCTATCTGCCACCCAAGTCCACCAA--------ACGGAGAACTAAAGT

TAAAACAGAG-----------------GAAAAAGTTG--------ATGTGAAAACCCTAG

TCAATGCCAAGAGCAGAAAGCGCAGGGCTGCCAAGGAT----------GAGCTG--GAAG

AGGATGTAGAGTTTGTTAGGCGCTTTGCTCC------CCGGCGTCCCTACCAGTGGCGGG

GTCGGCGCGTGCGCGCTCTGCCTAGGCCCGGGGTGCCCGTGGTGTTTACTCCCGGCCAG-

AGGT--CGGGCACCGCCTCAAAAAGGTCTTATGACGAAGTGTACGCAGATGAGGATGTAC

TGGACCAGGCGGGAAACATGATTAATGAGTTTGCTTATGGCAAGCGAGTCAGGGT---GC

TTACCCACAAAAACCCCACTCCCTCACAAGTGCCC---ATCACCCCCCAGGAGCCCGTGG

CGCG---------------TCCTGGCGAGGCACACCTGCTACCCACAGTGCAAGTGCTGG

CTCCGCGAGGCTCTAGGCGGGAAACCATGTTGCCCGTAACCA--AGTCCGAGG-GGGGAG

ACGTGAAAGTGGAAAACAAGGGCTTTGAGCAAA--TCACCCCCGGGCTGGGGGTGCAGAC

TGTAGACATCAAAGTGCCCGTGAAGCGCAAAGGCGA-------------TGCAGAGGATG

AAATAATAAAGAGGGTTAAGATGGAGCT------TGAACCATATGAGACCACTATGAAAA

TGGAGTACTCTGAGGAGCCCCAGGTTGA---GGCTTTTGACACAGGGATAGAGCCCAGCT

CTTTTTTTGAGGT-GCGCTC---------------TCA--AGCGCGTCCCATCGCTGTGG

CTAGGAAGCGCCGCACCGCAGCTGCCAGC-------GCCCCCGCTGTAGAGGTGATGGAG

GT--TCAGCAAAGCAAC----------CCTGCCACCCCAGCTACCGCTGCCGCCAGAACC

GCGA---CCGCGC----TGGGCCCGCGTTTGGC-TCGTCGGCCTTCA-AGATGGG----G

GCCCGCCAATGCCATCTTTCC----AGATTACAAATACCACCCCAGCA--TCACGGCTAG

GAAAATGAGGGGCCCCAAGCCTACAGGCAAGATCAGCCGCTGGGGGCCTGCCAACAGCAT

CCTGCCTGAGGTGCGGCTGCACCCCAGCATGGTGTCTGCG--------GTGACCAGAGCT

GCCCCT------CGCAGAGTCACCAAAACCCGGAGACGCCGGCGTGCCA---GAACCCGC

CAGGCT------TTTGTGCTGCCCGCGCGCACCAAGACGGGCGCGCTGCTTTCCCAGAAC

GTGCGCTACCACCCTTCCATCTCCT---TGCTCCGCCGAGC-CTAA---------TCTG-

------TAT---GTTTTGCTTTGC------------GCAAGGATGGCCGGCAGGAATGTC

ACCCTTCGTCTGAGAGTCCCAGTGCGCACCAAAATTACAGGCGCAGGTCGTCGGCGCGG-

--ACGGCGCCCAC-GCGGCATACGCTGCGGGCG----CATGAGAGGAGGCTTTTTGCCAG

CCCTCATCCCTTTGATAGCTGCCGCCATTGGGGCAGTACCAGGAATAGCTTCGGTAGCGC

TCCAAGCTGCCCGGCACTAGC-TTTTTACAGA----------------------------

----------------------TTGACTCAC------TCCCATTT---------------

----------TTCTTGT---GCCAGAAGAAGACAA-------------------------

-AATGGATGCTGTCAATTTTTCCATCTTGGCACCACGCTACGGGGCCCACCCTATGATGA

GTGGTTGGTCTGGCATCGGCACCAGCGGCATGAACGGCGGGGCCTTTAACTGGGGGGGCA

TTTGGAGCGGCAT---------------CAAAAACTTTGGCAGTAATGTAAAAAGCTGGG

GCAGCAAGGCTTGGAACAGCCAAACGGGCAAGCTGCTCAGGCAAAAGCTTAATGACACCA

AAGTTAGAGAAAAGCTGGTGGAGGGCATTTCCACCGGGGTGCACGGGGCACTTGATATCG

CCAACCAGGAGTTAGCAAAGCAAATAGAGCGGCGTTTGGAGC---GTCAGCAGCCCCTAG

AGCCGGAGGTGGAGG----------------TAGAGGAGGAAGTAGTGGACATAAAACCC

GAAGCGCAG------------GCCCCGCTGGTGGTACAAATACCCAAAAAGCGCCCCCGC

GACGAAGAC---TTGCTCATAACTGCGGACGAGCCTCCCTCCTATGAAGAGTCCATCAAA

ACCATGGCA------CCC-CTGATGCC----CATGACCCGCCCGC-----ACCCTTCCAT

GG------------CCAAACCTGTTTTGGTAGACCGTCCCACCACCTTGG-AGCTAA---

-----------AGCCCTCTGAC-------CAGCCGCCAGCCTACTCCCCCC---------

-----------CGGCGCCCAGCGCCGTCAGG------GTCACAGTGCC---------CTC

TAACATTCCC---------GTGGTCACTCCTGCCAGAAGCAGAGGCTGGCAAGGAACTCT

GGCCAACATTGTAGGAGTGGGTCTAAGTAACGTGAAGCGCCGTAGATGCTTCTAAC---T

GTGATTAAAT-ATACTCAAGTGA-CTTGAAGT-------TTGCCGACCCTGTCTTTTTT-

------------------TGACCGCGCCAGCTGA-------------------GAAGATG

GCAACCCCGTCGATGCTGCCACAATGGTCTTACATGCACATTGCTGGCCAGGACGCCGCC

GAATACTTGTCTCCCGCCCTGGTTCAGTTTGCCCAAGCAAC-CAGTTCTTACTTTAAGTT

GGACAACAAGTTCAGAAACCCCACTGTGGCCCCCACCCACGATGTGACCACTGAGAGGTC

GCAGCGCTTGCAGCTGCGCTTTGTGCCAGTCATGCAAGAGGATGGCCAGTACACTTACAA

AACCCGCTTCCAGCTTGCGGTGGGAGACAACAGGGTGCTGGACATGGCCAGTACTTACTT

CGATATCAGGGGTACCCTAGACAGAGGCCCCTCCTTCAAGCCTTACAGCGGCACCGCCTA

CAATGCCCTCGCCCCCAAGGCCGGGGCTAACAACTGTC-------------------TTT

TTAATGGACAGGGTG---------------------------------------------

-----CCAATA------------------------TTAACACTTTAGCCCAGGTGCCCTC

TGCAGGTGCCATAACTGTGA---ATGG---------------------------------

-----C--------CAAGCTGCTGTCACAAACAATACCTACCAGCCAGAGCCCCAGCTGG

GCCCTGAAAGCTGGGTCGATGGCAGC---CTAGCAGAGCTGGGGGATGCGTCTGGCCGTG

CCCTTAAGGCTTCAACCCC---GCGCATGCCTTGCTATGGTTCCTATGCTCCCCCCACCA

ACGAAAATGGAGGTCAAGCAA------------------CTGGTCC--------------

-AGTGG----------AATCCAGA-----TTTTAT---------------AAGGTGACCA

CCAACAATAAC---AATGAAGCAGATGCCATGCTATACACTGAAGATGTAAACCTGCAGG

CCCCAGACACCCACCTGGTGCACCAAGTGCCAGAGGGTCAGGTTAC------AGGGGTGC

AAGGGCTGGGCCAGCAGGCTGCGCCCAACAGGCCGAACTACATAGGCTTCAGGGACAACT

TCATAGGCCTCATGTACTACAATAGTAATGGAAACCTAGGGGTGCTGGCGGGTCAGTCAT

CTCAGCTCAATGCCGTGGTGGACTTGCAAGACAGAAACACAGAGCTCTCTTACCAGCTGC

TGCTGGATGCCCTCACAGACAGGTCCCGCTACTTTTCCATGTGGAACCAGGCTGTAGATA

GCTATGACCAGGATGTTAGGATTATTGACAACCATGGCGTGGAAGATGATATGCCCAACT

ATTGCTACCCACTGAGCGGCATGGGGCCCCTAACAAACATGACCACCATGAAGGTTAAC-

-AA------CCAAAA----------------CTTTCAGGCAGAAAATACCAATGTGGG-G

CCCATTCAAAAGATTGGTTTTGGAAATGTTGAGGCCATGGAAATCAACCTCAATGCCAAC

CTCTTCAAAAGCTTCCTTTACTCCAATGTGGCCTTATACTTGCCTGATGCCTTTAAATAC

-------ACACCTGAAAACATTGTGGCC--CCTGCCAATGTGAA---TACCT---ATGCT

TACATGAATGTTAGATTACCCGCCGCCAACCTTATAGATACCTTTGTAAATATTGGCGCC

AGATGGTCACCAGATGTAA-TGGACACTGTTAATCCTTTCAACCACCACAGGAATGCAGG

ACTCCGCTACCGTTCACAACTGCTTGGCAATGGCCGCTATTGCTCGTTCCATATTCAGGT

CCCTCAAAAATTTTTTGCAATCAAAAATCTCCTCCTACTGCCTGGAACGTACACGTACGA

GTGGTCTTTCAGAAAGGATGTAAACATGATCCTTCAAAGCAGCTTGGGCAATGACCTCCG

AGTGGATGGGGCCACCATCAACATTCAGAGCATCAACCTATATGCAAGCTTTTTCCCAAT

GGCACACAACACTGCCTCCACTCTGGAAGCCATGCTGCGCAACGATGTAAATGACCAGTC

CTTTGCAGACTACCTGTCTTCTGCCAACATGCTTTATCCCATCCCTGCCAACACTACTAA

CCTGCCAATCTCCATTCCCGCCAGAAACTGGGCGGGATTTAGAGGGTGGAGCTTTACCAG

AATTAAGCAACGAGAAACTCCTGCCCTGGGCTCGCCTTATGATCCCTACTTCACTTATTC

GGGCAGTATTCCATATCTGGATGCAACTTTTTACCTCAGCCACACCTTTAGAAGAGTTTC

CATCATGTTTGACTCTTCCGTGTCTTGGCCTGGCAATGACAGGCTGCTTACCCCCAATGA

GTTTGAGATTAAAAGGTATGTA------GACGGTGAAGGTTACAATGTGGCCCAGTCCAA

CATGACAAAAGACTGGTTCATGGTTCAAATGCTAGCCCACTACAACATCGGCTACCAAGG

CTACCACCTGCCAGAAAGCTACAAGGACAGAATGTACTCCTTCCTAAGAAACTTTGAGCC

CATGTGCAGACAGTTGGTGGACGTGGC----CAACT---ATGCTGCCTACCAG--CCGGT

TACCGTGGGCCACCAG-----------------------CAT-------AACAATTCTGG

TTATGCTAGCGCCCTTTC---GGCCTTTAACCCGCGTGAG----GGGCACCCATACCCAG

CAAACTGGCCTTACCCACTCATTGGAGCCAATGCAGTA---------CCCACTGTCACCC

AGAAA--AAGTTCCTCTGCGACAGGTCCCTGTGGCGCATCCCATTCTCCTCCAACTTTAT

GTCTATGGGAACCCTCACTGACCTGGGCCAAAACCTGCTGTACTCTAACTCCGCCCACG-

CCCTTGACATGACTTTTGAGGTTGATGCCATGAATGAGCCCACTCTGTTGTACGTTTTGT

TTGAAGTGTTCGACGTGGCACGCGTCCATCAGCCCCACCGGGGGGTTATTGAGGTAGGGT

ACCTCAGAACTCCCTTCTCCGCCGGCAACGCCACCACCTAAGCTAAT-------------

------------------------------------------------------------

---------------------------------------------ATGGCGGAAGGAGG-

-------------------------------------------GTCCTCAGAAGAAGAGC

TGCGAGCCATAGTACATAACTTAGGCGTGTCTCCATTTTTCTTGGGCACCTTTGACAAGA

GGTTTCCGGGTTTCATCT-CTTCACAAAGAATGGCCTGCGCCATAGTAAACACCGCAGGC

CGAGAAACGGGGGGCGTGCACTGGCTGGCCATGGCCTGGAACCCCCGCTCAAAAACGTTT

TACATGTTTGACCCCTTTGGATTTTCAGACAGTAAGCTCAAGCAAGTGTACAGCTTTGAG

TATGAGGGCCTACTCAGGCGCAGCGCCATAGCCTCCAGCCCGGATAGGTGTGTCACTTTG

GCCAAAAGCAATGAAACCATTCAGGGTCCCAATAGCGCCGCCTGTGGACTGTTTTGTTGT

ATGTTTTTGCATGCCTTTGTCAACTGGCCTGACGACCCCTTTGACCACAATCCCACCATG

GGACCCCTCAAGAGCGTGCCTAACTACAAACTCAATGATCCCAC-AGTGCAGT-ATGTGC

TCTGGGGAAATCAAGAAAAGCTGTATAAGTTTTTGGAAAAACACTCTGCTTACTTTCGCG

CCCACGCCGCTGCAATTAAAGCCAGGACTGCTTTTAATAAATTGAAA-------------

----CAATAAA-----CCG-------TTTATTCGAAAAAT-ATAAATGGTTT--ACAGTG

TGAT-TATT------------------------------------AAAAAAGGACAAGGT

CATCGTCATGC-----------TGGCCCTGG----------------GGCAAAATGGTAT

TTTGGTACCTGTG-----TTCATCGGACCAGTGGAACTCTGGGAATTTAATTGGGGGC--

----CTCTCGCCCACTGTGGACG---TCCAT-----ATTTGCTTAGCAATTTGGACACAT

GACACCAGGTCCACGCTGGAAATCTTAAAATCACAATTTTTTTGGGGGGCGGCCTTGC--

-----TGTTGCGGTACAC------TGGA--TTGCAACATTGAAACACTAGCATGGCGGGG

TTATTAAGGGTGGC---TAGCATTTTTTGGTCATCCACTTCACTCTTGTCAATGTGACTA

GCGC-TTGCA-----AGAGAGAATGGG---GTAATCTTGC----------AAGTTT---G

CCTGCCCAGC---------------------AAAGGAAGGTTACTG---TTCCAATTG--

CACTCA-----CACTTGAGTGGCAT-TAGCAAGTGACTCTCA------GCATTTGGCATA

GAGGGGTAGCAAGCCTTAAGAAAA-------GCCATGATCTGCTGAAAGGCCATAATAGC

TTTGGGTCC----ATCGGTATAAAACATACCGCAAGAGGCCCCGCT-------AAAGTTG

CCCCCGCTCATATT---------AACATCCATGCTGCAGCAGAAAGCATCCTCATTC---

-TTAAACTGTA--CTACATTCCTT--------CCCCACCGGTTAGAAACAAT----TTTG

GTTTTTTCTGGGTTTTCCT----TAAGGGCCCGCTGGGCA-----TTTTCACTGT-----

----TAACATCCATTTCTACTAGCTGCTCTTTTTGGAT--------------CATGGGCG

ACCCGTGCAGACACATAAGGCTGCTCT-------------------G--GCATCCATGTT

----------GCCACA----CCACACAC-----CCGCTGGGGTTA---A-----------

-CCCCAGGGGCAAGCTCTGCTGCTTTGATCACAAAATCAAGCAGCAGGCGCCCTGCCACG

TGC----AAAAAA----------------------GTTTTCTGGTTGGTAAAGGTGTAAG

TAACGT---------GCGTTTTAGAGGTGGTAATGTAAGCC----TGGACTGCTTTTTTA

AAGCACTCCAGCGTGCCCTCGTC------TGGAAGCAAGGT-------------------

-T--------AGCTTGCTGTGGTCTACCT----GGTACGCG--GTGAGAACCTTAAGGGC

AAGCTCCAT---GGCAGACTGCCACTTGT-------------------------------

--------------------GCTCA---CTAGCTTTAAAAAACTTAGAGGCC--------

-------------------------------------ACTAGCCCC-------GAGGGCG

CGTTGGGCTTGCTCCAAACGGACT----TTTGCTTGGGTGGGATAAATACCACATTTCCC

TCCTTGTCAGTGTCCACC-GTTTGGCCACAAAAGGTCTGCAC-TACAATGCTCCCGGGAT

CCCCCC-------G--CGCCTTGAGGGTGGCGGTGGCTTG-----------ACG-GGCTA

TTTCGACCATGGCTT---------------------------------------------

----------------------GGTGGCCCCTT---------GGCTCCTC----------

-AACG-GCTTTCCGCTGGCGCTTCTTTGGGG---------------GCGCCG--------

------------------------------------TAGGCGGTG---------------

--------------CCACAGGAACCTCTTCAT---------C---GCTGG--AGCTTTCA

GAGATTG------------------CAACAACCTTCTTGTGGCT----------------

-----CATCTTTT---------------T-CCTAGATGTCA----GAAGAGCCCGTCAGT

GGGACTACC--------------------------------------------------G

TGGAGACA---AAGGAG----------------------------GACATTCACAC----

---------ACCTCCAA-------------------------------------ACTCCC

CTGTCCTCG---------------------------------------------------

-------AGACA------------------------------------------------

A--------------TCTCTCTAAGCCCCGAACCCGAGGCT-------------------

-----------------------------------------------GAGGCCTG-----

-------------C---CCAAA---TAC--------------------------------

------------------------------------------------------------

--AGAC--------------------------------------------AAATACCTAA

GCACCAATTTGC------TTTGCAAACACCTGCAACGTCAAAGCGCAATTGTTCTGGACA

GTATCAAAGATCATCTACA-AGTGCCCACC-AGTGTATCAGAGCTAAGCTGCGCCTAC--

GAACGAAGCTTATTCTCCC-------CAAACACCCCACCAAGG-----CAACAAAGCAAT

GGAACGTGTGAAGCAAATCCTAAACTTAACTTTTACCCTACTTTTTTGGTGCCTGAAACT

TTGGCAACTTATCACATCTTTTTTGTTAATCAA-AAGATACCAGTATCTTGCAGAGCTAA

CAGAGCTAAAGCAGACAGAGCTCTTGCTTTGCAAGAGGGAGATTGCTTACCTGACTATGA

AACAATGGACACAGTCAGCCGGGTGTTTGAGGGTTTAGGCGGTGAGGTGGT---TGCGGA

TCACG---CGCTGCA------AAATAATGAC---------------TCTGTATTAGTGGA

GCTTAAGGAAGACAACCCCCGTTTGGCTGTGGTCAAAAGAAACCTCA---GCGTCTCCCA

CTTTGCCTACCCCGCCGTGCACCTGCCGCCAAAGGTCATAACCACTGTCATGGACAACCT

G---------CTAGCTAAAAGGGCCGCCCCCAGCGC---------CGACGTCTCAGAGCT

AGA---TCCCGAGGGGGGCCAAGAGGTGGTGTCTGACACCGAGCTAAGTAAGTGGCTAAA

----CACCTCTGAC-----CCCGAGGCCT------------TAGAAAAGCAGCGCAAGAC

AGTAATGGGAAGCGTGCTGGTTACTGTAGTGCTTGAGCGCATGCAAAGGCTTTTCACCTC

AGAAGAAATGGTGAAAAAAATTGGGGAGGCCCTCCACTACACCTTTAGGCACGGCTACGT

ATCTCTGGCTTGCAAGATTTCTAATGTAGAGCTGACCAACGTGGTCACCTATATGGGCAT

CCTGCATGAAAACCGCCTGGGTCAAAC-CACCCTGCATCACACAGTGCAGGGCGAGGCCC

GTCGAGACTACATTAGGGACTCCATCTTCCTCATCCTTATCCATGCCTGGCAAACCGCGA

TGGGCATCTGGCAGCAGTGCCTCGAAGAGGAAAACCTTAAAGAACTAGCCAAACTGCTGC

AAAGGATTAAAAAGCCATTGTACACTGAAACCTCCCAGCGCCTCATGGGCAAG-CGCCTG

GCAAACGTAGTC-TTCCC--ACCCAAACTGCTAGAAACCTTCCACAAGGGCCTTCCT-GA

TATTGTTAGCCAAAGCATGATGCAAAACTTTAGGTCTTTCATTTTGGAGAGGTCCGGCAT

CCTGCCCTCCATGACCTGCGCGCTGCCCACAGATTTTGTACCAATTCACTTTAAAGAGTG

CCCCCCCACTATGTGGCCCTACACCTACTTGCTCAAATTAGCAAACTTCTTTATGTATCA

CAATGACCTTTGC------TATGATGTGGGAGGCGAGG------GC---CTGCTAGAGCA

CTA---CTGCCGCTGCAACCTCTGCACCCCCCACCGTTGCCTAGCCACCAACCCCGCCAT

GCTTAATGAAACTCAGCTAATAGGTACCTTTGATATTCGGGGTCCCGGCGGAGAAA--AC

GGAGCAGAGTCTTCCTC-TGGCCTTAAGCTCACCGCTGGAATGTGGACTTCCGCGTTTCT

GCGAAAATTTGAAAGCTCTGATTATCACGCCCACAAAATTCAGTTTTATGAAAACC---A

ATCAAAGCCCCCCTCAGTGGAGCCCACTCCCTGCGTCATCACCCAAAGTAGCATTTTGGC

CCAATTGCATGACATTAAAAAAGCAAGGGAGGAGTTTCTGCTCAAAAGCGGGCAGGGTCA

GTATCTAGACCCTCAGACTGGGGAGCCGCTCAACGC------------------------

--------------------------------------------------TGCGGATCCT

TCCGTAGAAAGCA--------GCCATGAGT------------------TCCAAGGAGATG

GAAGAC---ACAGAAACC-----------CTAAGCGTGGAAGACATTTCCGG--------

--------------------------------CAGCGAGG---------------AGGAC

CTAGAAAGCCT------------CCCAG---AGTCCATGCCGGAGCAGAGCGAGATG---

---------------TCAGAGGAA------------------------------------

------------------------------------------------------------

CGAGCT---------------------------------------------CCTAGATGG

GACCAAAAAGAGAAGCCTCTGGG-------TAAG---CAGCCCC----------------

------------GCAACTACCACTCCTGGAGAGCGCATAAGTTTAAGATTCTTAGCTGCC

TGGGCGCCAGTGGGAACAATGTAGCCTTTACCAGAAGATACATGCTCTTTCGCGAGGGAG

TTAACCTTCCCAACAA-CATCATTCACTACTATAA--CTCTCGCTACCGCAGCAAA----

--ACAGAAGCTC----------------------------AAGCGCA-------GACCGA

CCCCACTAAAGGGCCCA--AAGCCAGC---------------------------------

-CGCCGGTG----------------AGGACAACCACCAGACAAGGCA-GCTGCGCAACCG

CCTTTTTCCAACACTGTACGCCATCTTCCAACAAAGCAGAGGCTCCCCCACGGCA-----

----TTAAAAATAAAAAACAGATCCCTCAGATCCCTCCTAAGAA-GCTGTCTCTACCACA

AGTCAGAGGAGCAG-CTGCTGCGCACGCGAAACGACGCCGAAGCTTTGCTCAACAAATAC

TGT-----------CAAGGACTCGAGTCCGGC----------------------ACAGAC

TGA-----------------------------------------------GCAATGTCTA

AAGAAA-TACCAACCCCTTATATGTGGAGCTACCAACCGCAAACGGGACACGCCGCCGGC

GCCTCCCAGGACTA---CTCCACCCAAATGAATTGGTTTAGTGCTGGGCCATCAATGATT

AGTCAAGTTTATGGCATTAGAGACTTGCGCAACAAAGTTTTGATAACCCAGGCAGAAATA

ACCAAAACTCCCAGAACAATAATGGATCCGCCAATTTGGCCAGCT--GCCATGCTTGTTC

AGGAAGCCGCCCCACCCAAA-ACGGTCACTCTGCCCAGAAACCACACCCTAGAACAGGCT

ATGACCAACTCTGGGG--CGCAGCTAGCGGGAGGACGACA-------------------G

CTGTGCCCCTCC-CAAATAGGTATAAAAAGCCCAG--------TGCTGGCTGGCACGGGC

ATTCAGCTTAGCGA---AGACATCCCCAGCG-CCTCCTGGAT---------CAGGCCCGA

CGGCATATTCCAGC--TAGGAGGGGGGTCTCGCTCGTCCTTCAGCCCAACGCAAG---CA

TTCCTCACCCTGCAACAGGCATCCTCGACGCCGCGCGCAGGAGGCGTGGGCACCTACCAG

TTTGTG-CGCGAATTTGTGCCAGAGGTATACCTTA-ACCCTTTTTCAGGACCACCGGACA

CCTTTCCTGATC---AGTTCA------TTCCTAACTACGACATTGTAACCAACTCTGTCG

ATGGCTATGACTGAGGAGAG---C--------ATGGACCAGGTGGAGGT-GAACTGCCTG

TGTGCTCAGCATGCCCAAACCTGCACGCGCCCTCGC-TGCTTTGCAAAGGAGGGTTTATG

-TGCTAACTGGTTTTACA-------ACCCAGCACTTGCCTTTGAAGGGTTTGAT-ATTCC

AGACTCTTACCAAGAGGG-----ACACGGTGTGGACATAGAAGTTAA-----GTGTTCCC

ACCACTCCAGCAAACTGTGCCACAATGGCCATGATAT---GATCTGCTCATACTCTCGCC

TGGGATCCCA-CATTAACATAAGATGTATTTGCAACAAGCCGCGGCCCCACATGAGCCTC

A---TTGAGGCAGCCTGTTCTATGTATAACCTTAACTAGATAATAT---------TATTA

--------AACTTGT---------TTTACAGCTACCACCATAATGC---GCTTCAGCTTC

TTCATCGCCGCCGTTCTTTTCTGCAC-------CACAGGGGCCAGCAATGACAT------

------------------TGTGACTTGCTGCGCCCACACACCTTGCCTCCTACACCTAG-

--AAGTGGGCTTGGGGGCCAATG-TCAGTTGGATAAACTCTGACACAGGCCAGGCCCCGA

TTTGC---------------CTCTCCAATGGCATGTGCAACGCTACCCAGCAAGGCCTGC

AGTTTTCTGCAAACTTTTCTGAGGATGGCCTG------------------TACATCGCCC

TCATT--------------------AAGGAGAGCAACTACGAGGGCGCTGAGCACTAC-T

A---------CCTTGTCTATATT-----TATGGAGACTGCT-------------------

---------ACCAAACTGCAAA-TGAGTCTGCCCACGGGCCTA-------TTTCCAGGCC

CCTCAACGAGA---------------TGCCTCTTCCCAGCGTAACCATAAATGCT--TCC

CTCTTCTATC----CCGCCTTTCTGGAGCTGCCCCCACAGTACAGCAATGACCTTAGCAA

TGTGCGCTGGTATAAAGTAGACCCCAG-CGGCTTCCAAGCCCAAAAAATCTCTAAAGTCA

GAAGCGGAG----------GCAGAAAAGAGAACCTGCATCCCAACTGGG-----CCTTGG

TTACCTATACTGGAGACCTTCTTGTCTTGCATGTTTCGC----CAAACACCCTTGGACTG

TGGCTGGCAGCCG--TGCAGCATCGCGGG--GGGCGCACTAATTTCATTACCTTCAACAT

AACTGTACCCAACTGGCAACAAAATCT---AGTAACCATATTTAATC-------------

-------AACA---------CGAGCCCCCAAAA---AAGGGCGATAATTATGAGGACAGT

TTTATGGAATGGACTCTGTTTAAAAAG------CTCAAAAAAGGCTTATTTAGAGTAACT

TGCAGAGCCAAGT---CAATATTCCCAGAGTGCGTCCTCAACATCACCCGCGACGGAACT

TTCCTGCTTATTGGGGATAGCAAAAAGACCCCCTATGTCATCCTGCTGCCCTTTTTTGCA

AACCCCAAAGA-----------------AGACACTCCAATTTTAATGGCCCTTAGCCATT

CCATGCCCGT-CGCCATACCTGACACTGCAATGCCTATATATATTTCC------------

ATCATG---TTTTTTATTGTGGCCATGCTAGCCACCCT-CAGCCTTCTAAT------GGG

ACTAAACAA-----------------CAAA-ATCAGGCCCATGTAGCTTGTCAAATAAA-

--------CTTACCTAATTT--TTGCTAA-----GACGTCTGGGTCCTGCGTTTCTATGT

CCACCAAAGTCCCCTCTT--------------------------CCCAGCTTTGGT---A

CTTCCACTTGTGCGCGCGAGCCAGCTTGCGGATGTGC--TTGAAAGATAATGTGGTCTCT

CCCA-----------ACAGCTTCCCGTTCACCAGCACCAGGGCCA--TGAAGCGG-ACAC

GAAGAGCTCTACCTGCAAATTATGACCCTGTATATCCATACGAC----------GCCCCC

GGGTCTTCCAC----------ACAACCCCCTTTTTTTAA-------TAACAAGCAAGGTC

TCACTGAGTCACCCCCAG-GAACCCTGGCTGTCAA----------TGTTTCCCCTCCACT

AACCTTTTCTACGTTAG-----------------------GTGCCATTAAACTTTCCACA

GGTCCCGGACTCACCC--TCAACGAGGGCAAGTTACAAGCCAGCTTAGGGC-CCGGCCTC

ATCACA-AATACCGAGGGCCAAATCAC-----------TGTTGAAAATGTCAACAAGGTT

T---TGTCTTTTACCTCCCCATTACA----------TAAAAATGAA--------------

-AACACTGTATCCC---TAGCGCTAGGAGATGGGTTAGAA--------GATGAAAATGGC

ACCCTTAAAGTGACC-----------TTCCCTACTCCCC-CTCCCCCGCTACAATTCTCC

CCTCCC------CTCACAA-AAACAGGTGGTACTGTTTCC---TTGCCCCTGCAAGACTC

CATGCAAGTGACAAATGGAAAA---------------------CTGGGCGTTAAGCCTAC

CACCTACGC----------------ACCTCC--CTTGAAAAAAACTGACCAGCAAGTTAG

CCTCCAAGTAGGCTCGGGTCTCACCGTGAT--T---AACGAACAGTTGCAAGC-------

--------TGTCCAG------------CCTCCCGCAA---CCACCTACAACGAGCCTCTT

TCCAAAACTGAC--AATTCTG------------------TTTCTCTGCAAGTAGGTGCCG

GCCTTGCCGTGCAGAGCGGAGCTTTGGTGGCAACCCCT--CCCC----------------

--C----------------GCCTCTCACCTTTACATCA---CCCCTAG---AAAAAAATG

AAAACACAG-------TGTCGCTACAAGTAGGCGCGGGCTTGT----CTGTACAAAAC--

-AACGCCCTAG-------------------------------------------------

----------------------TAGCCACACCTCCCCCACCCTTAACCTTTGCCTATCCC

TTAGTAAAAAATGACAACCATGTAGCTCTAAGTGCTGGA-----AGTGG--TTT------

---------------------------------------AAGAATATCTGGAGG------

-------------CA---------GCCTCACGGTGGCCACTG-GACCTGGCCTTTCCCAT

CAAAA-----TGGAACAATAGGGGCTGTAGTAGGTGCAGGCCTCAA-----------GTT

TGAAAACAATGCCA--TTCTTGCAAAACTAGGCAACGGTCTAACCATTA--------GAG

ATGGCG----CTATTGAAGC-----AA------C-------CCAACCCCCAG--------

-----CTGCCCCCA---------------------TAACTCTGTGGACTGGGCCTGGCCC

TAGCATTAATGGCTTTATT---AATGACACTCCAGTAATTAGGTGCTTTATATGCCTAAC

CAGAGACAGCAACTTAGT-CACAGTAAATGCTA-----GCTTTGTGGGAGAGGGGGGGTA

TCGAATAGTCAGCCCTACCCAGTCACAATTTAGCCTAATTATGGAGTTTGATCAGTTTGG

ACAGCTTAT---------------GTCCACAGGAAACATTAACTCCACCACTACTTGGGG

AGAAAAGCCCTGGGGCAATAAC--ACTGTACAGCCACGCCCAAGCCACACCTGGAAACTG

TGCATGCCTAACAGAGAAGTTTA--------CTCCA---CTCCCGCCGCCACCATCTCCC

GCTGT------GGACTAGACAGCATTGCAGTCGACGGTGCCCCCAGCAGAAGTATCGACT

GCATGCTAATTATTAACAAACCAAAAGGCG---T---TGCCACTT---ACACCCTTACCT

TTAGGTTTTTAAACTTTAACAGACTAAGCG---GAGGTACCCTGTTTAAAACTG--ATGT

CTTAACCTTTACCTATGTAGGCGAAAATCAAT----------------------------

----------------AAAACCAGAAAAAAATAAG--TTTAAAA---GCTTTATTT----

-------TTCATACACGCGA----------GCGGTAAGGCTGCCGCCTTCAGGAAAAGTT

ACTCTGTA-AACAGTTCTTTCACAACAGCACAAAACATA-GGTATTAGTTAACAG-----

-----TTCATTTGGGCTATAATAATATACAT--TTTCTTGGGTGGCAAAGCAAGGGTCGG

TAATCTCAACAAAACC---ATCAAC-------------------TGGAA-TGCAAGAAT-

-----AGTCCAGCACGGTGG---GTTCA-ATCTAAAAATGAAGAAACGCGTTGAGGTTCA

CTAAGCACAGGTTTTGAATCTGTCGG-----CAGCGTCCATGCATCATAGCTTGTCTCAA

AGCAGATTGTC-TTCTTTCCTCTGCCT-TGGAAGTGGT-TTGGTGAAGC--ACTACAGG-

TGTCTTT------TCAACCTCTTTCAGCACCCGCACTATTACAGATCTCACCC--ACACA

GCACAGTTTTTAAGAGAACAATAGTTTTGAAGGCTACAAGATTTACACTTAAGCAC----

------------CAGCCAGTAATTATAAGTGCTTTTAAGAACTACCCCTAGCTCAGGGTT

AATGCACCTTTTAATGGCCTCCAT-GCAGGCTTTATGGACAGT-TCTAAAAAAAGACAGT

CTAAAATAAATGTAGTGAGTGTTTCTAAATATAATACTCCCCACATA-GTTAATTTCATC

AGGCCTGCTAGAATTTACAAACTCTCGGTACCACATATACTTTTTATTCATAGCCCC---

-------------ACCCTTAATAAAGTCCTCAATCACTTTCTGAACCACATGCTTGCTAG

CCATGCATTGTAAAGACAAGCTGTTAG--AGCAGTGACAGTGTACTCGCCACGTTTGAG-

-CCTCTGCCAGGCAGCAGTGCTTAGTTACTATCAACTCAATACCCGCATTGCATGTAAAC

CCCCCAAAGA--GCAGTTTTTCATGCCTGTGTAGCACATCATCCCACAAAATAGGAATTT

CATAGCATAAAGCAAAGCAATTACAATATTTAGGAACTCTCA------CCACAGCAGTCA

CGTGACATGTTGT---CTCAGCAGTGCAGTTGCCTTCCAT----------CCTACAA---

-TTATGAACAAAAACTAAACACTTCTAACAAAGATACAGTGA----CAATCTCCCTTCCT

CTAAAAGCATTGTTTACATTAGGGTGATTATTAACAACGTCAGAAATTTC---TTTAATT

AAAGTGCCTTTAAAAT-GTGCAA-GAGCATCATCATACTCAAAACCAAGCTGAGAG---T

AAAAGACCAC---CTTAAAAGTAATCCCA-GGCTTGTTTTTATCAACAGCCTTAAACATG

CTTTCACAAAATATAGAAGCAGTAACATCATCAATGGTGTCGAAGAGAAACTCCATAGG-

-AGACTCCAGCAT----TGATCCAAGCTCTCTAACAAAATCTTCCTCAAAATGAATAATG

CCCTTTACACA---AACGCGGGGCAGAC--GATGGTGG-GCCATCGCG---T-CAACCTG

AAACACATT--TTACA---GTAAACAAAGCTAGCTCCGCAGTGGTAAAGTCATGCCCATG

GGTGAGGCCAAAATCCTTAAAAAAGCTACCTAAGTAGTTGGT----CATCCCCTCAGTTA

AAAAGTTTTGCAGCTGGGTGGTGCATACCACATAGT------GCCAGCTTATA--GCTAC

AAAGACCTCCATCCCCTCC----TTA--GCAGACAGCTCTTGC---------ACACACGC

AGTAACTATCCACCGCTTAAGAAAAGCTTTAAGCCCAGCGCACATAACAGCTCCAATGTT

TTTATCCAAGGAGA---GCAAAATTTCAGCAAGCGCAGGCTCAACAGTAATAGTGAAGC-

---------------------------AGAGGCATTTCAGACGAGGCTCACTAGCTG---

CAG------------------------------------------------TCGCCATTT

--------------------------------------------------------ATGA

GGT--------CTGCAATA-AAAAACAA--------------------------------

-------------------------CTCATCAGCAGCTGAAAAAGTGCACTTTGACCTCA

TTAAGCCACTGCATAT---------------GCAAGTCCTCATCTATGCCGCAGCCCAGA

CCCTCAATCCAGCCC-CGAATGT-ACACTTTAATAAGAGATTCAACCTCTTCTTTTAGCA

AAGTACACATGCTGTTTGGACTAGTATACACAATAGAAGTCACAATGAGGGGCCCGCTGT

GGCTGGAAAGCCTGCGCAC-AGCCCGAAGGTTAAAAATGGACTGTAACAGCATTGAAACC

CCGCGACACAGGTCAGTCTCGCGGTCT-----------TGATCTCTTATTATAGCGACCA

AATGGTCCTTCAGAGTGATGTTGCACTCATAGAAG----TAGGCAGCTCCGGCAGC-CAT

TCTG-CAAAATAACAAAACACCACTAA-GCATAGCA---------CCATCAC-CAAGC--

ATGAAAACAG-GTAAAAACAAAAGCA--ACACT-TACTT-ATTCAGCA--GTCACAAGAA

TGTTGGGCTCC---------CAAGTGACAGACAAGCC--TAATGCAAGGTGGGCACAGTC

CTCCGGAATAAGTTGACAAAAGTCACGCCGCAAAGCTTCCTGAAGAGAAACGGCGGTAGC

CTGGATATCTGCAACGGACCCAAAACCTTCAGTGTCACTTC-CAATAAACAGATAAAACT

CTAAATAGTCCCCACTTAAAAC-CGAAACAGCCGCGG------CAAAGGTAGGACACGGA

CGCACTTCCTGAGCCC-TAATAAGGCTAAACACCACACGGCGCAGTTCAGAAGGCAAAAA

GTCTGTA-AGCTCTAGCTGAGCACACACACT--CTCCACTAGACTCTTGTGAAGCCTCAG

ACAAAAACATGCTCCCATAGACACTCCTAAAGCTGCCATT--GTACTCACGGACGGCTGG

CTGTCAGA-GGA--GAGCTATGAGGATGAAATGC---CAAG-----------CACAGC-G

TTTATATAGTCCTCAAAGTAGGGCGTGTGGAAAACGAAAAGGAATATAACGGGG------

---------CGTTTGAGGAA---GTGGTGCCAAGTACAGTCATAAAATGTG-----GGCG

CGTGGT--AAATGTTAAGTGCAGTTTCCCTTTGGCGGTTGG-----------CCCGGAAA

GTTCACAAAAAGTACAGCACGTCCTTGTCACCGTGTCAACCA-CAAAACCACAAATAGGC

ACA-----ACGCCCAAAAACCCGG-GGCGCCGGCCAAAAGTCC-----------------

-----GCGGAACTC-----GCCCTGTCGTAAA---ACCACGCCTTTGACGTCA---CTGG

ACA-TTCCCGTG---GGAACACCCTGACCAGGG-CGTGACCTGAACCTGACCGT------

CCCATGACCCCGCCCCTTGCAACACCCAAATTTAAGCCACACCTCTTTGTCCTGTATATT

ATTGATGATG--------------------------------------------------

------------------------------------------------------------

------------------------------------------------------------

------------------------------------------------------------

------------------------------------------------------------

------------------------------------------------------------

------------------------------------------------------------

------------------------------------------------------------

------------------------------------------------------------

------------------------------------------------------------

------------------------------------------------------------

------------------------------------------------------------

------------------------------------------------------------

------------------------------------------------------------

------------------------------------------------------------

------------------------------------------------------------

------------------------------------------------------------

------------------------------------------------------------

------------------------------------------------------------

------------------------------------------------------------

------------------------------------------------------------

------------------------------------------------------------

------------------------------------------------------------

------------------------------------------------------------

------------------------------------------------------------

------------------------------------------------------------

------------------------------------------------------------

------------------------------------------------------------

------------------------------------------------------------

------------------------------------------------------------

------------------------------------------------------------

------------------------------------------------------------

------------------------------------------------------------

------------------------------------------------------------

------------------------------------------------------------

------------------------------------------------------------

------------------------------------------------------------

------------------------------------------------------------

------------------------------------------------------------

------------------------------------------------------------

------------------------------------------------------------

------------------------------------------------------------

------------------------------------------------------------

------------------------------------------------------------

------------------------------------------------------------

------------------------------------------------------------

------------------------------------------------------------

------------------------------------------------------------

------------------------------------------------------------

------------------------------------------------------------

------------------------------------------------------------

------------------------------------------------------------

------------------------------------------------------------

------------------------------------------------------------

------------------------------------------------------------

------------------------------------------------------------

------------------------------------------------------------

------------------------------------------------------------

------------------------------------------------------------

------------------------------------------------------------

------------------------------------------------------------

------------------------------------------------------------

------------------------------------------------------------

------------------------------------------------------------

------------------------------------------------------------

------------------------------------------------------------

------------------------------------------------------------

------------------------------------------------------------

------------------------------------------------------------

------------------------------------------------------------

------------------------------------------------------------

------------------------------------------------------------

------------------------------------------------------------

------------------------------------------------------------

------------------------------------------------------------

------------------------------------------------------------

------------------------------------------------------------

------------------------------------------------------------

------------------------------------------------------------

------------------------------------------------------------

------------------------------------------------------------

------------------------------------------------------------

------------------------------------------------------------

------------------------------------------------------------

------------------------------------------------------------

------------------------------------------------------------

------------------------------------------------------------

------------------------------------------------------------

------------------------------------------------------------

------------------------------------------------------------

------------------------------------------------------------

------------------------------------------------------------

------------------------------------------------------------

------------------------------------------------------------

------------------------------------------------------------

------------------------------------------------------------

------------------------------------------------------------

------------------------------------------------------------

------------------------------------------------------------

------------------------------------------------------------

------------------------------------------------------------

------------------------------------------------------------

------------------------------------------------------------

------------------------------------------------------------

------------------------------------------------------------

------------------------------------------------------------

------------------------------------------------------------

------------------------------------------------------------

------------------------------------------------------------

------------------------------------------------------------

------------------------------------------------------------

------------------------------------------------------------

------------------------------------------------------------

------------------------------------------------------------

------------------------------------------------------------

------------------------------------------------------------

------------------------------------------------------------

------------------------------------------------------------

------------------------------------------------------------

------------------------------------------------------------

------------------------------------------------------------

------------------------------------------------------------

------------------------------------------------------------

------------------------------------------------------------

------------------------------------------------------------

------------------------------------------------------------

------------------------------------------------------------

------------------------------------------------------------

-----------------------------------------------------

>Ovine AdV-D

------------------------------------------------------------

------------------------------------------------------------

------------------------------------------------------------

------------------------------------------------------------

------------------------------------------------------------

------------------------------------------------------------

------------------------------------------------------------

------------------------------------------------------------

------------------------------------------------------------

------------------------------------------------------------

------------------------------------------------------------

------------------------------------------------------------

------------------------------------------------------------

------------------------------------------------------------

------------------------------------------------------------

------------------------------------------------------------

------------------------------------------------------------

------------------------------------------------------------

------------------------------------------------------------

------------------------------------------------------------

------------------------------------------------------------

------------------------------------------------------------

------------------------------------------------------------

------------------------------------------------------------

------------------------------------------------------------

------------------------------------------------------------

------------------------------------------------------------

------------------------------------------------------------

------------------------------------------------------------

------------------------------------------------------------

------------------------------------------------------------

------------------CATTCATATATATTAACATGCACCGGGGGCGTGGCGCGGCCA

TTTTGAATACTCGGCGTTTGGCGGCGGCGCGAAAATTTGAATGCTGC----GTAAGAATA

CAAGTTTTGTGTGAAAAATTTATTAAAAATGAAGTTTAAAGAATATTAAATTTGACGTAC

AGGCTTTAATTTTATTCTTATTTTAAACCTTATTGGACATTGAGGTGAGTTTTTGGTATT

TATTCTACTTTTCCCGCATTTTGCTTTTCCTTTGATTCGCTTTGTGTTTTCTTTAATGTT

CTACCACATGTTTTTTCGGGCGGT---TGGCTATAAAAATTATTTTCCAGAAATCATTTT

TTAGT-TTTTACAAGACTTTTTACCTTTGAAATGGTTTAATGA--CTGCAAGTGGGATGG

CTGGAAGTGCAATTTTCAGATGCGTTCCTGCAGATGCAGCTGCGGATAATAATGCTGCTA

AC---GTTCGCATAAAGCCTCCTCCTAGCTGTTCTTCTGCTGCACGTACTATGTCTTCCG

TTAGACGACCATTAATGTTTCTTTGGATGTTTGTTCCGAACTTAATCAGGTCCGGATCAT

ATGCAACAAGATTTGGATTGTTGTTTTTTCCCAAAAAGTACCCTAATCCGTCTAAAGCTA

GTAGTAAAATTTTACCGCCAGGAACTAAAGAAACTATAAGGCGCAGCAATCGCGTTGTTA

ACCCCCACGGACCTCCAAGTAAAGCATCTA----TTATTAGACCAAGGGTGGCGCCAAAT

AAGCT----------------TGTTAGTATTACTTTTCTAACTTGCTTAGGAATGTGCTG

TAAATATGAAATTACATCTTCAAGGCTCCA------------------------------

----------TTTGTTTTTGGCTTGATAGTCCACATCCTCTTCTCC---GTCTGGAGGTC

TTCTGTAATTAAAA----------AACTGTCTGGAAGCAATTGAGTGTTTTGGTTCTTCT

ATTTGGTGAAGTTGAACGCTTGATATGGGCATGTGTAGCACTGCTGACATGCGTCTTGGT

TTGCTGTGATATTCGTTGACTGCAGTAGGGGCCAATCTCAAGTGACTATATAAATTTGAA

TTTCGTGCGATTCTGGTTC----TTTTTTTTAGATTTACGTGACTGCGTCTGCTGTCTTT

GCGTTTCTTCGCTTTGCGTGTTCGTCTATAGGTTCTT--CTGTAGGCCCCACCCATTAAG

TCAT-TATAAAAGTCTGTTAGCATTTTGTTCTTCACTCTGTCTGATTTTCCGCGAGAAAA

GATTTCTTCGTAAAATGGATGTTCTGCAA--GCGCCGTTGATAATTAAACACATGGACTG

TGAATGGGTAGAAGAATTGCATCGCAGCAAATTTCATTTTTTTGACTGGTATGCAGTAAA

ATTTTATCAGCAGGGAACGGT----------------GTCATGGCTATCATTGCGCAATC

TAT-------------------------------------TCTTCATTGATGA-------

---------TATGTGGT-TGCCTATAGCCTTTGCTATTGCTAACAAACGG----------

-------------TTCGGTTTGGTTCATTATTATCGCGAAGGCAAAGGAAAATGTTTTGT

TGTGAATGCTGACACAAGTAATTGGAACTGGCCCTGCGAATAT---TTATATTTTTTGTG

TTCTTTTTTACATTCTAAAGATAAAAGCAGTTTTCCTGGACCGTCTCGTTGGTATATAGA

AGAGGCATGAAGCTGGTTTTATCTCAGGAGGACATTTCGGATTTACTTTTTGAATGTACT

CTAAGATGGTATCAACAGTATTGCAACAGAATATGGTTGAAACCTAGACTTTGGAGACTT

TCCAGATTGTTAAGATATTTTCAACAGCACGAAATGCCATCTATGATTAGTTTGGATGTT

CTTTTTCAAGGAAATGTAATTTGCAAAGTTTTGT--CGCTGCGAGAGACTGTGCCTAAAC

CTTTGAAATTT----TCTCTTTTCTGCTTTTGTCTGGCTTGGTTGGGCAGACACTGCAAG

TATGAAACTCAT-----------GCAAAGACGTATCAGACTTGCATTGCGGTGGCTATTG

AATTACAAAAACTTTTGGATTATGTAACCTGAAAGGTTTTTTCTTTCTTTTTAAAGATGC

TAAAACATGGAATGTTTATCATCCGCTGATTGTCCTTTTGTATCTAATCCAATTGTTACC

T-GGCCGCAAGCCGTTCATCAAAATGTGGCCCCATCTACTGCTCGTTCGCATATATTACT

GCC-TGTGGATGCTGATCCAGAAGTATATTTCAACAAGTATTCTGCTGTATATCTAATAC

CGGACGCTACTTACACATGGAAGAATGTGGAAATTACTACTCATATACATATATATGGAC

AGGGAGCTACAGTTAAACTGCATGGAAGTGGACCTAT-ACTTTCTA--TACGGA-ACGCT

CAGATCGAA-----CCGAA----AGTTTTAAGGGTGT---TAATTTCAGA---CATACAC

TTTATAGGTGGCGAATTGCCTAACAGAAATTCTTCTATGGGGTCTGACTGTCT-ACATCA

TGGTGCTATTTTTTGTCATAATGTTTGGAAGACTACAATTTCAAACTGTGTTTTTGAAAA

TTTTAAAGGTGCTGCTGTGTGGTATTCAAGTGTTCCTTATTCTAATTTTAGAAAATGGGG

ACAACAGCATTGCATTAC-----AAGTTGTAAATTTGACTCCTGTCGTATTGGTGTTGCA

AATA-CTAGTTATTCTGAATTTTCG--ATGGCAAATAATAATCATTTTTTTGATTGTCA-

AGTGTGCTTCAATGTTGTGGGAGGATATTGGATGAGAAATGATAATTTATTTGTTCAATG

CAGGTGTGCTTATTTACATACTAAGAAGC---------ATATGTGGTATTCAGGAG----

-----ATGGGCAAAATTTTCAAGCTGCTCGGGGCACTTTCAA-------------TAACA

ATATG---------------TTAAATCACTGTGATTACGGTTCAATGTGGCCGACGGAAT

TTGTATT-AGCGA-ATGGGTCTGTGATTGAAC-TTGCTGGGTTTTATTTTG--ATGATGA

TGAAGAACAGCCTCCTACATTCGTAGGAAACATGTTATGGTATGGAGAT--------GTT

AAGATTT-TAAAACTACCAATGGCTCGTT-TAAAACATTGGTGTATAAA-----------

-----------------TGCTTGTCATATCTATGGAATTACTTATGGAATGCCAGATGC-

---------TGGATGTATAACC-ATATCCCCAGAATTAGCAGATATTGTATTTGTTATTG

GATGTTCTGGCAATAATGTTGTTATTTATAATGTTAATGAGG-------AACACATTGTA

CCCAGTGTTGGCATAGTTAAACAAAAAAATTTACAACACGCATAAAACTTTAATAAAATC

TTTTATTTTTACTCAAAATAAAAAGGGATTGC----ATTTATTTTTTTAATATATTGCAT

CTGTGTCTGAGACCTTTTTCGAAATACTCTTCGAATTTCCAGACATGAATTGTAAAACAA

AGTTTGCAAATTCATAAACATTGGTTTAACTTGGTCTGATG-GTGAATAATAACTCCATC

TAAAAGATTCTGAAACAGGCACATTATTATAAATTAACCAGCTATACTTTGAGTTTAATC

TAGCATGGTTTAGAATATCTTTTACAACAGGAATTAGAGCTGACGGAAAACCAAATGAAT

AATTTTTTATAAACCTGTGTACTTGACTGATTTCAAGCTGTGGACTTATTACATGGCACT

TCGATTGAATTTTTAAATCTTTAATGTTTCCTCTAT---CGTGCCTAGGATTCATATTGT

GTAAAACGACTATAACGGTATAGCCGCTACATTTTGGAAATCTGCCAGAAATTTTAGATG

GCAAGGCATGAAAAAATGAACTGATTGAATGACAGCTGCCTAAAAAGTTCATACATTCAT

CCATAACAATACAAATAGGTCCATTGCTTGCCGCTT-TGCAAAATATGTTTTCAGGGTTG

TCTATATTTAAGTTTATGTCTGATACAGCGTCTTTAAAAGGTAAAAGAATAAAGGTAGGA

TTAAGTTTTGTAGTAAGTGGGT------TTCCTTGAGCAT------CATAGTTCCCTTCA

GCACAATGAGCTTCCCAAGCTACTTTTTCTTCTAATGGCACCGTTCCTTTTTCTGGTGTT

ATAAAAAATATGGTTTCTGGACTTGGTTCTATCAATCTTGATGATATAATGTTTCTTATA

AATTGTGATTTTCCACTTCCAGTTGGACCGTAAACAGTAACTATAAAAGGCTGAATTCCA

TAGTTTAGACTTTTCATACAGCCGT--TTG-TGAGC------------------ATAGCG

TGTGCTTCGTTAACGGTTTTTTG-ATATTTA---ATAGCCGTGTTGTATAAATCGTT---

-----------CATC-AGTTGAGCATGATAT-----------------------------

AAACA-TTTTTCAAAAA-TCTCTTCTGGATCTGG--AAATGGATC----TTTACAAATA-

----GAAGGATCTATTT-----TTGTTACATTTTCTTTCCATTGTAAAGC-------TTC

AGTTATGTATCGT-CCC----AGAAAGGATTTTCTGTCA----------AGC--ACTCTT

GTGG-TCTTG--------GGTTTGGGTGTCTCTTGTCGTATGGGT--AAAGCAAATATCC

TTCGCTCAACTGGA---------TCCCCTTTCTTAATGTCATGTCTTTCCAGGGTCTCAA

TATTCGAAGGAGTTGTTTTTCCACTACAGTGAATGGTTTGGAAATTTCGTTCCCTGTCTG

CAGAGTTCTTTTCAAACTCTTCCTTTTTGTCATGAAGTTCTC-CT-----TCGGGTTTTC

TAACAGATAATAATCATT---AAAACAACGTTTTAGAACA---TCATATGACAGACATTC

TTTAGCATGCCCCTTTGCTCTGAGTTTTCCTGAACCTATG------TGATTACACTTTGT

ACATTTAATTTCTTTCAAACCATATAATTTTGGAGCCAAGAAACAACTTTCTGTTGCAAA

AGCTACACTGTG-ACACTCCTCACAAACTGTTTCGCATTCGACTAACCACTGTAATTGTG

GTTCATTTTCATCAAAGACTAATTTGCCATTATTATGTTTTAAGCGGTGCGCTCCCTTTG

AACACATTAGTTCATGACCTTTTTGAGATAAAAACAGTGAGTCAGTATCCCCATATAGAC

TTTTTAAGTCTCTTTCACTATAGC---TTTTCCCACGGTCTTCCCCGTAAAGAATATCAG

CCCACTCACTCATGAATGCTCTCGTCCATGCCAGAACAAATGAAGCGATTTGAGTCGGAT

ACCGGTTGTTCTTGATCCATTCTGCTTTG--TCTTCGATCGTT----GTCAAAACTAAGT

CATT-ACATTCAGCGGAGAGAAAAGTT-ATA---GGCTTAAAAATCACGT--------GA

TCATTTTTC-----------TCTATAAAAGCCGTTTTTCCTGATTTTTCATTCAAATTTT

TGTTGGTGGTGCTGCA--CACTTCAG-----------GTAGGTTTAAAAAATAC------

-----------------------------------------------------TTATTCC

AATCATAGTCTGTGGACTTT------GGTAAAG--ATGAACTAATCACTGTTGT------

------------------ATAT---------------GAAGTAA----------------

TTTCACTTTTTCCTTCTCTAAGTAATTTTTTGTCT---TTTTCTTCAATATCCTCAGCAA

ATATTACTTTTTTTTTATCTATTTTTGTTGCAAAGGAACCATATAGAGCATTTGATAGCA

GCTTACTAATGCTTCTTTGAGTTTTATTTTTTTCTTTATCTGCTCTTTCTTTAGCTGCTA

TATTGATCTCTACGTATTGTTTACATAATGTTTTCCAATTAGGCCATACTGCATACAACT

CATTTTTCATGATTTTACATTTCCAACCTCTATTGTGTAACGTTATCAAATCTATACTTG

TTACAGTTTCATTTATCAGCGGCTCATTTGTCCAACATAGTTTTCCACTTTTTTTTGAGC

ATAGCGGCGGCAACACATCTAAGTATTCTAATGTGGGAGGCTCACAGTCAGCCACAACTA

TCATAGCTTTAATT-CTAGGATCAAAGTATGATAAAACCTCTGAA---GAGTCTAACATA

TTTTGC---ATCTCATCTATAGAAGTATTCGCCTCAAAAGGGTTAAGTGTTCTACCATAT

GGCATTGGATGTGTTAAAGCACTTGCATACATTCCACAAATGTCATACACATATACTGGT

TCAGTATATATGCCTAAAAATGTGGGATAACACCGTCCTCCTCTCACACTGGCTCTTACA

AATGAGTACATTTTTTCTGATGGAGCTTCAAGA---------TTGTTTAAATATTCTGAA

TTTATGTTTTCTTCCCGAT-----------------------------------------

----------------------------AATGCAACTGTTTAAATAGCATTTGAGTGTTA

CTGCTGATGGTTGGTCTCTGAAATATATTAAACAAACA---AGGTAATTTTAGTGTAGAA

TTACAAAATTCTTGATATCCGTCTATTAATTTAGATACCAATTGTGCTGTAACCTTTACA

TCATCTATGCAATATTTTTTTGCTTCTTCTATGATATTGTATTGT---------------

------------------------CCATTAGGTT-------TATTCTG--------TTCA

TATTCTGCTTTGTCTTTCCAGTATTTCTCTACAGGATATCCATT---TTTGTCTTTT---

TCATAGTCTCCTAACATAAAATAATCATTTATTGCATCATAAGGGCAATGACCTTTTGAT

ACATTTAATTGATAAGCAGCAGCAGCGTTGCGTAAAGAACA---ATGTGTAAGAAGAAAT

GTGTCTCTAACCATAAATTTTA--------------GCCCTTGCCAGGGTAAAT-CTTCT

GGTAGCAGAATGCCTTT---TTTCCATCTTTCGAACGTGGTTTTATCTGGTTTTTTGTAA

CAT---GGATTAGGAAGGGACAATGTCACATCGTTAAATAACAATTTTCCTGCTCTGGGC

ATAAAACTTCTTGAAAAAT-TAAACATGGCTA---AATTTTCTTGGTCCCTTATACCTTC

TAACACATGACTTGCAAGAACGATCTCATCAAAACCACATATATTGTGACCAATTACAAA

AATTTCAACAT-----ACTGAGGAT----AAGCTAAT-----TTCAACTTT---TTTTCT

TT-----ATTTAGTTTTAAAATCTCA-TCATAATT--------TA--ATAAT--GTTTCA

ATTTCCT-GTTCTTTGGAG---AATCGTAACCAA--ATGTTTTTGGCTACCGTTTTCTGT

ATCATAATTCTGAATTTCTTAAAAGCATTTCCTATGACATCATGTCTGTGGTTTA---AC

ATAAAAAAACAACCCCTTTCCATCATAAATCCACAGTCATAAGCAATCTGTGCAGCAATT

TTTTGCAATGTTTTA---GACCCAATAAGTTCAAACACTATTAAATAAGGAAG-------

--TAATTGTTTTCCATACGTGCTGTGGTAAGTATATGTTTCTATATCATACACTATAAAA

AGCCTTTTTGTGGATTCCAGTGCACCTACTGGGTTGAACTTTATCTGCTCCCACCAGTGC

TTTGTTTCATGATTAATAAAATGGTAATAAAAATCCCTCCTTTTTACATTACAGGTATGA

ATACTATTAAAAGTCACTCCACAATTTTCACACTTTTGT---TGTTT------TTTAATA

------TCTTTAA-----CTAA---GAACCA---------GGCATTTT------------

----------------------TATACTGTACTAATTCTACTGCCATTGTTTT-------

--------------------------TCCATCAGTTTTACATTCATCATAATATAAAAAT

TTT--TTGTTCAGTTTCCATATTTTTATTACAGATGGATATTC--ATCTTCTGAAAAATA

TTGTAA----TATATCTTCTGGTTTCAAGAAACTTTTA---TATT---GTGCTTTA-GGC

T------GCAGTCCTTGAAG---CTGAAGAAAGTTAAAAAAGCTTTTTTTAAAATCTTTA

T----AATAAATG--ATTTTTATAGGTGTATCTTCAAAATATCCTGTTATAT--------

------------------------------------------------------------

-------------------------------------AATGTGTAGTTCTGTG----TTT

GACTATGGTCT--TTGAAGCC-----ATCTTGCTAAAGCCGCTTGT-------CTTGCTC

TTTCAAAGCTTCTTAGGACGATTGGATTTGTGCTGTAAGCAACAATACCAGACAATTTCA

GTCTAAATGCTAAATCAACAGAATCAAGTTC-TGTAAATGGAGTATTTATCTGAGATAAA

ATTTCATCCACTGTGCCACTATTTTCAACAAATTGAATCTCTTGCAACAATTGGTCTCTT

TCCTCTGGAGAAGTGAAATTGATATCTTTGCCGGCAGCATCAATTATTCCAATTATGTCT

TTTACAATCCTTTTATACAGAGTTTTTAA------AGCTCTACTTTCTCTATTAAACCAC

ACCCTAGTAAAAATTTCTTCTCCTTGATTGTTCCTACCTCTTAAAATTACCTGTGCAAAA

CGTATTCCAATGTTTCTATTAGCTAATCTGTTTTGGACAAAATGGGCATGCAAATAATAT

AAAGTGCTGGCTATGTGCTCTATAATGAAAAAATACATCAGCCACTTATGAATTAATTCT

TCTGATAGCTCATTTCGGTTATAATATCCAATTAATAATTCAAAAAAGTCTCTGCCATAA

TCAAAGAAGTCGCTTCTCCTGGCTTCTGGTGTTAGTTCATCTTCTAAAGTTTGTATCAAG

TCAACAATTGTAGCTATAACTTCATCGTTAAATC-----TACTCCTACTCAGACCGCTGG

TT-TCAGGAAACTCGCGGTCTGATTCTGTTTCATCTTCCATTTCAGAAAAGACTTCAGGA

ACCCCCTCAGTTTCTGATTCTAAGGCCTCTGTGGGTCGTGTTCGAGTTCTAACAGGTAAC

CTATCTACAAATCTTCGAATAGATTGTCCTCTTTGACGCCTCATTGA---TTCAGTTACT

GCCCTTCTGTTT---TCCCTACCTCTTAGTATAAATGGAAGACCTACCCTAGTGCCAGAT

CTTAGTGTTAATGCTCCACCTTTTAGTCCCCCTTTTCCTAAAG------TTAAAACTGTT

GCTAGATC----TTTT-------AATGAGATATTATAA-------------TCATTTCTA

------GGTAGTTCCAATTTACTAAATTCTT-CAATAAAAGAGGATAACCAAAAGTTTGA

AAAGGGTAAGT--TTAGGATGTTTTCTGCATTTTC-------------------------

---------------------------------------ATTTTTGCTTAAAACTAAAAA

ATTGCACAAGGCTTTTTTGATTTTGCATATAGTATTTAAGATCTGTGCATCCCTATTTTC

TGCTCCGCTTACTCTGAAAGATT---GGGAAAATGGAAGG--------------------

-------TGG---TTTACTTCTAAATTATAACTCCTTCTTTCATTTTGTT----------

------------------------------------------------------------

-------------GATTTATTGCTTGTCCAAAGCCTTGCATGTCTCCGCTTTG---AAGA

CTACTCAAAATTCTATCCATAAGTACTGCATTTTGTATTTGATTAATTGTGTTTGTCAAA

TCTTG---------ACTGCTTTCAATTGCTCTGGAATATGAGCCTGT-ATCTATTGAATA

TGAACAATCAGTAAGAATCTGCCAGGTCATTTTTCTTTCAAAACTAATTGGAGACAAATA

TAAATCATATGATATGTTACTGAAAGTTCTTTGATCAAAT-AGATAATTATTTAAAGCCT

TAGCAGAATAAAAATA-ACCTAAGAGTAAATTTGGAGGAGGTTCTCCAATAAAAGGCGCT

TGCGACGTAACAGGTCCTCCAGGACGCAAATCAAGAAGTTGTGTTACAGGATAATCAAAA

TATCTACTGGCCCACTTGATTCCTCT--TTCTGAGCTGGT--GCGGCTTTGGACAATGGA

AGGTTGATTTAAATCAGTAGTTAAACGCATGTAACG----AAGAGTATGTTGACTTTGCC

CGGTGAGCTGTTGCCATTGTTGAATCTGAAA-----------------------------

------------------------------------------------------------

------------------------------------------------------------

------------------------------------------------------------

------------------------------------------------------------

------------------------------------------------------------

------------------------------------------------------------

-----------------------AGACAAATAAAA-CATT--------------------

------------------------------------------------TGTCTATTGCT-

-----------ACAG-ATGCATCCTATTTTGCAAAATTTTCGCTCATCTTTGGAAGATT-

-------------------CATCTCATGCCATCAGCTC----------------------

----------ATCGGGCACATCTGAATTAATCGATGATAAA-------------------

-----------------------AACAAAAATTTTAATGTTGGG-ATTGCTAGCAAACAC

GATGAAGAAACCACATTAAGAGAA----ACGCAAAGAAATGATAAAATACCTAAATCAAA

GATTCATAAAATTGATGTGTTTAGAGA---------TGAAGAACCTAAAATGGCAGAAGA

AAGAGATTTAAT---CTATAATGCTTCCAATCACATAAG-------TTTAAACTTAGATA

AAAAATTAGAACCAGAAATGTT--TAAACCTGATTTCC---CTGGCA------CAAATGC

AGCACAGAGACATATTGAGGCAGCAGAAGTTCATAGAAATGGTAATCATACACGTGACTT

AGAACAATGGTGTCATGACACTTTTGTAAGTCATGCAAAGCAGCTTCTTCTGAGGCCAAA

TTTATCCCTTGGAATGACTTATTTAGATGATTTTTTACAGACATATTTAGAACATTCTAA

TTCTTCTGAATTAACTTTTCAACTAATAACTTTATTAAGTCATACAACAGAGCCC-ACAC

TAAGACGCTT-GTTGAGTAGTGTGTCTCAAAAAGATTCAAAGGGAAACTTCAAACAGCAA

TGGCTGATTGATTTAGTTACGTGTTTATACTTAATTATAAGAGATGAACAAAATATAAA-

-----TGAAAGACTTAGTGCTCTTCTAACTACAAGCAATCATTTAGCTTTATATTTTGCT

AAAAAAGCGAGTGGAGGGTTTTATCCTACTGCTGACAAGCTAGCAAAAACCCACATATAT

TTTAAAAGAATTAT--TTTAGCTGTTTTA----GCTTTAGCAGATAATATAGGATCATAT

AATAGGAATCC-TCACTGTAGACGTCCTCTTAAAAAATCAAAACTAGAAGTGGAACCAAG

TGACGACTCTTATATGTTTAGTCTAAAAGGTGC---------------------------

------------------------------------------------------------

----ACTTGAAACCCCAG------------------------------------------

------------AAAGTGACGA------AGAAGAAGAA-------------------TGG

AACAAAA-----------------------------------------------------

------CAGAACACCAAATTCACAGTGG-ACTGCAAATATAATTGAT-------------

---------------------AATATTTTAAGAAAT-CAAAAGCTGTCTAACATTGCTGA

ATTCAAAAAA--CAACCTTTAGCCAAC-AAATTAGTAGCTTTGGAAAACGCAGTAGTTCC

TGCTAGGAAAAATGAAACAGCAGATATGATTGTTTCTTTGCTGAAAGACTTAGTAAATTT

GCAAGCCTTGAGGGCAGAAGAAGTTGGTCCTATATATTCTGATTTACTTATACGTGTTCA

TAAATACAACAGCATGAATATTCAAAACAATCTGCAAAACTTAGTGAATGATATTA----

--GAGCCAGTCAATCGGATGCAATACGATCCACAGATTTAAAAAGTTTGACTAATCAAAG

TGTTTTAAATGCTTTTTTGAACTCATTACCCCCTACTGTGCAACTAGGGCAACATAATTA

CGAAGCTTTTAAGCAAACACTAAGACTGTTTGTAAATGAAGCTCGCAA---TGTTACTGT

GTTTAAATCTGGTTTTCAGACAATGTTACAGGTTAA-TATAA--CTGGAGTTCATACTGT

AAATTTAAATGATGCTTTCAAAAACTTGAATTCTTTTTGGGGAAT---AGTCATAACTGG

AGAATCAATTCCTGGTGAAATTTCTAGCAGACTTTCAGCAAATACGAGAGTTCTTCTATA

CTTCCTT-GCTCCTTTTA-CAAATGATAATACTTTTACCCCAGATACTTTCATTTCAACT

ATAATGAAGTTATATAGATTAACAGTATCAGCTTCATTAGAATTTCCTGAGGAGACAGAA

TGGGAAGTAGCAGAGAC-AGCAAATGATTTAGGGACGGATTCCCTG-----AACTTAACA

AGTACATTAGGATATCTAATTAAAAATAGAGAAGAAGAAATTTTGAATCCTAGAACTTTA

TCACCAAGACAAACACAAATTCTTCGATTTGTACAAAAGAGCTTAAGTGATAGAATTGAT

AGAAACAATGAAGACCC---AGAAGAGGCTTTACAGACACTCTCATATTCATTTGCTCCT

TCATTTTATGAGGATAACGGGGCTTTCATAAGACGTTTAATATCTTATATGGAATTTGCT

TTACATAATTCGCCAACGTATTTCAGAGAAATCTATTCAAATAAATTTTGGACCCCTCCA

GCTTCTTTCTGGACTCAGAATTATGCGGATTTTTTTTCGGAAAACCCCAGACAGATCTTT

CAAAATGCGTTCGAAACAGACCC-----------------------GTTTTTCAATGACC

GCCTTGATAGAGGAT-TTCTTGAACAAGATGAAGATTATGAAACAGCTT----------T

GCCTCCGTCAACTGCACAGCCTTCAGT-------CATTGTTGCAC---------------

------------ACAATTCAGAATATA--------------GTGCAGTGCCTGTATCACC

TTTTGATCCCCTCAGAGACAGAA----TCCGAGAAAGCATC------------------A

CCCGAGCAGT-GATTCCTCCCCTAACAGGCTATGTTGGAAAGCAAATAGGGGAGTCGATA

CTGCCTGGTAGTGGCGA-------------------------------------------

------------TGTAATTGCACCTGCAGCCTCTTTAGTAGCAGCACAGCTTTTAGATTC

CAAATATAACTCTAGTCGCGAAAGACTAAAGGAAG---------------CTGCCAAAAA

ACGCCATCCTAACATTAGAGAAGCGGCTGATTATCCTGAT-------------GATAAAT

C-------------AGTTGACTCAGATGACACAGTAGT----TACACCGCTTTTTGG---

AGATGGAGTTA------------GGGGTGCATGTAACAAATATTC----------CCATC

TTAAGCCCAAAGGTGGGTATAGTATTTACTGATTATTACC--------------------

------TGACGGGCCGTCATCCTTTTTAGATGCAG-AAGTATGTTCCACCTCCTCGCGTC

CTTGCG-CCAACAGATGGTCGAAACAGTATTACTTACACTCCAATTGCTTCACTGCAAGA

TACCACCAAAGTTTACTATATTGACAATAAGACATCAGATATTGAAAGTTTAAACTATTA

CAATGATCATAGTAATTTTTTCACAAATGTAATACAAAATGCAGACTTAGATTCCGCCGA

AGCTTCAACCCAAGATATAAAGCTTGATGAAAGATCACGATGGGGAGGAGAGCTAAAAAC

C--TATTTAAAAACAAACTGTCCAAATGTTTCAGAGTTTTTTAATAGTAATTCATTGAGA

GTTAAACTAATGGT------CGATAAAACTGATGA-------------------------

-----------AAAGCCTGTTTATGATTGGGTATCATTGACAGTTCCAGAGGGCAACTAC

TCTGTAGGAGAATTGATTGATGCTTTAAATAATGCCATAATAGAACATTATATGGCCGTA

GGTAGACAATTAGAGGTAGAAATTAGCGATATTGGTGTAAAATTTGATACTAGGGATTTT

TCATTAGGTTTAGATCCTATTACATCTTTGGTTACTCCTGGAAAGTACACCTATAAGGCT

TTTCATCCTGATATAATTTTGTTACCAGAATGTGGAGTTGATTTTACGTATTCTAGAATA

AATAATATTTTAGGCATTAGAAAAAAAAATCCTTACGAAAAAGGTTTTAAAATAATGTAT

GAGGATTTGACAAATGGCAATATACCACCACTTTTGGATATAGAAA--------------

---AGCTACCGAG-----------------------CATTGAACCCCTT-----------

------------------ATGAATGATG------------------AAAATGA-------

------------------------------------------------------------

------------------------------------------------------------

-----------------TGTTTCTTA-CAATGTTGTCAAAGTAACCAGC--GATCCAGAT

ACG-----------TGGGAAACTATGTATAGATC----TTGGGCTCTAAGCTATCATAG-

-----AAAAGGAGGAGCTTATTTAAACACATTATTAACTGTGCCTGATGTCACTGGCGGC

GTTGGACAAGTTTATTGGTCTTTGCCAGATACCTTCAAACCTCCTATAACATTTACAAAT

AATACTACAAATGCAGAAACCTTGCCCGTAATCGGAATGCATATGTTCCCGTTAAAAGCT

GGTTTAGTACATAATACAAATGCAGTTTATTCGCAATTACTAGAGCAGGTAAC-AAACCG

--CACAGCTGTATACAACAGATTTCCACAAAATGCTATTTTAATGCAGCCTCCATACA-A

TACAGTAACATGGATAAGTGAAAATATCCCCTTTGTTGCAGATCACGGAATTCAGCCTGT

AAAAAATACTCTAACAGGTGTTCAAAGAGTAACTATAACGGACGATAGAAGGCGGCCCTG

TCCATACATTCAAAAATCACTAGCGACTGTCGTGCCAAAAGTGCTGTCGAGCGCTACTCT

CCAGTA------------------------------------------------------

---------A-------ACTGGCAGATATAGCGGGTCCTTACCCTCCGGGTTCT---GCA

A-------------------TGTC---AATTTTAATGAGCCCGTCAGAT---AATACA--

-GGCTGGGGAAGCATTGGTACAGCTCTTATGAGAGC------------------------

------------------------------------------------------------

------------------------------------------------------------

------------------------------------------------------------

------------------------------------------------------------

------------------------------------------------------------

------------------------------------------------------------

------------------------------------------------------------

------------------------------------------------------------

------------------------------------------------------------

------------------------------------------------------------

------------------------------------------------------------

------------------------------------------------------------

------------------------------------------------------------

------------------------------------------------------------

------------------------------------------------------------

------------------------------------------------------------

-----------------------------------CACTGGAGTT--AAATTTTCTAAAA

GACAACCAGTTAGAGTTAGACCATACTATAGAGCT-CAATGGGGTCAATTGAATGCTAGA

ACAC---CTTTAGCAAAACTGAGAGAAAATTTAAAAAAGTATG----AAAGAAAAC--AC

CGAAAGAGGCAGAAAAAAACCCAGAC-AGTTTTTGTAGAAGA------------------

--------------TTCCCCTCCTCCTAGAAGACG---TCGAAGAGAAAATAAAACACGA

CGCA--------------------------------------------------------

-----------------------------------------------TAGAAAAATACTT

AGATG-----------CTATTGCTAA----------------AATTAGAGCTTTTAATAG

AGCAAGAAGAAGAGCTGCTATTTAGTA---------------------------------

---------------T--------------------------------------------

---------------------------------GCTTTTCTTTTATTTCAGATG------

------------------------------------------------------------

------------------------------------------------------------

------------------------------------------------------------

------------------------------------------------------------

------------------------------------------------------------

------------------------------------------------------------

-------------------------------AAGGTAG----------TTCATGTGCTTA

AACCTT----------------------------------CTC----GACGCAGA-----

--------------------------------------------------------AAAC

GTTCAACGCGA-------------------------------------------------

------------------------------------------------------------

---------AAAAAAATTTTTTTAAAGCGTTATTCT------------------------

---------ACCCCAATTGTTTTG-AACAAAGA----ATTGACAGGCGGTTTTCTTCCAG

CTTTGATTCCAATCATTGCCGCAGCAATTAGTGCGGCTCCTGCAATTGC---AGGAACAG

TTATAGCTGCACAAAATGCAAAGCGTTC--------------------------------

---------------------TTAAAACTAGATTTTTTTTTTAAC---------------

------------------------------------------------------------

---AGATCACTATGGCCTTTGCAAGATTAGCTCCCCATTGCGGGCTAACACCTGTATATG

GT------CACACCGTTGGAATCTGTGATTTA---AGAGGAGGTTTCAGCTGGTCTAGTT

TAGGGAATAGTTTAAGTTCTG--GCTTAAGAAATGTAGGGTCGTTTCTATCAAAC-ACTG

CTCAAAAAATATCAAATTCTGAAGGTTTTCAACAAGCAAAACAAGGTCTTTTACAATCAG

GCGTATTAGAAAATGTGGGGAACTTGGCAGGTAGCACTCTTAGTAATTTGGTAGATATAG

GACGTATAAAAATAGAACAGGATCTGCAAAA---ACTAAAAA------ATAAAGCTTTAG

GT-------------------------------------------------GGAGAAAAT

GTTTC------------------ACAAGAACAAATCGCGCAACTTCTTGC-TGCTATAAA

--------------------TTCTTCTAAACCGATGTCTCAGACTCCT---ACTGTTAAA

CCTGT-------------------TATAAATTCTCAAGA------------TACAACTAC

CA---------------------------------CATCTCTTACTCCTGCCATACATTC

TGAA-------GAACCGTCTTCAG-------------AAT-CAATTCC------------

-----------------------CTTTCGT-----------------------CCAAGAA

AAAGAAAAC-------------------------GTGTATCCGGTTGGGGTGCTTTT-CT

GAATGACATGACCGGAGACGGCGTGAATTCTGTTACGAGAAGATATTGTTATTAA-----

-----------------------------------------------------AAAC---

--------------------------------------------------------ATTT

TTTGTTTACAGATGGA-GCCACAGCGTGAATTTTTTCACATTGCGGGTAGAAATGCGAGG

GAATATCTGTCTGAAAATCTGGTGCAGTTCATCACTGCCACACAAAGCTTTTTTAATCTT

GGA-GAAAAATTTAGAGATCCTTTTGTAGCTCCAACATCTGGAGTCACTACCGACCGGTC

TCAAAAACTTCAACTTCGTATTGTGCCGATACAAGTAGAAGATAATGAAAACTTTTTTAA

AGCTAGATTTACCCTAAACGTTGGTGATAATCGGATAGCTGATTTAGGAAGCGCATTTTT

TGATATAGAAGGATTTATTGACCGCGGTCCTTCTTTTAAGCCTTATGGAGGAACAGCATA

TAATCCATTAGCTCCTAAATCTGCTTTGCCCAACATGGC-------------------TT

TTAAAAACAATGAAG---------------------------------------------

---AAACAACATATATAGC-------------TCAGCTTCCACA-AATTTATGCAGCAGA

AGACAAAGGCTTAGAAACAAGCCAGTTGGTATTCCTGTTTC-------------------

------CGGCTACTAAACCTAATCCTCAAGAAGGTAGAAGTGATGAAGGAATTATAATGG

CCCTAATGATATTGGATACAAAAAAA-----TTTGGCGCTT-ATGGACGTCTTATTGGTG

CTGACAGTCAAGGGAAAAT---TTT---TCCAGCTTATGGATCATATGTTAAGCCCGTTA

GTGTAGAAGGAAGTGTTTCTA------------------CTGCCGA--------------

----------------TGT-ACAA-AGAGTTTATT---------------TAAA-TACTA

CAGATGTTAATGATC--GAGTAT-CTGGAGTACTTGCAGTTGATACCGTACAAAGATTAA

ACCCCGATTGTCATTATGCCGAATATACAAATGAGGTTAAAATTAC--------------

---ATCTAGT---GGA----------AACAGACCCAACTATATTGGATTTAGAGATAATT

TTGTGGGTCTTATGTATTATAACAATGGTTCAAATGCTGGAACTTTTCCTTCTCAAACCC

AACAGCTAAATGTAGTCCTAGACTTAAATGATAGAAATAGTGAATTAAGTTATCAATATT

TAATTGCTGAAATTTCCAGCAGATACAAGCATTTTGCTTTATGGAATCAGGCAGTTGATA

CTTATGATGAAAATGTAAGAATAATCCATAATGAAGGTTATGAGGAAGGACCACCAGTCC

TGTCTTTTCCTCCGTTTGGCATTCAGAATTTCTTTTCTG--ATACTGATGAGGGAACTGC

AA--------TGACAATCC----------AAGAGGGAAACCAACAAGCACAAAAGTTAAC

AATACAAAGCTTGT-GGTTATGGAA-TATTCCAACTCTAGAAATGAATTTAGCAGCTAAC

CTACAGAGAACTTTTTTGTGGGCTAATGTTGCAATGTACCTTCCAGATAATCTAAAAAG-

------AACACCACCAAATGTTGATCTT--CCAGCAAATAAAAA---TACTT---ATGGT

TATATGAATGGAAGAATACCATATCCAAACGTTATAGATACATGGACAAATATTGGAGCC

AGGTGGTCTTTAGATATTA-TGGATACAATTAACCCTTTTAATCATCATAGAAATACAGG

CCTTAAGTATAGATCACAGTTGTTAGGAAATGGTAGATTCAGTAGATTTCACATTCAAGT

TCCACAGAAGTTTTTTGCAATTAAAAACTTACTTCTGCTACCTGGAACTTATAATTATGA

GTGGTATTTTAGGAAAGATCCAAATATGATTTTTCAATCTTCAATTGGAAATGATTTAAG

AGCAGATGGAGCATATATAAATTATTCCAATATAAATCTATATGTTTCTTTTTTCCCAAT

GAATTATGACACTGTCAGCGAACTTGAATTGATGCTAAGAAATGCTACTAATGATCAAAA

TTTTGCAGACTATTTGGGAGCTGTTAACAATTTGTATCAGATTCCTCCCAATACTTCTAC

GGTTGTTGTTAATGTTCCGGATAGATCTTGGGGCGCTTTTAGAGGTTGGAGCTTTAACAG

AGTTAAAGCATCGGAGACTCCCATGATAGGAGCTACAAAAGATCCCAACTTTTTATATTC

TGGAAGCATTCCATATCTAGATGGCACGTTCTATTTAACACATACGTTTCAACGCGTTTC

AATCCAGTGGGATTCTAGCGTGCCATGGCCCGGGGATGATAGGCTTCTTATACCAAATTG

GTTTGAAATCAAGAGAGATTATTCAATTGATGCCGAAGCTACCAATATGAGTCAATGTAA

TATTACAAAAGACTGGTATATGATACAAATGGCTGCTAATTATAATCAAGCCTATCAAGG

TTACAAACTTCC----AATAAG--TCATAAATATTATGGTTTTCTTGAAAATTTTCAACC

AATGAGTCGTCAAGTACCTATCTATG---GAGGGGATTTATATGATTTATATACAGAA--

TATATCAAAAATCCAAAT---------------TTGATGCAA-ATTTGGAATAAC-TGTG

GTTTGCAACAAAAAACTTCAGGTATTCCTTTACTAGAAAATTCTGGTCATTTGTATGTAG

CTAACTGGCCTTATCCACTAATTGGAAAGCATGCTATT---------GAAAGTCAAAAAC

TGCAA--AAATTTTTATGTGATAAATACATGTGGCAAATACCATTTTCTAGTAATTTTTT

AAATATGGGAAATTTGACAGATTTAGGACAAAATGTTTTATATGCCAATGCTAGCCATT-

CGCTTAACATGGTGTTTACTGTAGATCCAATGCAAGAAACTACATACCTTATGTTGCTTT

TTGGTGTCTTTGATCAAGTGGTAATTAATCAGCCAACTCGCAGTGGCATTAGTGTTGCTT

ATCTACGCCTCCCTTTTTCAGCTGGTAGTGCTACGACATGAG------------------

------------------------------------------------------------

-----------------------------------------------------CGGA---

--------------------------------------------ACATCAGAAAGTGAAT

TGAAACATTTGCTTTCATCGCTTCATTTAACTTACGGTTTTTTAGGAACTTTTGATTGTC

GATTTCCAGGATTTTTGC-AAAAAAATAAAGTTCAGACAGCTATAGTGAATACTGGACCT

AGAGAAAAAGGCGGTGTTCATTGGGTTGCGATGGCTTGGGATCCTATATATTATAAAATG

TACATATTTGACCCATTAGGATGGAAAGAATCTCAGTTACAATCCCTTTATAACTATTCT

TATCAATCAATGTTAAAAAGATCGGCTTTGACTGAAAGT---GAGCGGTGCATTACAGTA

GAAAAAAATACACAAAGCGTACAATGTACGTGTTCAGGAGCATGTGGTTTATTTTGTGTG

TTCTTTTTATATTGTTTTTATAAGTACAGAGGAAAAGCTTTTAATAATGAATTGTTTCAA

TCCCT---TAATGGCGCTTC-ACCTTC----TCTGACTCCT--TCCGACCCTT-CATCTT

TACATAAAAATCAAGATATTTTATATGACTTTTTTATATGTAAAAGTAGTTATTTTCGAC

ATAACAAAAAAATGTTAATCAGTAATACAAAACTTGGTTTAATAAAATCACATTGAAAAC

ACTTTAATCGTCTTCATCAT---CTTCTTCGCTGCTCGATTC---CAAAACAGTACGTTT

TTTCTTTTTTGGT-----------GGAGCTTCCTCCACTTCTCCAAAAGGATT--TGCTT

CGACATCTTCT-----------TCAATTGAG----------------GGC-AACACTGTA

TTTTTTACTTGAAA----ACATTGATTCCATTTAAATTGAGGAAAGTTAATAGGAAGG--

----GGAGTTCCCATACATTCAA---ACCAG-----AATTTCCGCACAAGAGTTAAACAT

TGAAGCATATCTGGTACACTTATCTTCATTTCACAAAATTTAGTTGTGTTTCTCT-----

-------TTGAAGTAAAT------TGAAAGTTACAGCATTGAAACACAAAAACTGCAGGA

TACATTACAGCGGCAGCTTGAACTTTAGAAACATC---TTGCGCGTCAATT---CCCTCC

ACGC--CAGT----AATAGTATATGCA---GTAATCTTAG----------GTAATT---G

TTTTCCAATA------------------------ACAAGTTTTCCA---CCATAATTA--

CATTCA-----CACTGCCCTAATAT-AAAAAGCATATTGCCAATC---------TTAGCA

TTCGGAAAAACAGCTTTTGTTGTT-------TGAAAGGCATTTTGCATTGCAATTAAAGC

CTTTTTTTC----ATCTGAAAAATTTAATCCACAGCTTCTA--GGA-------GAGCACT

GCCCAAAAC--GTTGT-----TGGGCGTCTTCCGAACATAACATAAAATTCTCT-TGA--

-ACTACTTTTA--CAACCTGTTTG-----------------TTCATTTTGCCATTACACA

GAATTCCTCTCCCTTCTTT-----TAAAGCTTGTATTCCC-----ATTTCGGAAG-----

----TAGGACTCATTTCAATTTCATTTACTTTTTTAAG--------------CATAAAAT

CCCCATGAAAACAACGTGGT------------CTTTC---------ATCCCAATCATGAT

----------ACCACA----AATAGCAA-----CCAGTAACATTAAAGT-----------

----TTGGCACTAAGCCAATT--TGCTTACA-----------------------------

-----------------------------------AATTGCACTGT-------------A

CAGCAT---------GCG------ACCTCCCACTATAGCCA------------T------

---TGATTTAAAATTAC--TATA-------GGTAA--GATT-------------------

----------AA------TCGACTTAT--------GTTTTT--TTAAATACTGTGAAAAC

A--ATTTTTCACAATCTCCTTCTTCAGGA-------------------------------

------------------------------------------------------------

------------------------------------------------------------

------------TGAAA---------------------TTTCATTTGAGAAGTTTCT---

------ACTTTTACATATTCCCCAAATTTAGACA-----------CAATTTCTAAAGCCG

CTTGTA-------------TTTTT-TGCTGTGTTTCTTCA------------TT---TTC

------------------------------------------------------------

------------------------------------------AGATCTTC----------

---------CTTTCCTAGCGAGTTTTGA--------------------------------

------------------------------------------------------------

---------------------------TCC------------------------CTTTTT

AGTAGAC----------------------------ATTTTAGTTTT--------------

-----CTTTCGTTG------------------TAGGGATGG------CTGAAAGAAATAT

GGAAGAGTC---------------------------------------------------

--AGAGAAGGCAA--------------------------------AAAGCAGCCAT----

------------------------------------------------------------

------------------------------------------------------------

------------------------------------------------------------

------------------------------------------------------------

------------------------------------------------------------

------------------------------------------------------------

------------------------------------------------------------

----------------------------------------------------GCTTTTGA

AG-AAACTATAC------TGTCTAAACAT---CTGGAAAGACAACTGAAAATTTGTAAAA

AT-------------CTTAACCTAAACATA-GATGATTTAGATATAGGT--TCAATTTTG

GAGAAGCATCTGTTTTGTC-------------------CAAAA--------G---AAAAA

AACACT---GATAATAATCCTCATTTAAATTTCTACCCTCCGTTTCTAATACCGGAGTGT

CTAGCCCTTCACTACCCATTCTTTTTAT-CAGTTCAAATTCCTCTATCATGTAAAGCAAA

TAGATCTGGAACAAAAACTTACCAAGAATGGATGGAAATTAACAG-TTTGGCAAC--GCC

TCTTCCAAAATTGG------AAAAGTGCAAATGGGATGATAGCCTTGGAAATGTAGCTGC

TATCGAAGAACTAGA------ATCAAACCAA---------------A---GATTGATAAA

GTTAGAACAAGATTCGCTTAGACTTTTTTGGTGCAAGGAGAAAGCAAAATACTTTTCTAC

ATTTTCCTACCCTTCACTAAGTTTGCCTCCAGCTCTACAAAAAATTTTAAT---TGAA--

----------ATTTTTATTGGAA--AATCTCA---A----------GAACCTAACAATCT

TTC------AGACAAATATGAACCAGCAATAACTCAATCAATGTTAG---ATCTGCTTGG

AATA--CAGGATAAT----CTCCACGAAG------------TTCAAGAG------AGATT

GTTCCTAGCCACAACTTATGGCACTACA---CTCGTGTGTATGAAAAAATTAATCGAGCA

GAAAAAATTTATAATGAACTGTCAAGAATCACTTCATTACACTTTTAATCATGGTTTTGT

CAAACTTATCCAGATGCTTACAGACATTAACCTTAGTGAATTTGTGACCTTTCATGGTGT

GACTCACAGAAACAGACTTAATAATCCGTATCAACATTCACAGC-TCGAAAATCAAGATA

AAATGGATTTTTTGACAGACAGCATTTATTTGTTTTTAGTACTGACGTGGCAGACCGCAA

TGGATATATGGGGTCAAACTCTAGATGAAACTACCATTAATCAGATTAAGGAAAGGCTTG

ACAGTGCTGCAACAAACATCTTAAAGGCGCCTACTGCCTTTGCAGTTGCAGAG-GAAATA

GCTAGCATAATA-TTTCC--AGACATTTTATTGACAGCTTTTACAAACAATTTACCC-GA

TTTTATAAATCAAGCTCAAATTGGAAATTTTAGAAATTTTATCTGTTGCAAATCTGGGAT

TCCCCAAAGCATTTGTCCTTTTTTGCCATCAGATTTTGTGCCCTTAAGCTATATGGAATC

ACATCCTATTTTATGGACTCATGTTACTCTACTTCGCACAGCACAGTTTTTACTGCTACA

GGGAAATTACCTA-------TATACACCAGATAAACCC-----TATACCATATCAACTCT

ATA---CTGTGAGTGCAACCTATGTTCCCCTCACAGAATGCCATGTTATAATAGCAATCT

CCTGAATGAAATACTAACCATAGGCAAGTTCGAATTTCAAAAACCTGTTGA--------A

GGCCAGCCCACA----------CTTAAACTTACTCCACAGTGCTTTGCTAATGCTTATCT

TTCGAAAGTAAATGCAGAAGATTTTTTCCATGACAAGATAGTCCACTATAACAATA---A

CAAAGACAAGTTCAATCACCAATTAACTGCTTGCGTTTTGAAAGATGAAAAATTATTGGC

TACATTAACAGAAATGCAAGTAAGGCGGGAAAAAGAATTGTTAAAAAGAGGTAGCGGAGT

TTATCTTGATCCAGAATCTGGGGAGCAATTAAATGGAA--AAGCCTTT------------

----------------------------CCAAATGGAGAAAACCTATCACCCTATA--GC

CAAG-----AAAGCAAAGGGAAAAGAAATGGCGATGATGTATCACTTTCTAAAAGAGAAC

AACCTAGACGGAGAGCCTCTCAGCGAAGGCGAAGTCAGTGAAGAAGAAACAACTGATCTA

CCTCATCCTCCACCTCTGCC-AGACA---AGATAACAAGTAAGTTAAAACTTAACATTTT

TTAAAAATTTATT--------TACTTTTAAAAATTTGTCAAGATCTTAAAATCGGTCTGC

TTTTCTGCATTTTTTTTAGAAAGCTA----------------------------------

---CAGCGTACACTCTTATCAAACTCATCTACCTCTTAAACATG--------AAATAGCT

GAAGTTCTGGAAAAAATTCGACACGAGTCTAAAAAGAGAC----CAGAAAGAGTGCATCA

AATCAGAAACCGAACTCCAGCAAGTATAACAAAACGATACCTGTACGAGCGGGACGTAAA

GAAGCTTTTTCAGTATCTGGAAGATGCGAAGAAGCTTCTGGCTAAATATCAAAGCTAACC

AGGTTGCTTAAATATTCAATTTTCCCGCCATGCAGCCCGTCACGCCATACATTTGGAAAT

ACCAACCAGAAACTGGTCATACAGCTGGAGCACA---TCAAGATTATGGCTCTGTCATTA

ACTGGCTTCAGTCCAACCCTCA-----------GATGTTTAATCGAATCCGGGATATTAA

CCTTCAAAGAAACAATATAGACCAAACACA-----------------------------A

GGTCTCATGACTCGAGATGACCTACACTCTAACATAAATAATTGGCCTGCTTCCCAAGTA

CTTCAAAGGCAAGGATTTCCTTATGTGCCAGCAAAGATGAATTTAGAAGCTTCCAAACAA

GATTTTATTGACTCAGCTCGAGGGATACAATTAAGTGGACCAAATCCTGTTTTTACGGGA

ACAGGATCAAACAA-CTTAACCTCTTATCCCATGCTTACAGAA-AACATACCGCCTTTGA

TGC-----------TATATCATAGACCCGGACAACAGCTGCAGGGCATGGGAACTATATC

TAAAGAAAATTTCCATTTGTTAGAAGAAGTTTCCAGAGTACCTCG--ATCCGGAGGCTTA

ACGCCA--ACAGAATTTCTAACTCAATTTCCTCCAATTGTTTACAGGCACCCCTTTTCGA

GCAGCTT--GATT----TATTTTCCAAAAGAATTTGATCCTTTGTTCTCCCCTCACAATG

ATCCACGTCTA-ACTTCAAGTAAAACTTTGCAATACGTTCCATAAAATAAAATTGTTATT

TTATAAAACTTTACTTACATGAGCAGGTCTTTTTTTGTCTCGTCGC-CATAAAATAAAAG

CAGGGAACCATAATTTTCTA-ATTTTAGTTTCGTTTGTTTGCTTATTTTTATCCATTTAA

AAGGAGGACAATAGAA--ATCA-CAGGTTACTAATAAGCTATTAT--------TAAAATA

AACTTGTGTCAT-TTTACAGATGAAAAGAGCACGT-------------TGGGACCCTGTT

TATCCGTTCACAGAAGAAAGATTGATACCTC-TACCTCCCTT---------TATTCAAGC

AGGAAATGGGTTAG--AAAGTGAAGGTTTGATTTTATCGCTTAAATTTACTGATCCAATC

ACAATCAACCCTGGAGGTTTTTTAACTCTTAAAACTGGAGACGGCGTGCAAATTAATAAA

AATGGT-GAACTAACTACAGATGCTGCGGTACAGG--TACAGGAACC-----TCTAGAGA

AAGATTCAAACG---GAATCA------AAATAAATTTAGA-TTCTGCTTTATCTGTAGAT

TCAGCTGGAAAATTAACTGTAGCGTTAAACAATCCCTTGGAAAATACTCTAACTGGCGTT

AGGTTAAAGCTGTCAAACTTCTTTTCTATTGATGAC-TCTGGAAAATTAGTTATAACGTC

--TCCTGAACTTCCACTT-------TC-TACCACCCCGGAGGGAAAACTCTTTATACAAC

TATCAAACTGTTTTGCAACAGATAATGGAAAATTAGTCCTGACATCACCAGAGCT-ACCA

CTTTCTGTCACTCCGGAAGGAAAAATATTTTTGCAGTTGTCAAACTCTTTTCATACTGAT

ACTAATGGAAATTTAGCTCTAGCATCTCCTAACCCTCCACTCTCTGTAAATAACGAGGGA

AAAATATATTTACAACTATCAAACTCTTTTAATATTGATAATACTGGAAAGTTAGTTTTG

GCAT-CTCCAG-AACTTCCACTTTCTGTCACTTCTGAGGGAAAGCTTTTTTTAA--AACT

AGGGCAACCTCTAACA-ATAACTGACA----GCCTGTTAACCTTAAAAACAGAGAATCCT

TT--AGCAGTAACTGATGGCTTTCTAAAAATTAAACTAAGTGACCCATTTGAAGAGCTA-

--AACGGCAACCTTGCAATAAAAACAGATTACCCATTAGCAGTAACTGAAGGCTCTCTAA

AAATTAA-----------ACTAAGTGATCCATTTGAAGAGC-GAAACAGCAACCTTACAT

TAAAAACAAACTACCCCCTATCGGTTGACACAGGTTTTCTTAATATTAAATTGGCTGAAC

CCTTTGAAGTTCTAAATGATAACTTGGCACTAGGCCTTAATAATT---CATTAACAGTAG

AAAGTGG---AAAACTGGCTGTGAAAACAGCAGGCCCAATTCAGAGCACTGAACAGGGCA

TTAATCTAAGTGTTGCTAATCCATTTTCTATCTCAAGTAATCAATTATCTTTAAAACTTG

CTTTTCCTTTAACTATCAACAC--TGCTGGTGCATTAACTACAAGCACCAGACAAGGATC

TCGGGTTGTTGGC-TTTATGGACTTTATCATTGCGCTGGGTTGGCAAATAATACCCAGCA

AC--ATAAGATACATTTAT-ATTTTGAATTGTTCACAATTTATGCCAACAAGTGATGTTA

CTACTATTTA----------TTTCCAAGCTGATAGTGGTTTGGAATCTATATTTGTGATG

GATTCTCCATTTTATGCCTCATGTA--CCCAACAGCTACCTGATAAAACTATTAAAA-CT

TATGGGGTGACTATTTCCAAAAAACAAAG----CATAATTTCAATTAATTTCTCTTCGTC

TTTAGAACCCAATATTATGGTAAGTGCTTGGACTGCTAGCATAACTCGCACGC-----AA

TAA----AAAGACTCAACTTTTTATTCTGTTAAACACCTTTA-TTAACATGTCTGTGATT

CATGCTGTAAGTTCAACGTAATCTGTTTTTCAATAAACGTTTCAAATATCTTTCTTGATA

T-TCCATGCCATTAAATTTTACAAAGCGTATTAAACAAGGTTGATTTTGCAAATCAGCAG

T--TTCAGCAGACTTCCAATAACAATTGTCATAGGGAGGCAGATGAAAACCTCTCAAGT-

-CTGGCAAATTTAATT---GAAAGTGTAACCTAGCCAAAGTTTCTCTCAACTTTACAATG

TTCTCTCTTATTATGAAAATACTTTTAAAATTTCCAAAGTTT-ATTCTCCTATAACTGTG

AGATTT-TACAATCTCTTCCTCAACATGTAAGGCATTC-CAAAATCTACAAAACACAATG

TATTCATTTACAACTTGATAGAATATTACATTTGAACAACAATTTGTCCAAGGAAGTACA

AATGGAATCTTTCTTTTTACATTTTTAAGCTTGTCTAATAAAAGCTGTTTAACACATAAA

CATTGAAGTGATGTGGGATCAGTACAATGACAAT----GAATTTTGTACATTTGACAAGG

AAGTGA-ACAAAGGTGAGTATATATTAACTTAAACTC--TATATGATCACATAAGCTTTG

AATTACATTAATAGAAAAGAATG----GAATATCCACCCTCAACAGAAAATGGGAATCTT

CACAATAACAA---GTAGAATTCACAAACATTTTACAGATGCCAAC-CAGATTCATTTGA

AGGCCTAAAAAGAAAAGTACAAGAATATAACA-TTCTGACAA--AATTGTATCTAACTTC

AGTCCTTCTTATACACTCCTTCA-TTGAAAATTTACATTTACATCTAACTACAATCATAC

TACAAGAATCACAGAAAGCAATC-------TCTTTTAAATG--CTGATAATTGAACCACT

CATTTTTAAATGATTGA-CCATTAGGTCCAC-ATAGTAGAAAATAACAATGCTTTTGTTT

CATATTAAATGCTCCAAGGAATATTATAGGACAACAAACCCATGTGTTGC-AACATACTG

GAACTGTCCTTAAAACTCTCTCAGAAAATAAATTTACAGGTACATTGTCAAAACATT--T

TAATAATACTTGCT-TAGCTGC-TAAACACTG--TAGAG----ACCCAGGAGAAGAACAA

TG-ACAATGATAAAAAAATTTGTA--GTCCATATCACTTTGCT-TAAAAAAAGAATCACG

TTTTTGGCTTACTGGAAAA-TAGCAAAAGTATTGATCATATATAACAGGAGTAAGCATCT

TCTTTAAGCAGGATACCCTTAAAGCAGGACAAAGTCGACCCCGTCCGTGACAACGAAACA

CTTCCATGCCATGGC--TACTTATATGAGGCTTATCGCAAGCAATGAGCTGTATATTGAG

TTCCATCACTCCCAATCTGGTGCAATTGGA--AAATAAGGAAAGTAATTTTCCCAATAAT

ATTTGAAAAATTCCCAACT----TTCATGACTTGTATTTCCAGGTATTAGTATTACTTCA

GCTGTTAGCATGGGTCTATCTA-----------------CCAAAGAATTAATTAAAAGTT

CCCATTCTATTGCAAAAAATCCCATGCCCATATTTGAAAAATTAGCCCACTTTAAAAAAT

CTAATGCATCAAA------CTCCGGAAACAGATCTTTCTGAGCCAACAAATACACAGTTG

AATCCCCTATA-AATCTA--AAACCCATCCTAAAAGGACCCTTTGCCCAATAAGCTAAAG

ACTTGGAAGTTGCAACACAATATATTGCATACTGATTAGAATTTAAATCAGTAAAGTAAT

TAACCAA-------TTCTGCAGCCCCCATAAAAGTTCTGGAAAGTTGATTCATCTAAAAA

ACAAAGAAACATATTTTTTACTAACCATTACAAAATATCT-TAAAAAATTTCTTGTATGT

AAAAGCACTTACT-TGGAGCTTCCTTAA-ATACAAAACAACAGGCAACTGAAAAATTAAA

AGCAT--------------AAAATTAAAGACAGTTAATAAAAAATTTTTCACATAAGAAT

TAAACTGTCTTCACTTACCTTAAAAGCTTATCAGAAGTAACCTGCAATTACACAAAAATA

ATAAATTATTAAGACATTCTTAAAGCATTTTACAACAAATCCAATGACCTTCTTCCTTAC

CTTGCC---TTTGCTGATAAAAATGGTGCAGATTCTAAACGAAAACCTCTTTATATATGT

ATAAGCTGTTTTTAATTAAAATGCATTAAAA----CTAGTCAAACAAAAACTTTTCCTGG

TAAAGCTACCTTACAAAAAGTTCACTGCACAGAGAACCTTCAGCTATGCTTTTTAAAATT

TTCCTTCTCTTCTGCAAAACTAGCAGCTCTGCTGG---CTTTTTAAACACCTAGAGCATT

TTTTCATCTCAGCTAAAAATAATTAAGAATTTTTTATAAACACTCTCTAAGGACCGTTTT

CCTAAAAAA---------CATTTTTCTCACGCTAGACAAACATCTTATATTCTTTTATCT

TTATGTTGCATGCTTCTGGTCCCACAAAGACTAAACTCCCTTAATAATAGTAACATCCTG

CATCTTACTCCCTTTAAAATCCATT------ACTCCTTAACATTCTTTTCCCAAACCACT

TGATTTTTCCTGAATTTTAAAAAATTTCCCGTGATTTTATCTTATCAAGCTAAAAATATA

ACATACAGA-ATGGAGCTAACATCCTTTCAATATGCTAACCTCATT---AAAACTATAAT

ATGCTATTATAACTTTTTTAAAAATTGACGAACATCCTCTCTGTATGCATTTCCCCTCCT

TACACATTCTTCTAATGTTAAGTGTCACCTTTCCTGTTTTTAAAATAATACCTACACCTT

ATAT-CCTTTAATATGCTAACCTCATTAAATCTATTATATTCCAAAAATTGCTCACATCC

-----TCTTTGAATGTATTTT--AAGCCTTATCCTCCTTAAACAGCCCTTTATGCACAAA

AATTTGCT-CTTCCTGTTTTAATCTGATTAGAAAACTAAAAATATAAGTTTTTTGTA---

-----TGATAACCCCAAGTTAACTCGTGGGCTGACATCTTCTTTTTTGGTTGCTGTAACC

TTAGAACCCATGACTTTTCAAGTATAATTAACATTCCTATTAAGGACAAAAAGTACTCTG

CTTG-TTCAAAACATGCTTTGATATGCTTTATTAACATTTAATCTGTTGTCCTGTTTACT

TAAACTAACTTTAAT-GGAGCATATCATGATTAGGATCT-GCCACGCTTTTCAAACACAT

TA----TAAACTTGTTCA---GCAAGTTTCTTCCCATGC-TCCCCCCCCCCCCCCCCTGG

CAAGTGTAGCATGCAAACTGCCTCTTTGATGTTATTTATATATGAATAACACGAGACCAA

CAGTATAAACTTTTTTATTTTTAACTAAATTATTTGCAGCAAGCACATTTAGGCCAAGTA

TAATCACATGAGTCAAGTTCACAATCCTTATTCCAAATTATTACATCCATCTTCCAAAAC

CACAAGTGATTTACACAGAAACAGCTTACACGCAGGGGATGAT-ACAGAAGCAAATAAGG

GTGAGTCAAACAAGCACAACAATGACCAGAAGGTAAAAAGTCTCTAA-ACATACACCATG

AT-GATGGAGGAATCAAAGGCTGAAATTTTGAATGAGTCCTCTCTGCCAATAAGTTTATC

AGTTCTCCACAAAGCACCTTTTCAAATTTTGACATATACTTCCTCAGAAATGACGGCCTT

TTTAAAATATAAAATAAGTCTAAATAGC-ATGAAACAAAAGAAGCAATTTCCAGGTTTGA

TAGGTATACAAAAATTTCTTCCCATATACTTGCAGGGACATCATAAGGAAAACTTGCTGC

CTGTAAAACAATAAAAAACTCACTTTAGACAAGCACAACAGTGTAAAATCTACTTTGTTC

ATTTTCTTTTTACAAAAATACTTACAAAAAGCGGTCAGAAACGCAGCAAAACAATTTTCA

GAAAAAGGATTTTCACAACAACAACCTCCACGAAGCACATTATGCACTGTCAAATGACAT

TTAGCTGTTGGATACGTCTCTCGTGAAACAAGAGTATCTTCTTTCAAAAGACAAAAATGT

TTCTTTTCAAAATGTGTTGCAGCTAGTTTCCATTTACCAGTTAAAAGACAAAAAGGAGAT

AAACAGTAATTTGAACTGGGACTATGATGTAACATTATAGCTTTACAATAAGCATTTTCC

ATTTTACTTGAATGACAAGAAACAGTATTTGATATATAATTAGTTGTAAGTTCAGATT--

---TACACATAAACTCAAGAGCAAACTTTACACACTTTATAGAACAGGGAAATTTACATT

TACAAGTAATATTTCCCTTCAACAGTCTTTGAAAAGTGTACAGATCCATATGATAAGATA

TAGGTATTGTCAAAGATACACAATGTCCAAATGAAGTTTTGACACATGA--TAAGACAAT

TCGTGAATTCCATTTAAGTGAACAGCCAAGTTTTGTACAAAATGCCACATGTTCCTTAAA

AGCTGTTTAAGAATGGAAACCTTCATGTTTTTAACAATTGTATTCTTTGCAACGCTCATA

AGCTTTTTTCCACAGACGAAGTCTTCTGGATTCATCAA----CATAAGCACCACATTCAG

GCCAAGTCCATCCACCATCCAAATTAGGCCAAAAATTTGAGGACCACAAAATTAAGTTCG

ACAGACCCCACAAATGACAATGATCTACACAAAAACAATAAGAATGATAAGGAGACGATA

AAAGCAAATGAGGATTTAACAGACAAGCTACACAATGACCTGAGGGCAAGTAATCATCAC

AG-GCACACCACCAGGTACCCACTTTTAAAGGGGCGTGATCTTTCCTTCTTAACACACTT

CTAAGTTTATCCAATGTTACATTACAAGTTACAGAATCAAACCTGCCAATGTGCTGTCTT

AAAAAATAACTATCTAACAAAATTACGTGAAACTCTGGAAAAATTAACACAAACTTTGCA

ATTTCTGAATTGCTCAGATATAAAAAAATCTTCATCCAAATCTCTGATGGTATGGCGAAT

GGAAAGCGAGCCTAGAAAACAAAAAAGACTCAAAAATTGTAAACTTGCTATAAGACAAAA

ATTTTTTGTTTTTAAAAAAGATCAGGTTTTAAAAAACTCACCGAAGCACCTTTAATATCT

TTACACACATATTCAGAGCACCTGCAAACATGCTCTGAAAATCCTAATTTTAATAAAACA

CTGTCCATGGCAGCAGCTGGCTTCTGATAACGTAGCAGGCAAGTCAATATCTTACACGGC

CTTCCTGTTTAAAAAAAATTAGCTCGAACAATCTGATCTCTTTTAACCACGGAAAGTACT

TTTTTATACAACCTCCTTCTTTTTTTAGG------ATCTAAAACTGGAACCCCACATTCT

GGCCAACTCCACCCAGGGCTTAAATCCTGCACCGTTCCCGCTTTCCAAAGAATTACTTTA

TTTAAACCATACCATAAAAGTCTATGATCTAAACAGTGACAGCCGCTTTGAAATGGGGAA

AAACACAATAAATGTGGATGTGTTAAACAAGCCACACAGTGACCTGAAGGTAGAAACACA

CGGTCATTGCACC-----AAACAGACCACATGCTTATAGGGTTAAAATTAGCCACAGTTC

TCTCAGCTCTCAAATAATTCAAAGTCTTATTACATGAAATCCTAGAAAAGCGCTGCCAAT

ATCTCTTTAAAAAATAAGGACGAACTATTTCAGAGTACAAC-TCTGCAAAACACAGCACA

AAGCTAGCTACATCTTGAGAGTCCAAATACAGTAATATCTCGCACCACAATTCTGATGGA

AGCACAGAATAAGGCTTGAAAACCTACAAAGAAAATAAAGCACTATAAATATTCCAAATT

AACAATAACACTTAAGGTTCTCAGATTACACAGAGGTGCCACCTACCGGATCTTGAGAAG

CCATAATCAATCCTGTCTTCCAAAATAGGAGAAAATAAGAAAATCTTCAAGAGAAGACAA

CCTCTCAGCTGTAGCAGCGGCACAAAAGCTGCATTGGAAAACTATTAAAAGGCTGTCTCC

GCCCTTATTCCAAGCAAGTCTCATCCTGTTTAAAAATTCCTCTCCGTTGCCAAGCTACCA

CAAGATTTTATCAGAGCTCAGTCTCCACAAAGCTCTATGGTCTGCACAAAAGCAGGATGC

ATATGCCGGAGGAATAAGGAGCAGATGGGGGTTCAACAGACAAGCCACACAATGTCCGGA

AGGCAAAAATTTTTTCTCACACCAAAAGGAAGCTGGAGACAAAGGCTTAAAAAATTTTTC

AGTCTGCCACTCTAATAATATTGTTAACACAGGGTTACAAGTTATTCTCATAAATCTTTC

TGCATGTAGTTTTAAAAAAGCAGAACTTTGTAAAATATATACTATATCTGGAAAAGACAA

AGCAAAACTAGCAATATCCTTACTAGTTAAATGTAACAAAATCTTAATCCAAATTTCTTC

TGGGAGAATGTGAACAACATTTCCAATATATCCCTATAAAAAAACAGAACATTTTTTCCA

CATCAAATCAGGAAAACAAGTACAATATTACTTTTCTAAAAAATTTTAAAGGTCATAATA

TTACCTTAGGACAATACACATCTGGAAAGCAGAAAAAATCAGTGCAGGAATATTCAGTAT

CAACATCCATAAACACAACTCGTCAGCTTTCAGTTTAAATGTAAAATTCAGGTCTTAAAA

TCTGCTCAGCCTTTAACTTGCACAATAGCTTCTTATAAAGCCTCTTTCTTTTCTTAACAC

TCAATTCAGGAACTCCACACTCCGGCCACTTCCAACCGTCTTCCAAATTTTGAAAAAGTC

CAGGCTTCCATAGAATCACCTCTTCCAATCCGGGCCAAAGCTTGCGATGATCTAAACAGT

TACAACCAGCTTCAGATGGAGAAGCACATAGTAAATGAGGATTAGTTAAACATGCAATAC

AGTGTCCTGATGGCAGAAACAATCGGTCGGAACACCAATAAGAATTCAAATCTAGAGGTT

CATAATTAGCTAAAGTTCTATCAGCCCGCAGACAGTTTAAAGCTTTATTACAAGAAACAC

GGAAAAAACGCTTAAAA-AAATGTTTCAGGAACGAAGGCGTGACAATGACATCAAAAAGT

TCCTCAAAGCTCAACACAAAGCTAGCAACATCTCGACTAGATAGATGAAGTAATATCTTC

GACCACAACTCAACGGGCAAAATAGCTAAAGGTTCAAAAACCTAAAAAGCAAATAAAAAG

CACATATTAACAACTGTCACTGCAGAGTACTCCAAAACAAAAACACCAGTAACATTTAAA

CCTACCCGCCATTCCAGGACCATTCACGACTTTCTCCAGAAGCCCCACCTCACGCTAGTA

GACGCCGAGTATTCAAAATGGCCGCGCCACGCCCCCGGTGCATGTTAATATATATGAATG

------------------------------------------------------------

------------------------------------------------------------

------------------------------------------------------------

------------------------------------------------------------

------------------------------------------------------------

------------------------------------------------------------

------------------------------------------------------------

------------------------------------------------------------

------------------------------------------------------------

------------------------------------------------------------

------------------------------------------------------------

------------------------------------------------------------

------------------------------------------------------------

------------------------------------------------------------

------------------------------------------------------------

------------------------------------------------------------

------------------------------------------------------------

------------------------------------------------------------

------------------------------------------------------------

------------------------------------------------------------

------------------------------------------------------------

------------------------------------------------------------

------------------------------------------------------------

------------------------------------------------------------

------------------------------------------------------------

------------------------------------------------------------

------------------------------------------------------------

------------------------------------------------------------

------------------------------------------------------------

------------------------------------------------------------

------------------------------------------------------------

------------------------------------------------------------

------------------------------------------------------------

------------------------------------------------------------

------------------------------------------------------------

------------------------------------------------------------

------------------------------------------------------------

------------------------------------------------------------

------------------------------------------------------------

------------------------------------------------------------

------------------------------------------------------------

------------------------------------------------------------

------------------------------------------------------------

------------------------------------------------------------

------------------------------------------------------------

------------------------------------------------------------

------------------------------------------------------------

------------------------------------------------------------

------------------------------------------------------------

------------------------------------------------------------

------------------------------------------------------------

------------------------------------------------------------

------------------------------------------------------------

------------------------------------------------------------

------------------------------------------------------------

------------------------------------------------------------

------------------------------------------------------------

------------------------------------------------------------

------------------------------------------------------------

------------------------------------------------------------

------------------------------------------------------------

------------------------------------------------------------

------------------------------------------------------------

------------------------------------------------------------

------------------------------------------------------------

------------------------------------------------------------

------------------------------------------------------------

------------------------------------------------------------

------------------------------------------------------------

------------------------------------------------------------

------------------------------------------------------------

------------------------------------------------------------

------------------------------------------------------------

------------------------------------------------------------

------------------------------------------------------------

------------------------------------------------------------

------------------------------------------------------------

------------------------------------------------------------

------------------------------------------------------------

------------------------------------------------------------

------------------------------------------------------------

------------------------------------------------------------

------------------------------------------------------------

------------------------------------------------------------

------------------------------------------------------------

------------------------------------------------------------

------------------------------------------------------------

------------------------------------------------------------

------------------------------------------------------------

------------------------------------------------------------

------------------------------------------------------------

------------------------------------------------------------

------------------------------------------------------------

------------------------------------------------------------

------------------------------------------------------------

------------------------------------------------------------

------------------------------------------------------------

------------------------------------------------------------

------------------------------------------------------------

------------------------------------------------------------

------------------------------------------------------------

------------------------------------------------------------

------------------------------------------------------------

------------------------------------------------------------

------------------------------------------------------------

------------------------------------------------------------

------------------------------------------------------------

------------------------------------------------------------

------------------------------------------------------------

------------------------------------------------------------

------------------------------------------------------------

------------------------------------------------------------

------------------------------------------------------------

-----------------------------------------------------

>Bovine AdV-B

------------------------------------------------------------

------------------------------------------------------------
[truncated: 949,462 more chars]
