# Supplementary material for: Characterization of a novel species of adenovirus from Japanese microbat and role of CXADR as its entry factor
Source: Sci Rep. 2019 Jan 24;9:573. doi: 10.1038/s41598-018-37224-z (PMC6345744; doi:10.1038/s41598-018-37224-z)
Supplement: Supplementary file 3 — Supplementary Data 2 [file 41598_2018_37224_MOESM3_ESM.docx]

>CAdV1

M-SLVQSHGTSGLFTEPPNSINQQESSGPSLPAQDATQASASSARAGATPAI--------

-------------------NSTKRKYRGAVVAQRATLSISAIL-DNGQCVQIKYHSNLAS

ALTNLCNTNLY-DLPACLNK-----------PITVHNLPTLIEEAAASYSLIYYY-QRGT

VRKVEF---------------------------RAEIP---LLSFPLKFLVKH---GK--

---VFLIKDISPMQKCEFCGSFFKVTHTCTLRRRDFYFHHVASHSADWWEKISFSPIGAP

ANTERLFIVYDVETYTWHGKFGKQLVPFMLVFQLLGDEHLVNAAKNL-ATTQNWDTWN--

SNEQTALYYCITPEKRAIGVKFKTFRDTLQQHFANNLWSHVLCQNPK--LMEEA-AALGL

ENPEDITANQLKKF----KLQGTPRFIEVYVVGHNITGFDEILLAAQVVST-RAEIPPVF

-DISRNFMPRAGRLLFNDITYSLPNPSY---------VPS--KDYRHWEQGQVLASDLKT

QYIKFMVRDTFSLTH-TSLKNAAKAYSLTVSKGCCPYQAVNEFYMLGSYQQDA--DGFPD

LKYWKDQEEYCFNKDLWKKEKKG------------AYDIIQQTLDYCALDVQVTAQLVNK

LIESYQIFIKNSVNLPETYFNVFQRPTISSNSHAIFKQILY-------------------

-------RAEKPNAPHLNTIIMAPSNEMYEYVRLSIRGGRCYPTYIGVLQEPVFVYDICG

MYASALTHPFPAGSPLNPYERAVAIKAYEHK--MQEHKT-ISYFDEDLLPGIFTIDADPP

AEEFLDVLPPFCSRKGGRLCWTNEPLRGEVTTSIDVITLHNRGWKVTLI-PDTRTTVFPE

WKCLAREYVQLNINAKEKADKSKNQTMRSIAKLLSNALYGSFATKLDNKKTVFSDQIESN

IAK-EIASGAYVVKSSSYIETDNLCA---------------EIMPEFVVAYPPVNFDVH-

RLAPPSYSEEYPTENPH-----------------------------AEGPFMQN------

-FNMTS----YRYKPIMFIDAEDDDFCLHTLEKSTPLITNNRYASQIASFVLAWTRAFVS

EWSQFLYENDAGIP-LEKRILKSVYGDTDSLFTTMEGYRLMEEKGKRRLKKNGGNLVFDP

NNPELTWLVECETKCEKCGADAYSSESVYLAPKLYALKDTTC--PECQYVGKGKLRAKGH

ATSTLSYDVLKACY--YA-DLQQGS-D----IFKTSRMSLRRTLTSVQTHVQPFTVTETT

LTRKLRPWKDKTLHAL----DMNRLIPYSRKYPNPRNN-----------ETTWMELQWMT

------------------------------

>CAdV2

M-SLVQGHGTSGLFTEPPNPINQQESSGPSLPAQDAAQAFASSPRAGATSTI--------

------------------VNPPKRKYKGAVVVQRATLSISAVL-DNGQCVEIKYHSNLAS

ALTNLCNTNLY-DLPACLNR-----------PITAHNLPALIEEAAAPYSLICYY-QRGT

VRRVEF---------------------------QAEVP---LLSFPLKFLVKQ---GK--

---VFLIKDISQMQKCEFCGSFFKVTHTCALRRRDFYFHHVAAHSADWWEKISFTPIGAP

PNTERLFIVYDVETYTWHGKFGKQLVPFMLVFQLLGDDHLVNVAKTL-ATEQNWEIWN--

GKEQDTLYYCITPEKRAIGVKFKKFRDTLQQHIAASLWSHVICQNPQ--LQEKA-TTLGL

ESPEELTPDQLKKF----KLKGNPRFIEVYAVGHNITGFDEILLAAQVVST-RAEIPPVF

-EICRNFMPRAGRLLFNDITYSLPNPSY---------VPA--KSYEHWEQGQVLASDLKS

QYIKFMVRDTFSLTH-TSLKNAAKAYSLTVSKGCCPYQAVNEFYMLGSYQQDA--DGFPD

LKYWKDQEEYSFNKDLWIKEKKG------------AYDIIQQTLDYCALDVQVTAQLVNK

LIESYQIFIKNSVNLPETSFNVFQRPTISSNSHAIFKQILY-------------------

-------KAEKPNTHHLSTILMAPSNEMYEYVRLSIRGGRCYPTYIGVLQEPVFVYDICG

MYASALTHPFPAGSPLNPYERALAIKAYEQK--MLNHKT-ISYFDKDLLPGIFTIDADPP

AEEFLDVLPPFCSRKGGRLCWTNEPLRGEIATSIDVITLHNRGWKVTLI-PDTRTTVFPE

WKCLAREYVQLNISAKEEADKSKNQTMRSIAKLLSNALYGSFATKLDNKKTVFSDQIESN

IAK-EIASGAYVVKSSSYIETDNLCA---------------EIMPEFVVAYPPVNSDVR-

QLAPPSCSEEDPTKDPL-----------------------------AEAPFMHN------

-FSMTS----YHYKPIMFIDAEDDDFCLHTLEKSTPLIANNRYPSQIASFVLAWTRAFVS

EWSQFLYENDAGTP-LENRVLKSVYGDTDSLFTTMEGYKLMEEKGKKRLKKNGGKLVFDP

SNPELTWLVECETQCEKCGSDAYSSESVYLAPKLYALKDTTC--PKCHHVGKGKLRAKGH

ATSTLSYDVLKACY--YA-DMQQGS-D----VFKTSRMSLRRTLTSVQAHVQPFTVTETT

LTRKLRPWKDKTLHAL----DMHRLIPYSRKHPNPRNT-----------ETTWMELQWMT

------------------------------

>BtAdV-Mm32

M-SLVQNDGARRLFPEQPNSDHQPPGRSPGEPAQIPAPSPAGAPLPGAAAPV--------

-------------------KRQRRQYKGAVATQRATLSVSAVL-DDGQFVEIKYHADWKA

ALASLCHANLC-EVPACFDN-----------PLTPHSLPTLLEQAAAAHSLICYY-QRGK

VRRVQM---------------------------QAAQP---LLHFPLQFLVKQ---GK--

---VHLIKEIAATHKCEFCGSFYKVTHTCALRRRDFYFHHVAAHSSDWWEKIAFTPLGSP

SETERLFIVYDVETYTWHGRFGKQLVPFMLVFQLVGAPALVAAARKL-AVAQGWNSWD--

VEGD--VFYCLTPEKKAIGIKFKKLRDTLQQLLASQLWSHFLSQNPD--LEAKT-RELGL

PNSEDISYAALKKL----KLQGAPRFIEVYVVGHNITGFDEILLAAQVVST-RAEIPPAF

-QISRNFMPRAGRLLFNDVTYALPNPAY---------FPR--KDYTNWEKGQLLASDLRN

QFIKFMVRDTFTLTH-TSLRNAAKAYNLPVSKGCCPYQAVNEFYMLGTYRRDA--DGFPA

EDYWKDVEEYRFNKQLWREEKKD------------AYDIIQSTLDYCAQDVKVTTQLVLK

LTEAYQAFVAESVNLPEASFNVFQRPTISSNSHALFKQILF-------------------

-------RAEKPNQSHLGTTLLAPSNEMYEYVRASIRGGRCYPTFIGVLDEPVFVYDICG

MYASALTHPFPAGPPLNPFERALAVRRYELK--MQTEAQ-ISYFDPELLPAILTIDADPP

ADEFLDVLPPFCSRKGGRLCWTNEPLRGEVATSIDVITLHNRGWKVRFV-PDARATVFPE

WKCLAREYVQLNIQAKEQADKNKNQTMRSIAKLLSNALYGSFATKLDNKKTVFSDQIDAE

TAH-DISTGAFVVKSSSYIETDNLCA---------------EIMPEFVVAYPPDPS----

-VALTH-TAVANDGDPH-----------------------------PETLFMSP------

-TNVTS----YTYKPITFLDAEDDDFCLHTLEKSTPLIANNRYPSQIASFVLAWTRAFVS

EWAQFLYADDAGTP-LERRNLKSVYGDTDSLFTTREGYRLMEEKGKKRLKKNGGKLVFDP

DHPELTWLVECETQCEKCGGDAYSTESVYLAPKLYALKNTVC--TRCGHVGKGKLRAKGH

ATSELSYDTLKACY--LA-DLQQGS-D----VFQTSRTSLRRTLASVQAHVQPFTVTETT

LTRKLRPWKDKTLHEV----DSHRLIPYSRRFPNPRNT-----------ETTWMELPWTS

------------------------------

>Bat AdV-A

M-SLVQNDGARRLFPEQPNSDHQPPGHSPGEPAQIPAPSPAGAPLPGAAAPV--------

-------------------KPQRRQYKGAVVTQRATLSVSAVL-DDGQFVEIKYHADWKA

ALASLCHANLC-EVPACFEN-----------PLTPHSLPTLLEQAAAAHSLICYY-QRGK

VRRVQM---------------------------QAAQP---LLHFPLQFLVKQ---RK--

---VHLIKDIAATHKCEFCGSFYKVTHTCALRRRDFYFHHVAAHSSDWWEKIAFTPLGSP

PETERLFIVYDVETYTWHGRFGKQLVPFMLVFQLVGAPALVAAAREL-AAAQGWNSWD--

AEGD--VFYCLTPEKKAIGIKFKKLRDTLQQLLASQLWSHFLSQNPD--LEAKT-RELGL

PNSEDISYAALKKL----KLQGAPRFIEVYVVGHNITGFDEILLAAQVVST-RAEIPPAF

-QISRNFMPRAGRLLFNDVTYALPNPAY---------FPR--KDYTDWEKGQLLACDLRN

QFIKFMVRDTFTLTH-TSLRNAAKAYNLPVSKGCCPYQAVNEFYMLGTYRRDA--DGFPA

EDYWKDAEEYRFNKQLWREEKKD------------AYDIIQSTLDYCAQDVKVTTQLVLK

LTEAYQAFVAESVNLPEASFNVFQRPTISSNSHALFKQILF-------------------

-------RAEKPNQSHLGTTLLAPSNEMYEYVRASIRGGRCYPTFIGLLDEPVFVYDICG

MYASALTHPFPAGPPLNPFERALAVRRYELK--MQTEAQ-ISYFDPELLPAILTIDADPP

ADEFLDVLPPFCSRKGGRLCWTNEPLRGEVATSIDVITLHNRGWKVRFV-PDARATVFPE

WKCLAREYVQLNIQAKEQADKNKNQTMRSIAKLLSNALYGSFATKLDNKKTVFSDQIDAE

TAH-DISTGAFVVKSSSYIETDNLCA---------------EIMPEFVVAYPPDPS----

-VALTH-TAVANDGDPH-----------------------------PETPFMSP------

-TSVTS----YTYKPITFLDAEDDDFCLHTLEKSTPLIANNRYPSQIASFVLAWTRAFVS

EWAQFLYADDAGIP-LEKRNLKSVYGDTDSLFTTREGYRLMEEKGKKRLKKNGGKLVFDP

DHPELTWLVECETQCEKCGGDAYSTESVYLAPKLYALKNTVC--TRCGHVGKGKLRAKGH

ATSELSYDTLKACY--LA-DLQQGS-D----VFQTSRTSLRRTLASVQAHVQPFTVTETT

LTRKLRPWKDKTLHEV----DSHRLIPYSRRFPNPRNT-----------ETTWMELPWTS

------------------------------

>Bat AdV-G

M-ALVQGNGTGSVYSKQANSDNQQEGSSPGHPAQVPPPPSASTPRSRPTASV--------

-------------------RPSKRQYKGTVVAQRATLSISAVL-DDGQSVEIKYHSNFEQ

ALSSLCHANLH-DVPGCLAG----------APVTVNNLPDLIEQAAAPFSLICYY-KRGT

VKRVQF---------------------------QASSP---LLSFPLKFLVKQ---GK--

---VFLIKEISPIQKCEFCGSFFKVAHTCTLRRRDFYFHHVSAQSSDWWEKISFSPIGSP

PETERLFIVYDVETYTWHGKFGKQLVPFMLVFQLVGTPSLVNTAQKL-AVQLQWNTWN--

NKED--IFYCLTPEKKAVGIKFKQFRDLLQQKVAASLWSHVLCENPE--LVEKA-ANLGL

ASPEDIPALEIKKT----KLKGTPKFIEVYVVGHNITGFDEILLAAQVVST-RADIPPVF

-EITRNFMPRAGRLLFNDVTYSLPNPAY---------FPR--KDFTEWEQGQLLSSDLKS

QYIKFMVRDTFTLTH-TSLRNAAKAYSLPVAKGCCPYQAVNEFYMLGTYQQDS--DGFPD

VKYWKDQAEYAENKQLWIESKKG------------AYDIIQSTLDYCALDVEVTTQLVQK

LTEAYQTFVVQSVNLPAASFNVFQRPTISSNSHAIFKQILY-------------------

-------RAEKPDQPHMGTTVLAPSNEMYEYVRASIRGGRCYPTYIGILKQPVYVYDICG

MYASALTHPFPAGPPLNPYERALAVRCYEVK--MQSQKT-ISYFDPELLPGILTIDADPP

ADEYLDVLPPFCSRKGGRLCWTNEPLRGEIATTIDVITLHNRGWTVRLV-PDERATIFPE

WKCLAREYVQLNIQAKELADKSKNQTMRSIAKLLSNALYGSFATKLDNKKTVFSDQIDPH

TSQ-EIASGSYIVKSSSYIETDNLCA---------------EIMPEFVVAYPPAAA----

-PPSPP-PSTSNAEKDD-----------------------------PEPPFISH------

-GNVTS----YTYKPIIFLDAEDDDFCLHTLEWASPLIANNRYPSQIASFVLAWTRAFVS

EWSQFLYEDDAGTP-LDQRPLKSVYGDTDSIFSTEEGYRLMEAKGKKRLKKNGGSLVFDP

AKPELTWLVECETQCEKCGGDAYSSESVYLAPKLYALKDTTC--PTCGHVGKGKLRAKGH

ATSTLSYDVLKACY--LA-DLQHGN-D----LFQTSRMSLRRTLASVQAHVQPFTVTETT

LTRKLRPWKDKTLHPL----DTHRLVPYSKRFPNPRNT-----------ETTWMELPWTN

------------------------------

>BtAd-Vs9

M-SLVQGDRTGGFLSEQTNSDNQPESGGAGFPAQVESQASAGTPRPCPA-----------

--------------------PLKRKYKGTVVTRRATLSISAIL-DDGQPIEIKYHSDLPS

ALTSLCNAHLY-DLPSCLAS-----------QITPANLPAIIEQAAAPLSVICYY-KRGT

VRQVMF---------------------------NAESP---VLSFPLRFLVKQ---GK--

---VYLIKDIASTNKCEFCGSFYKVTHTCTLRRRDFYFHQVAAHSADWWEKIAFTPIGSP

PSTERLFIVYDVETYTWHGKFGKQLVPFMLVFQLVGDHHLVAEAIKL-AQEQEWDTWG--

GYDN--IFYCVTPEKRAIGLKFKKFRDTLQQQLANTVWAHVLGQNPH--LHQEA-INKGL

SNSEELTPADLKKC----KLNGHPRFLEVYAVGHNITGFDEILLAAQVVST-RAVIPPVF

-EISRNFMPRAGRLLFNDVTYSLPNPAY---------VSQ--RVYENWEKGQLRETDLKN

QYIKFMVRDTFTLTH-TSLKNAAKAYSLSVAKGSCPYQAVNEFYMLGTYQQDD--SGFPA

KKYWRDEEEYQLNRDIWLSKKKG------------AYDIIQETLDYCALDVQVTTQLVEK

LIESYQQFIETSVGLPDTYFNVFQRPTISSNSHAVFKQIMY-------------------

-------RAEKPQQNNLGNTLLAPSNEMYDYVRSSIRGGRCYPTYIGVLSEPVFVYDICG

MYASALTHPFPAGPPLNPYERALAVRAYEVK--MQSQSN-VSYFDPDLLPGIFTIDADPP

DDVFLDVLPPFCSRKGGRLCWTNEPLRGEIATSIDVVTLHNRGWKVKII-PDARTTVFPE

WKCLAREYVQLNINAKEKADREKNQTMRSIAKLLSNALYGSFATKLDNKKTVFSDQIEPT

TAK-EIASGEYVVKSSSYIETDNLCA---------------EIMPEFVVAYPPPPA----

-IADPS-AEGHTIQEKT-----------------------------TDPPFISN------

-QGVTS----YTYKPIMFIDAEDDDFCLHTLEKSSPLIVNNRYPSQIASFVLAWTRAFVS

EWAQYLYQTDAGIP-LEKRLLKSVYGDTDSLFTTFEGYKLMEEKGKKRLKRNGGNLVFDP

ANPELTWLVECETQCEKCGSDAFSSESVYLAPKLYALRDTTC--TKCGHVGKGKLRAKGH

ATSSLNYNMLKDCY--LA-DLQQGS-D----IFSTSRMSLRRTITSVQAHVQPFTVTETT

LKRTLRPWKDKTLHAL----DTNRLIPYSRKHPNPRNT-----------ETTWMDLPWMN

------------------------------

>Bat AdV-B

M-SLVQGDRTRGILPKQTDSNYQPKGSSASLSAEIQSQTTPGTPRPSTASSL--------

-------------------KTPRRKYKGAVVAQRATLSISAML-DDGQPAEIKFHSNLLT

ALTALCNTNLH-DLPECFLA-----------PITAPNLPALIEQAAAPYSLICYY-NRGS

VRRVQF---------------------------QAESP---LLSFPLKFLVKH---NK--

---VFLIKDISPVNKCEFCGSFFKVTHTCSLRRRDFYYHHVAAHSSDWWEKIAFAPIGSP

AETERLFIVYDIETYTWHGKFGKQLVPFMLVFQLHGDAHLVAAATAL-ARELGWDRWQ--

DKED--VFCIVTPEKRVIGIKFKQFRDRLQQCLANSLWSHFLVANPQ--IQEEA-TNRGL

ACPEDLFPEDLRKI----KVKGVPRFIEVYAVGHNITGFDEILLAAQVVST-KAEIPSVF

-EISRNFMPRAGRLLFNDVTYSLPNPAW---------VPT--KTFEHWEAGQLSACDLKN

QFLKFMVRDTFTLTH-TSLRNAAKAYSLPVAKGCCPYQAVNDFYMLGTYQQDE--QGFPA

VEYWKDREEYLLNKALWLDKKTG------------AYDIVQQTLDYCALDVQVTAQLVQK

LIASYQEFIATSVNLPAASFNVFQRPTISSNSHAIFKQILY-------------------

-------RAEQPGQGHFNTILLAPSNEMYEYVRASIRGGRCYPTYIGVLAEPVFVYDICG

MYASALTHPFPAGAPLNPYERALAMRAYELK--MQQCPQ-ISYFDAELLPAIFTIDADPP

EDSHLDVLPPFCSRKGGRLCWTNEPLRGEIATSIDVITLHNRGWKVTLV-PDARATVFPE

WKCIAREYVQLNINAKEKADKDKNQTMRSIAKLLSNALYGSFATKLDNKKTVFSDQIEHN

LAK-EIAAGEFVVKSSSYIETDNLCA---------------EIMPEFVVAYPPADT----

-PSSE--VDASPLSEDP-----------------------------EETPFIGN------

-DRMTS----YTYKPITFLDAEDDSFCLQTLERASPLIANNRYPSQIASFVLAWTRAFVS

EWSQFLYADDVGIP-LEDRQLKSVYGDTDSLFTTLEGYKLMETKGKHRLKKNGGKLVFDP

SNPELTWLVECETQCEKCGSDAYSSESVYLAPKLYALKDTTC--VSCGHVGKGKLRAKGH

ATSSLSYDVLKACY--LA-DMQHGS-D----VFHTSRMSLRRTLTSVQSHVQPFTVTETT

LKRRLRPWKDKTLHPL----DANRLVPYSRRFPNPRNT-----------ETTWMEWQ---

------------------------------

>Equine AdV-A

M-SLVQGHGTGGVYAEPADPEYQPPGGCQGDTAASQPTAAPCAQPGGAAAAL--------

--------------------KATRQYRGTVVARRATLFLPAVL-EDGQTVEIKYHAQLTP

ALVSLFKVNLH-EVPECLSV-----------PLTPKNLPEALERAAAPYSLICYY-KRGA

VRRVQM---------------------------QAADP---LLSFPLRFLVKR---GR--

---VYLIKDIAVSNKCDFCGSFYKVTHTCSARRRDFYFHHISTHSADWWETISFTPIGSP

P-TDRLFIVYDVETYTWHGRFGKQLVPFMLVFQVFGTEDLADLARQV-AFQEGWDRWQ--

DRED--TFFCLNPQKKTVGMRFKRFRDILQSKVVAALWSHVLSQNPQ--LEEFA-TQAKL

PYSEEMTPEQLKEL----KLQGLPRFIEVYIVGHNITGFDEILLAAQVINN-RAAIPPAF

-RVTRNFMPRAGRLLFNDVTYSLPNPVY---------RQR--KDFAEWEAGCLLPEDCKW

QYVKFMVRDTFALTH-TSLRNAAKAYGLETSKGHCPYQAVNEFYMLGSYQQDE--DGFPA

QKYWGDPEEYRANKALWLQEKRD------------AYDIVRHTLDYCAQDVIVTAQLVRK

LAESYRAFVAESVNLPAASFNVFQRPTISSNSHAIFKQVLF-------------------

-------RAERPDKNNLGEALLAPSHEMYEYVRASIRGGRCYPTYIGVLSEPVYVYDICG

MYASALTHPFPAGYPLNPYDRAVAVSVYERK--MKAGGP-LGYFDPDLLPAVFTIDADPP

DDEMLDVLPPFCSRKGGRLCWTNEPLRGEIATSIDVITLHNRGWSVTIL-PDEKTTVFPE

WKCIAKEYVQLNIAAKEKADREKNQTMRSIAKLLSNALYGSFATKLDNKKTVFSDHLEED

TVR-DIAAGVYSVKSSSYIETENLCA---------------EIMPEFVVAYPPPRH----

--ADTS-AARDSNDSDS-----------------------------EGDPFIPV------

-TPVTS----YQYKPITFVDAEDQDFCLHTLEKNSPLVSNNRYASQIASFVLAWTRAFVS

EWAQFLYAEDAGTP-LERRALKSVYGDTDSLFLTARGYRLMEEKGKKRIKKHGGPLVFDP

ENPDLTWLVECETQCSRCGADAYSSESVFLAPKLYALKDTTC--PACGFVGKGKLRAKGH

AAAQLSYELLTLCY--YA-DAQQGS-D----KFHTSRMSLRRALASVQAHVQPFTVTETT

LTRTLRPWKDKTLHEL----DEHRLVPYCRRWPNPRNT-----------EITWMDLSWMN

S-----------------DSSGPDSQ---P

>Skunk AdV-A

M-SLIQSHGTCGVYSEQADSEHQQASGGLGHSAEIRTQASASTSPECAASLI--------

-------------------KRQKQKNKGTVVSQRATLSLSAVL-DDGTNVEVKYHARLET

ALAHLFHVNLF-DVPTCLQC-----------SITVNNLPHLLEVVNAPQSTIFYF-KKGL

LKRVKF---------------------------FGVES---LLTFPLNFLVKQ---GK--

---VYLIKEVNETNKCEYCGSFFKVSHTCTLRRRDFYFHHVSGHTADWWEKIPFKPLGAP

RETQRVYLVYDIETYTWHGSFGKQLVPFMLVFKVFGDPALSSLIISL-VKQEQWCEWE--

-EPN--IFYLITPEKKTIGLKFKKLRDLIQQKIASSFWSSVLQLNPH--LEKES-RKLGL

ACSEDLSPEQLAKM----KLKGPPQFIEVYIIGHNITGFDEIVLAAQVISA-RTEIPAVF

-QITRNFMPRSGRLLFNDITYSLPNPDY---------KAR--QDYEDWEQGIFLSSDSKT

QYIKFMIRDTYTLTH-TSLQNAAKAYSLTNNKGSCPYQAINDFYMFGTYQQDP--DGFPA

LHYWKNAEEYTLNKEIWGEKKQD------------AYDIIQHTLDYCALDVKVTAELVLK

LIESYQDFIQQAVNLPSCSFHIFQRPTISSNSHAIFKQILY-------------------

-------RNEKPNQQNMAALLLAPSKEMYDYVRSSIRGGRCYPTYIGIYSEPIFVYDICG

MYASALTHPMPSGMPLNPFERALAVRAYEVK--LKKAGCLIDYFDPDLLPGIFTIDADPP

PSESLDVLPPFCSKKGGRLCWTNEPLRGEIATSIDVITLHNRKWRVRIV-PDSRTIIFPE

WKCLAREYVQLNIQAKEQADKNKNQTMRSIAKLLSNALYGSFATKLDNKKIVFSDQLEHQ

TIK-EIASGAYVVKSSSYIETDNLSA---------------EVLPEFVVAYPPLSS----

-AASAKSSSGNTDDEEE-----------------------------EAPPFMPP------

-TSMTS----YVYKPITFLDAEENDFCLHTLEKSSPLITNNRYSSQIASFVLAWTRAFMS

EWSQFLYDSDVGIP-LEERQIKSVYGDTDSMFVTQKGYQLMEQKGKKRLKKNGGPLVFDP

EHPQLTWLVECETQCEKCGADAYSPESVFLAPKLYALKSVVC--TVCNHVGKGKLRAKGH

ATSDLSYDILSACY--YA-DVQQGT-E----KFQTSRMSLRRTLASVQAHVQPFTVTETT

LTRTLRPWKDKTLFAL----DLHRLVPYSRQHPNPRNT-----------EVTWMELPWMT

------------------------------

>Ovine AdV-A

M-SVVQSDRPSGIFRPAGDSEHQPQSYPSSHSLQAGKKAPSSSKRARAASP---------

---------------------GSGVKRSTIIAKRATLAAHGTL-EDGTHVKVFYHSSLVA

ALENLFHVHLL-QTPQSIQL----------NSIDGKNALEMLEALKPAEGATFTY-IKGR

LIKQQA---------------------------AVENP---ILPFPIFFLIKQ---NK--

---VFLIKSFQSSQKCEFCGDFFANSHTCSVRRRDFYFHHINFKSSEWWSQISFQPIGSC

DDTKRFFLTYDVETYTWHGKHGKQLVPFMLVFHLSGELELVSLSANI-AEQQRWLAWD--

-TPH--TYYYVSPIKGEIGKAFKDLRYEIQKQVTRLLWDNFVGENPE--LAEIQ-ERHHV

NHIDDITAE-MHKI----KTKGSPQFIEIYVIGHNICGFDEIVLAAQVIHN-RTDVLPAF

-KINRNFMPRNGKILFNDISFCLPNPKY---------EKR--KDFADWECGKLTAADHKY

QFVKFMVRDTFALTH-TSLRNAAGAYELPVEKGSCPYEAVNEFYRIGSYQQDE--DGFPS

LRYWKSSEEYQLNKALWREKNVG------------AYDIIQQTLHYCVQDVLVTSALVNK

LQESYKNFIASQVNLPDASFNIFQRPTISSNSHAIFKQILY-------------------

-------REVRPNKANLDNVLLAPSHEMYDYVRQSIRGGRCYPTYIGIMEQPIYVYDICG

MYASALTHPFPSGQPLNPYERALAATEWIRK--LENLEQKIDYFDECLLPGIFTIDADPP

DELFLDELPPFCSRKGGRLCWANEPLRGEVATSIDLITLHNRGWAVRIL-PDERTTIFPE

WKCVAKEYVQLNIGAKEKADKEKNQTMRSIAKLLSNALYGSFATKLDNKKIVFSDQLEAS

ASK-TIARGNFSIKSSSFIETDNFSA---------------EIMPEFVVTYPPAPS----

-------AELDESDENE-----------------------------EHTLFIPK------

-DSHVT----YKYKPITFLETEDDDICLHTLENNSPLIENNRYASHIASFVLAWTRVFVS

EWAEFLYAEDRGKP-LHQRTIKSVYGDTDSLFVTEEGHRLMEQRGKHRIKKNGGKLVFDP

KNPSITWLVECETQCEKCKSDAFSSESVFLAPKLYALKNTVC--TCCGHVGKGKLRAKGH

ATTELCYDTLAKCY--LS-DAQQGS-Q----RFHTSRLSLKRTLATNQSNAAPFTVTETT

LTRTVRPWKDKTLVDI----DGHRLMPYSKSNPNPRNN-----------DVCWMTLPWNM

------------------------------

>Porcine AdV-C

M-SVVPSDGSGRLLGAEGHPKHQQKGRGHGDEAETGRQTYAGTARGRPARS---------

-----------------------GPVRGNLVAKRATLSASGTL-EDGTHVDIKFHSKTLE

ALENLFHLHLL-QLPPL-------------PPISTSNVSDVLQLLAPREGSVVVY-NRGR

VSVKQV---------------------------EIREP---KAHFDIQFLLKQ---GK--

---VYLIKEIQDTQKCEYCGDFFKTSHTCSVRRRDFYFHHVHHKSSDWWENIPFQPLGSY

RETERLYITYDVETYTWHGKHGKQLVPFLLVFHISGEPHLVKVAESV-AQDLNWCPWT--

-EKH--TFYYLNPQKSAVGRMFKSFRDELQNRVTQDLWKTFLSQNPH--LVETA-ARLNL

GSVDDITPEVLKKE----KICGEPRFIEVYVIGHNISGFDEIVLAAQVICH-QSKTIKAF

-KISRNFMPRNGKILFNDITFGLPNPLF---------EKR--KEFQEWERGSMAVQDMKQ

QFVKLMVRDTLMLTH-TSLRNAAKAYDLPVEKGCCPYEAVNEFYRTGTYQKDE--DGFPS

LRYWKDQEEYALNKELWREKKCG------------AYDLISSTLTYCAQDVLVTSSLVRK

LQESYQSFIANEVNLPDSSFNIFQRPTISSNSHAIFKQILY-------------------

-------RAEKPERQHLGEVLLAPSNEMYDYVRQSIRGGRCYPTYIGILHEPIYVYDICG

MYASALTHPMPSGSPLNPFERALAVAVWEDQ--LKSVGQKMDYFDEKLLPGIFTIDADPP

DESFLDVLPPFCSRKGGRLCWTNEPLRGEVATSVDVITLHNRGWRVRLV-PDERTTIFPE

WKCLAKEYVQLNIAAKEKADREKNQTMRSIAKLLSNALYGSFATRLDNKKIVFSDQLEEE

SSK-NISRGKYSVKSSSFIETDNFSA---------------DIMPEFVVAYPPVAD----

-------VSNEDNEAAE-----------------------------EATPFIGK------

-SDHVT----YKYKPITFLDVEDDDVCLHTLESSSSLVLNNRYASHLASFVLAWARVFVS

EWSEFLYENDRGVP-MEERQIKSVYGDTDSLFVTEEGHRLMKEKGKHRIKKNGGSLVFDP

QHPQVTWLVECETRCDKCGEDAYSPTSVFLAPKLYALKSTVC--SVCGYVGKGKLRAKGH

ATSELSFDVLQRCY--LE-DLQLGS-E----KFKTSRLSLKRTLASCQSNAAPFTVTEAT

LTRTLRPWKDKTLTQI----DQNRLIPYSTSRPNPRNT-----------DLCWMTLPWDS

------------------------------

>Tree shrew AdV-A

M-SLVQSDRPRSLQPESGNSKHQPESRRLRVSSEAATPASSGPQPSRSSAP---------

---------------------ALTRYKGTVVAKRATCVINATD-EEGCFVEIKYHVDTDK

SLQALFDVHLS-ERPEEA------------GNFTAHNVVAKLEAIRPSQASIVKY-FRGK

IKTTHV---------------------------AIENP---RFSFPIRFLIKN---HQ--

---LFLIKEICTTMKCEHCGRYFKGTHTCSARRRDFYFHHINAQSSSWWEPISFFPLGAP

PETKRLYITYDVETYTWHGKFGKQLVPFMLVLHLSGDDELLADAITL-IRQTSYIPWP--

KHDN--TFYYLNPTKKTVGQLFKSLRNTLQTHFAKKLWQLFLDQNPK--ITEYC-QLKQL

-DPDQLTADELKNM----KIVGTPSFWEVYVVGHNINGFDEIVLAAQIISN-KSDVPASF

-HITRNFMPRNGKILFNDITFGLPNPAS---------KKR--QDFTLWEQGGCEASDFKW

QYVKFMVRDTFALTH-TSLRKAAAAYALDVEKGSCPYKAVNEFYMFGSYQVDE--VGFPD

EHYWSSREEYLFNREEWLKKKEH------------AYDIITQTLEYCVMDVVVTAKLVQK

LQTSYAAFVQESVNLPESSFNVFQRPTISSNSHAIFRQIVY-------------------

-------RAEKPQKTHLGQCLLAPSNEMYDYVRSSIRGGRCYPSYIGLLSEPIYVYDICG

MYASALTHPFPSGRPLNPFDRALAVKNWEMR--LKNSQT-IDYFTPHLLPGIFTIDADPP

SETYLDVLPPFCSRKGGRLCWTNESLRGEVATSVDIITLHNRGWKVRIV-TDERTTVFPE

WKCIAQEYVKLNIAAKEKADKEKNQTIRSIAKLLSNALYGSFATKLDNKKTVFADQLDSS

TAK-GIANGEFSVKSSSFIETDCLSA---------------EIMSELVVAYSPDTL----

--LHPQ-QEGATEANTQ-----------------------------ETPTFIGP------

-TDHVT----YTYKPITFLEAEEDEICLQTIEKNSPLIPNDRYPSHIASFVLAWTRAFVS

EWASFLYSEDLGTP-MERRCLKSVYGDTDSLFLTEEGRRLMETKGKHRIKKNGGKLVFDP

ANPDLTWLVECETQCEKCGADAFSSQSIFLAPKLYALKDTTC--PVCKHVGKGKLRAKGH

ATDGLSFEIMSKCY--FA-DSQLGS-D----KFQTSRMSIKRTLASGQSHVQPFTVTETT

LTRTLRPWKDKTMTAL----DQHRLIPYSNSCPNPRNN-----------EVCWIEMSETM

------------------------------

>Bat AdV-C

M-ALVPRHRPGGLLPEARDPEHQPPGRPAGHAAPTSTPAPAGTQPGGGAAPR--------

--------------------RGRPPLKGTIVNRRHTQRVYGTN-AEGEALEVSYALDPVK

GLRNLFLVHLC-SDPPEDLL--------RPEALRRDRLLTTLENIQPPQGDIYTH-ARGF

FQVTSV---------------------------SYPES---SLPFPIRFLVKN---SNGK

PV-VGLIKEIHRVTKCDHCGSFYKAAHTCNVRRRDFYFHQIQTRSAAWWEPISFFPIGSN

PATERLFITYDVETYTWHGQHGKQLVPFMLVMHFSGTEELVRLAEEL-AHRQEWQVWD--

GAEH--TFYYLNPQKSSIGSKFKTFRHSLQKRTARLLLNFVTKQNPQ--LAEFC-QLNAI

RSPEELSPQQMEAL----RLRGQPRFIEVYVVGHNINGFDEIVLAAQVISN-RTDIPSCF

-RISRNFMPRNGKILFNDITFSLPNPDY---------RKRVPGDFELWERGACETSDFKF

QYVKFLVRDTFALTH-TSLRNAAQAYSLQVEKGCCPYQAVNQFYMFGSYLKDG--DGFPA

PHYWKDEQEYSFNKEIWKKKGET------------EYDIVQHTLEYCALDVVVTAQLVQK

LQDSYRTFVAESVGLPQARFNIFQRPTISSNSHAIFRQVVY-------------------

-------RAERPNRANLGSVLLAPSMELYDYVRASIRGGRCYPSYLGVLEEPIYVYDICG

MYASALTHPFPAGRPLHPFDRALAMRAWEQR--LQDPAA-ISYFNTTLLPGIFTIDADPP

PEDYLDVLPPFCSRKGGRLAWTNERLRGEIVTSIDVITLHNRGWRVTLL-PDERTTVFPE

WRCVAREYVQLNIRAKEQADRDKNQTIRSIAKLLSNALYGSFATKLDNKTTVFADQMEDK

YLK-GIATGEYTVKSSSYIEVDNFSA---------------EILPEFKIIYSPEPNQP--

-RELADESQLSDCGDPR-----------------------------GRAPFYTPPAGETP

DSGHVT----YTYKPITFLDVEDGDYCLHTLEKNSPLITNDRYPSQIASFVLAWTRAFVS

EWAGFLYEEDRGVP-LAQRALKSVYGDTDSLFVTERGRYLMETQGKHRIKKNGGRLVFDP

DHPDLTWLVECETQCSFCGADAYASESVFLAPKLYALKDTCC--TVCKKVGKGKLRAKGH

ATTTLSYDLLKSCY--YA-DAQQGS-D----RFSTSRMSLKRTLASAQSHVQPFTVTETT

LKRTLRPWKDQTMNLL---DDHHRLVPYSNSAPNPRNR-----------EVCWIQLPWDM

------------------------------

>Human AdV-A

M-ALVQNNRAGSVYTTPTDSRYQSPGSRPCQSTSGSTPSPARASGRHTASPASRRTRTAA

SSGSSSAPPILSR-------RIGKVHRGTVVSPRAHGILHAIDSANGTPLEIKYYLHLQP

ALTRLCEVNLR-AVPADMLFS-------LTDTMDSSRLYALISRFRPSRAEIWTCRSRGT

VTLSVLA-------FEDPNGSGNAAEEEED---ERQLP--SGIDFPICFLVRG---RQ--

---VHLIQQIQPVQRCEHCARFYKHQHECSVRRRDFYFHHINSHSSNWWQEIHFFPIGSH

PRTERLFITYDVETYTWMGAFGKQLVPFMLVMKLSGDDNLVKHALQL-ALELGWDQWE--

KDST--TFYCLTPEKMKVGQQFRTYRNRLQTSLATDLWMTFLQKNPH--LSQWAQEENGL

VALEDLSYEDLKRA---PAIKGEPRFVELYIVGHNINGFDEIVLAAQVINN-RLDVPGPF

-KISRNFIPRAGKILFNDITFALPNPHY---------KKR--TNFLLWEHGGCDDQDFKY

QYLKVMVRDTFALTH-TSLRKAAQAYALPVEKGCCPYKAVNQFYMLGSYRADA--NGFPL

EEYWKDKEEYLLNQELWKKKGEK------------NYNLIGETLNYCALDVLVTASLVEK

LRSSYAQFVTDAVGLDAAHFNVFQRPTISSNSHAIFRQILY-------------------

-------RAEKPQRTHLGPNILAPSHELYDYVRASIRGGRCYPTYIGVLKEPIYVYDICG

MYASALTHPMPWGPPLNPYERALAVRQWQVA--LENYTCKIDYFDKNLCPGIFTIDADPP

DENQLDVLPPFCSRKGGRLAWTNESLRGEVVTSVDLVTLHNRGWRLRLL-SDERTTIFPT

WKCLAREYVQLNIAAKERADRDKNQTLRSIAKLLSNALYGSFATKLDNKKIVFSDQMEES

LMK-EIAAGRLNIKSSSFIETDTLST---------------EVMPAFERVYSPNQL----

--ALVN-SEAEESDEDQ-----------------------------GPAPFYSPP---PE

NCEHVT----YTYKPITFMDAEEGDMCLHTLESSNPLINNDRYPSHVASFVLAWTRAFVS

EWSEFLYEEDRGIP-LKDRPLKSVYGDTDSLFVTEKGRRLMESQGKKRIKKYGGKLVFDP

SCPELTWSVECETVCSYCGADAYSPESVFLAPKLYALKCLQC--PHCGSTSKGKIRAKGH

ATEALSYDLMLKCY--LA-EAQGED-T----RFSTSRLSLKRTLASAQPGAHPFTVTETT

LTRTLRPWKDKTLVHL----DAHRLVPYSNSQPNPRNE-----------EVCWIEMA---

------------------------------

>Human AdV-B

M-ALVQAHGAGSFHPEASDPGCQPPRRRACESSQGAVPEPTRARRRRTTASHAAGSRAAP

AARCAPSTPLLNMQEATEPPPSKMKNKGTVVAPKGHGTLQAIDISTNGPVEIKYYLNLPH

ALQKIMQVNLL-TLPTNLTPQ-------RLRTLDSSGLRALVLELRPCRAEVWTCLPRGL

VSMTTI---------ETEDGHADADNIVKR---EVQAP---GLNFPLKFLVKG---SQ--

---VQLIHKVHPVNRCEYCGRLYKHKHECSARRREFYFHHINSHSSNWWQEIQFFPIGSH

PRTERLFLTYDVETYTWMGSFGKQLIPFMLVMKLSGDQRLVNIAYDL-AIKLKWDRWR--

QDPQ--TFYCVTPEKMAVGQHFRQYRDQLQTALAVDLWSSFLKANPH--VHEWALEHYAL

TDPTDLTFEELKKL---PHVRGTPRFLELYIVGHNINGFDEIVLAAQVINN-RAEVPQPF

-KITRNFMPRAGKILFNDVTFALPNPAY---------KKR--VDFQLWEQGACDDIDFKY

QFLKVMVRDTFALTH-TSLRNAAQAYSLPVEKGCCPYKAVNQFYMLGSYRAEK--DGFPL

EEYWKDHEEYLLNRELWEKKSQP------------RYDIIQETLDYCALDVLITAELVAK

LQESYAHFIRDSVGLPHAHFNIFQRPTISSNSHAIFRQIVY-------------------

-------RAEKPNRTNLGPGLLAPSHELYDYVRASIRGGRCYPTYIGILEEPLYVYDICG

MYASALTHPMPWGTPLNPYERALAVREWQMT--LDDPAT-ISYFDKDLLPGIFTIDADPP

DEFMLDPLPPFCSRKGGRLCWTNEPLRGEVATSVDLITLHNRGWRIRIV-PDELTTIFPE

WKCVAREYVQLNITAKERADKEKNQTMRSIAKLLSNALYGSFATKLDNKKIVFSDQMDES

LMK-GISAGTVNIKSSSFLETDNLSA---------------EVMPAFEREYLPQQL----

--ALLD-SDPEDSEDEQ-----------------------------RPAPFYTPP---AG

TPGHVA----YTYKPITFLDVEEGDMCLHTVEKVDPLVDNDRYPSHVASFVLAWTRAFVS

EWAGFLYEEDRGTP-LEDRPIKSVYGDTDSLFVTQRGHELMETKGKKRIKKHGGKLVFDP

DEPDLTWLVECETVCASCGADAYSPESIFLAPKLYALKCIYC--PACHKTSKGKLRAKGH

AAEALNYELMVNCY--LA-DMQGANRQ----RFSTSRMSLKRTLASAQPGAHPFTVTETT

LTRTLRPWKDRTLAAL----DAHRLIPYSRSRPNPRNE-----------EVCWIEMP---

------------------------------

>Human AdV-E

M-ALVQTHGSRGLHPEASDPGRQPSRRRSRQSSPGAVPEPARARRRRAPATTASGSRAAP

TARRASSPPLLTM-EAKPLPPA-KKKRGTVVTPQGHGTLQAIDVATNGAVEIKYHLDLPR

ALEKLLQVNRAPPLPTDLTPQ-------RLRTLDSSGLRALVLALRPVRAEVWTCLPRGL

VSMTTI---------EAEEGQADHHDVVQH---QMQAP---RLHFPLKFLVKG---TQ--

---VQLVQHVHPVQRCEHCGRLYKHKHECSARRRHFYFHHINSHSSNWWQEIQFFPIGSH

PRTERLFLTYDVETYTWMGSFGKQLVPFMLVMKLSGDDRLVELALDL-ALQLKWDRWH--

GDPR--TFYCVTPEKMAVGQQFRQYRDRLQTALAVDLWTSFLRANPH--LADWALEQHGL

SDPDELTYEELKKL---PHVKGRPRFVELYIVGHNINGFDEIVLAAQVINN-RAEVPQPF

-RITRNFMPRAGKILFNDVTFALPNPAY---------KKR--TDFQLWEQGGCDDIDFKH

QFLKVMVRDTFALTH-TSLRKAAQAYALPVEKGCCAYKAVNQFYMLGSYRADQ--DGFPL

EEYWKDREEFLLNRELWKQKGQL------------KYDIIQETLDYCALDVLVTAELVAK

LQDSYAHFIRDSVGLPHAHFNIFQRPTISSNSHAIFRQIVY-------------------

-------RAEKPSRANLGAGLLAPSHELYDYVRASIRGGRCYPTYIGILDEPLYVYDICG

MYASALTHPMPWGTPLSPYERALAVREWQAS--LDDLGTCISYFDPDLLPGIFTIDADPP

DELMLDPLPPFCSRKGGRLCWTNEPLRGEVATSVDLITLHNRGWQVRIV-PDELTTVFPE

WKCVAREYVQLNIAAKERADKEKNQTMRSIAKLLSNALYGSFATKLDNKKIVFSDQMDEG

LLK-GISAGTVNIKSSSFLETDNLSA---------------EVMPAFEREYLPQQL----

--ALLD-SDPEDSEDEQ-----------------------------RPAPFYTPP---AG

TPGHVA----YTYKPITFLDVDEGDMCLHTLEKVDPLVDNDRYPSHVASFVLAWTRAFVS

EWSGFLYDEDRGVP-LEDRPIKSVYGDTDSLFVTQRGHELMETRGKKRIKKNGGKLVFDP

NQPDLTWLVECETVCAHCGADAYAPESVFLAPKLYALKSLLC--PACGQTSKGKLRAKGH

AAEALNYELMVNCY--LA-DAQGADRE----RFSTSRMSLKRTLASAQPGAHPFTVTETT

LTRTLRPWKDRTLAAL----DAHRLAPYSRSRPNPRNE-----------EVCWIEMP---

------------------------------

>Human AdV-D

M-ALVQSHGARGLHAEAADPGCQPPRRRARQRSQGAAPGPARAPRRRASAAPARGARTAA

AAGSTPATPLL------------KAHRGTVVAPRSYGLMQCVDTTTNSPVEIKYHLHLKH

ALTRLYEVNLR-TLPPDLDLR---------DTMDSSQLRALVFALRPRRAEIWTWLPRGL

VSLSVL---------EEPQGESHAGEHESH---QPGPP-------LLKFLLKG---RA--

---VYLVDEVQPVQRCEYCGRFYKHQHECSVRRRDFYFHHINSHSSNWWQEIQFFPIGSH

PRTERLFVTYDVETYTWMGSFGKQLVPFMLVMKFSGEPELVALARDL-AVRLRWDRWE--

RDPL--TFYCVTPEKMAVGQQFRLFRDELQTLMARELWASFMQANPH--LQEWALEQHGL

QCPEDLTYEELKKL---PHIKGRPRFMELYIVGHNINGFDEIVLAAQVINN-RASVPGPF

-RITRNFMPRAGKILFNDVTFALPNPLS---------KKR--TDFELWEHGGCDDSDFKY

QFLKVMVRDTFALTH-TSLRKAAQAYALPVEKGCCPYKAVNHFYMLGSYRADD--RGFPL

REYWKDDEEYALNRELWEKKGEA------------GYDIIRETLDYCAMDVLVTAELVAK

LQDSYAHFIRDSVRLPHAHFNIFQRPTISSNSHAIFRQIVF-------------------

-------RAEQPQRTNLGPAFLAPSHELYDYVRASIRGGRCYPTYIGILSEPIYVYDICG

MYASALTHPMPWGPPLNPYERALAAREWQMA--LDDASSKIDYFDKELCPGIFTIDADPP

DEHLLDVLPPFCSRKGGRLCWTNEPLRGEVATSVDLVTLHNRGWRVRIV-PDERTTVFPE

WKCVAREYVQLNIAAKERADRDKNQTMRSIAKLLSNALYGSFATKLDNKKIVFSDQMDES

LLK-SIAAGQANIKSSSFLETDNLSA---------------EVMPALEREYLPQQL----

--ALVD-SDAEESEDEH-----------------------------RPAPFYTPP---SG

TPGHVA----YTYKPITFLDAEEGDMCLHTVEKVDPLVDNDRYPSHVASFVLAWTRAFVS

EWSEFLYEEDRGTP-LQDRPIKSVYGDTDSLFVTERGHRLMETRGKKRIKKNGGKLVFDP

EQPELTWLVECETVCAHCGADAFAPESVFLAPKLYALQSLLC--PACGRSSKGKLRAKGH

AAEALNYELMVNCY--LA-DSQGEDRA----RFSTSRMSLKRTLASAQPGAHPFTVTETT

LTRTLRPWKDMTLAAL----DAHRLVPYSRSRPNPRNE-----------EVCWIEMP---

------------------------------

>Human AdV-C

M-ALVQAHRARRLHAEAPDSGDQPPRRRVRQQPPRAAPAPARARRRRAPAPSPGGSRAPP

TSGGPPASPLLDA-SSKDTPAAHRPPRGTVVAPRGCGLLQVIDAATNQPLEIRYHLDLAR

ALTRLCEVNLQ-ELPPDLSPR-------ELQTMDSSHLRDVVIKLRPPRADIWTLGSRGV

VVRSTITP------LEQPDGQGQAAEVEDH---QPNPP-GEGLKFPLCFLVRG---RQ--

---VNLVQDVQPVHRCQYCARFYKSQHECSARRRDFYFHHINSHSSNWWREIQFFPIGSH

PRTERLFVTYDVETYTWMGAFGKQLVPFMLVMKFGGDEPLVTAARDL-AVDLGWDRWE--

QDPL--TFYCITPEKMAIGRQFRTFRDHLQMLMARDLWSSFVASNPH--LADWALSEHGL

SSPEELTYEELKKL---PSIKGTPRFLELYIVGHNINGFDEIVLAAQVINN-RSEVPGPF

-RITRNFMPRAGKILFNDVTFALPNPRS---------KKR--TDFLLWEQGGCDDTDFKY

QYLKVMVRDTFALTH-TSLRKAAQAYALPVEKGCCAYQAVNQFYMLGSYRSEA--DGFPI

QEYWKDREEFVLNRELWKKKGQD------------KYDIIKETLDYCALDVQVTAELVNK

LRDSYASFVRDAVGLTDASFNVFQRPTISSNSHAIFRQIVF-------------------

-------RAEQPARSNLGPDLLAPSHELYDYVRASIRGGRCYPTYLGILREPLYVYDICG

MYASALTHPMPWGPPLNPYERALAARAWQQA--LDLQGCKIDYFDARLLPGVFTVDADPP

DETQLDPLPPFCSRKGGRLCWTNERLRGEVATSVDLVTLHNRGWRVHLV-PDERTTVFPE

WRCVAREYVQLNIAAKERADRDKNQTLRSIAKLLSNALYGSFATKLDNKKIVFSDQMDAA

TLK-GITAGQVNIKSSSFLETDNLSA---------------EVMPAFEREYSPQQL----

--ALAD-SDAEESEDER-----------------------------APTPFYSPP---SG

TPGHVA----YTYKPITFLDAEEGDMCLHTLERVDPLVDNDRYPSHLASFVLAWTRAFVS

EWSEFLYEEDRGTP-LEDRPLKSVYGDTDSLFVTERGHRLMETRGKKRIKKHGGNLVFDP

ERPELTWLVECETVCGACGADAYSPESVFLAPKLYALKSLHC--PSCGASSKGKLRAKGH

AAEGLDYDTMVKCY--LA-DAQGEDRQ----RFSTSRTSLKRTLASAQPGAHPFTVTQTT

LTRTLRPWKDMTLARL----DEHRLLPYSESRPNPRNE-----------EICWIEMP---

------------------------------

>Human AdV-F

M-ALVPSPRAGGFLPAETHSGPQPPRRRVRQSTAGAAPTATRAPRRRAATASPGEPPSTT

ASGRPPAANNVSL-------TPNSRLRGTIVAPRGQGLLYAIDTATNSPMEIKFHRRLAS

ALTRLLQVNLR-SVPADLNEA-------FLDSLDSSQIRTLALKLKVPRVEVWTCGSRGV

VVPSIIH-------PQQERAGAEEGDEGER---QDTED---FLNFPLRFLVRG---RQ--

---VHLIQEMQSVQRCEYCARFYKYQHECTVRRRDFYFHHINAHSSGWWQKINFFPIGSH

PRVERLFVTYDVETYTWMGAFGKQLVPFMLVMHLSGEEALVKEACRL-ACELQWDTWG--

NDER--TFYVVTPEKLAVGKKFREYRNRLQAHFALQLWRGFLAANPQ--LAEWACLEMGL

FSPDYLTYEELQKA---PKLQGRPRFLELYIVGHNINGFDEIVLAAQVINN-RSDVPGPF

-KITRNFMPRAGKILFNDITFALPNPSS---------KKR--TDYRLWEQGACDDSDFKY

QFLKVMVRDTFALTH-TSLRKAAQAYTLPVEKGCCPYKAVNEFYMLGSYRADE--RGFPA

EDYWKDREEYLLNRELWEKKQCP------------HYDLVRETLDYCALDVLVTAALVQK

LRESYAQFIRDAVGLPEASFNVFQRPTISSNSHAIFRQILY-------------------

-------RTVKPQRSDLGGSLLAPSHEMYDYVRASIRGGRCYPTYIGVLREPLYVYDICG

MYASALTHPMPWGFPLNPYERALAVRDWEHA--LLQVGTPIDYFNRTLLPGIFTIDADPP

PENLLDVLPPLCSRKGGRLCWTNEPLRGEVVTSVDLITLHNRGWHVRLL-PDERATVFPE

WRCVAKEYVHLNITAKERADREKNQTLRSIAKLLSNALYGSFATKLDNKKIVFSDQMDSA

TIK-SIAAGQINIKSTSFVETDTLSA---------------EVMPTFQRAYSPEQL----

--AVVH-SDAEESDEEP-----------------------------GHAPFYTPT---HK

PNDHVT----YTYKPITFMDAEEDDLCLHTLEKVDPLVENNRYPSQIASFVLAWTRAFVS

EWSEILYAEDRGTP-LEQRTLKSVYGDTDSLFVTEAGYRLMETRGKKRIKKHGGNLVFDP

KHPELAWLVECETVCAQCGADAYSPESVFLAPKLYALKCLRC--PSCQQISKGKLRAKGH

AAETLNYDLMLKCY--LA-DFQGED-A----RFHTSRMSLKRTLASAQPGARPFTVTETN

LTRTLRPWKDITLAPL----DAHRLVPYSQSRPNPRNQ-----------EVCWIEMP---

------------------------------

>Bovine AdV-B

M-ALVQSDGASSIHPTPGDSENQPQSRAARIRPAPTAATHARTQRSGPATAP--QARKAK

AAPRPST------------SPLKKAPRGPLVAKRSTLRLHGQT-EDGTTLEVKYHADVSS

SLRNLFELHLE-HCPFDL------------KPENILSFQDTLVRFCNANMCVYKT-YKGK

LKITSIPA-------------------------RADRP---RLPFPLNFLIHK---RK--

---LYLIDTIAEVQRCADCGSYFKQTHTCSTRRRDFYFHHINTQSSDWWEEIKFFPWAAH

PDTRRLFVVYDVETYTWHGSFGKQLMPFMLVFTLFGDAQLCEQAVNI-AKKQKWSSWP--

KQAN--TFYYLNPQRNKVGSLFKQYRDALQEAASTLLWRQFLADNPC--LENLC-LKLGY

VHASDIPFEELCTL----ELKGQPTFLEVYVVGHNINGFDEIVLAAQVINN-KQGIPAAF

-KVSRNFMPRCGKILFNDLTFALPNPTH---------AAR--KDFKDWEEGTPTSADYKF

QFVKFMVRDTFALTH-TSLRNAAAAYALPVEKGCCPYKAVNEFYMLGTYRTDA--DSFPQ

RDYWSSDEEYLLNKSLWLQENSG------------AYDIVQRTLDYCAMDVLVTAELVKK

LQASYLDFVHTSVGLPHCNFNVLQRPTISSNSHAIFRQVVY-------------------

-------RSQRPNRSSLGNFLLAPSNEMYDYVRESIRGGRCYPTYIGVLTEAIYVYDICG

MYASALTHPFPAGKPLNPFDRALAIKNWQDR--LTQLHRPIDYFDRTLLPAIFTIDADPP

PEAFLDVIPPFCSRKGGRLCWTNETLRGEVVTCLDAITLHNRGWRVQIL-NDPRTTVFPQ

WECLARDYVQLNIAPKERADKEKNQTLRSIAKLLSNALYGSFATKLDNRVTVFSDQMEDK

YVR-GISDGTYDIKSTAFVETDNLSS---------------SVMAELKITYSPVKQQTDA

TRKHRQ-CTPTSNSSSD-----------------------------EDAPFYTLG---DP

QNHHVT----YTYKPITFLEADDSALCLHTLQKKSSLIFNNRYPSHIASFVLAWTRAFVS

EWADILYLEDRGTP-LEDRILKFVYGDTDSMFLTQRGKELMDTRGKHRLKGNNRPLVFDP

TNPQLTWLVECETQCPRCHGDAHSQESVFLAPKLYALKNIYC--PSCRRREFWQTSSKGH

ATSQLSYDLLVTCY--YS-TEQLGD-E----KFGTSRLSLRRSLVSRQTHQQPFTVTETT

LARTLRPWKDRTLRAI----DRHRLAPYSNSHPNPRNK-----------ELCWMEMY---

------------------------------

>Bat AdV-E

M-ALVQVHGTSSSQSTPRNSEHQQESGSISIPATLPTTSSPRTPPSGTTATL--------

---------------------RLKPLKGTRVAQRACNTVQGID-TGGNVLEVKYYNDTMK

ALQNLFHVHLQ-ELPPCM------------YNISCASVHSKVMQCGPSLGLIYNF-YRGK

FYVTDV---------------------------GVKEQ---KYDFPLEFLVYK---SK--

---LYLINEVSRMQKCQHCGRIYKNNHTCSLRRRDFYFHCVNAQTSDWWESISFFPIGSF

HGTEQLFITYDVETYTWHGQYGKQLVPFMLVFKLTGNTNLVKIASQI-AHAEQWDCFQPL

KYEN--IFFYINPQKRSIGYKFKRFRELVQKQLTDILWHHFITNNNDE-IEKFK-TEKNI

SETDELTFEQLKKL----KLKGYCKIIEIYIVGHNINGFDEIVLAAQVINN-KFDLPKGF

-KIMRNFMPRCGKILFNDITYALPNPQF---------IKPGKNEYKIWQEGICQAQDLKY

QFVKVMVRDTYALTH-TSLQNAAKAYNLKISKGCCPYQAVNDFYMIGQYEIDE--DGFPA

SKYWKNQDEYQENKQLWKQSKKS------------KYDIIDETLEYCVLDVIVTSELVDK

LLNSYKNFIAETVNLPVANFNIFQRPTISSNSHAIFKQILF-------------------

-------KSENLLSSTLDNILFAPSKEMYDYVRSSIRGGRCYPTYIGVLEDPIYVYDICG

MYASALTHPFPSGKPLNTFERNIEVNKWQFQILMHKNQK-LSYFNKSLLPGIFTIDAIPP

AEEFLDVLPPFCSKKGGRLCWTNEPLKGEVVTSIDIITLHNRGWTVTIL-PDERTTVFPE

WKCVAAEYVRLNIMAKEKADQDKNQTLRNIAKLLSNALYGSFATKLDNKKTLFSDQMDEN

CKK-SIANGSYKIKNSTFIETDNFSA---------------QIIPQFSVLYSPLEP----

-DAGEQ-QQPPENQNTK-----------------------------ELTSLNTP------

-QEHVT----YVYKPITFLDCDEDDACLHTLELNSSLITNDRYPSQIASFVLAWTRAFIS

EWADFLYADDRGTP-LEHRKLKSVYGDTDSLFLTKLGKDLMEQKGKHRIKKNGGSLTFDP

KNPDLTWLVECETQCSKCGQDAYSSESLFMAPKLYALKDTTC--DFCKHVGPGKLRAKGH

AKQDLSYDVMLACY--YA-DVQQGS-E----VFQTSRTSLRRTLTTTVNNVQPFTVTQST

LTRTLRPWKDKTLHQL----NQNILIPYSNSNPNPRNN-----------EVCWTQLPWEN

------------------------------

>Bat AdV-D

M-ALVQVHGTSSSQSTPRNSEHQQESGSISIPATLPTTSSPRTPPSGTTATL--------

---------------------RLKPLKGTRVAQRACNTVQGID-TGGNVLEVKYYNDTMK

ALQNLFHVHLQ-ELPPCM------------YNISCASVHSKVMQCGPSLGLIYNF-YRGK

FYVTDV---------------------------GVKEQ---KYDFPLEFLVYK---SK--

---LYLINEVSRMQKCQHCGRIYKNNHTCSLRRRDFYFHCVNAQTSDWWESISFFPIGSF

HGTEQLFITYDVETYTWHGQYGKQLVPFMLVFKLTGNTNLVKIASQI-AHAEQWDCFQPL

KYEN--IFFYINPQKRSIGYKFKRFRELVQKQLTDILWHHFITNNNDE-IEKFK-TEKNI

SETDELTFEQLKKL----KLKGYCKIIEIYIVGHNINGFDEIVLAAQVINN-KFDLPKGF

-KIMRNFMPRCGKILFNDITYALPNPQF---------IKPGKNEYKIWQEGICQAQDLKY

QFVKVMVRDTYALTH-TSLQNAAKAYNLKISKGCCPYQAVNDFYMIGQYEIDE--DGFPA

SKYWKNQDEYQENKQLWKQSKKS------------KYDIIDETLEYCVLDVIVTSELVDK

LLNSYKNFIAETVNLPVANFNIFQRPTISSNSHAIFKQILF-------------------

-------KSENLLSSTLDNILFAPSKEMYDYVRSSIRGGRCYPTYIGVLEDPIYVYDICG

MYASALTHPFPSGKPLNTFERNIEVNKWQFQILMHKNQK-LSYFNKSLLPGIFTIDAIPP

AEEFLDVLPPFCSKKGGRLCWTNEPLKGEVVTSIDIITLHNRGWTVTIL-PDERTTVFPE

WKCVAAEYVRLNIMAKEKADQDKNQTLRNIAKLLSNALYGSFATKLDNKKTLFSDQMDEN

CKK-SIANGSYKIKNSTFIETDNFSA---------------QIIPQFSVLYSPLEP----

-DAGEQ-QQPPENQNTK-----------------------------ELTSLNTP------

-QEHVT----YVYKPITFLDCDEDDACLHTLELNSSLITNDRYPSQIASFVLAWTRAFIS

EWADFLYADDRGTP-LEHRKLKSVYGDTDSLFLTKLGKDLMEQKGKHRIKKNGGSLTFDP

KNPDLTWLVECETQCSKCGQDAYSSESLFMAPKLYALKDTTC--DFCKHVGPGKLRAKGH

AKQDLSYDVMLACY--YA-DVQQGS-E----VFQTSRTSLRRTLTTTVNNVQPFTVTQST

LTRTLRPWKDKTLHQL----NQNILIPYSNSNPNPRNN-----------EVCWTQLPWEN

------------------------------

>Bat AdV-F

M-ALVQTNGTSDSQSTSRNSEYQQESSVISNSTTSSKVTTTPSTSGCTTSTL--------

---------------------KISKLKGTRVVRRACNYIQGID-SDGEIIEIKYFNDTLK

ALQNIFHVHLQ-ELPFCL------------THLNVSNIFTKIEECQPTNAKIYSY-FKGK

FKTTNI---------------------------NFNEP---IVNFPLQFLIYK---NK--

---VFLINEINAMQKCDYCGRIYKNFHTCNLRRREYYFHNINVQTADWWESISFFPIGSC

TLTEQLFVTYDVETYTWHGQFGKQLVPFLIVFKLSGNTNLVKIATEI-ALEQKWDQYN--

SSEN--LFFYINPQKRSIGYKFKNFRELVQHKLTNLLWNEFIESNKNS-LDTFM-QLNNI

TDYEELSFNQIKKL----TLSGKTRLIEFYIVGHNITGFDEIVLAAQVINN-KYSVPKGF

-KILRNFMPRCGKILFNDVTFALPNPEY---------IKPTKETYKIWQEGICNPQDLKY

QFVKVMVRDTYALTH-TSLENAAKAYNLNIKKGCCPYTAVNEFYMLGTYQTDE--DGFPA

CKYWKNSEEYKENKQIWKQSKKP------------SYDIVQETIDYCILDVNVTSELVNK

LLLSYKNFIAEAVNLPNSNFNIFQRPTISSNSHAIFKQCLY-------------------

-------KSENLLGPNLDEILYAPSKEMYEYVRSSIRGGRCYLNYIGILEEPVYVYDICG

MYASALTHPFPAGKPLNTFERNIEINKYVYY--LQKYKT-ISYFDKNLLPGILTIDATPP

PEHFLDLLPPFCSKKGGRLCWTNEPLKGEIATTIDIITLHNRGWKVEIL-PDDRSTIFPE

WKCIAAEYVKLNIAAKEKADKEKNQTLRNISKLLSNALYGSFATKLDNKKTVFSDQMDDI

CKK-DIAKGTYKIKNSTFIETDNYSA---------------EIIPQFTVLYSPLKR----

-ADSEQ-PQLLNDQNLD-----------------------------TETSIYSV------

-INHVT----YHYKPITFLDCDEDEACLHTLELNSPLIVNNRYASHIASFVLAWTRAFIS

EWSDFLYENDRGIP-IDKRPLKSLYGDTDSLFLTELGRSIMEQKGRHRIKKNGGKLVFDP

KNPDLTWLVECETKCIKCGKDAYSSESIFMAPKLYALKDITC--KFCKYVGPGKLRAKGH

PKTELSYETMLACY--LA-DIQQGN-D----HFQTSRLSLRRTLVTSMNNVQPFTVTQST

LTRTLRPWKDKTLHQV----NQHLLIPYSTSNPNPRNT-----------EVCWTELPWEN

------------------------------

>Murine AdV-A

MAQIVPAFGSSGIHRKSTDTNLNPSSITGGDSSSSTTTPDADAQSSSRTPAR--GRPRKK

AT-----------------VSSPKQKQQSKVLQKAHKTLRAAC-PRGDHMVIGYFTSYVV

PFTNLLCLQRCGPFPHPFLLDAFKAADIGHKSISVTSLTRLLQKEEWPAPVNYVW-YDGL

YIRQSKWPGLKGHAGRYPQVDKDASYEEKRWCWQNYRPELTFLHIQKGLGTKG---SNYH

KIGLYLVDTMHPCQRCEDCGSFFRFKHTCNARRRSFFFHNLKPLSRQWWTKISFTPLGSI

PTTKRLFIIYDLETYCWHGSCGKQLVPFMLVMELYGDMFLCAKAHSI-AKQMGYSEHH--

LRPY--ILWSINPQKEAIGRQFRRFRDRLQEAFAEELWQNNI--YPE--LQTYI-DSLPL

GLL-DLTPERLEQE--APPLKNTPKFIEVYVVGHNINGFDEIVVAAQVLNN-RVNFPPPL

-RVHRHFMPRNGRILFNDITFSLPNPRY---------KKR--TDFEQWEQGCLTTSDSKI

QYVKFMVRDTFALTH-TSLRKAAEAYSLPLEKGVCPYAAVNDFYMTGRYDTINIFDGFPH

RKYWNSEAEYEEGLQEWKKEKEELRKRCPDRVCPIKYNLIEHTLKYCIQDVTVTTKLVCK

LLDSYHHFIQNAVGLPKCLFNIFQRPTISANSHAIFKQTLY-------------------

-------SELRPEKPNFDDVLLAPSREMYDFVRQSIRGGRCYPTVLGELKEPIYVYDICG

MYASALTHPMPSGWPLEPKARAEALADWTKH--LSNSAP-ISYFNTCLLHGIVLIDADPP

CETQLDVLPPFCSRKGGRLCWTNEPLRGEITTTIDVITLHNRGWKVNIL-PEALTTLFPH

MKCLVRKYVTINITAKEKADRQKNMVMRSIAKLLSNALYGSFATRQDNKSTVFASQLYPG

TIK-EICTGRQKIKALHVVETDNLCA---------------EILTEFKTIYQPSCENTR-

-PPHVA-DSAELSPRGS-----------------------------HPAPFYRE----QQ

VVDHAL----FNFKPIKFLDAEADDLTLVTLEHSNPLVDNDRYASHLASFVLAWTRGFMS

EWSDILYESDRGLQ-LEKRLIKSVYGDTDSLFVTEAGRNLMESRGKHRLKKNGGKLVFNP

EKPSLTWLVECETQCKYCGSDAFSPRTVFLAPKLYALQKLVC--PTCGQEGAGKLRAKGH

AVASLSYDILLACY--EAYRLNESD-P----NFYTSRQSLKRTLVNMNKQESAFTVTETV

LTRSLRPWQNPTLALL----EDGRLIPYSTAHPNPRNR-----------ETTWIAL----

------------------------------

>Ovine AdV-D

M-----------------------------------------------------------

-------------------------ASKTIIKQRTTTYITGIY-ED-TYIKIIYYKNFEK

SFYNYFAIQGL-KLNSFK------------KVENVLDVLKLCDSNTPVSLKIWKF-NKRF

FKMTEE---------------------------ILNGN-----LLPIELLILK---GE--

---IYLIKTVKQQNKCEFCGTIYSNIHNCSSRRRDFYYHNIHHETKLWWEKIKFNPVGAI

-KAKRLFIVYDIETYTYHSLYGKQLTPYLLVFKLIGCKSLIKIASKI-ATDIGYE-----

VNRE--CFVMLNKKEDEIGIAFKKFRTELQISVAKKYWSTFCENHG---------LESNL

SYEDIMVLNKEKKL-----KQCEPRYIELYVVGHNICGFDEIILASHVLEGIDSEELSMF

-KLTRSFMPRAGKLLFNDITLSLPNPCF---------KKPSHTTYERWKNGIINFEDMKW

QGIKFMVRDTFLLTH-SSLRDAATAYQLSVSKGYCPYRAINDFFMLGEYEKEN--NGYPV

QKYWNSFEEYLQNK----PKHNQ------------KYNLLEEAKEYCIDDVIVTAQLVEK

LIEGYQEFCTTSLKL-ECSFNIFQRPTISSNTQALFKQIFY-------------------

-------NEEDHPSEFLRN-LEAPSEKMYDFVRMSLRGGRCYPSFLGIFEEAIYVYDICG

MYASALTHPLPYGKTLNAFEANAQIDYFQEL--LQRKEK-IDYFDNSIKPMIVVADCEPP

SLDYLDVLPPLCSKKSGKLCWSNETLINEVLTSIDLITLHNRGWKCKIIKSSEMYAVWSD

WKPLCQKYVKVNIMAKEKASKSNNKIQRSISKLLSNALYGSFATRIDKKKVVFAENIEEK

DKK-RLENGSAEITSYTTVISKSLPK---------------RSF-EWNQYFSNLPEMPP-

-------KDSKDTNENF-----------------------------NFPLFIGN------

-QDHVT------FKPITFLSADCNDLILTTIEDKEEWIKNNRYPTQIASFVLAWTRAFMS

EWAEILYGEDMGKP-YIEREIKSIYGDTDSLFLTEKGHQLMLSKGLHRLKKYNSNLIFDE

KHPCLTWLVECETVCNRCGAEAFSSETCILAPKLYALKDITC--KNCKFIGEGKLRAKGH

AKNCLNYEILKHCFTDYN-LLEQPQ-K----EFQTSRKSLKKTLQTASGTDVPFTVVEKQ

LTRILRPWKDQTMRKGIQWKKGYLLYPYDKRHPNPRPQ-----------EPLTENPFWEN

F-----------------------------

>Frog-AdV

MSAF--------------------------------------------------------

---------------------------GSIENDHFRLKLNSKP-AKLAILDICWQFNLFN

CKKFKHKKHIA-SLIYSQ-----------CSYNSNKSLYELCKTLPIKTLQLWKI-QKGS

IIRTQK--------------------------LDLDQG-----EIIIDLLSPK-------

---HLWIVEWFEQNKCKNCGRFFSYNHVCNTNRASYYYHKI-ANTKTQWETINFSPIGEP

KGTKKLFLVYDIETYTKACPVGYKLCPVLLCFTIFGDETLNKEVHELISSNSNILELN--

NLGE--CFYWLSKETNYISSKFRKTRLLIQNFLIEKMIKNFLTEENKEILTDYA-KAHNY

ESIYEIDLLKDKNL--ILTLQAPFINLEFYVIGHNISSFDEILLAHQILQKDTTTIPQPF

IQIKRNFLPRQGKILFNDITFSLPYPEYYVHEEENTLTKK--EAIEIEKTGEPSLFRTKE

ICVKTMVRDTYQLTH-CSLRLAAEAYGLKTTKGNCPFKAVTEFYSVNSFDKDK--FGFPA

PKYWTDNEEYMTQLKIWQQNDE-------------PYDLQKELIKYCAQDVNVTKNLTIS

ILNSYNEFIKENFKL-TCNYNVFKRPTIPSNSQAIFKQLLY-------------------

-------KKHGKKFGKMPD-IMAPSAEMYEFVRESVRGGRCYPSLLGEIKEPIYVYDICG

MYASALTHPMPFGVPLSQKEKNQEIHILQSK--LQNEKT-LNYFDPEIKPMIISISAFPP

PVEYLTNIPPICSRKSGRLCWTNEALYDETVTIVDVITLHNCGWKVKIE-DHPLNTVFPN

WNTCCKEYVEVNIAAKEKADKDKNQIMRSISKLLSNSLYGSFATKEDNSMIVFEHMLKSN

HIKTKLEQNEIKIDAVFGIPTNGIEH---------------TTIDNKLFYLSNTNTLND-

-------SNAPPIDDEL-----------------LTPFSEEAYAE-EEVEQLAP----EN

SNCHVR-----TYQPFTLIDVDTEDLTLYSLKSNKKFTENKRYPTQLASFVLSWTRAFMA

DWNAILYEKDSKSI-LV-KELNAIYGDTDSLFLTEKGHKRMIDHGQHRLKENVKSLVFQE

CCPDITWAVECETKCPNCKSSAFSNRTIFLAPKLYALKRIVC--NSCNTETEGKLRAKGH

AKNDMTFELLCTCFEYHK-NCHTKG-K----RFSTERTALKRTLYKPYGKFEPFTIHQVR

LIRELRPWHDPTMLER----DDGFLIPYYVGHPNPRDK-----------RVKYLE-----

------------------DIDG--------

>Fowl AdV-A

M-----------------------------------------------------------

------------------------------------------------------------

------------------------------------------------------------

-------------------------------------------------LIAKNVTGE--

---WVWITSRTPVQQCPTCGRHWVRRHSCNERRSAFYYHAVQGSGSDLWQHVHFSCPAQH

PHIRQLYITYDIETYTVFEKKGKRMHPFMLCFMLSGDPQLVSRAERL-ARQD--DRLK--

ALDE--GFYWLDSHPGEVARRFRNFRSRLQIEFAQNLVDRYAAANRD--YCDQLVKDGKY

GSVHKIPYELFEKPTSPLSLPDNFYSVDIVVLGHNICKFDELLLATELVER-RDLFPEAC

-KCDRSFMPRVGRLLFNDIIFRMPNPNY---------VKKDASRVERWSRGIVSHQDARS

VFVRFMVRDTLQLTSGAKLSKAAAAYALDLCKGHCPYEAINEFISTGRFHADA--DGFPV

ERYWEDPSVIAEQKNLWQKEHPGQ-----------QYDIVQACLEYCMQDVRVTQKLAHT

LHDSYDAYFQRELGM-EGHFNIFVRPTIPSNTHAFWKQLTFSNYVREQRATCPPSVPEPP

KKKGRTKKKKQPSPDYVAE-VYAPHRPMFKYIRQALRGGRCYPNVLGPYLKPVYVFDICG

MYASALTHPMPHGMPLDPKFTAQHVEELNRL--LTNESH-LSYFDARIKPSILKIEAYPP

PPEMLDPLPPICSRRGGRLVWTNEALYDEVVTVIDILTLHNRGWRVQVL-HDEMNIVFPE

WKTLCADYVTKNILAKEKADREKNEVIRSISKMLSNALYGAFATNMDTTRIIFEQDLSEA

DKK-NIYEGTEIVKHVTLLNDDSFNGTEVTLENAPNPFSEESLRQQFRYADDPEQE----

-EPEAE-EDGEEEGDDSDRESARKPKNALTEDDPLVAVDLEVEATLATGPYIPEG---EL

SSAHYARANETRFKPMRLLEATPEALTVLHLESLDKQVANKRYATQIACFVLGWSRAFFS

EWCDILYGPDRGVHILRREEPRSLYGDTDSLFVTETGYHRMKSRGAHRIKTESTRLTFDP

ENPGLYWACDCDIKCKACGSDTYSSETIFLAPKLYGLKNSICVNEQCRTVGPGKIRSKGH

RQSELIYDTLLRCWRRHE-DVQFGA-QSNIPELHTRRTIFKTTLLNKVSRYDPFTIHNEQ

LTRVLRPWKDLTLYEH----GDY-LYPYDNEHPNPRTTGDVRPVPIVGHEDPLAPLRWEP

YAFLSEEECGQVHDLLFADDSSQEAESLGV
